# Site-selective redox isomerizations of furanosides using a combined arylboronic acid/photoredox catalyst system†

**DOI:** 10.1039/c9sc05173b

**Published:** 2020-01-03

**Authors:** Victoria Dimakos, Daniel Gorelik, Hsin Y. Su, Graham E. Garrett, Gregory Hughes, Hiromitsu Shibayama, Mark S. Taylor

**Affiliations:** Department of Chemistry, University of Toronto 80 St. George St. Toronto ON M5S 3H6 Canada mtaylor@chem.utoronto.ca; Global Process Chemistry, Merck Research Laboratories P. O. Box 2000 Rahway NJ 07065 USA

## Abstract

In the presence of an arylboronic acid and a hydrogen atom transfer mediator under photoredox conditions, furanoside derivatives undergo site-selective redox isomerizations to 2-keto-3-deoxyfuranosides. Experimental evidence and computational modeling suggest that the transformation takes place by abstraction of the hydrogen atom from the 2-position of the furanoside-derived arylboronic ester, followed by C3–O bond cleavage *via* spin-center shift. This mechanism is reminiscent of the currently accepted pathway for the formation of 3′-ketodeoxynucleotides by ribonucleotide reductase enzymes.

## Abstract 2

The combined action of boronic acid, photoredox catalyst and hydrogen atom transfer mediator enables the transformation of furanosides to 2-keto-3-deoxyfuranosides, a synthetic analog of the process catalyzed by the ribonucleotide reductase enzymes.

## Graphical abstract

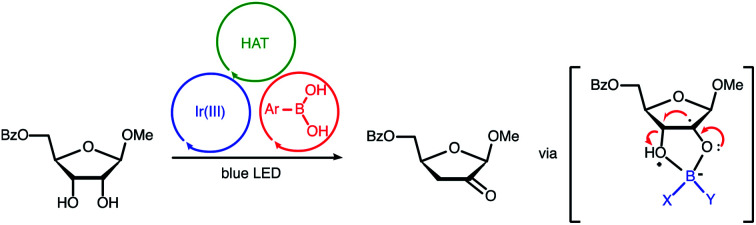

## Introduction

**Scheme 1:**
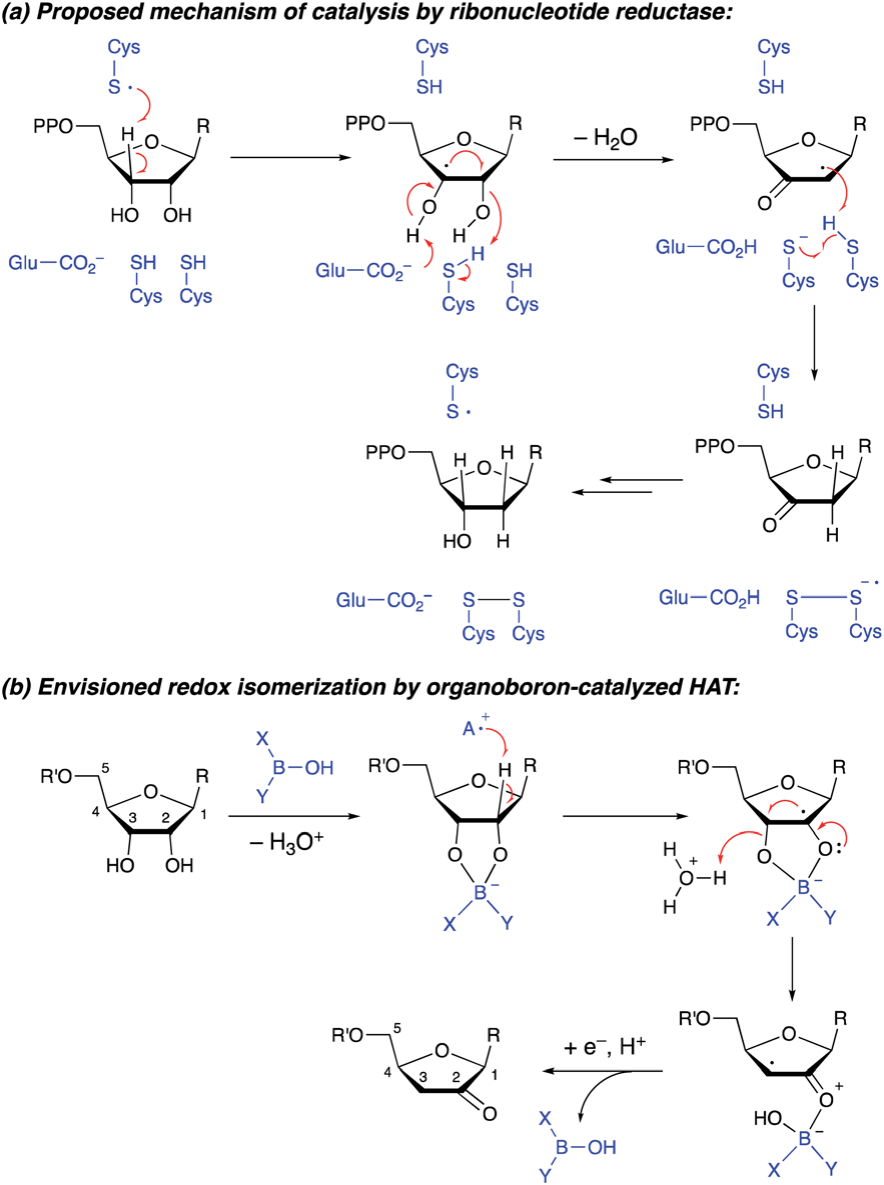
(a) Conversion of furanosides to ketodeoxyfuranosides by ribonucleotide reductase. (b) Envisioned organoboron-catalyzed redox isomerization.

The transformation of ribonucleotides to 2′-deoxyribonucleotides, catalyzed by ribonucleotide reductase enzymes, is a crucial process in DNA biosynthesis and repair. Detailed mechanistic studies have pointed towards a pathway involving abstraction of the 3′-hydrogen atom by an enzyme-derived radical, followed by dehydration and delivery of a hydrogen atom to the 2′-position, generating a 3′-ketodeoxynucleotide (Scheme 1a).^1^ Reduction of the latter intermediate delivers the 2′-deoxynucleotide. Synthetic methods for conversion of furanosides to ketodeoxynucleotides are of interest, both from the perspective of fundamental reactivity development and as a way to prepare useful derivatives of furanosides, nucleosides or nucleotides.^2^ Whereas furanosides equipped with a leaving group at the 2- or 3-position can be transformed to 3- or 2-ketodeoxy derivatives (by semipinacol-type rearrangement^3^ or by elimination to an enol derivative^4^), protocols that capitalize on radical reactivity to achieve redox isomerization of the diol group in a furanoside substrate have not been reported. Here, we show that the combined action of organoboron catalysis and photoredox hydrogen atom transfer (HAT) catalysis enables the synthesis of 2-ketodeoxyfuranosides from the corresponding furanosides. Although the regiochemical outcome differs from that of the enzyme-catalyzed process, the proposed mechanism is reminiscent of that of the ribonucleotide reductases, with the organoboron catalyst playing key roles in accelerating HAT from the furanoside as well as the dehydration of the resulting radical intermediate. The protocol provides access to useful furanoside derivatives that are challenging to generate by other means.

We recently showed that a diarylborinic acid co-catalyst could be used to achieve site-selective and stereoselective C–H alkylations of pyranoside derivatives in the presence of an iridium(iii) photocatalyst and quinuclidine as an HAT mediator.^5,6^ Computational modeling suggested that the formation of a tetracoordinate borinic ester at a *cis*-1,2-diol group served both to weaken the α-C–H bonds and to accelerate HAT with the electron-deficient quinuclidine radical cation through polarity-matching and/or ion-pairing effects.^7,8^

We hypothesized that the ability to activate a carbohydrate derivative towards radical formation at a particular site could be exploited to achieve the transformation of furanosides to ketodeoxysugar derivatives (Scheme 1b). Considering the known activity of boronic acids as catalysts for substitution and rearrangement reactions of alcohols,^9,10^ we anticipated that organoboron complexation could promote C–O bond cleavage, accelerating the dehydration of the radical intermediate. Other important precedent for this proposal includes: (i) MacMillan and co-workers' discovery that benzylic alcohols can be employed as alkylating agents under photoredox conditions, *via* elimination of water from a radical intermediate (spin-center shift);^11,12^ and (ii) detailed studies of the reactivity of nucleoside-derived radicals.^13^

## Results and discussion

**Scheme 2:**
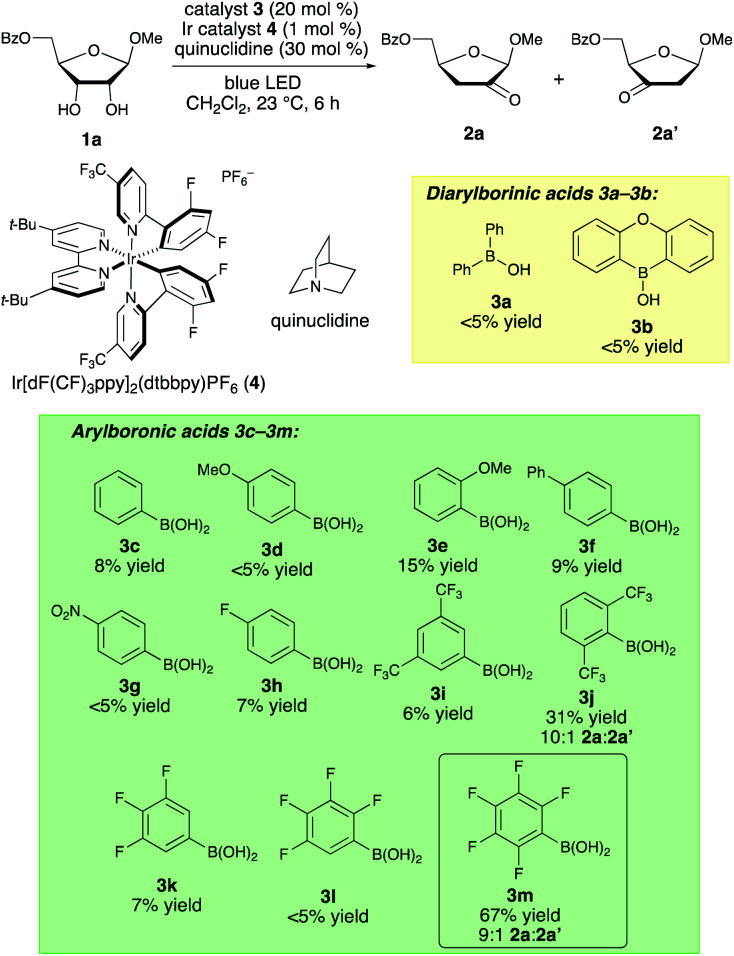
Evaluation of catalysts for the isomerization of ribofuranoside **1a** to 2-keto-3-deoxyfuranoside **2a**. Yields were determined by ^1^H NMR spectroscopic analysis of unpurified mixtures using a quantitative internal standard.

Structurally diverse organoboron acids (**3a–3m**) were evaluated as catalysts for the redox isomerization of β-ribofuranoside **1a** in the presence of Ir(iii) photocatalyst **4** (ref. 14) and quinuclidine as HAT mediator (Scheme 2).^8*a*^ The catalyst structure–activity relationships for this transformation were markedly different from those of our previously reported C–H alkylation reaction of carbohydrates;^5^ diphenylborinic acid **3a**, the optimal co-catalyst for the latter reaction, was inactive, as was heterocyclic diarylborinic acid **3b**,^15^ but the use of phenylboronic acid led to the formation of 2-keto-3-deoxyfuranoside **2a** in 8% yield. Thus, diarylborinic acid catalysis is useful for promoting the coupling of the radical with an electron-deficient alkene *via* C–C bond formation, whereas arylboronic acid catalysis enables dehydration of a similar intermediate through C–O bond cleavage. Evaluation of a series of substituted arylboronic acids led to the identification of perfluorinated **3m** as the optimal catalyst for this transformation. The structure–activity relationships for **3c–3m** indicate that the best-performing catalysts possess relatively high acidities^16^—perhaps causing acceleration of the C–O bond-breaking step^9^—and that *ortho*-substitution tends to have a beneficial effect (*e.g.*, catalysts **3e**, **3j** and **3m**). The results of additional optimization experiments illustrating the effects of changing the solvent, HAT mediator and catalyst loadings, and control experiments demonstrating the importance of each element of the catalyst system, are provided in the ESI.†

**Scheme 3:**
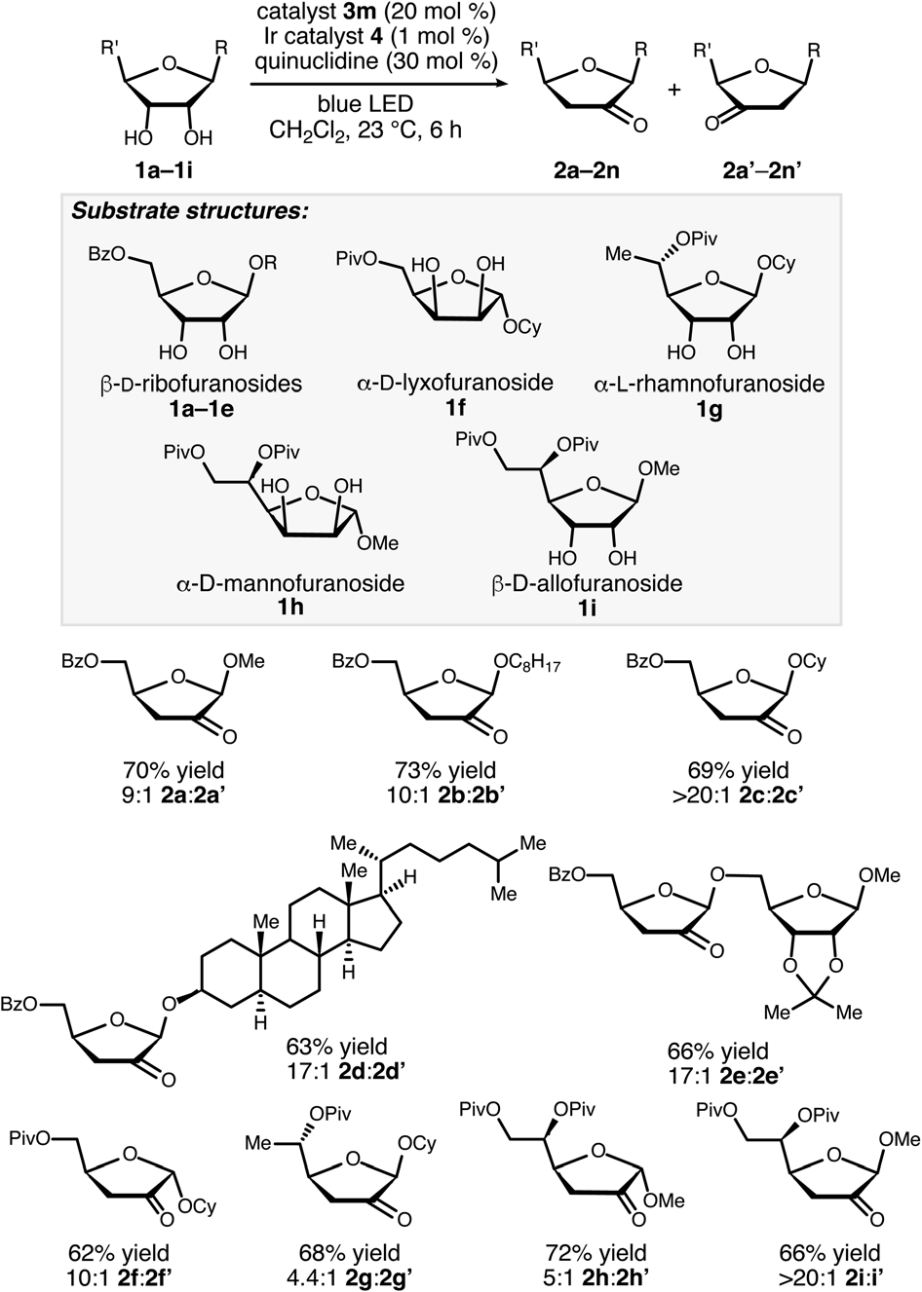
Redox isomerizations of furanosides to 2-keto-3-deoxyfuranosides. Combined yields of the 2-keto and 3-ketofuranoside products after purification by chromatography on silica gel are reported. Regioisomer ratios were determined by ^1^H NMR spectroscopic analysis of unpurified reaction mixtures.

A noteworthy aspect of the transformation of **1a** to **2a** is the regioselective formation of the 2-keto-3-deoxyfuranoside, in contrast to the pattern of selectivity observed for the ribonucleotide reductase-catalyzed process. The isomeric 3-keto-2-deoxyfuranoside **2a′** was observed as a minor side product in the reactions catalyzed by **3j** and **3m** (quinuclidine-mediated aldol dimerization of the ketodeoxysugar product was another observable side reaction – see the ESI for details†). Selectivity for the 2-keto-3-deoxy derivative, which presumably arises from hydrogen atom abstraction from the 2-position, was observed for a variety of furanoside substrates having *cis*-configured 2-OH and 3-OH groups, including α-lyxofuranosides, α-rhamnofuranosides, α-mannofuranosides, and β-allofuranosides (Scheme 3).^17^ Variation of the aglycon in β-ribofuranoside substrates was tolerated, including steroidal glycoside **1d**.

**Scheme 4:**
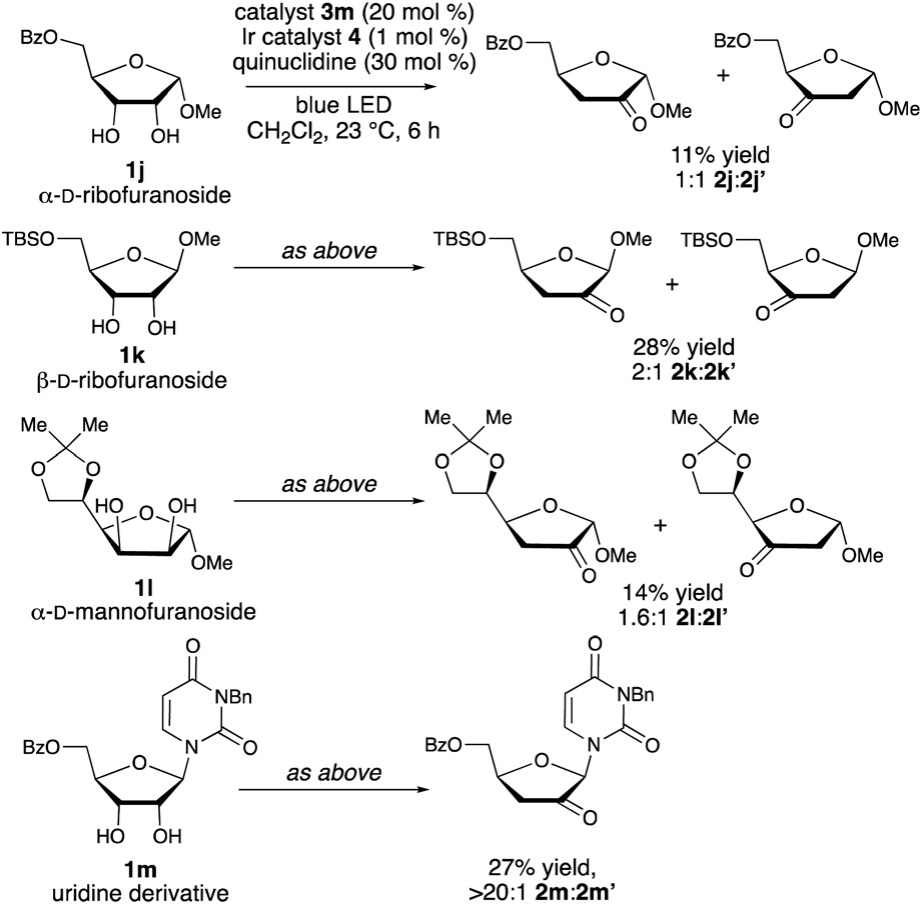
Limitations of the boronic acid/photoredox-catalyzed redox isomerization reaction. For **2k** and **2l**, yields were determined by ^1^H NMR spectroscopy. Regioisomer ratios were determined by ^1^H NMR spectroscopic analysis of unpurified reaction mixtures.

As shown in Scheme 4, the configuration and substitution pattern of the furanoside substrate influenced the efficiency and selectivity of the redox isomerization reaction. In contrast to the successful isomerizations of the β-ribofuranoside substrates, the reaction of α-ribofuranoside **1j** was low-yielding and generated a 1 : 1 mixture of the 2-keto and 3-ketosugar products, indicating that a 1,2-*trans*-configuration is important for site-selectivity in the HAT step. We note that a product having this configuration can be accessed from the corresponding α-d-lyxofuranoside (Scheme 3, **1f** → **2f**). The nature of the protective group at the 5-position is also important: mixtures of isomers were obtained in low yields from 5-*O*-silylated ribofuranoside **1k** and 5,6-*O*-isopropylidene mannofuranoside **1l**. We propose that the inductive effects of the C5-substituents influence the relative rates of hydrogen atom abstraction from C-3 *versus* C-2, with the electron-withdrawing acyl protective groups deactivating the 3-position. *N*-Benzyluridine derivative **1m** was transformed to ketodeoxy derivative **2m** in a regioselective fashion, but in low yield. Loss of 3-benzyluracil, presumably *via* a competing spin-center shift from the C-2 radical,^13*b*^ was the major side reaction. Substrates that did not undergo redox isomerization under the optimized conditions include an α-arabinofuranoside, which lacks the *cis*-1,2-diol group needed for binding to the boronic acid catalyst, a C-aryl β-ribofuranoside and a fucopyranoside (see the ESI†).

**Scheme 5:**
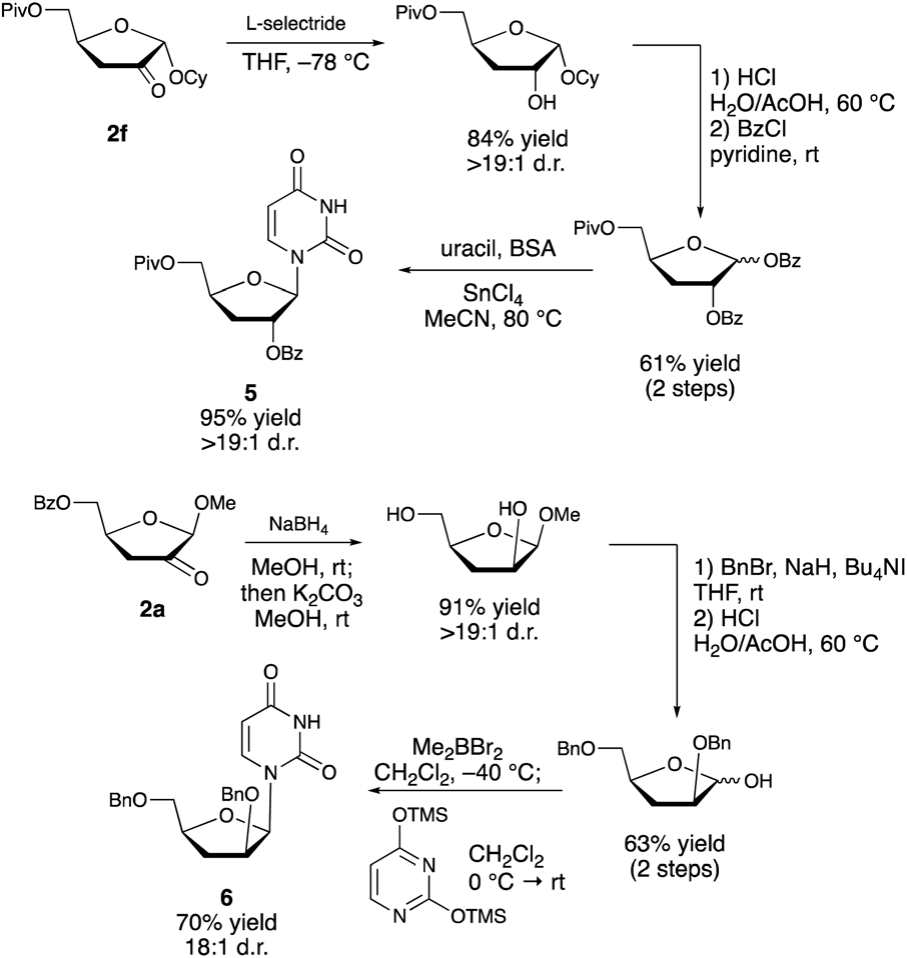
Synthesis of uridine derivatives from alkyl 2-keto-3-deoxyfuranosides.

Although protocol is presently limited in scope to a rather narrow class of substrates (furanosides having a *cis*-diol group at C2/C3 and a *trans* relationship between the anomeric substituent and the 2-OH group), it provides direct access to useful compounds that are challenging to generate by other means. Approaches based on semipinacol-type rearrangements^3^ require the site-selective introduction of a leaving group at the 3-position. This is challenging to achieve directly^18^—organotin-promoted sulfonylations of nucleosides are C2-selective, for example^19^—and necessitates multistep protection/deprotection sequences. Deoxygenation of furanosides has been achieved by conventional means (*e.g.*, the Barton–McCombie protocol), but a site-selective variant^20^ has not been reported for such substrates. Furthermore, the transformation of the deoxy product to the corresponding ketodeoxy derivative would require an additional oxidation step. One of the most important applications of such ketodeoxyfuranosides is in the synthesis of nucleoside analogs, an important class of therapeutic agents. While additional optimization is needed to achieve the direct redox isomerization of a nucleoside derivative using the boronic acid/photoredox catalyst system (**1m** → **2m**, Scheme 4), the alkyl furanoside-derived products can be employed in the stereoselective synthesis of such targets (Scheme 5). Thus, diastereoselective reduction of the carbonyl group of compound **2f** (presumably dictated by the steric effect of the α-configured anomeric substituent),^21^ followed by hydrolysis of the glycoside and bis-benzoylation of the resulting hemiacetal, allowed for Lewis acid-catalyzed *N*-glycosylation to deliver protected 3-deoxyribonucleoside **5** in 95% yield. Product **6**, having the arabino configuration, was accessed from **2a***via* one-pot diastereoselective reduction and ester saponification, bis-*O*-benzylation, glycoside hydrolysis and 1,2-*cis*-selective *N*-glycosylation under the conditions developed by Guindon and co-workers.^22^ We anticipate that the 2-keto-3-deoxyfuranosides will provide access to a variety of other nucleoside analogs by carbonyl addition^23^ and enolate functionalization reactions, and by installation of other anomeric substituents. In cases where analogs bearing a canonical nucleobase are needed, the direct transformation of the corresponding protected nucleosides (*e.g.*, **1m** → **2m**, Scheme 4) may be the most efficient option, particularly if the yield can be improved through further optimization.

**Scheme 6:**
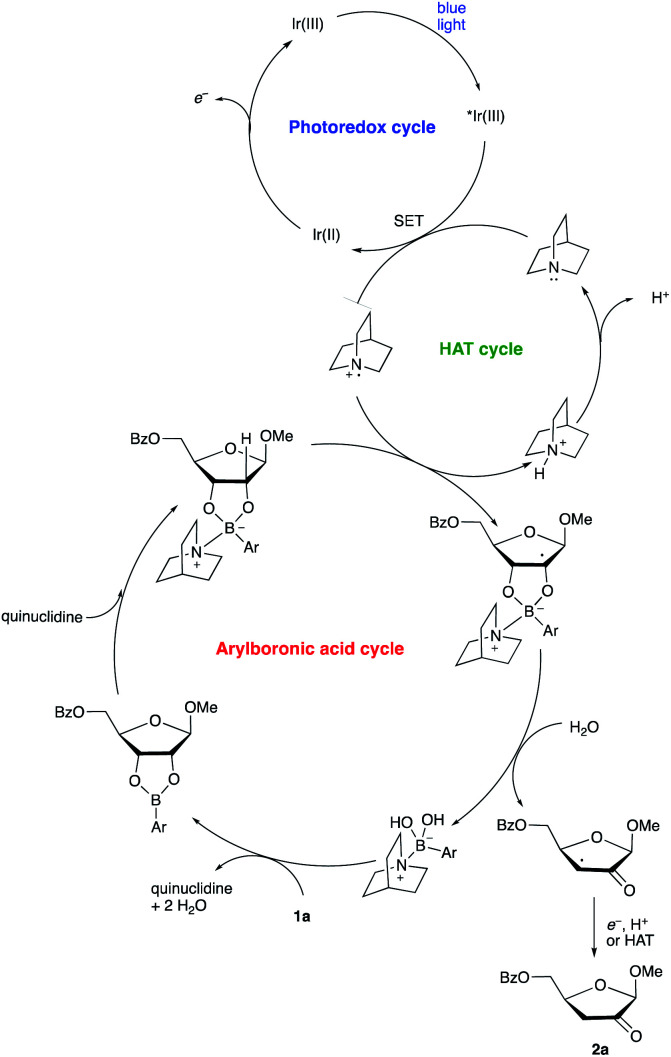
Proposed co-catalytic cycle.

A proposed co-catalytic cycle for the redox isomerization is depicted in Scheme 6. In line with previous mechanistic proposals,^8^ electron transfer to the photoexcited Ir complex serves to generate the quinuclidine radical cation. The latter engages the tetracoordinate quinuclidine–arylboronate complex *via* abstraction of the hydrogen atom at the 2-position, a kinetically favorable process due to the increased hydridic character of the α-C–H bonds.^5,7^ The resulting boronate-derived radical undergoes C3–O bond cleavage, releasing an α-keto radical that is subject to reduction and protonation (closing the photocatalytic cycle) or HAT (propagating a radical chain) to deliver the 2-keto-3-deoxyfuranoside product.

**Scheme 7:**
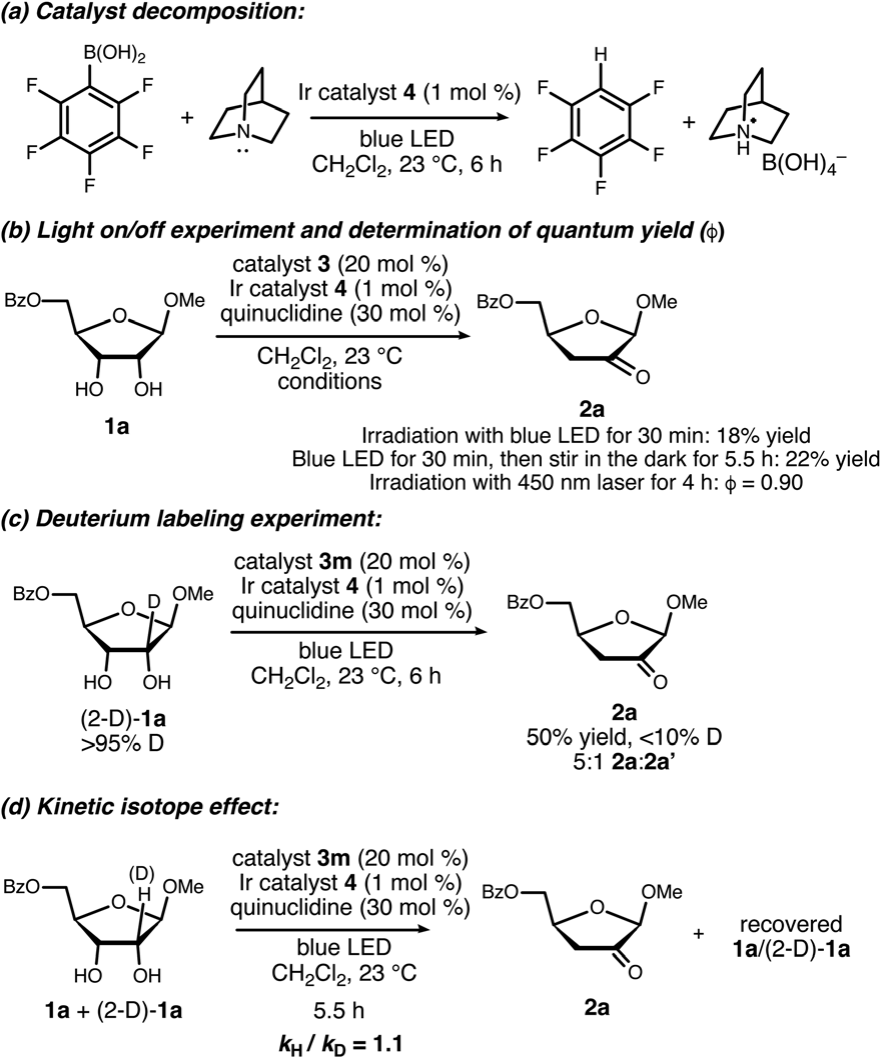
Experiments relevant to the mechanism of catalysis.

Results of experiments relevant to the mechanism of catalysis are summarized in Scheme 7. Upon consumption of the furanoside starting material, the formation of a precipitate identified as quinuclidinium borate was observed, implicating C–B bond cleavage as a catalyst decomposition pathway. Control experiments showed that this decomposition takes place upon irradiation of **3m** in the presence of quinuclidine and complex **4**. The formation of a perfluoroaryl radical *via* reduction of the quinuclidine–**3m** complex is likely involved.^24^ This catalyst decomposition pathway may be important to keep in mind when interpreting structure–activity relationship data and in further optimization studies.

Ceasing irradiation with the blue LED while the reaction of **1a** was in progress largely halted the formation of the ketodeoxy product (18% yield of **2a** after irradiation for 30 minutes, *versus* 22% yield after irradiation for 30 minutes followed by stirring in the dark for 5.5 hours). However, the quantum yield (*ϕ*) for the photoredox process using a 450 nm laser was determined to be 0.90.^25^ The fraction of quenching of the excited Ir(iii) complex by quinuclidine was estimated to be 0.4–0.7, depending on the concentration of free quinuclidine *versus* the quinuclidine–boronate complex under the reaction conditions. Taking the quenching fraction into consideration, the value of *ϕ* is inconsistent with a closed photoredox cycle, pointing towards radical chain processes with average chain lengths on the order of 1.3–2.2.

When deuterated furanoside (2-D)-**1a** was irradiated in the presence of catalytic **3m**, **4** and quinuclidine, the isomerization took place in diminished yield and site-selectivity in comparison to the non-deuterated isotopolog (50% yield, 5 : 1 **2a** : **2a′**). The deuterium content of the product was less than 10%; H/D exchange at the 3-position of **2a** was observed under the reaction conditions (see the ESI†). An intermolecular competition experiment between **1a** and (2-D)-**1a** gave an apparent KIE of 1.1 based on the deuterium content of the recovered starting material. Although the observation of a primary kinetic isotope effect in this type of experiment would not imply that the C–H cleavage step is turnover-limiting, its absence does rule out this possibility.^26^

**Scheme 8:**
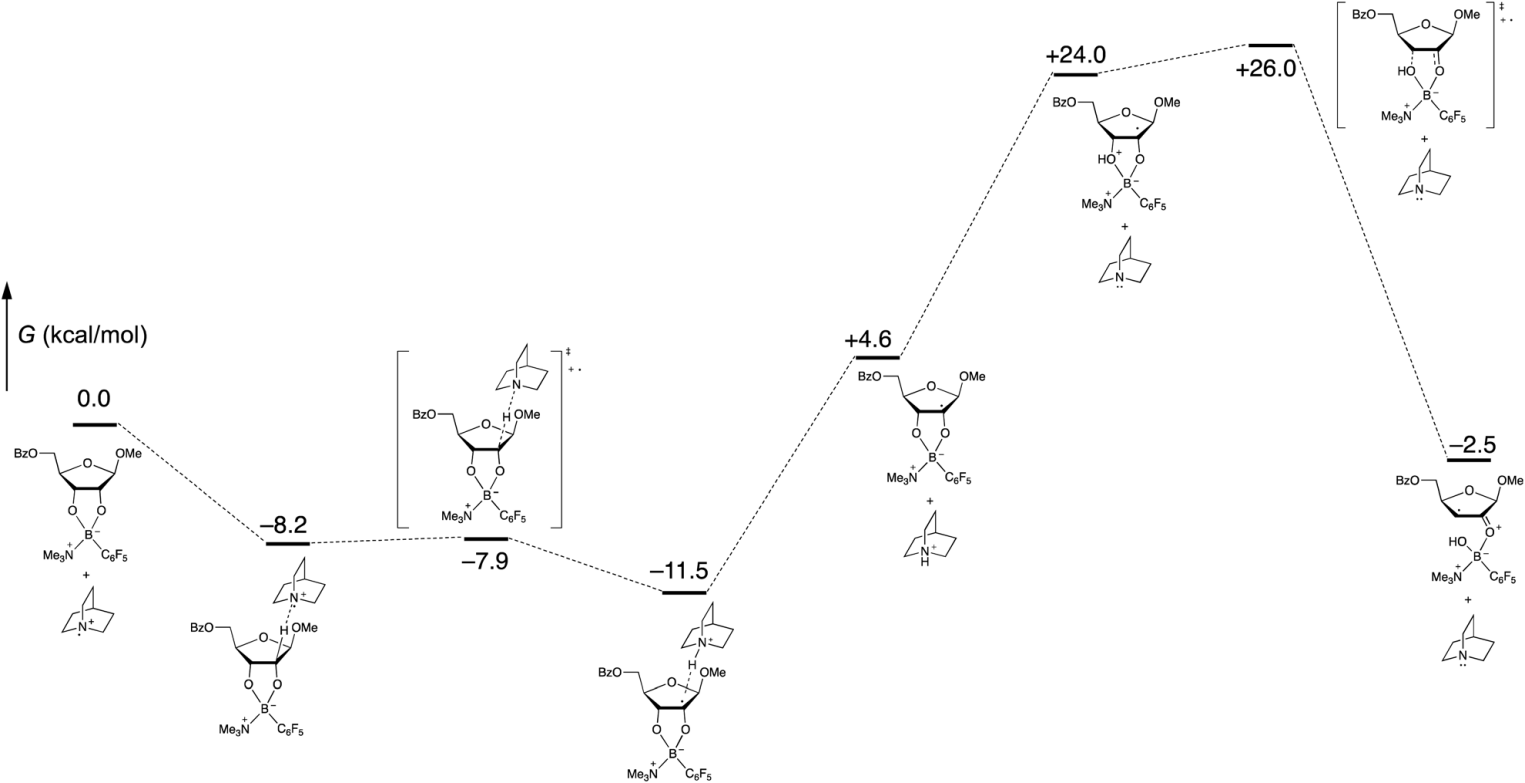
Calculated free energies of proposed organoboron intermediates and transition states (B97-D3/Def2-TZVP, gas phase).

Computational modeling with density functional theory (B97-D3/Def2-TZVP, implemented with Gaussian 16)^27–29^ was used to assess the feasibility of the organoboron-mediated steps of the proposed catalytic cycle. To reduce the number of basis functions, trimethylamine was used as a surrogate for quinuclidine in the tetracoordinate organoboron adduct. Gas-phase geometry optimizations and frequency calculations were conducted, enabling identification of intermediates and transition states corresponding to the proposed pathway, including the key spin-center shift step (Scheme 8). Intermolecular interactions involving the proposed radical cation intermediates were observed; for example, the formation of a pre-reactive complex from the quinuclidinium radical cation and the tetracoordinate boronate was calculated to be exergonic by 8.2 kcal mol^−1^. Whether such interactions are relevant to the mechanism of the solution-phase process (rather than in the gas phase) is not clear at this stage. In any case, the free energy profile, which implies that the proton transfer and spin-center shift steps are turnover-limiting, appears to be qualitatively consistent with the low intermolecular competition deuterium kinetic isotope effect of 1.1 at the 2-position (Scheme 7).

**Fig. 1:**
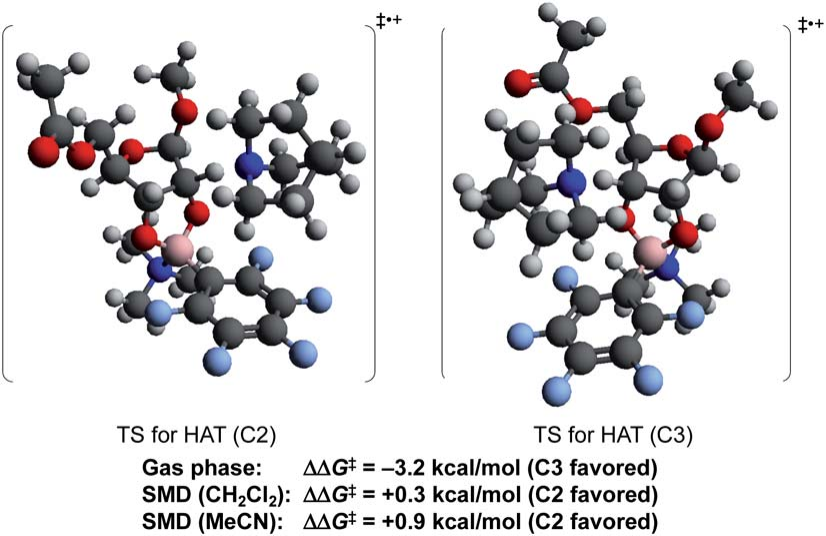
Calculated transition state energies for HAT from C-2 and C3 (B97-D3/Def2-TZVP).

To probe the origin of site-selectivity, the transition state energies for HAT from C-2 and C-3 of the tetracoordinate boronate were compared (Fig. 1). In the gas phase, HAT from C-3 (leading to the minor isomer **2a′**) was calculated to be kinetically preferred over C-2 by more than 3 kcal mol^−1^. However, the use of the SMD model for CH_2_Cl_2_ gave a difference in free energies of activation (ΔΔ*G*^‡^) of 0.3 kcal mol^−1^ favoring HAT from the 2-position, in qualitative agreement with the experimentally observed result. The corresponding ΔΔ*G*^‡^ value in acetonitrile was calculated to be 0.9 kcal mol^−1^; we note that a marginally higher selectivity—12 : 1 **2a** : **2a′** in acetonitrile *versus* 9 : 1 in dichloromethane—was obtained experimentally, although the yield of the redox isomerization was low in the former solvent (see the ESI†). The results suggest that the transition states for HAT from C-2 and C-3 show distinct charge distributions, with moderately polar solvents playing a role in favoring the pathway that leads to the 2-keto-3-deoxyfuranoside.

## Conclusions

In conclusion, the merger of boronic acid catalysis and photoredox/HAT catalysis has enabled a direct transformation of furanosides to 2-keto-3-deoxyfuranosides. The formation of a boronic ester from a *cis*-1,2-diol group is proposed to accelerate the abstraction of the hydrogen atom from the 2-position (*via* a tetracoordinate amine–boronate complex) as well as the cleavage of the C3–O bond to trigger the key spin-center shift step. It is noteworthy that this combination of synthetic catalysts is able to emulate aspects of the proposed mechanism of action of the ribonucleotide reductase enzymes, but shows a distinct pattern of site-selectivity. The products are useful precursors to modified carbohydrate and nucleoside derivatives. The results further illustrate the utility of organoboron catalysts in triggering the site-selective formation of radicals from carbohydrate substrates.

## Conflicts of interest

There are no conflicts to declare.

## Supplementary Material

SC-011-C9SC05173B-s001
