# Supplementary material for: Site-selective redox isomerizations of furanosides using a combined arylboronic acid/photoredox catalyst system
Source: Chem Sci. 2020 Jan 3;11(6):1531–7. doi: 10.1039/c9sc05173b (PMC8148048; doi:10.1039/c9sc05173b)

Electronic Supplementary Material (ESI) for Chemical Science.
This journal is © The Royal Society of Chemistry 2019

Electronic Supplementary Information

Site-selective redox isomerizations of furanosides using a combined arylboronic acid/ photoredox catalyst system

Victoria Dimakos,[†,§] Daniel Gorelik,[†,§] Hsin Y. Su,[†] Graham E. Garrett,[†] Greg Hughes,[‡] Hiromitsu Shibayama[†] and Mark S. Taylor*[†]

[†] Department of Chemistry, University of Toronto, 80 St. George St., Toronto, ON M5S 3H6, Canada
[‡] Global Process Chemistry, Merck Research Laboratories, P. O. Box 2000, Rahway, NJ 07065, United States

Table of Contents

General Information **3**

Materials 3

Instrumentation 3

Preparation of Substrates **4**

General Procedure A 4

General Procedure B 5

1a –Methyl 5-O-benzoyl β-D-ribofuranoside 7

(2-D)-1a –Methyl 5-O-benzoyl β-D-[2- $^{2}H_{1}$] ribofuranoside 7

1b –Octyl 5-O-benzoyl β-D-ribofuranoside 8

1c –Cyclohexyl 5-O-benzoyl β-D-ribofuranoside 8

1d –Dihydrocholesteroyl 5-O-benzoyl β-D-ribofuranoside 9

1e –Methyl 2,3-O-isopropylidene-5-O-(5’-O-benzoyl β-D-ribofuranoside)- β-D-ribofuranoside 9

1f –Cyclohexyl 5-O-pivaloyl α-D-lyxofuranoside 10

1g –Cyclohexyl 5-O-pivaloyl α-L-rhamnofuranoside 10

1h –Methyl 5,6-di-O-pivaloyl α-D-mannofuranoside 11

1i –Methyl 5,6-di-O-pivaloyl β-D-allofuranoside 11

1m –5’-O-pivaloyl-3-benzyluridine 12

Reaction Optimization **13**

Optimization Procedure 13

Table S1: Optimization of reaction conditions 13

Tabe S2: Control reactions 14

Table S3: Effects on the ratio of boronic acid to quinuclidine on the yield of 2a 15

Isomerization of Furanosides to 2-Keto-3-deoxyfuranosides **16**

General Procedure C 16

General Procedure D 16

General Procedure E 16

2a –Methyl 5-O-benzoyl-2-keto-3-β-D-deoxyribofuranoside 17

2b – Octyl 5-O-benzoyl-2-keto-3-β-D-deoxyribofuranoside 18

2c – Cyclohexyl 5-O-benzoyl-2-keto-3-β-D-deoxyribofuranoside 19

2d – Dihydrocholesteroyl 5-O-benzoyl-2-keto-3-β-D-deoxyribofuranoside 20

2e – Methyl 2,3-O-isopropylidene-5-O-(5’-O-benzoyl 2-keto-3-deoxy-β-D-ribofuranoside)- β-D-ribofuranoside 20

2f – Cyclohexyl 5-O-pivaloyl 2-keto-3-deoxy-α-D-lyxofuranoside 21

2g – Cyclohexyl 2-keto-3-deoxy-α-L-rhamnofuranoside 22
2h – Methyl 5,6-di-O-pivaloyl 2-keto-3-deoxy-α-D-mannofuranoside 23
2i – Methyl 5,6-di-O-pivaloyl 2-keto-3-deoxy- β-D-allofuranoside 24
2m –5’-O-pivaloyl-2-keto-3-deoxy-*N*^{3}-benzyluridine 24
Synthesis of Uridine Analogs from 2-Keto-3-deoxyfuranosides 26
S1 – Cyclohexyl 3-deoxy 5-*O*-pivaloyl α-D-ribofuranoside 26
S2 – 1,2-*O*-Benzoyl 3-deoxy 5-*O*-pivaloyl D-ribofuranoside 27
5 – 1-(β-D-2'-*O*-Benzoyl-3’-deoxy-5'-*O*-pivaloylribosyl)uracil 27
S3 – Methyl 3-deoxy β-D-arabinofuranoside 28
S4 – Methyl 2,5-di-*O*-benzyl 3-deoxy β-D-arabinofuranoside 29
S5 – 2,5-Di-*O*-benzyl 3-deoxy D-arabinofuranose 30
6 – 1-(β-D-2',5'-Di-*O*-benzyl-3’-deoxyarabinosyl)uracil 30
Additional investigations into substrate scope 32
General Procedure F 32
Quantum Yield Determination 33
Preparation of Ferrioxalate and Buffered Phenanthroline Solutions 33
Characterization of the Ferrioxalate Solution 33
Characterization of the Iridium Catalyst 33
Determination of Photon Flux 34
Determination of Quantum Yield 36
Stern-Volmer Data and Chain Length Approximations 37
Kinetic Isotope Effect Experiments 39
H/D Exchange Experiment 40
Computational Data 41
Table S4. Calculated energies of reaction intermediates depicted in Figure S5 and S6 43
Table S5. Calculated energies of transition states for HAT from C-2 versus C-3 in acetonitrile 44
Table S6. Calculated energies of transition states for HAT from C-2 versus C-3 in dichloromethane 44
References 74
^{1}H, ^{13}C and 2D NMR Spectra 75

General Information

Materials

Stainless steel needles and syringes were used to transfer air and moisture-sensitive liquids. Schlenk flasks were dried at 140 °C for at least 24 hours prior to use. Dichloromethane was HPLC grade and purified using a solvent purification system equipped with columns of activated alumina under nitrogen (Innovative Technology, Inc.). 4 Å molecular sieves were stored for at least 24 hours at 140 °C prior to use. Other reagents and solvents were used without further purification. Flash column chromatography was carried out using neutral silica gel (60 Å, 230–400 mesh, Silicycle). Analytical thin layer chromatography was carried out using aluminum-backed silica gel 60 F254 plates (EMD), and compounds were visualized using UV light and aqueous basic $KMnO_4$ stain.

Instrumentation

^{1}H and ^{13}C NMR and 2D NMR spectra were recorded using a Varian Mercury 400 MHz, Bruker Avance III 400 MHz, Agilent DD2 600 (600 MHz), or Agilent DD2-500 (500 MHz) spectrometer equipped with a XSens cryoprobe. ^{1}H NMR are reported in parts per million (ppm) relative to tetramethylsilane and referenced to residual protium in the solvent. Spectral features are tabulated in the following order: chemical shift (δ, ppm); multiplicity (s-singlet, d-doublet, t-triplet, q-quartet, m-complex multiplet); coupling constant(s) (J, Hz); number of protons; assignment. Assignments were made on the basis of coupling constants and 2D NMR spectra. High-resolution mass spectra (HRMS) were obtained on a JEOL AccuTOF JMS- T1000LC mass spectrometer equipped with a DART (direct analysis in real time) ion source. Infrared (IR) spectra were obtained on a Perkin-Elmer Spectrum 100 instrument equipped with a single-bounce diamond/ZnSe ATR accessory as neat samples, or as thin film from CH_2Cl_2 as indicated. Spectral features are tabulated as follows: wavenumber (cm^{-1}); intensity (br-broad, s-strong, m-medium, w-weak). Specific rotations were measured with a Rudolph Autopol IV digital polarimeter equipped with a sodium lamp source (589 nm) and concentration (c) is reported in g/100 mL. Radical rearrangement reactions were all run in ½ dram or 1 dram vials and placed approximately 5 inches from a Kessil® LED lamp (either: A160WE Tuna Blue (40 W, at the highest intensity and colour settings) or H150-Blue (32 W).

Preparation of Substrates

General Procedure A

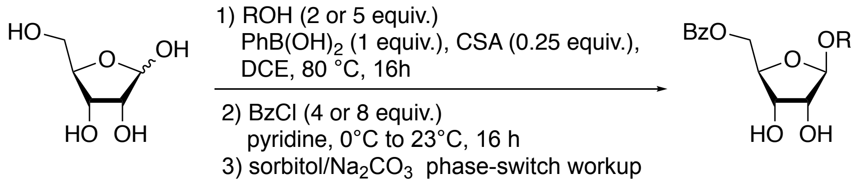

Synthesized according to adapted literature procedures.[1,2] To an oven-dried round-bottom flask containing a magnetic stir bar was added D-(-)-ribose (750.7 mg, 5.0 mmol), phenylboronic acid (609.7 mg, 5.0 mmol, 1.0 equiv) and (1S)-(+)-10-camphorsulfonic acid (290.4 mg, 1.25 mmol, 0.25 equiv). A rubber septum was used to seal the flask, which was then evacuated and backfilled with argon three times on a Schlenk line. Under a balloon of argon, dry dichloroethane (25 mL) and alcohol (10.0 or 25.0 mmol, 2.0 or 5.0 equiv) were added. The balloon and septum were removed and quickly replaced with a glass stopper. The flask was sealed using Teflon tape and parafilm and the mixture was stirred at 80 °C. After 16 hours, the crude mixture was concentrated under reduced pressure and residual water was removed by co-evaporation with toluene. The resulting crude was dissolved in pyridine (10 mL), cooled to 0 °C and benzoyl chloride (2.32 or 4.64 mL, 20.0 or 40.0 mmol, 4.0 or 8.0 equiv) was added slowly. The mixture was warmed to room temperature and stirred vigorously. After 16 hours, excess benzoyl chloride was quenched with methanol, the solvent was removed under reduced pressure and residual pyridine was removed by co-evaporation with toluene. The crude was suspended in ethyl acetate (100 mL) and a 1M sorbitol:1M sodium carbonate solution (100 mL) and transferred into a separatory funnel where it was shaken vigorously for 5 minutes. The aqueous layer was back-extracted with ethyl acetate (2 x 100 mL) and the combined organic layers were dried over magnesium sulfate. The solvent was removed by rotary evaporation and the crude material was purified by flash chromatography on silica gel.

General Procedure B

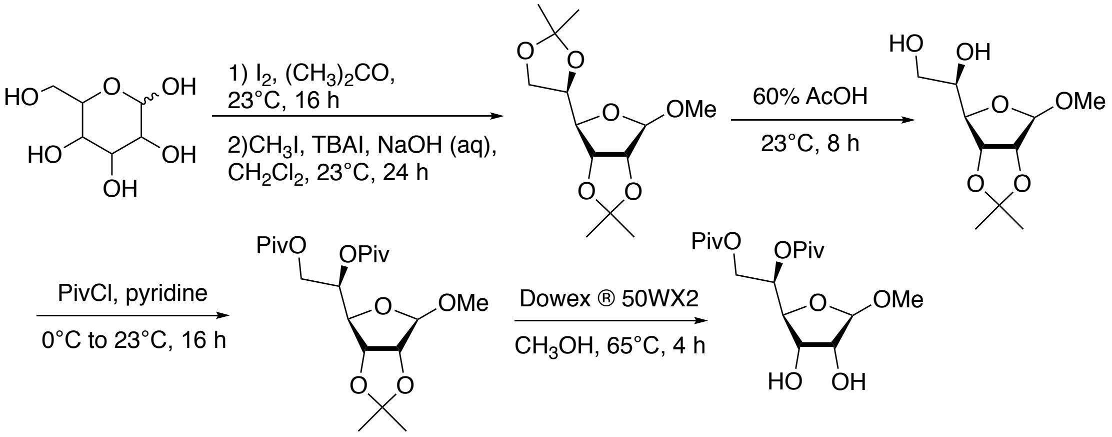

Synthesis was modified from previous literature procedures.[3, 4]
To an oven-dried, three-necked flask containing a magnetic stir bar was added sugar (900.8 mg, 5.0 mmol). Rubber septums were used to seal the flask, which was then evacuated and backfilled with argon 3 times on a Schlenk line. Acetone (50 mL, 0.1M) and iodine (253.8 mg, 1.0 mmol, 0.2 equiv.) were added and the solution was stirred vigorously. After 16 hours, the reaction was cooled to 0 °C and sequentially quenched with saturated sodium thiosulfate (12.5 mL) and sodium bicarbonate (12.5 mL) solutions. The mixture was extracted with chloroform (40 mL), and the organic layer was subsequently washed with saturated sodium bicarbonate solution (3 x 10 mL) and then dried over magnesium sulfate. The solvent was removed under reduced pressure and the product was transferred to an oven-dried round-bottom flask containing a magnetic stir bar. To the flask was added tetrabutylammonium iodide (0.1 equiv). A rubber septum was used to seal the flask, which was then evacuated and backfilled with argon three times on a Schlenk line. Under a balloon of argon, dichloromethane (1.5 mL per mmol substrate), 10M NaOH solution (0.25 mL per mmol substrate) and iodomethane (5 equiv.) were added. The solution was stirred vigorously at 30 ºC for 24 hours (complete consumption of substrate observed by TLC) and was then transferred to a separatory funnel. The organic layer was separated and the aqueous layer was back-extracted with dichloromethane (2 x 0.5 mL per mmol substrate). The combined organic layers were washed with saturated sodium bicarbonate, dried over magnesium sulfate and the solvent was removed under reduced pressure. The crude material was then purified by flash chromatography (10% to 20% ethyl acetate in hexanes).
To an oven-dried round-bottom flask containing a magnetic stir bar was added the methyl 2,3:5,6-di-O-isopropylidene furanoside and 60% acetic acid solution (3.0 mL per mmol substrate) and the reaction was stirred vigorously for 8 hours (complete consumption of substrate observed by TLC). The solvent was removed under reduced pressure. Residual acetic acid was removed through co-evaporation with toluene. The resulting crude material was purified by flash chromatography (75% to 100% ethyl acetate in hexanes).
To an oven-dried round-bottom flask containing a magnetic stir was added the methyl 2,3-O-isopropylidene furanoside. The flask was sealed with a rubber septum and was then evacuated and backfilled with argon 3 times on a Schlenk line. Under a balloon of argon, pyridine was added (5.0 mL per mmol substrate). The solution was cooled to 0 °C and be pivaloyl chloride (4.0 equiv) was added slowly. The solution as warmed to room temperature and stirred vigorously for 16 hours.

Excess pivaloyl chloride was quenched using methanol and the solvent was removed under reduced pressure. Residual pyridine was removed through co-evaporation with toluene. The crude material was dissolved in dichloromethane, washed with water and then with brine. The organic layer was dried over magnesium sulfate and then the solvent was removed under reduced pressure. The crude material was then purified by flash chromatography on silica gel (5% to 15% ethyl acetate in hexanes).

To an oven-dried round-bottom flask containing a stir bar was added the methyl 2,3-O-isopropylidene-5,6-di-O-pivaloyl furanoside and acid resin (Dowex® 50WX2 Hydrogen Form, 10.0 g per mmol substrate). The flask was sealed with a rubber septum and then evacuated and backfilled with argon 3 times on a Schlenk line. Under a balloon of argon, methanol (15.0 mL per mmol substrate) was added and the septum was quickly replaced with a glass stopper. The stopper was secured onto the flask using Teflon tape and parafilm and the heterogenous mixture was refluxed for 4 hours. The resin was then filtered off, the solvent removed under reduced pressure and the crude was purified by flash chromatography on silica gel.

1a –Methyl 5-O-benzoyl β-D-ribofuranoside

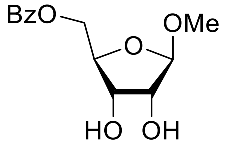

Prepared according to previous literature from methyl β-D-ribofuranoside and benzoyl chloride.[5]

^{1}H NMR (400 MHz, $CDCl_3$): δ (ppm) = 8.10–8.04 (m, 2H), 7.60–7.53 (m, 1H), 7.48–7.40 (m, 2H), 4.88 (s, 1H), 4.58 (dd, *J* = 11.8, 3.9 Hz, 1H), 4.48–4.38 (m, 2H), 4.30–4.22 (m, 1H), 4.09 (dd, *J* = 4.2 Hz, 1H), 3.33 (s, 3H), 2.62 (d, *J* = 3.6 Hz, 1H), 2.55 (d, *J* = 7.2 Hz, 1H).

(2-D)-1a –Methyl 5-O-benzoyl β-D-[2- ^{2}H$_1$] ribofuranoside

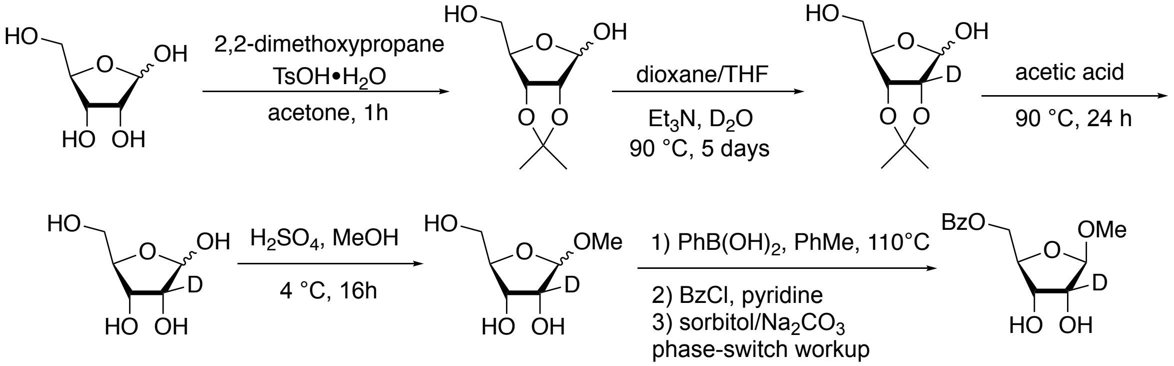

Prepared according to previous literature procedures.[6, 7, 5]

^{1}H NMR (500 MHz, $CDCl_3$): δ (ppm) = 8.11–8.03 (m, 2H), 7.61–7.53 (m, 1H), 7.49–7.40 (m, 2H), 4.88 (s, 1H), 4.58 (dd, *J* = 11.9, 3.8 Hz, 1H), 4.47–4.38 (m, 2H), 4.25 (ddd, *J* = 6.9, 5.0, 3.8 Hz, 1H), 3.33 (s, 3H), 2.71 (s, 2H).

^{13}C NMR (126 MHz, $CDCl_3$): δ (ppm) = 166.8, 133.3, 130.0, 129.9, 128.6, 108.1, 81.2, 74.8 (d, $J_{C\text{-}D}$ = 24.2), 72.1, 65.2, 55.3.

HRMS (DART$^+$, m/z): calculated for $C_{13}H_{19}{}^{2}H\ NO_6$ $[M+NH4]^+$: 287.13534, found: 287.13568.

$[\alpha]_D^{20}$ = −27.5 (c= 0.160 g/100 mL, $CHCl_3$)

1b –Octyl 5-O-benzoyl β-D-ribofuranoside

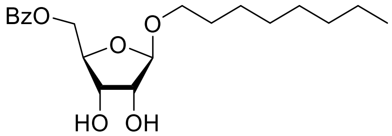

Prepared according to **General Procedure A** from n-octanol (5 equiv.) and benzoyl chloride (8 equiv.) The product was isolated as a white solid after flash chromatography on silica gel (10% to 50% ethyl acetate in hexanes).

^{1}H NMR (500 MHz, $CDCl_3$): δ (ppm) = 8.10–8.02 (m, 2H), 7.60–7.52 (m, 1H), 7.47–7.39 (m, 2H), 4.97 (s, 1H), 4.58 (dd, *J* = 11.9, 3.9 Hz, 1H), 4.43–4.37 (m, 2H), 4.24 (ddd, *J* = 6.9, 5.3, 3.8 Hz, 1H), 4.08 (d, *J* = 4.7 Hz, 1H), 3.67 (ddd, *J* = 9.5, 7.1, 6.5 Hz, 1H), 3.33 (ddd, *J* = 9.5, 6.9, 6.9 Hz, 1H), 2.88 (d, *J* = 14.3 Hz, 2H), 1.46 (ddd, *J* = 13.7, 9.7, 6.2 Hz, 2H), 1.29–1.21 (m, 10H), 0.86 (dd, *J* = 7.0, 7.0 Hz, 3H).

^{13}C NMR (126 MHz, $CDCl_3$): δ (ppm) = 166.8, 133.3, 129.9, 129.9, 128.5, 107.3, 81.0, 75.4, 72.3, 68.4, 65.5, 32.0, 29.6, 29.5, 29.3, 26.2, 22.8, 14.2.

HRMS (DART^{+}, m/z): calculated for $C_{20}H_{34}NO_6$ [M+NH4]$^{+}$: 384.23861, found 384.23916.

$[\alpha]_D^{20}$ = −7.6 (c= 0.485 g/100 mL, $CHCl_3$)

1c –Cyclohexyl 5-O-benzoyl β-D-ribofuranoside

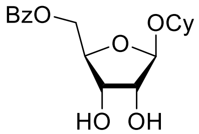

Prepared according to **General Procedure A** from cyclohexanol (5 equiv.) and benzoyl chloride (8 equiv.). The product was isolated as an oily white solid after flash chromatography on silica gel (20% to 40% ethyl acetate in hexanes).

^{1}H NMR (400 MHz, $CDCl_3$): δ (ppm) = 8.09–8.04 (m, 2H), 7.58–7.51 (m, 1H), 7.45–7.38 (m, 2H), 5.13 (s, 1H), 4.56 (dd, *J* = 11.7, 4.0 Hz, 1H), 4.44–4.33 (m, 2H), 4.25–4.18 (m, 1H), 4.05 (d, *J* = 4.7 Hz, 1H), 3.61–3.51 (m, 1H), 3.22 (s, 2H), 1.91–1.72 (m, 2H), 1.72–1.56 (m, 2H), 1.52–1.42 (m, 1H), 1.32–1.07 (m, 5H).

^{13}C NMR (101 MHz, $CDCl_3$): δ (ppm) = 166.9, 133.3, 129.9, 129.9, 128.5, 105.2, 80.7, 75.7, 75.7, 72.5, 65.9, 33.6, 31.5, 25.7, 24.2, 24.1.

HRMS (DART^{+}, m/z): calculated for $C_{18}H_{28}NO_6$ [M+NH4]$^{+}$: 354.19166, found 354.19210.

$[\alpha]_D^{20}$ = −28.9 (c=1.015 g/100 mL, $CHCl_3$)

1d –Dihydrocholesteroyl 5-O-benzoyl β-D-ribofuranoside

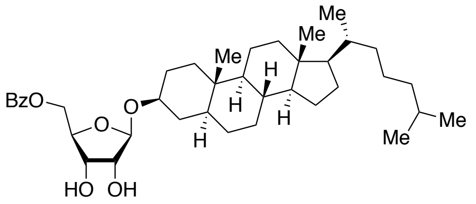

Prepared according to **General Procedure A** from dihydrocholesterol (2 equiv.) and benzoyl chloride (4 equiv.). The product was isolated as a white solid after flash chromatography on silica gel (10% to 40% ethyl acetate in hexanes).
^{1}H NMR (400 MHz, $CDCl_3$): δ (ppm) = 8.09–8.04 (m, 2H), 7.58–7.52 (m, 1H), 7.45–7.39 (m, 2H), 5.13 (s, 1H), 4.56 (dd, J = 11.7, 4.0 Hz, 1H), 4.44–4.33 (m, 2H), 4.25–4.17 (m, 1H), 4.03 (d, J = 4.7 Hz, 1H), 3.59–3.46 (m, 1H), 3.25 (d, 2H), 1.97–0.63 (m, 46H)
^{13}C NMR (101 MHz, $CDCl_3$): δ (ppm) = 166.9, 133.3, 130.0, 130.0, 128.5, 105.3, 80.8, 75.7, 72.4, 65.8, 56.6, 56.5, 54.4, 44.7, 42.7, 40.2, 39.6, 37.1, 36.3, 35.9, 35.7, 35.6, 34.3, 32.2, 29.5, 29.0, 28.4, 28.1, 24.3, 24.0, 22.9, 22.7, 21.3, 18.8, 12.3, 12.2.

HRMS ($DART^{+}$, m/z): calculated for $C_{39}H_{64}NO_6$ $[M+NH4]^{+}$: 642.4728, found 642.4726.

$[\alpha]_D^{20}$ = −14.5 (c=1.015 g/100 mL, $CHCl_3$)

1e –Methyl 2,3-O-isopropylidene-5-O-(5’-O-benzoyl β-D-ribofuranoside)- β-D-ribofuranoside

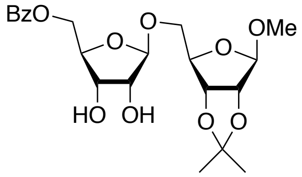

The glycosylation and subsequent deprotection were adapted from previous literature.[8, 9]
To an oven-dried round-bottom flask containing a magnetic stir bar was added methyl 2,3-O-isopropylidene-5-O-(β-D-ribofuranoside)-β-D-ribofuranoside (2.0 mmol, 1.0 equiv) and phenylboronic acid (2.0 mmol, 1.0 equiv). The flask was sealed with a rubber septum and then evacuated and backfilled with argon three times on a Schlenk line. Toluene (10 mL) was added under a balloon of argon and the rubber septum was replaced with a glass stopper, which was secured onto the flask using Teflon tape and parafilm. The solution was stirred at 110 °C for 16 hours. The solvent was removed under reduced pressure and the residue was resuspended in pyridine (10 mL). The solution was cooled to 0 °C, benzoyl chloride (4.0 mmol, 2.0 equiv) was added dropwise and the solution was stirred vigorously at room temperature. After 16 hours, excess benzoyl chloride was quenched by addition of methanol and the solvent was removed by rotary evaporation. Residual pyridine was removed by co-evaporation with toluene. The crude material was dissolved in ethyl acetate (60 mL) and transferred to a separatory funnel along with a 1M sorbitol:1M sodium carbonate solution (60 mL). The separatory funnel was hand shaken vigorously for 5 minutes. The organic layer was isolated, the aqueous layer was back-extracted with ethyl acetate (60 mL x 2) and the combined organic layers were dried over magnesium sulfate.

The solvent was removed under reduced pressure and the crude material was purified by flash chromatography (60% to 80% ethyl acetate in hexanes). The product was isolated as a white solid.
^{1}H NMR (400 MHz, $CDCl_3$): δ (ppm) = 8.08–8.02 (m, 2H), 7.59–7.52 (m, 1H), 7.47–7.40 (m, 2H), 5.00 (s, 1H), 4.92 (s, 1H), 4.60–4.47 (m, 3H), 4.45 – 4.34 (m, 2H), 4.29–4.20 (m, 2H), 4.16 –4.10 (m, 1H), 3.67 (dd, *J* = 10.2, 7.7 Hz, 1H), 3.44 (dd, *J* = 10.2, 7.0 Hz, 1H), 3.27 (s, 3H), 3.25 –3.19 (m, 1H), 3.17–3.04 (m, 1H), 1.43 (s, 3H), 1.26 (s, 3H).
^{13}C NMR (101 MHz, $CDCl_3$): δ (ppm) = 166.8, 133.4, 129.9, 129.9, 128.6, 112.6, 109.3, 107.6, 85.2, 85.1, 81.9, 81.2, 75.2, 72.0, 69.2, 65.4, 55.0, 26.5, 25.1.

HRMS (DART^{+}, m/z): calculated for $C_{21}H_{32}NO_{10}$ $[M+NH4]^{+}$: 458.20396, found: 458.20349.

$[\alpha]_D^{20}$ = −36.0 (c=1.010 g/100 mL, $CHCl_3$)

1f –Cyclohexyl 5-O-pivaloyl α-D-lyxofuranoside

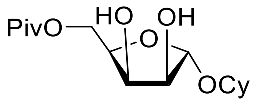

Prepared according to **General Procedure A** from D-(-)-lyxose instead of D-(-)-ribose, cyclohexanol (5 equiv.) and pivaloyl chloride (8 equiv.). The product was isolated as a white solid after flash chromatography on silica gel (10% to 50% ethyl acetate in hexanes).

^{1}H NMR (500 MHz, $CDCl_3$): δ (ppm) = 5.10 (d, *J* = 2.2 Hz, 1H), 4.51 (dd, *J* = 11.4, 5.6 Hz, 1H), 4.28 (dd, *J* = 4.9 Hz, 1H), 4.22–4.18 (m, 1H), 4.14 (dd, *J* = 11.4, 5.3 Hz, 1H), 4.12–4.08 (m, 1H), 3.59–3.48 (m, 1H), 3.24 (s, 1H), 2.90 (s, 1H), 1.93–1.83 (m, 2H), 1.75–1.67 (m, 2H), 1.55– 1.47 (m, 1H), 1.37–1.22 (m, 5H), 1.21 (s, 9H).

^{13}C NMR (126 MHz, $CDCl_3$): δ (ppm) = 179.2, 105.7, 77.5, 77.0, 76.1, 71.0, 62.4, 39.0, 33.8, 31.9, 27.3, 25.8, 24.3, 24.2.

HRMS (DART^{+}, m/z): calculated for $C_{16}H_{32}NO_6$ $[M+NH4]^{+}$: 334.22296, found 334.22260.

$[\alpha]_D^{20}$ =+48.0 (c= 0.575 g/100 mL, $CHCl_3$)

1g –Cyclohexyl 5-O-pivaloyl α-L-rhamnofuranoside

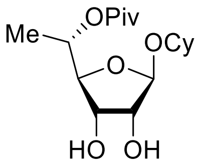

Prepared according to **General Procedure A** from L-(+)-rhamnose monohydrate instead of D-(-)-ribose, cyclohexanol (5 equiv.) and pivaloyl chloride (8 equiv.) The product was isolated as a colourless oil after flash chromatography on silica gel (10% to 50% ethyl acetate in hexanes).

^{1}H NMR (500 MHz, $CDCl_3$): δ (ppm) = 5.13–5.00 (m, 2H), 4.16–4.06 (m, 1H), 4.00–3.95 (m, 1H), 3.94 (d, *J* = 3.4 Hz, 1H), 3.80 (dd, *J* = 8.6, 2.6 Hz, 1H), 3.58–3.48 (m, 1H), 3.05–2.95 (m, 1H), 1.93–1.85 (m, 2H), 1.78–1.67 (m, 2H), 1.56–1.47 (m, 1H), 1.38–1.35 (m, 3H), 1.28 (d, *J* = 64.6 Hz, 5H), 1.20 (s, 9H).

^{13}C NMR (126 MHz, $CDCl_3$): δ (ppm) = 179.9, 107.1, 81.8, 78.4, 76.8, 70.3, 68.4, 39.1, 33.8, 32.2, 27.2, 25.8, 24.3, 24.2, 17.4.

HRMS (DART^{+}, m/z): calculated for $C_{17}H_{34}NO_6$ [M+NH4]$^{+}$: 348.23861, found 348.23842.

$[\alpha]_D^{20}$ = −50.9 (c= 0.980 g/100 mL, $CHCl_3$)

1h –Methyl 5,6-di-O-pivaloyl α-D-mannofuranoside

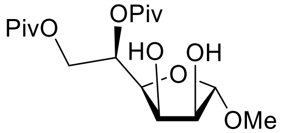

Prepared according to **General Procedure B** from D-(+)-mannose. The product was isolated as a white solid after flash chromatography on silica gel (20% to 40% ethyl acetate in hexanes).
^{1}H NMR (400 MHz, $CDCl_3$): δ (ppm) = 5.21 (ddd, *J* = 8.4, 6.0, 2.2 Hz, 1H), 4.85 (d, *J* = 2.9 Hz, 1H), 4.51 (dd, *J* = 12.2, 2.2 Hz, 1H), 4.27 (dd, *J* = 12.2, 6.0 Hz, 1H), 4.13 – 4.08 (m, 1H), 4.05 – 3.97 (m, 2H), 3.86 (d, *J* = 3.5 Hz, 1H), 3.36 (s, 3H), 3.22 (d, *J* = 8.2 Hz, 1H), 1.19 (s, 9H), 1.19 (s, 9H).
^{13}C NMR (101 MHz, $CDCl_3$): δ (ppm) = 179.4, 178.2, 109.5, 78.1, 77.6, 70.5, 69.7, 63.3, 55.9, 39.1, 39.0, 27.3, 27.2.

HRMS (DART^{+}, m/z): calculated for $C_{17}H_{34}NO_8$ [M+NH4]$^{+}$: 380.22844, found 380.22921.

$[\alpha]_D^{20}$ = +47.1 (c=1.015 g/100 mL, $CHCl_3$)

1i –Methyl 5,6-di-O-pivaloyl β-D-allofuranoside

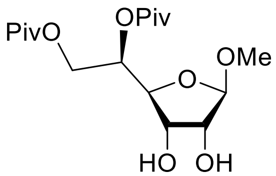

Prepared according to **General Procedure B** from D-allose. The product was isolated as a white solid after flash chromatography on silica gel (20% to 40% ethyl acetate in hexanes).
^{1}H NMR (400 MHz, $CDCl_3$): δ (ppm) = 5.16 (td, *J* = 6.6, 2.9 Hz, 1H), 4.82 (s, 1H), 4.48 (dd, *J* = 12.1, 2.9 Hz, 1H), 4.33 (m, 1H), 4.13 (dd, *J* = 12.1, 6.7 Hz, 1H), 4.06 – 3.97 (m, 2H), 3.34 (s, 3H), 1.21 (s, 9H), 1.18 (s, 9H).
^{13}C NMR (101 MHz, $CDCl_3$): δ (ppm) = 178.5, 178.0, 108.6, 81.5, 75.3, 72.7, 72.2, 63.1, 55.7, 39.1, 39.0, 27.3, 27.2.

HRMS (DART$^+$, m/z): calculated for $C_{17}H_{34}NO_8$ $[M+NH4]^+$: 380.22844, found: 380.22893.

$[\alpha]_D^{20} = -14.4$ (c=0.515 g/100 mL, $CHCl_3$)

1m –5'-O-pivaloyl-3-benzyluridine

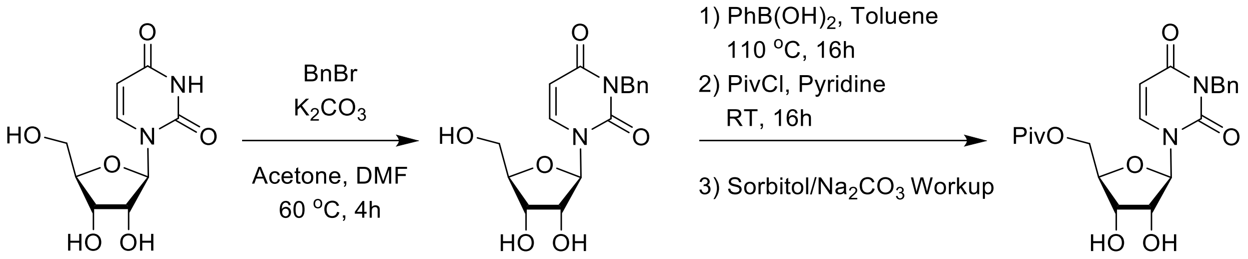

To a flame-dried round-bottom flask containing a magnetic stir bar was added uridine (1.0 equiv.) and K_2CO_3 (1.7 equiv.). The flask was sealed with a rubber septum and evacuated and backfilled with argon three times on a Schlenk line. Under a balloon of argon, acetone (1.0 mL/mmol substrate) and DMF (1.0 mL/mmol substrate) were added. Benzyl bromide (1.5 equiv.) was then added dropwise. The septum was replaced with a glass stopper and sealed using Teflon tape and parafilm. The solution was heated to 60 °C and stirred for 4 hours. The solution was then cooled to room temperature and allowed to stir overnight. The K_2CO_3 was removed by filtration and the reaction mixture concentrated under reduced pressure. The crude material was purified by flash chromatography on silica gel (5% to 15% methanol in dichloromethane), giving 3-(phenylmethyl)-ß-D-ribofuranosyluracil as a white solid.

To a flame-dried round-bottom flask was added 3-(phenylmethyl)-ß-D-ribofuranosyluracil (1.0 equiv.) and phenylboronic acid (1.0 equiv.). The flask was sealed with a rubber septum and evacuated and backfilled with argon three times on a Schlenk line. Under a balloon of argon, dry toluene (5.0 mL per mmol substrate) was added. The septum was quickly replaced with a glass stopper and sealed with Teflon tape and parafilm. The solution was stirred overnight at 110 °C. The reaction mixture was concentrated under reduced pressure. The resulting boronic ester intermediate was then dried through azeotropic removal of water with toluene. The flask was sealed with a septum and evacuated and backfilled with argon three times on a Schlenk line. To the flask was added pyridine (5.0 mL per mmol substrate) and the solution was cooled to 0 °C using an ice bath. Pivaloyl chloride (2.0 equiv.) was added dropwise, the flask was removed from the ice-bath and the solution was stirred overnight at room temperature. Excess pivaloyl chloride was quenched using methanol, the reaction mixture concentrated under reduced pressure and residual pyridine was removed by co-evaporation with toluene. The crude material was resuspended in ethyl acetate (20.0 mL/mmol substrate) and a 1M sorbitol:1M Na_2CO_3 solution (20.0 mL/mmol substrate). The solution was transferred to a separatory funnel and shaken vigorously for 5 minutes. The organic layer was set aside, and the aqueous layer was extracted two more times with ethyl acetate. The organic fractions were combined and dried over magnesium sulfate. The solvent was removed under reduced pressure and the crude material was purified by flash chromatography (10 to 25% acetone in dichloromethane), giving the titled compound as a waxy white solid.

^{1}H NMR (400 MHz, DMSO-d_6): δ (ppm) = 7.69 (d, J = 8.1 Hz, 1H), 7.34 – 7.20 (m, 5H), 5.83 (d, J = 8.1 Hz, 1H), 5.80 (d, J = 4.4 Hz, 1H), 5.53 (d, J = 5.4 Hz, 1H), 5.29 (d, J = 5.7 Hz, 1H), 5.04 – 4.92 (m, 2H), 4.29 – 4.18 (m, 2H), 4.14 – 4.07 (m, 1H), 4.07 – 4.00 (m, 1H), 4.00 – 3.93 (m, 1H), 1.16 (s, 9H).

^{13}C NMR (101 MHz, DMSO-d_6): δ (ppm) = 177.3, 161.8, 150.7, 139.4, 137.0, 128.3, 127.6, 127.2, 101.1, 90.0, 81.1, 73.0, 69.5, 63.7, 43.5, 38.3, 26.9.

HRMS (DART^{+}, m/z): calculated for $C_{21}H_{27}NO_7$ $[M+H]^{+}$: 419.1813 , found 419.1821.
$[\alpha]_D^{20}$ = +18.4 (c= 0.995 g/100 mL, $CHCl_3$)

Reaction Optimization

Optimization Procedure:

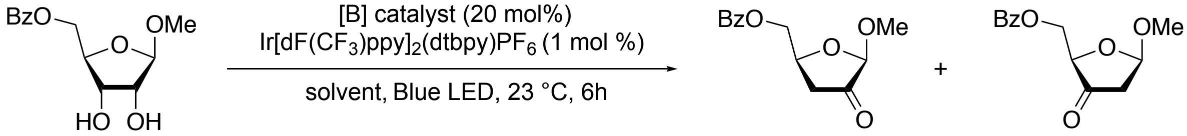

1a **2a** **2a'**

Methyl 5-*O*-benzoyl β-D-ribofuranoside (26.8 mg, 0.1 mmol, 1 equiv.), (Ir[dF(CF_3)ppy]$_2$(dtbpy))PF_6 (1 mg, 0.001 mmol, 1 mol%), quinuclidine (3.3 mg, 0.03 mmol, 30 mol%), organoboron catalyst (0.01 mmol, 20 mol%) and a small magnetic stir bar were combined in a ½ dram vial. A rubber septum was used to seal the vial, which was then evacuated and backfilled with argon three times on a Schlenk line. Dry, degassed dichloromethane was added under a balloon of argon. The balloon was removed, the rubber septum was quickly replaced with the vial cap and sealed with Teflon tape. The vial was placed 5 inches from a blue LED Kessil lamp and stirred at 1050 rpm (the temperature under the light was monitored and remained at ~25 °C throughout the course of the reaction). After 6 hours, the crude reaction mixture was concentrated under reduced pressure, and analyzed by ^{1}H NMR spectroscopy.

Table S1: Optimization of reaction conditions

Entry	Organoboron Catalyst	HAT Catalyst	Additive (20 mol%)	Solvent (concentration M)	NMR yield 2a[a]	Regioselectivity (2a: 2a')[b]
1	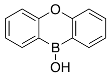	quinuclidine	-	CH_2Cl_2 (0.125M)	<5%	-
2[c]	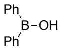	quinuclidine	-	CH_2Cl_2 (0.125M)	<5%	-
3	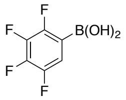	quinuclidine	-	CH_2Cl_2 (0.125M)	<5%	-
4	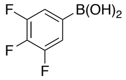	quinuclidine	-	CH_2Cl_2 (0.125M)	7%	4:1
5	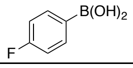	quinuclidine	-	CH_2Cl_2 (0.125M)	7%	8:1

**6**	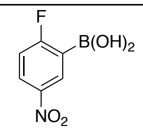	quinuclidine	-	CH_2Cl_2 (0.125M)	<5%	-
**7**	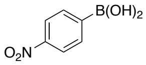	quinuclidine	-	CH_2Cl_2 (0.125M)	<5%	-
**8**	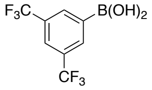	quinuclidine	-	CH_2Cl_2 (0.125M)	6%	6.6:1
**9**	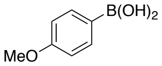	quinuclidine	-	CH_2Cl_2 (0.125M)	<5%	-
**10**	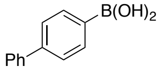	quinuclidine	-	CH_2Cl_2 (0.125M)	9%	3:1
**11**	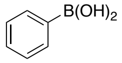	quinuclidine	-	CH_2Cl_2 (0.125M)	8%	14:1
**12**	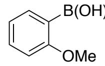	quinuclidine	-	CH_2Cl_2 (0.125M)	15%	6:1
**13**	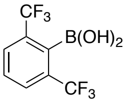	quinuclidine	-	CH_2Cl_2 (0.125M)	31%	10:1
**14**	$F_5C_6B(OH)_2$	quinuclidine	-	CH_2Cl_2 (0.125M)	67%	9:1
**15**	$F_5C_6B(OH)_2$	quinuclidine	*t*BuOH	CH_2Cl_2 (0.125M)	48%	9:1
**16**	$F_5C_6B(OH)_2$	quinuclidine	CF_3CH_2OH	CH_2Cl_2 (0.125M)	31%	9:1
**17**	$F_5C_6B(OH)_2$	quinuclidine	HFIP	CH_2Cl_2 (0.125M)	33%	9:1
**18**	$F_5C_6B(OH)_2$	quinuclidine	-	CH_2Cl_2 (0.17M)	45%	10:1
**19**	$F_5C_6B(OH)_2$	quinuclidine	-	CH_2Cl_2 (0.1M)	69%	10:1
**20**	$F_5C_6B(OH)_2$	quinuclidine	-	CH_2Cl_2 (0.0625M)	47%	9:1
**21**	$F_5C_6B(OH)_2$	Bu_4NOBz	-	CH_2Cl_2 (0.125M)	<5%	-
**22**	$F_5C_6B(OH)_2$	$MeO_2C(CH)_2SH$	-	CH_2Cl_2 (0.125)	<5%	-
**23**	$F_5C_6B(OH)_2$	3-quinuclidinol	-	CH_2Cl_2 (0.125)	<5%	-
**24**	$F_5C_6B(OH)_2$	quinuclidine	-	DME (0.125M)	6%	4.9:1
**25**	$F_5C_6B(OH)_2$	quinuclidine	-	DMF (0.125M)	14%	3:1
**26**	$F_5C_6B(OH)_2$	quinuclidine	-	CH_3CN (0.125M)	25%	12:1

[a]Yields of 2a was determined from ^1H NMR spectra of the crude reaction mixture (integration of ddd at 2.73 ppm relative to 1,3,5-trimethoxybenzene.[b]Ratios of regioisomers were determined by integration of the ^1H NMR spectrum of the crude reaction mixture in $CDCl_3$ 2a (ddd, 2.73 ppm, H-3a): 2a' (dd, 2.82 ppm, H-2a). [c]Reaction was run with 10 mol% of diphenyl borinic anhydride $(Ph_2B)_2O$.

Tabe S2: Control reactions

Entry	Changes to above reaction conditions	NMR yield 2a[a]	Regioselectivity (2a: S1)[b]
**1**	No Blue LED	<5%	N/A
**2**	Blue LED on for 30 min, no light for 5.5h	22%	11:1
**3**	30 min reaction time	18%	17: 1

**4**	No Ir cat.	<5%	N/A
**5**	No $F_5C_6B(OH)_2$	<5%	N/A
**6**	No quinuclidine	<5%	N/A
**7**[c]	No $F_5C_6B(OH)_2$, Benzoic acid (20 mol%)	<5%	N/A
**8**[c]	No $F_5C_6B(OH)_2$,, CSA (20 mol%)	5%	N/A

[a] Yields of 2a was determined from ^{1}H NMR spectra of the crude reaction mixture (integration of ddd at 2.73 ppm relative to 1,3,5-trimethoxybenzene. [b]Ratios of regioisomers were determined by integration of the ^{1}H NMR spectrum of the crude reaction mixture in $CDCl_3$ 2a (ddd, 2.73 ppm, H-3a): 2a' (dd, 2.82 ppm, H-2a). [c]Reaction was run at 0.25M instead of 0.125M.

Table S3: Effects on the ratio of boronic acid to quinuclidine on the yield of 2a

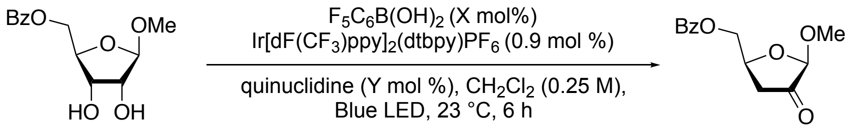

		$F_5C_6B(OH)_2$ (mol %)			
		5	10	20	30
	20	27%	31%	<5%	
**quinuclidine (mol %)**	30	12%	38%	**52%**	
	40	24%	40%	48%	
	50				48%

Isomerization of Furanosides to 2-Keto-3-deoxyfuranosides

General Procedure C

Furanoside derivative (0.2 mmol, 1 equiv.), (Ir[dF(CF_3)ppy]$_2$(dtbpy))PF_6 (2 mg, 0.002 mmol, 1 mol%), quinuclidine (6.7 mg, 0.06 mmol, 30 mol%), pentafluorophenyl boronic acid (8.5 mg, 0.04 mmol, 20 mol%) and a small magnetic stir bar were combined in a 1 dram vial. A small rubber septum was used to seal the vial, which was then evacuated and backfilled with argon three times on a Schlenk line. 1.6 mL of dry, degassed dichloromethane was added under a balloon of argon. The balloon was removed, the rubber septum was quickly replaced with the vial cap and sealed with Teflon tape. The vial was placed 5 inches from a blue LED Kessil lamp and stirred at 1050 rpm (the temperature under the light was monitored and remained at ~25 °C throughout the course of the reaction). After 6 hours, the crude reaction mixture was concentrated under reduced pressure, and analyzed by ^{1}H NMR spectroscopy. The crude material was then subjected to flash chromatography on silica gel.

General Procedure D

(Ir[dF(CF_3)ppy]$_2$(dtbpy))PF_6 (2 mg, 0.002 mmol, 1 mol%), quinuclidine (6.7 mg, 0.06 mmol, 30 mol%), pentafluorophenyl boronic acid (8.5 mg, 0.04 mmol, 20 mol%) and a small magnetic stir bar were combined in a 1 dram vial. A small rubber septum was used to seal the vial, which was then evacuated and backfilled with argon three times on a Schlenk line. A 0.125 M solution of the furanoside derivative was prepared under argon in dry, degassed dichloromethane. 1.6 mL (0.2 mmol uridine derivative) of the solution was added to the 1 dram under a balloon of argon. The balloon was removed, the rubber septum was quickly replaced with the vial cap and sealed with Teflon tape. The vial was placed 5 inches from a blue LED Kessil lamp and stirred at 1050 rpm (the temperature under the light was monitored and remained at ~25 °C throughout the course of the reaction). After 6 hours, the crude reaction mixture was concentrated under reduced pressure, and analyzed by ^{1}H NMR spectroscopy. The crude material was then subjected to flash chromatography on silica gel.

General Procedure E

Furanoside derivative (0.2 mmol, 1 equiv.), (Ir[dF(CF_3)ppy]$_2$(dtbpy))PF_6 (2 mg, 0.002 mmol, 1 mol%), quinuclidine (6.7 mg, 0.06 mmol, 30 mol%), pentafluorophenyl boronic acid (8.5 mg, 0.04 mmol, 20 mol%) and a small magnetic stir bar were combined in a 1 dram vial. A small rubber septum was used to seal the vial, which was then evacuated and backfilled with argon three times on a Schlenk line. 1.6 mL of dry, degassed dichloromethane was added under a balloon of argon. The balloon was removed, the rubber septum was quickly replaced with the vial cap and sealed with Teflon tape. The vial was placed 5 inches from a blue LED Kessil lamp and stirred at 1050 rpm (the temperature under the light was monitored and remained at ~25 °C throughout the course of the reaction). After 6 hours, the crude reaction mixture was concentrated under reduced pressure, and analyzed by ^{1}H NMR spectroscopy. The crude material was partially purified by flash chromatography on silica gel and then transferred to a scintillation vial. $NaBH_4$ (11.4 mg, 0.3 mmol, 1.5 equiv) and MeOH (3 mL) were added to the vial and the mixture was stirred at room

temperature for 1 hour. The crude reaction mixture was then concentrated and purified by flash chromatography on silica gel.

2a –Methyl 5-O-benzoyl-2-keto-3-β-D-deoxyribofuranoside

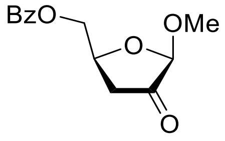

Prepared according to **General Procedure C** from methyl β-D-ribofuranoside **(1a)** (53.7 mg, 0.2 mmol). **2a** was obtained as an off-white solid (33.5 mg, 67%, >20:1 2a:2a') after flash chromatography on silica gel (10 to 30% ethyl acetate in hexanes) along with a mixed fraction containing **2a** and an aldol dimer of the product (light-yellow oil, 3.9 mg, 2.7:1 dimer: product) to give a total combined yield of 70% of **2a.** Analysis of the crude ^{1}H NMR spectrum revealed that the dimer by-product was present in 7%.

Regioselectivity: 9:1 (**2a** H-3a: **2a**' H-2a)

^{1}H NMR (500 MHz, $CDCl_3$): δ (ppm) = 8.10–8.03 (m, 2H, ArH), 7.62–7.54 (m, 1H, ArH), 7.49–7.41 (m, 2H, ArH), 4.90–4.81 (m, 1H, H-4), 4.72 (s, 1H, H-1), 4.54 (dd, J = 11.7, 4.8 Hz, 1H, H-5a), 4.47 (dd, J = 11.7, 5.9 Hz, 1H, H-5b), 3.45 (s, 3H, OCH_3), 2.73 (ddd, J = 18.8, 8.3, 0.6 Hz, 1H, H-3a), 2.61 (ddd, J = 18.8, 4.4, 0.8 Hz, 1H, H-3b).

^{13}C NMR (126 MHz, $CDCl_3$): δ (ppm) = 207.1, 166.3, 133.4, 129.9, 129.8, 128.6, 98.9, 74.2, 67.0, 55.9, 36.1.

IR (thin film, cm^{-1}): 3067 (w), 2938 (w), 2834 (w), 1773 (s), 1719 (s), 1602 (w), 1585 (w), 1452 (m), 1316 (m), 1269 (s), 1178 (m), 1117 (s), 1092 (s), 1071 (s), 1040 (s), 1026 (s), 712 (s).

HRMS (DART^{+}, m/z): calculated for $C_{13}H_{18}NO_5$ [M+NH4]$^{+}$: 268.11850, found 268.11786.

$[\alpha]_D^{20}$ = −17.6 (c= 0.085 g/100 mL, $CHCl_3$)

Select peaks for the minor regioisomer **(2a')**:

^{1}H NMR (500 MHz, $CDCl_3$): δ (ppm) = 5.37 (dd, J = 5.7, 1.0 Hz, 0.04H, H-1), 3.40 (s, 0.15H, OCH_3), 2.82 (dd, J = 18.3, 5.7 Hz, 0.07H, H-2a), 2.53 (ddd, J = 18.3, 1.2, 1.2 Hz, 0.07H, H-2b).

2b – Octyl 5-O-benzoyl-2-keto-3-β-D-deoxyribofuranoside

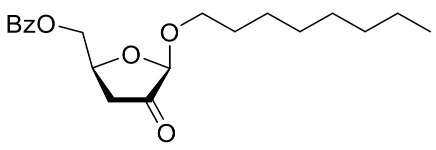

Octyl β-D-ribofuranoside **(1b)** (36.7 mg, 0.1 mmol)., (Ir[dF(CF_3)ppy]$_2$(dtbpy))PF_6 (1 mg, 0.001 mmol, 1 mol%), quinuclidine (3.3 mg, 0.03 mmol, 30 mol%), pentafluorophenyl boronic acid (4.2 mg, 0.02 mmol, 20 mol%) and a small magnetic stir bar were combined in a 0.5 dram vial. A small rubber septum was used to seal the vial, which was then evacuated and backfilled with argon three times on a Schlenk line. 0.8 mL of dry, degassed dichloromethane was added under a balloon of argon. The balloon was removed, the rubber septum was quickly replaced with the vial cap and sealed with Teflon tape. The vial was placed 5 inches from a blue LED Kessil lamp and stirred at 1050 rpm (the temperature under the light was monitored and remained at ~25 °C throughout the course of the reaction). After 6 hours, the crude reaction mixture was concentrated under reduced pressure, and analyzed by ^{1}H NMR spectroscopy. The crude material was then subjected to flash chromatography on silica gel. **2b** was obtained as a colourless oil (15.6 mg, 47%) after flash chromatography on silica gel (10 to 30% ethyl acetate in hexanes) along with a mixed fraction containing **2b** and a dimer by-product (8.7 mg, 4:1 (**2b**: dimer)) to give a total yield of 65% **2b.** A second fraction containing **2b** and **2b'** was additionally isolated in 8% combined yield (2.9 mg, 11:1 (**2b**': **2b**)). Analysis of the crude ^{1}H NMR spectrum revealed that the dimer by-product was present in 6%.

Regioselectivity: 10:1 (**2b** H-3a: **2b'** H-2a)

^{1}H NMR (400 MHz, $CDCl_3$): δ (ppm) = 8.09–8.02 (m, 2H, ArH), 7.60–7.54 (m, 1H, ArH), 7.47 –7.43 (m, 2H, ArH), 4.86–4.80 (m, 1H, H-4), 4.79 (s, 1H, H-1), 4.53 (dd, J = 11.7, 4.8 Hz, 1H, H-5a), 4.46 (dd, J = 11.6, 6.0 Hz, 1H, H-5b), 3.78–3.69 (m, 1H, -OCH_2-), 3.56–3.49 (m, 1H, -OCH_2-), 2.72 (dd, J = 18.7, 8.3 Hz, 1H, H-3a), 2.59 (ddd, J = 18.8, 4.4, 0.8 Hz, 1H, H-3b), 1.58–1.47 (m, 2H, CH_2), 1.30–1.21 (m, 10H, CH_2), 0.92–0.84 (m, 3H, CH_3).

^{13}C NMR (101 MHz, $CDCl_3$): δ (ppm) = 207.4, 166.3, 133.3, 129.9, 129.8, 128.5, 98.2, 74.0, 69.5, 67.1, 36.3, 31.9, 29.6, 29.4, 29.3, 26.1, 22.7, 14.2.

IR (neat, cm^{-1}): 2926 (m), 2856 (w), 1775 (m), 1721 (m), 1602 (w), 1452 (w), 1267 (s), 1176 (m), 1116 (s), 1097 (s), 1070 (s), 1026 (s), 710 (s).

HRMS (DART$^+$, m/z): calculated for $C_{20}H_{32}NO_5$ [M+NH4]$^+$: 366.22804, found 366.22790.

$[\alpha]_D^{20} = -17.6$ (c= 0.245 g/100 mL, $CHCl_3$)

Select peaks for the minor regioisomer (**2b**'):

^{1}H NMR (400 MHz, $CDCl_3$): δ (ppm) = 8.13–8.03 (m, 2H, ArH), 7.61–7.52 (m, 1H, ArH), 7.51–7.40 (m, 2H, ArH), 5.47 (dd, J = 5.8, 1.3 Hz, 1H, H-1), 4.64 (dd, J = 11.5, 3.2 Hz, 1H, H-5a), 4.48–4.36 (m, 2H, H-4 and H5b), 3.86 – 3.78 (m, 1H, -OCH_2-), 3.50–3.40 (m, 1H, -OCH_2-), 2.82 (dd, J = 18.3, 5.7 Hz, 1H, H-2a), 2.54 (ddd, J = 18.3, 1.3 Hz, 1H, H-2b), 1.54–1.44 (m, 2H, CH_2), 1.28–1.19 (m, 10H, CH_2), 0.89– 0.84 (m, 3H, CH_2).

2c – Cyclohexyl 5-O-benzoyl-2-keto-3-β-D-deoxyribofuranoside

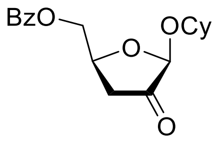

Prepared according to **General Procedure C** from cyclohexyl β-D-ribofuranoside **(1c)** (67.3 mg, 0.2 mmol). **2c** was obtained as a light-yellow oil (44.2 mg, 69%) after flash chromatography on silica gel (10 to 30% ethyl acetate in hexanes).

Regioselectivity: >20:1 (**2c** H-3a: **2c'** H-2a)

^{1}H NMR (400 MHz, $CDCl_3$): δ (ppm) = 8.10–8.00 (m, 2H, ArH), 7.61–7.51 (m, 1H, ArH), 7.48 –7.39 (m, 2H, ArH), 4.93 (s, 1H, H-1), 4.85–4.78 (m, 1H, H-4), 4.52–4.48 (m, 2H, H-5a and H-5b), 3.72–3.63 (m, 1H, -OCH-), 2.71 (dd, J = 18.8, 8.2 Hz, 1H, H-3a), 2.58 (dd, J = 18.8, 4.2 Hz, 1H, H-3b), 1.94–1.83 (m, 2H, CH_2), 1.75–1.63 (m, 2H, CH_2), 1.48 1.52–1.44 (m, 1H, CH_2), 1.39–1.11 (m, 5H, CH_2).

^{13}C NMR (101 MHz, $CDCl_3$): δ (ppm) = 207.9, 166.3, 133.3, 129.9, 129.8, 128.5, 96.5, 77.7, 77.4, 73.8, 67.2, 36.3, 33.4, 31.8, 25.6, 24.1, 24.0.

IR (neat, cm^{-1}): 2932 (m), 2858 (w), 1773 (s), 1719 (s), 1602 (w), 1451 (m), 1316 (w), 1267 (s), 1176 (m), 1116 (s), 1094 (s), 1069 (s), 1022 (s), 950 (m), 710 (s).

HRMS (DART$^+$, m/z): calculated for $C_{18}H_{26}NO_5$ [M+NH4]$^+$: 336.18109, found 336.18047.

$[\alpha]_D^{20}$ = −27.1 (c= 0.28 g/100 mL, $CHCl_3$)

2d – Dihydrocholesteroyl 5-O-benzoyl-2-keto-3-β-D-deoxyribofuranoside

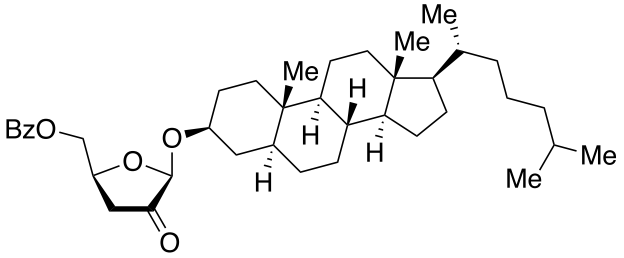

Prepared according to **General Procedure C** from dihydrocholesteroyl β-D-ribofuranoside **(1d)** (124.2 mg, 0.2 mmol). **2d** was obtained as a white solid (76.6 mg, 63%) after flash chromatography on silica gel (5 to 20% ethyl acetate in hexanes).

Regioselectivity: 17:1(**2d** H-3a: **2d'** H-2a)

^{1}H NMR (400 MHz, $CDCl_3$): δ (ppm) = 8.11–8.02 (m, 2H, ArH), 7.62–7.53 (m, 1H, ArH), 7.49–7.40 (m, 2H, ArH), 4.95 (s, 1H, H-1), 4.86–4.75 (m, 1H, H-4), 4.56–4.43 (m, 2H, H-5a, H-5b), 3.70–3.58 (m, 1H, $CH_{dihydrocholesterol}$), 2.70 (dd, J = 18.7, 8.2 Hz, 1H, H-3a), 2.59 (dd, J = 18.9, 4.1 Hz, 1H, H-3b), 2.10–0.50 (m, 46H, $CH_{dihydrocholesterol}$).

^{13}C NMR (101 MHz, $CDCl_3$): δ (ppm) = 208.0, 166.3, 133.4, 130.0, 129.8, 128.5, 96.4, 78.6, 73.8, 67.1, 56.6, 56.4, 54.4, 44.9, 42.7, 40.2, 39.6, 37.1, 36.3, 35.9, 35.7, 35.6, 34.4, 32.2, 29.4, 28.9, 28.4, 28.1, 24.3, 24.0, 23.0, 22.7, 21.3, 18.8, 12.3, 12.2.

IR (neat, cm^{-1}): 2930 (m), 2866 (m), 2849 (m), 1770 (m), 1723 (s), 1602 (w), 1584 (w), 1467 (m), 1450 (m), 1382 (m), 1271 (s), 1174 (m), 1116 (s), 1070 (s), 1026 (s), 951 (m), 708 (s).

HRMS (DART^{+}, m/z): calculated for $C_{39}H_{62}NO_5$ $[M+NH_4]^+$: 624.46280, found 624.46278.

$[\alpha]_D^{20} = -14.2$ (c=0.515 g/100 mL, $CHCl_3$)

2e – Methyl 2,3-O-isopropylidene-5-O-(5'-O-benzoyl 2-keto-3-deoxy-β-D-ribofuranoside)-β-D-ribofuranoside

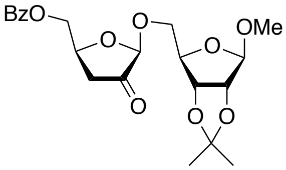

Prepared according to **General Procedure C** from methyl 2,3-O-isopropylidene-5-O-(5'-O-benzoyl β-D-ribofuranoside)-β-D-ribofuranoside (**1e**) (88.1 mg, 0.2 mmol). **2e** was obtained as a light-yellow solid (55.6 mg, 66%) after flash chromatography (15 to 35% ethyl acetate in hexanes).

Regioselectivity: 17:1 (**2e** H-1: **2e'** H-1)

^{1}H NMR (400 MHz, $CDCl_3$): δ (ppm) = 8.09–8.02 (m, 2H, ArH), 7.60–7.53 (m, 1H, ArH), 7.48–7.41 (m, 2H, ArH), 4.90 (s, 1H, H-1'), 4.89–4.83 (m, 2H, H1, H-4'), 4.59 (d, *J* = 5.9 Hz, 1H, H-2), 4.52–4.48 (m, 3H, H-3, H-5a',H-5b'), 4.35–4.26 (m, 1H, H-4), 3.73 (dd, *J* = 9.8, 8.3 Hz, 1H, H-5a), 3.60 (dd, *J* = 9.9, 6.5 Hz, 1H, H-5b), 3.26 (s, 3H, OCH_3), 2.73 (dd, *J* = 18.8, 8.4 Hz, 1H, H-3a'), 2.58 (dd, *J* = 18.8, 4.2 Hz, 1H, H-3b'), 1.41 (s, 3H, CH_3), 1.24 (s, 3H, CH_3).

^{13}C NMR (101 MHz, $CDCl_3$): δ (ppm) = 206.4, 166.3, 133.4, 129.9, 129.7, 128.6, 112.5, 109.4, 98.2, 85.2, 84.8, 82.0, 74.3, 69.9, 67.0, 55.0, 36.1, 26.5, 25.0.

IR (neat, cm^{-1}): 3008 (w), 2982 (w), 2930 (w), 1774 (m), 1715 (s), 1449 (w), 1440 (w), 1378 (m), 1271 (s), 1251 (m), 1192 (m), 1176 (m), 1159 (m), 1118 (s), 1093 (s), 1082 (s), 1035 (s), 1022 (s), 993 (s), 961 (s), 865 (s), 709 (s).

HRMS (DART$^+$, m/z): calculated for $C_{21}H_{30}NO_9$ $[M+NH_4]^+$: 440.19206, found 440.19260.

$[\alpha]_D^{20}$ = −32.3 (c=0.520 g/100 mL, $CHCl_3$)

2f – Cyclohexyl 5-O-pivaloyl 2-keto-3-deoxy-α-D-lyxofuranoside

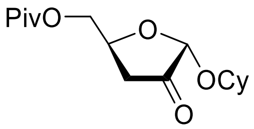

Prepared according to **General Procedure C** from cyclohexyl α-D-lyxofuranoside **(1f)** (63.3 mg, 0.2 mmol). **2f** was obtained as a light-yellow oil (33 mg, 55%) after flash chromatography on silica gel (5 to 25% ethyl acetate in hexanes) along with the minor C3 regioisomer (**2f'**) as a colourless oil (4.1 mg, 7%). Analysis of the crude ^{1}H NMR revealed 9% of a dimerization by-product.

Regioselectivity: 10:1 (**2f** H-1: **2f'** H-1)

^{1}H NMR (400 MHz, $CDCl_3$): δ (ppm) = 4.92 (d, *J* = 0.8 Hz, 1H, H-1), 4.74–4.66 (m, 1H, H-4), 4.35 (dd, *J* = 12.0, 3.5 Hz, 1H, H-5a), 4.21 (dd, *J* = 12.0, 4.7 Hz, 1H, H-5b), 3.69–3.60 (m, 1H, -OCH-), 2.63 (ddd, *J* = 18.4, 6.7, 0.8 Hz, 1H, H-3a), 2.27 (ddd, *J* = 18.4, 8.0, 0.9 Hz, 1H, H-3b), 1.95–1.83 (m, 2H, CH_2), 1.78–1.66 (m, 2H, CH_2), 1.55–1.46 (m, 1H, CH_2), 1.45–1.22 (m, 5H, CH_2), 1.19 (s, 9H, $C(O)CH_3$).

^{13}C NMR (101 MHz, $CDCl_3$): δ (ppm) = 207.5, 178.2, 97.0, 76.8, 73.1, 65.0, 36.3, 33.6, 31.9, 27.2, 27.2, 25.6, 24.2, 24.0.

IR (neat, cm^{-1}): 2933 (m), 2859 (w), 1776 (m), 1731 (s), 1481 (w), 1453 (w), 1282 (m), 1151 (s), 1084 (m), 1024 (s), 946 (w), 893 (w).

HRMS (DART$^+$, m/z): calculated for $C_{16}H_{30}NO_5$ $[M+NH4]^+$: 316.21239, found 316.21223.

$[\alpha]_D^{20}$ =+23.5 (c= 0.315 g/100 mL, $CHCl_3$)

Select peaks for the minor regioisomer (**2f'**)*:*

^{1}H NMR (400 MHz, $CDCl_3$): δ (ppm) = 5.62 (d, *J* = 5.3 Hz, 1H, H-1), 4.44 (dd, *J* = 12.0, 2.7 Hz, 1H, H-5a), 4.22 (dd, *J* = 12.0, 3.5 Hz, 1H, H-5b), 4.17 (dd, *J* = 3.1 Hz, 1H, H-4), 3.76–3.66 (m, 1H, -OCH-), 2.59 (ddd, *J* = 17.9, 5.4, 0.8 Hz, 1H, H-2a), 2.43 (d, *J* = 18.1 Hz, 1H, H-2b), 1.93–1.83 (m, 2H, CH_2), 1.75–1.68 (m, 2H, CH_2), 1.35–1.24 (m, 8H, CH_2), 1.17 (s, 9H, C(O)CH_3).

^{13}C NMR (126 MHz, $CDCl_3$): δ (ppm) = 211.7, 178.0, 98.4, 75.6, 75.4, 62.4, 44.3, 38.9, 33.6, 31.8, 27.3, 25.7, 24.3, 24.1.

IR (thin film, cm^{-1}): 2931 (s), 2858 (s), 1769 (s), 1736 (s), 1481 (w), 1453 (w), 1398 (w), 1283 (m), 1155 (s), 1069 (s), 961 (m), 934 (w), 802 (w).

HRMS (DART$^+$, m/z): calculated for $C_{16}H_{30}NO_5$ [M+NH4]$^+$: 316.21239, found: 316.21224.

$[\alpha]_D^{20}$ =+168.8 (c= 0.080 g/100 mL, $CHCl_3$)

2g – Cyclohexyl 2-keto-3-deoxy-α-L-rhamnofuranoside

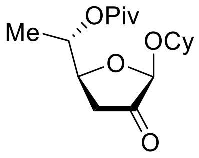

Prepared according to **General Procedure D** from cyclohexyl α- L-rhamnofuranoside **(1g)** (66 mg, 0.2 mmol). **2g** was obtained as a colourless oil along with a minor C3 regioisomer (**2g'**) (42.5 mg, 68%, 4.4:1 C2: C3) after flash chromatography on silica gel (5 to 20% ethyl acetate in hexanes). Analysis of the crude ^{1}H NMR revealed 10% of a dimerization by-product.

Regioselectivity: 4.4:1 (**2g** H-1: **2g'** H-1)

^{1}H NMR (400 MHz, $CDCl_3$): δ (ppm) = 5.19–5.08 (m, 1H, H-5), 4.87 (d, *J* = 0.8 Hz, 1H, H-1), 4.53–4.43 (m, 1H, H-4), 3.72–3.58 (m, 1H, -OCH-), 2.58 (dd, *J* = 18.6, 6.6 Hz, 1H, H-3a), 2.36 (ddd, *J* = 18.5, 7.8, 0.8 Hz, 1H, H-3b), 1.94–1.79 (m, 2H, CH_2), 1.77–1.64 (m, 2H, CH_2), 1.56–1.45 (m, 2H, CH_2), 1.43–1.18 (m, 12H, CH_2, CH_3), 1.16 (s, 9H, C(O)CH_3).

^{13}C NMR (101 MHz, $CDCl_3$): δ (ppm) = 207.7, 177.6, 96.9, 77.2, 70.3, 44.8, 39.0, 35.3, 33.6, 31.9, 27.2, 25.6, 24.2, 24.0, 16.2.

Select peaks for the minor regioisomer (**2g'**)*:*

^{1}H NMR (400 MHz, $CDCl_3$): δ (ppm) = 5.55 (d, *J* = 5.4 Hz, 0.24H), 3.97 (dd, *J* = 2.8, 0.9 Hz, 0.23H).

^{13}C NMR (101 MHz, $CDCl_3$): δ (ppm) = 211.4, 98.2, 77.8, 77.0, 69.6, 31.8, 27.2, 25.7, 14.8.

IR (neat, cm^{-1}): 2933 (m), 2860 (w), 1772 (m), 1729 (s), 1480 (m), 1453 (m), 1281 (m), 1155 (s), 1085 (s), 1026 (s), 923 (w), 769 (w), 733 (m).

HRMS (DART$^+$, m/z): calculated for $C_{17}H_{32}NO_5$ $[M+NH_4]^+$: 330.22805, found 330.22772.

2h – Methyl 5,6-di-O-pivaloyl 2-keto-3-deoxy-α-D-mannofuranoside

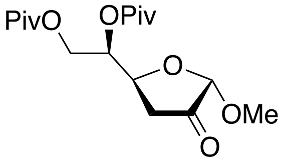

Prepared according to **General Procedure C** from methyl α-D-mannofuranoside **(1h)** (72.5 mg, 0.2 mmol). A mixture of regioisomers (6:1 **2h**:**2h'**) were obtained as a light-yellow oil (49.4 mg, 72%) after flash chromatography on silica gel (5 to 25% ethyl acetate in hexanes).

Regioselectivity: 5:1 (**2h** H-3a: **2h**' H-2a)

^{1}H NMR (400 MHz, $CDCl_3$): δ (ppm) = 5.33–5.27 (m, 1H, H-5), 4.64 (s, 1H, H-1), 4.60 (m, 1H, H-4), 4.40 (dd, *J* = 12.1, 3.4 Hz, 1H, H-6a), 4.13 (dd, *J* = 12.1, 5.5 Hz, 1H, H-6b), 3.42 (s, 3H, OCH_3), 2.59 (dd, *J* = 18.7, 6.5 Hz, 1H, H-3a), 2.43 (ddd, *J* = 18.6, 8.2, 0.6 Hz, 1H, H-3b), 1.18 (s, 9H, $C(CH_3)_3$), 1.16 (s, 9H, $C(CH_3)_3$).

^{13}C NMR (101 MHz, $CDCl_3$): δ (ppm) = 206.5, 178.0, 177.2, 98.9, 74.2, 71.6, 62.4, 55.7, 39.0, 38.9, 36.2, 27.2, 27.1.

Select signals from the minor regioisomer (**2h'**)*:*

^{1}H NMR (400 MHz, $CDCl_3$): δ (ppm) = 5.38–5.33 (m, 1H, H-5), 4.32 (dd, *J* = 11.8, 4.3 Hz, 1H, H-6a), 4.25 (dd, *J* = 11.8, 7.4 Hz, 1H, H-6b), 4.06–4.03 (m, 1H, H-4), 3.40 (s, 3H, OCH_3)

^{13}C NMR (101 MHz, $CDCl_3$): δ (ppm) = 210.0, 177.9, 177.2, 101.4, 75.1, 69.7, 61.8, 55.2, 43.8, 39.0, 38.9, 27.2, 27.1.

IR (neat, cm^{-1}): 2972 (m), 2936 (w), 2874 (w), 1777 (m), 1732 (s), 1490 (m), 1462 (m), 1397 (m), 1366 (m), 1278 (m), 1135 (s), 1090 (s), 1037 (s), 928 (w), 767 (w).

HRMS (DART$^+$, m/z): calculated for $C_{17}H_{32}NO_7$ $[M+NH_4]^+$: 362.21788, found 362.21768.

2i – Methyl 5,6-di-O-pivaloyl 2-keto-3-deoxy- β-D-allofuranoside

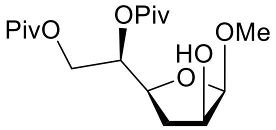

Prepared according to **General Procedure E** from methyl 5,6-di-O-pivaloyl β-D-allofuranoside (**1i**) (72.5 mg, 0.2 mmol). **2i** was obtained as a clear oil (45.7 mg, 66%) after flash chromatography on silica gel (20% to 35% ethyl acetate in hexanes).

Regioselectivity: >20:1

^{1}H NMR (400 MHz, CD_3CN): δ (ppm) = δ 4.97 (ddd, *J* = 7.1, 6.3, 2.6 Hz, 1H, H-5), 4.66 (d, *J* = 4.5 Hz, 1H, H-1), 4.45 (dd, *J* = 12.1, 2.6 Hz, 1H, H-6a), 4.18–4.06 (m, 2H, H-2, H-4), 4.03 (dd, *J* = 12.1, 6.2 Hz, 1H, H-6b), 3.37 (s, 3H, OCH_3), 2.24 (ddd, *J* = 11.8, 7.7, 6.7 Hz, 1H, H-3a), 1.64 (ddd, *J* = 11.8, 10.4, 9.2 Hz, 1H, H-3b), 1.17 (s, 9H, $C(CH_3)_3$), 1.15 (s, 9H, $C(CH_3)_3$).

^{13}C NMR (101 MHz, CD_3CN): δ (ppm) = 178.6, 178.1, 103.3, 75.9, 74.5, 72.9, 63.7, 55.6, 39.5, 39.4, 34.4, 27.4, 27.3.

IR (neat, cm^{-1}): 3459 (br), 2967 (m), 2935 (m), 1730 (s), 1480 (m), 1459 (w), 1397 (w), 1366 (w), 1282 (m), 1142 (s), 1054 (s), 1030 (s), 979 (m), 883 (w), 767 (w).

HRMS (DART$^+$, m/z): calculated for $C_{17}H_{34}NO_7$ $[M+NH_4]^+$: 364.23298, found 364.23275.

$[\alpha]_D^{20}$ = −34.4 (c=0.270 g/100 mL, $CHCl_3$)

2m –5’-O-pivaloyl-2-keto-3-deoxy-*N*^{3}-benzyluridine

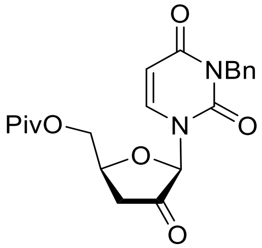

Prepared according to **General Procedure D** from 5’-O-pivaloyl-3-N-benzyluridine (**1m**) (83.7 mg, 0.2 mmol), but stirring for 17 hours instead. **2m** was obtained as a white solid (21.3 mg, 27%) after flash chromatography on silica gel (35% to 55% ethyl acetate in hexanes).

Regioselectivity: >20:1

^{1}H NMR (500 MHz, $CDCl_3$): δ (ppm) = 7.43–7.37 (m, 2H, ArH), 7.32–7.22 (m, 3H, ArH), 7.08 (d, *J* = 8.0 Hz, 1H, H-5), 5.79 (d, *J* = 8.0 Hz, 1H, H-6), 5.10–4.98 (m, 3H, H1’, NCH_2Ph), 4.64 –

4.54 (m, 1H, H-4’), 4.36 – 4.30 (m, 2H, H-5a’, H-5b’), 2.94 (dd, *J* = 18.5, 8.5 Hz, 1H, H-3a’), 2.64 (dd, *J* = 18.5, 7.5 Hz, 1H, H-3b’), 1.20 (s, 9H, $C(CH_3)_3$).

^{13}C NMR (126 MHz, $CDCl_3$): δ (ppm) = 205.2, 178.3, 162.3, 150.8, 141.6, 136.2, 129.0, 128.6, 127.9, 103.1, 88.0, 74.1, 65.3, 44.5, 39.0, 36.5, 27.3.

IR (neat, cm^{-1}): 2959 (w), 1772 (m), 1732 (m), 1713 (s), 1683 (s), 1651 (s), 1480 (w), 1450 (s), 1383 (m), 1357 (m), 1286 (m), 1264 (w), 1232 (m), 1169 (s), 1155 (s), 1089 (m), 1058 (s), 1035 (m), 935 (w), 911 (w), 803 (s), 763 (m), 706 (m), 694 (m).

HRMS (DART$^+$, m/z): calculated for $C_{21}H_{25}N_2O_6$ $[M+H]^+$: 401.17071, found 401.17075.

$[\alpha]_D^{20}$ = +16.9 (c=0.320 g/100 mL, $CHCl_3$)

Synthesis of Uridine Analogs from 2-Keto-3-deoxyfuranosides

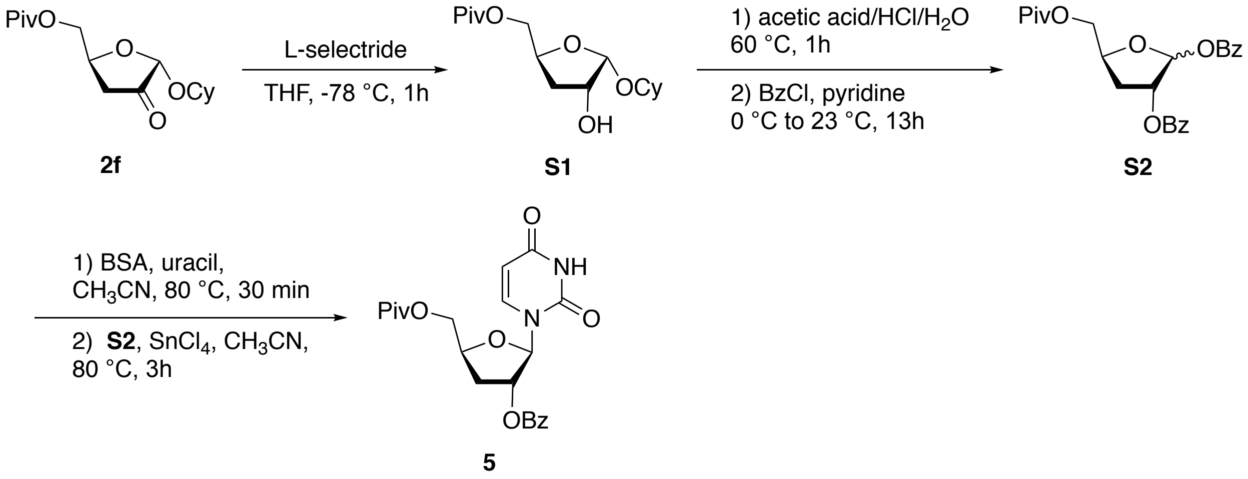

S1 – Cyclohexyl 3-deoxy 5-*O*-pivaloyl α-D-ribofuranoside

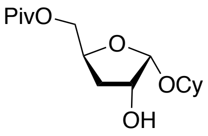

To a scintillation vial containing **2f** (44.4 mg, 0.149 mmol, 1 equiv.) and a magnetic stir bar was added THF (2.5 mL) under argon atmosphere. A solution of L-selectride in THF (0.30 mL, 1 M, 2.0 equiv.) was then added dropwise at –78 ºC, and the reaction mixture was stirred at –78 ºC for 1 hour. The mixture was quenched with saturated NH_4Cl aq. (5 mL) and then extracted with ethyl acetate (3 × 10 mL). The combined organic extracts were dried over magnesium sulfate, filtered, and concentrated under reduced pressure. ^{1}H NMR spectroscopic analysis of the unpurified reaction mixtures indicated formation of **5** as a single diastereomer. **5** was isolated by flash chromatography on silica gel (10% to 15% ethyl acetate in hexanes) as a pale yellow amorphous solid (37.4 mg, 84%).

^{1}H NMR (400 MHz, $CDCl_3$):δ(ppm) = 5.11 (d, J = 4.4 Hz, 1H, H-1), 4.44–4.34 (m, 1H, H-4), 4.27–4.20 (m, 1H, H-2), 4.11 (dd, J = 11.7, 3.7 Hz, 1H, H-5a), 4.01 (dd, J = 11.7, 4.7 Hz, 1H, H-5b), 3.72–3.59 (m, 1H, C_6H_{11}), 2.62 (d, J = 8.6 Hz, 1H, OH), 2.05–1.81 (m, 4H, H-3, C_6H_{11}), 1.75–1.65 (m, 2H, C_6H_{11}), 1.55–1.47 (m, 1H, C_6H_{11}), 1.46–1.10 (m, 14H, $C(CH_3)_3$, C_6H_{11}).

^{13}C NMR (101 MHz, $CDCl_3$):δ(ppm) = 178.4, 100.3, 76.2, 74.4, 71.8, 66.0, 38.9, 34.7, 33.8, 32.1, 27.3, 25.7, 24.1, 24.0.

IR (neat, cm^{-1}): 3520 (br, w), 2934 (s), 2859 (m), 1732 (s), 1481 (w), 1453 (w), 1400 (w), 1284 (m), 1159 (s), 1091 (m), 1072 (m), 1028 (s).

HRMS (DART$^+$, m/z): calculated for $C_{16}H_{29}O_5$ $[M + H]^+$: 301.2010. Found: 301.2009.

$[\alpha]_D^{20}$ = +34.0 (c= 0.430 g/100 mL, $CHCl_3$)

S2 – 1,2-*O*-Benzoyl 3-deoxy 5-*O*-pivaloyl D-ribofuranoside

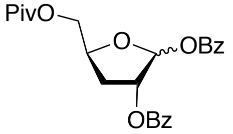

To a scintillation vial containing **S1** (67.9 mg, 0.226 mmol, 1 equiv.) and equipped with a magnetic stir bar was added acetic acid (0.90 mL), water (2.8 mL) and conc. HCl (0.05 mL) at room temperature. The reaction mixture was stirred at 60 ºC for 1 hour. It was then quenched with saturated $NaHCO_3$ aq. (5 mL) and extracted with ethyl acetate (3 × 10 mL). The combined organic extracts were dried over magnesium sulfate, filtered, and concentrated under reduced pressure to give a yellow amorphous solid which was used in the next step without further purification.

To a scintillation vial containing the crude material was added pyridine (1.1 mL), and the solution was cooled to 0 ºC. Benzoyl chloride (0.12 mL, 1.02 mmol, 4.5 equiv.) was added to the reaction mixture under argon atmosphere and it was stirred at room temperature for 13 hours. The mixture was then concentrated under reduced pressure and dried by azeotropic removal of pyridine with toluene. The resulting mixture was purified by flash chromatography on basic alumina (10% to 20% ethyl acetate in hexanes) to give the diastereomeric mixture of products as a colorless amorphous solid (58.8 mg, 61%).

^{1}H NMR (400 MHz, CD_3CN):δ(ppm) = 8.10–7.98 (m, 2.8H, ArH), 8.01–7.91 (m, 1.2H, ArH), 7.69–7.61 (m, 1.4H, ArH), 7.65–7.54 (m, 0.6H, ArH), 7.53–7.47 (m, 2.8H, ArH), 7.46–7.37 (m, 1.2H, ArH), 6.72 (d, J = 4.2 Hz, 0.3H, H-1(minor)), 6.52 (s, 0.7H, H-1(major)), 5.67–5.57 (m, 1H, H-2), 4.80–4.70 (m, 1H, H-4), 4.29–4.21 (m, 1H, H-5a), 4.21–4.09 (m, 1H, H-5b), 2.57–2.42 (m, 0.6H, H-3(minor)), 2.43–2.35 (m, 1.4H, H-3(major)), 1.24 (s, 2.7H, $C(CH_3)_3$(minor)), 1.10 (s, 6.3H, $C(CH_3)_3$(major)).

^{13}C NMR (101 MHz, CD_3CN):δ(ppm) = 178.8, 178.6, 166.4, 166.3, 166.0, 165.7, 134.6, 134.5 (two carbons), 134.4, 130.7 (two carbons), 130.6 (two carbons), 130.5, 130.4 (two carbons), 130.3, 129.6 (two carbons), 129.5, 101.1, 96.6, 80.0, 79.0, 77.3, 73.9, 66.8, 66.3, 39.5, 39.4, 32.3, 30.6, 27.5, 27.4.

HRMS (DART$^+$, m/z): calculated for $C_{24}H_{30}NO_7$ $[M + NH_4]^+$: 444.2017. Found: 444.2013.

5 – 1-(β-D-2'-*O*-Benzoyl-3'-deoxy-5'-*O*-pivaloylribosyl)uracil

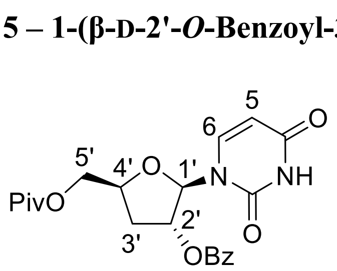

Procedure was adapted from literature.[10] To a scintillation vial containing uracil (25.7 mg, 0.229 mmol, 2.1 equiv.) were added acetonitrile (0.80 mL), *N*,*O*-bis(trimethylsilyl)acetamide (0.11 mL,

0.458 mmol, 4.2 equiv.) at room temperature under argon atmosphere. The reaction mixture was stirred at 80 ºC for 30 minutes. It was then cooled to room temperature, and a solution of **S2** (46.3 mg, 0.109 mmol, 1 equiv.) in acetonitrile (0.80 mL) was added. $SnCl_4$ (0.38 mL, 1 M, 3.5 equiv.) was then added dropwise, and the mixture was stirred at 80 ºC. After three hours the reaction mixture was quenched with saturated $NaHCO_3$ aq. (5 mL) and then extracted with ethyl acetate (3 × 10 mL). The combined organic extracts were dried over magnesium sulfate, filtered, and concentrated under reduced pressure. ^{1}H NMR spectroscopic analysis of the crude reaction indicated formation of **7** as a single diastereomer. The crude material was subjected to flash chromatography on silica gel (50% ethyl acetate in hexanes) to give **7** as a colorless amorphous solid (43.2 mg, 95%).

^{1}H NMR (400 MHz, CD_3CN):δ(ppm) = 9.66 (s, 1H, NH), 8.08–8.00 (m, 2H, ArH), 7.67–7.61 (m, 1H, ArH), 7.57 (d, *J* = 8.1 Hz, 1H, H-6), 7.54–7.44 (m, 2H, ArH), 5.99 (d, *J* = 2.1 Hz, 1H, H-1'), 5.67 (d, *J* = 8.1 Hz, 1H, H-5), 5.55 (dt, *J* = 6.2, 2.3 Hz, 1H, H-2'), 4.65–4.55 (m, 1H, H-4'), 4.33 (dd, *J* = 12.4, 2.9 Hz, 1H, H-5'a), 4.28 (dd, *J* = 12.4, 4.7 Hz, 1H, H-5'b), 2.37–2.13 (m, 2H, H-3'), 1.21 (s, 9H, $C(CH_3)_3$).

^{13}C NMR (101 MHz, CD_3CN):δ(ppm) = 178.8, 171.0, 166.3, 164.4, 151.3, 141.5, 134.5, 130.5, 129.6, 102.8, 91.7, 79.1, 79.0, 65.3, 39.5, 33.1, 27.5.

IR (neat, cm^{-1}): 2974 (w), 1721 (s), 1693 (s), 1453 (w), 1379 (w), 1263 (m), 1157 (m), 1108 (m), 810 (w), 714 (m).

HRMS (DART$^+$, m/z): calculated for $C_{21}H_{25}N_2O_7$ $[M + H]^+$: 417.1656. Found: 417.1656.

$[\alpha]_D^{20}$ = −12.8 (c= 1.21 g/100 mL, $CHCl_3$)

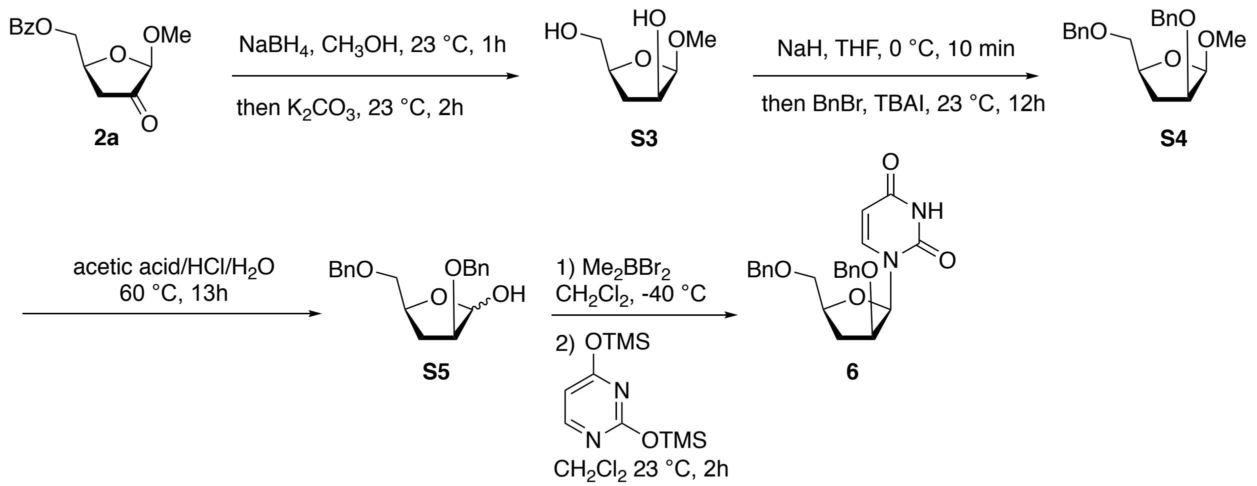

S3 – Methyl 3-deoxy β-D-arabinofuranoside

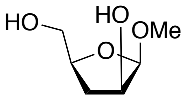

To a scintillation vial containing **2a** (107 mg, 0.427 mmol, 1 equiv.) and a magnetic stir bar was added methanol (8.5 ml) and $NaBH_4$ (24.2 mg, 0.641 mmol, 1.5 equiv.). The reaction mixture was stirred at room temperature for 1 hour. K_2CO_3 (118 mg, 0.854 mmol, 2.0 equiv.) was then added to the mixture, which was stirred at room temperature for 2 hours and then concentrated under reduced pressure. ^{1}H NMR spectroscopic analysis of the crude reaction indicated formation of **S3** as a single diastereomer. **S3** was isolated after flash chromatography on silica gel (50% ethyl acetate in hexanes, then 9% methanol in ethyl acetate) as a yellow oil (57.5 mg, 91%).

^{1}H NMR (400 MHz, $CDCl_3$):δ(ppm) = 4.77 (d, *J* = 4.5 Hz, 1H, H-1), 4.30–4.19 (m, 1H, H-2,4), 3.72 (dd, *J* = 11.8, 2.9 Hz, 1H, H-5a), 3.50 (s, 3H, OCH_3), 3.54–3.45 (m, 1H, H-5b), 2.27 (ddd, *J* = 12.4, 8.0, 7.1 Hz, 1H, H-3a), 1.77 (ddd, *J* = 12.4, 9.2, 9.2 Hz, 1H, H-3b).

^{13}C NMR (101 MHz, $CDCl_3$):δ(ppm) = 102.6, 78.6, 72.8, 65.3, 55.8, 32.3.

IR (neat, cm^{-1}): 3353 (br, m), 2924 (s), 2854 (m), 1651 (br, w), 1455 (m), 1365 (w), 1238 (w), 1197 (w), 1104 (m), 1026 (s), 881 (w), 804 (m).

HRMS (DART$^+$, m/z): calculated for $C_6H_{13}O_4$ $[M + H]^+$: 149.0808. Found: 149.0806.

$[\alpha]_D^{20}$ = −41.3 (c= 0.755 g/100 mL, $CHCl_3$)

S4 – Methyl 2,5-di-*O*-benzyl 3-deoxy β-D-arabinofuranoside

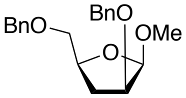

To a scintillation vial containing NaH (77.6 mg, 1.94 mmol, 5.0 equiv.) and a magnetic stir bar were added THF (1.9 mL) and **S3** (57.5 mg, 0.388 mmol, 1 equiv.) in THF (2.0 mL) at 0 ºC under argon atmosphere. The reaction mixture was stirred at 0 ºC for 10 minutes. Then, benzyl bromide (0.14 ml, 1.16 mmol, 3.0 equiv.) and tetrabutylammonium iodide (28.7 mg, 0.0776 mmol, 0.2 equiv.) were added to the mixture at 0 ºC and it was stirred at room temperature for 12 hours. It was quenched with methanol (1 mL), diluted with water (10 mL) and then extracted with ethyl acetate (3 × 10 mL). The combined organic extracts were dried over magnesium sulfate, filtered, and concentrated under reduced pressure and purified by flash chromatography on silica gel (10% to 30% ethyl acetate in hexanes) to give **S4** as a colorless oil (106.8 mg, 84%).

^{1}H NMR (400 MHz, CD_3CN):δ(ppm) = 7.39–7.26 (m, 10H, ArH), 4.78 (d, *J* = 4.1 Hz, 1H, H-1), 4.61–4.46 (m, 4H, CH_2Ar), 4.21–4.12 (m, 1H, H-4), 3.99 (ddd, *J* = 10.6, 7.8, 4.1 Hz, 1H, H-2), 3.53–3.42 (m, 2H, H-5), 3.29 (s, 3H, OCH_3), 2.25 (ddd, *J* = 11.7, 7.8, 6.9 Hz, 1H, H-3a), 1.65 (ddd, *J* = 11.7, 10.6, 9.2 Hz, 1H, H-3b).

^{13}C NMR (101 MHz, CD_3CN):δ(ppm) = 139.8, 139.6, 129.3, 128.9, 128.7, 128.6, 128.5, 102.1, 79.9, 76.7, 75.7, 73.7, 72.6, 54.7, 32.0.

IR (neat, cm^{-1}): 2926 (s), 2855 (m), 1497 (w), 1454 (m), 1365 (m), 1310 (w), 1261 (w), 1205 (w), 1181 (w), 1119 (s), 1099 (s), 1064 (s), 1047 (s), 1029 (s), 1012 (s), 888 (w), 811 (w), 738 (s), 698 (s).

HRMS ($DART^+$, m/z): calculated for $C_{20}H_{28}NO_4$ $[M + NH_4]^+$: 346.2013. Found: 346.2011.

$[\alpha]_D^{20}$ = −17.4 (c= 0.780 g/100 mL, $CHCl_3$)

S5 – 2,5-Di-*O*-benzyl 3-deoxy D-arabinofuranose

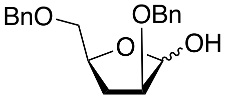

To a scintillation vial containing **S4** (107 mg, 0.325 mmol, 1 equiv.) and a magnetic stir bar was added acetic acid (1.3 mL), water (3.3 mL) and 1N HCl (0.81 mL) at room temperature. The reaction mixture was stirred at 60 ºC. After 13 hours, the mixture was quenched with saturated $NaHCO_3$ aq. (5 mL) and then extracted with ethyl acetate (3 × 10 mL). The combined organic extracts were dried over magnesium sulfate, filtered, and concentrated under reduced pressure. Flash chromatography on silica gel (20% to 50% ethyl acetate in hexanes) provided a diastereomeric mixture of products as a colorless amorphous solid (76.4 mg, 75%).

^{1}H NMR (400 MHz, CD_3CN): δ(ppm) = 7.38–7.25 (m, 10H, ArH), 5.31 (d, *J* = 4.3 Hz, 1H, H-1), 4.52 (s, 2H, CH_2Ar), 4.49 (s, 2H, CH_2Ar), 4.37–4.29 (m, 1H, H-4), 3.98 (d, *J* = 4.3 Hz, 1H, OH), 3.90 (dd, *J* = 6.5, 2.8 Hz, 1H, H-2), 3.57–3.43 (m, 2H, H-5), 2.29 (ddd, *J* = 13.8, 7.9, 6.3 Hz, 1H, H-3a), 1.70 (ddd, *J* = 13.5, 6.0, 2.9 Hz, 1H, H-3b).

^{13}C NMR (101 MHz, CD_3CN):δ(ppm) = 139.7, 139.5, 129.3 (three carbons), 128.8, 128.7 (two carbons), 128.6 (three carbons), 128.5, 128.4, 101.9, 96.3, 84.8, 79.7, 77.8, 76.2, 74.4, 74.1, 73.8, 73.7, 72.4, 71.8, 33.4, 31.4.

Select peaks for the minor product:

^{1}H NMR (400 MHz, CD_3CN):δ(ppm) = 5.15 (dd, *J* = 8.2, 4.2 Hz, 0.3H, H-1), 4.15–4.07 (m, 0.3H, H-4), 1.82–1.74 (m, H-3a).

HRMS ($DART^+$, m/z): calculated for $C_{19}H_{26}NO_4$ $[M + NH_4]^+$: 332.1856. Found: 332.1850.

6 – 1-(β-D-2',5'-Di-*O*-benzyl-3'-deoxyarabinosyl)uracil

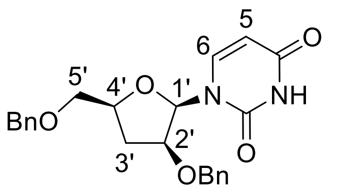

Procedure was adapted from literature.[11] To a scintillation vial containing uracil (15.5 mg, 0.138 mmol, 3.0 equiv.) were added acetonitrile (0.66 mL), *N*,*O*-bis(trimethylsilyl)acetamide (0.08 mL,

0.323 mmol, 7.0 equiv.) at room temperature under argon atmosphere. The reaction mixture was stirred at 80 ºC for 30 minutes. Then, it was concentrated under reduced pressure and dried in vacuo for 1 h to give the mixture of *O*,*O*'-bis(trimethylsilyl)uracil and byproducts. To the other scintillation vial containing 2,5-di-*O*-benzyl 3-deoxy D-arabinofuranose **S5** (14.5 mg, 0.0461 mmol, 1 equiv.) was added DCM (0.46 mL) and it was cooled to –40 ºC. Bromodimethylborane in DCM (0.05 mL, 1.7 M, 1.8 equiv.) was added dropwise to the mixture under argon atmosphere and it was stirred at 0 ºC for 30 minutes. Then, to the mixture was added *O*,*O*'-bis(trimethylsilyl)uracil in DCM (0.20 mL) at 0 ºC under argon atmosphere. It was stirred at room temperature for 2 hours and then quenched with saturated $NaHCO_3$ aq. (5 mL) and extracted with ethyl acetate (3 × 10 mL). The combined organic extracts were dried over magnesium sulfate, filtered, and concentrated under reduced pressure. ^{1}H NMR spectroscopic analysis of the unpurified product indicated formation of **6** as a pair of diastereomers in a 18 : 1 (1',2'-*cis* : 1',2'-*trans*) ratio. Purification of the crude mixture using flash chromatography on silica gel (50% ethyl acetate in hexanes) gave **6** as a colorless amorphous solid (13.2 mg, 70%).

^{1}H NMR (400 MHz, CD_3CN):δ(ppm) = 9.36 (s, 1H, NH), 7.76 (d, *J* = 8.1 Hz, 1H, H-6), 7.42–7.21 (m, 8H, ArH), 7.23–7.15 (m, 2H, ArH), 6.14 (d, *J* = 5.5 Hz, 1H, H-1'), 5.34 (d, *J* = 8.1 Hz, 1H, H-5), 4.55 (s, 2H, CH_2Ar), 4.53–4.31 (m, 3H, H-2', CH_2Ar), 4.20 (dddd, *J* = 8.3, 6.7, 4.9, 3.2 Hz, 1H, H-4'), 3.72 (dd, *J* = 10.7, 3.3 Hz, 1H, H-5'a), 3.65 (dd, *J* = 10.8, 5.0 Hz, 1H, H-5'b), 2.32 (dt, *J* = 13.4, 6.8 Hz, 1H, H-3'a), 2.03–1.93 (m, 1H, H-3'b).

^{13}C NMR (101 MHz, CD_3CN):δ(ppm) = 164.2, 151.7, 143.2, 139.4, 139.0, 129.4, 129.3, 128.8, 128.7, 128.6, 128.4, 101.1, 85.9, 79.0, 77.6, 73.9, 73.0, 71.8, 32.9.

IR (neat, cm^{-1}): 3179 (br, w), 3063 (br, w), 2927 (br, w), 1682 (s), 1456 (m), 1382 (w), 1275 (m), 1105 (m), 1067 (m), 812 (w), 739 (w), 699 (w).

HRMS ($DART^+$, m/z): calculated for $C_{23}H_{25}N_2O_5$ $[M + H]^+$: 409.1758. Found: 409.1770.

$[\alpha]_D^{20}$ = +31.3 (c= 0.640 g/100 mL, $CHCl_3$)

Additional Investigations into Substrate Scope

General Procedure F

Carbohydrate (0.1 mmol, 1 equiv.), (Ir[dF(CF_3)ppy]$_2$(dtbpy))PF_6 (1 mg, 0.001 mmol, 1 mol%), quinuclidine (3.3 mg, 0.03 mmol, 30 mol%), pentafluorophenyl boronic acid (4.2 mg, 0.01 mmol, 20 mol%) and a small magnetic stir bar were combined in a ½ dram vial. A rubber septum was used to seal the vial, which was then evacuated and backfilled with argon three times on a Schlenk line. Dry, degassed dichloromethane was added under a balloon of argon. The balloon was removed, the rubber septum was quickly replaced with the vial cap and sealed with Teflon tape. The vial was placed 5 inches from a blue LED Kessil lamp and stirred at 1050 rpm (the temperature under the light was monitored and remained at ~25 °C throughout the course of the reaction). After 6 hours, the crude reaction mixture was concentrated under reduced pressure, and analyzed by ^{1}H NMR spectroscopy.

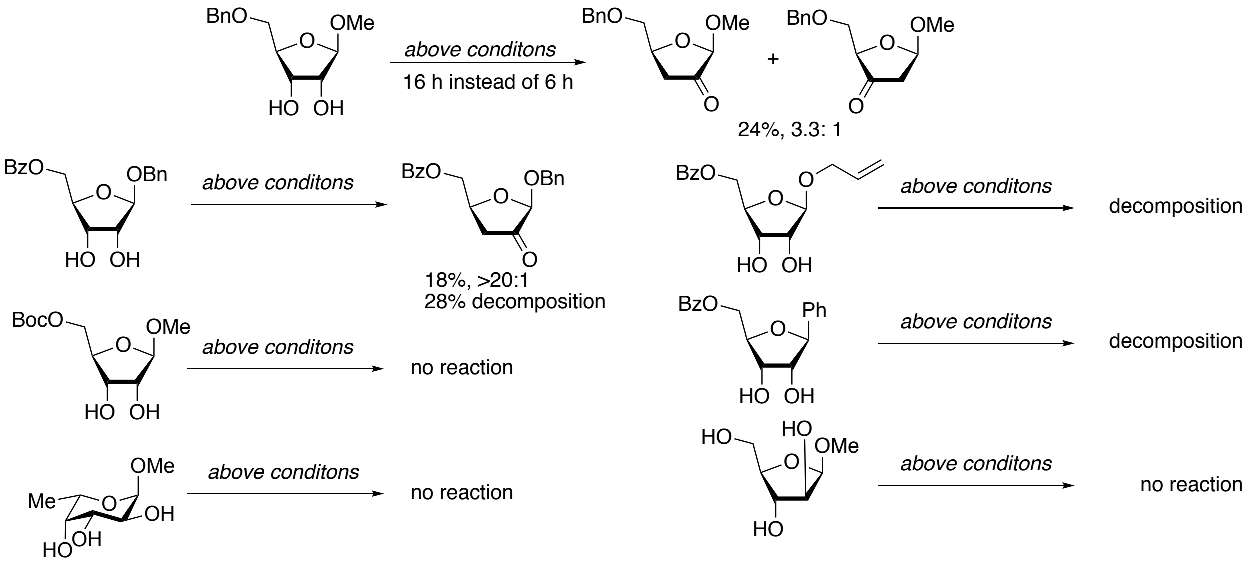

Quantum Yield Determination

Quantum yield and Stern-Volmer quenching experiments were performed according to modified literature procedures.[12]

Preparation of Ferrioxalate and Buffered Phenanthroline Solutions:

A 0.15M ferrioxalate solution was prepared by dissolving 1.842 g of potassium trioxalatoferrate(III) trihydrate in 25 mL of 0.05M H_2SO_4. A buffered phenanthroline solution was prepared by dissolving 25.0 mg of 1,10-phenanthroline and 5.625 g of sodium acetate in 25 mL of 0.5M H_2SO_4. Both solutions were stored in the dark when not in use.

Characterization of the Ferrioxalate Solution:

0.5 mL of the ferrioxalate solution was transferred to a cuvette, diluted to 2.0 mL using 0.05M H_2SO_4 and its absorption spectrum was measured. This process was repeated twice. The absorbance of the ferrioxalate solution at 450 nm, on average, was found to be 2.32808423 (0.582021058 following dilution).

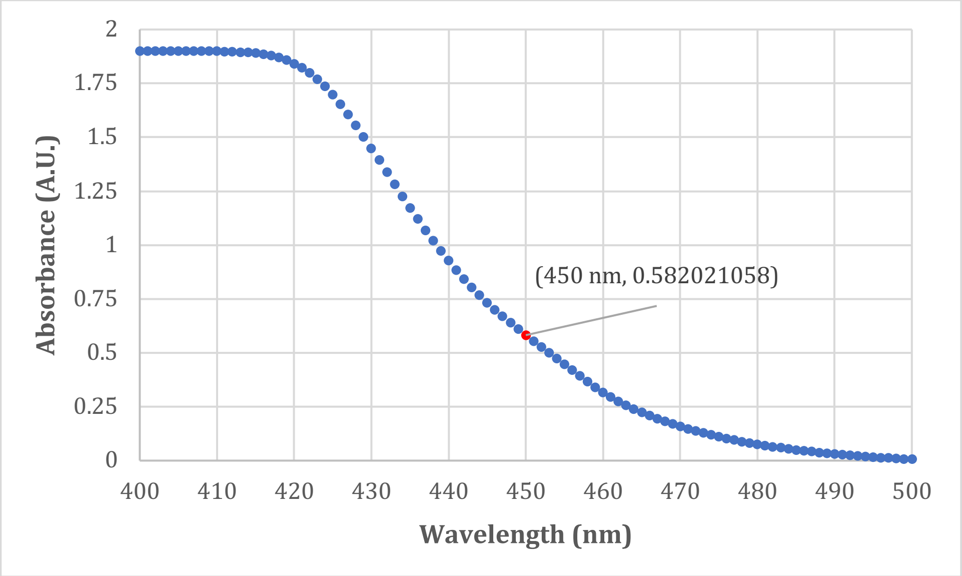

Figure S1. The averaged absorption spectrum of the prepared ferrioxalate solution.

The fraction of light absorbed by the ferrioxalate solution at 450 nm ($f_{ferrioxalate}$) was found to be 0.99530197, using the following equation:

$$\begin{aligned} f_{ferrioxalate} &= 1 - 10^{-A} \\ &= 1 - 10^{-2.32808423} \\ &= 0.99530197 \end{aligned}$$

where A is the absorbance at 450 nm.

Characterization of the Iridium Catalyst:

2.0 mg of $(Ir[dF(CF_3)ppy]_2(dtbpy))PF_6$ was dissolved in 1.6 mL of dry, degassed dichloromethane and the solution was transferred to a cuvette. The absorbance of the solution at 450 nm was measured and found to be 0.476994902

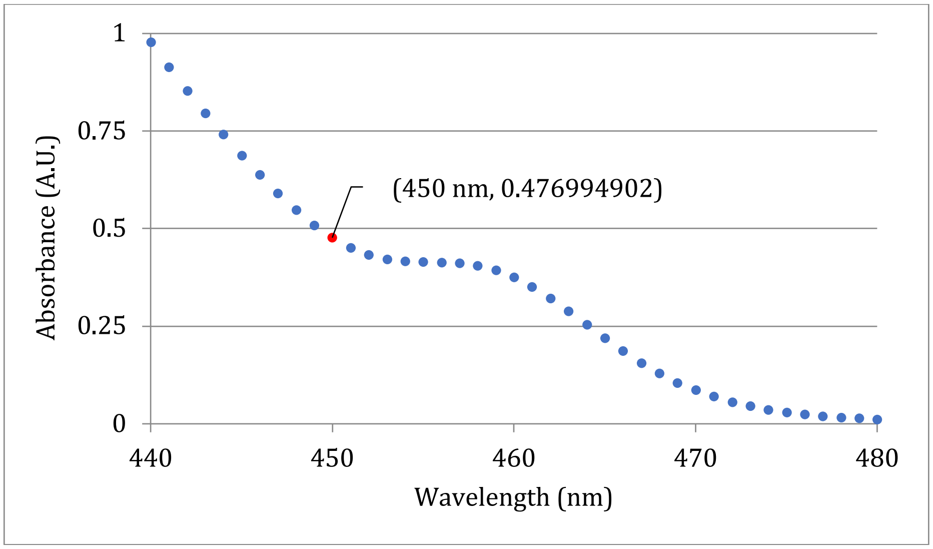

Figure S2. The averaged absorption spectrum of the prepared catalyst solution.

The fraction of light absorbed by the catalyst solution at 450 nm ($f_{catatalyst}$) was found to be 0.666569673, using the following equation:

$$f_{catalyst} = 1 - 10^{-A}$$

$$= 1 - 10^{-0.476994902}$$

$$= 0.666569673$$

Determination of Photon Flux:

1.0 mL of the ferrioxalate solution was transferred to a ½ dram vial and the vial was placed 5 inches from a 450 nm laser. After irradiating the solution for 90 s, 0.175 mL of the phenanthroline solution was added and the resulting solution was stored in the dark for 1 hour. 0.5 mL of the solution was then transferred to a cuvette, diluted to 2.0 mL using 0.05M H_2SO_4 and its absorbance at 510 nm was measured. This process was repeated twice.
On average, the absorbance at 510 nm was found to be 2.793991804 (0.698497951 following dilution).

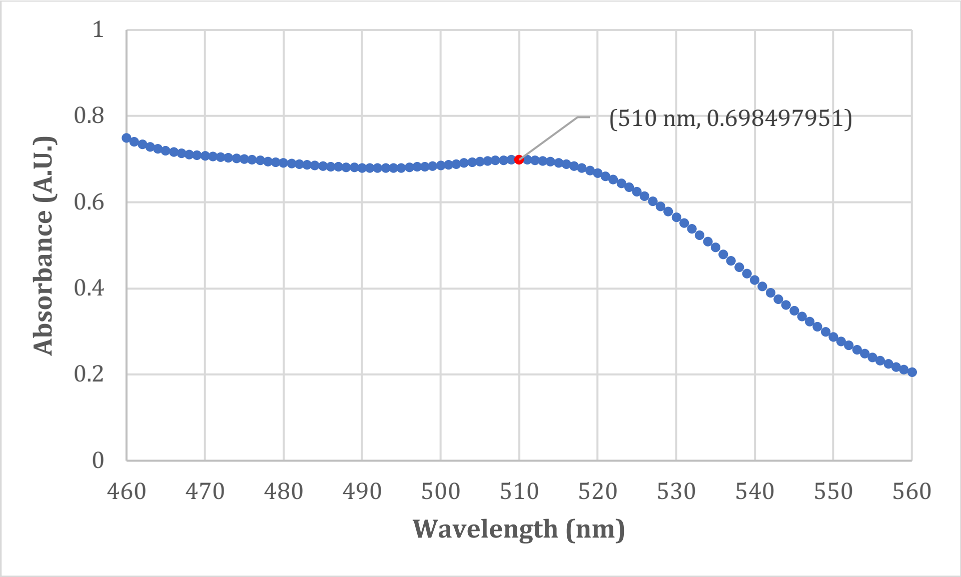

Figure S3. The averaged absorption spectrum of the ferrioxalate solution following irradiation and phenanthroline addition.

The above process was repeated, but without irradiating the solution. This process was repeated twice. On average, the absorbance at 510 nm was found to be 1.00764519 (0.251911297 following dilution).

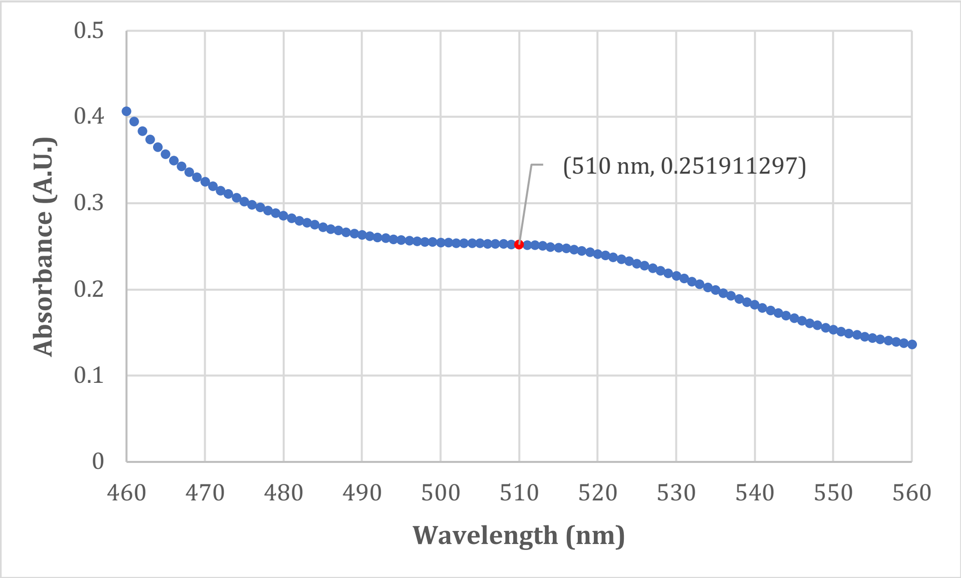

Figure S4. The averaged absorption spectrum of the ferrioxalate solution following phenanthroline addition.

The amount of Fe^{2+} formed during irradiation was found to be 1.89095×10^{-7} mol, using the following equation:

$$\text{mol Fe}^{2+} = \frac{(V)(\Delta A)}{(l)(\epsilon)}$$

$$= \frac{(0.001175\ \mathrm{L})(1.786346614)}{(1.0\ \mathrm{cm})\left(11100\,\frac{\mathrm{L}}{\mathrm{mol} * \mathrm{cm}}\right)}$$

$$= 1.89095 \times 10^{-7}\ \mathrm{mol}$$

where V is the sample volume (L), ΔA is the absorbance difference at 510 nm (between irradiated and non-irradiated ferrioxalate samples), l is the pathlength (cm) and ε is the molar absorptivity at 510 nm.

The photon flux of the system was found to be 2.18754 × 10^{-9} einstein s^{-1}, using the following equation:

$$\text{Photon Flux} = \frac{\mathrm{mol\ Fe^{2+}}}{(\Phi_{\mathrm{ferrioxalate}})(\mathrm{t})(\mathrm{f_{ferrioxalate}})}$$

$$= \frac{1.89095 \times 10^{-7}\ \mathrm{mol}}{(0.965)(90\ \mathrm{s})(0.99530197)}$$

$$= 2.18754 \times 10^{-9}\ \frac{\mathrm{einstein}}{\mathrm{s}}$$

where $\Phi_{ferrioxalate}$ is the quantum yield of the ferrioxalate actinometer at 450 nm (approximately 0.965)[13] and t is the irradiation time (s).

Determination of Quantum Yield:

Methyl 5-O-benzoyl β-D-ribofuranoside (26.8 mg, 0.1 mmol, 1 equiv.), $(Ir[dF(CF_3)ppy]_2(dtbpy))PF_6$ (1 mg, 0.001 mmol, 1 mol%), quinuclidine (3.3 mg, 0.03 mmol, 30 mol%), pentafluorophenyl boronic acid (4.2 mg, 0.02 mmol, 20 mol%) were combined in a ½ dram vial. A small rubber septum was used to seal the vial, which was then evacuated and backfilled with argon three times on a Schlenk line. 0.8 mL of dry, degassed dichloromethane was added under a balloon of argon. The balloon was removed, the rubber septum was quickly replaced with the vial cap and sealed with Teflon tape. The vial was placed 5 inches from a 450 nm laser and stirred at 1050 rpm (the temperature under the light was monitored and remained at ~25 °C throughout the course of the reaction). After 4 hours, the crude reaction mixture was concentrated under reduced pressure, and analyzed by ^{1}H NMR spectroscopy. This NMR yield was determined to be 0.000019 mol (19%).

The quantum yield of the reaction was found to be 0.904877158, using the following equation:

$$\Phi_{\mathrm{rxn}} = \frac{\mathrm{mol\ Product}}{(\text{Photon Flux})(\mathrm{t_{rxn}})(\mathrm{f_{catalyst}})}$$

$$= \frac{0.000019\ \mathrm{mol}}{\left(2.18754 \times 10^{-9}\ \frac{\mathrm{einstein}}{\mathrm{s}}\right)(14400\ \mathrm{s})(0.666569673)}$$

$$= 0.904877158$$

where t_{rxn} is the reaction time (s).

Stern-Volmer Data and Chain Length Approximations

Stern-Volmer Quenching Data:

The Stern-Volmer constant, K_{SV}, $(Ir[dF(CF_3)ppy]_2(dtbpy))PF_6$ by quinuclidine in CH_2Cl_2 was determined using Stern-Volmer kinetics

$$\frac{I_0}{I} = K_{SV}[\text{quencher}] = k_q\tau_0[\text{quencher}]$$

where I_0 is the luminescence intensity without quencher, I is the luminescence intensity with quencher, k_q is the rate of quenching of the photocatalyst by the quencher and τ_0 is the lifetime of the photocatalyst. Samples were prepared by adding degassed solutions of photocatalyst, quinuclidine and CH_2Cl_2 into a cuvette under inert atmosphere. The concentration of photocatalyst in the cuvette was 2.5 x 10^{-4} M. Samples were irradiated at 450 nm and emissions were detected at 492 nm (local maximum).

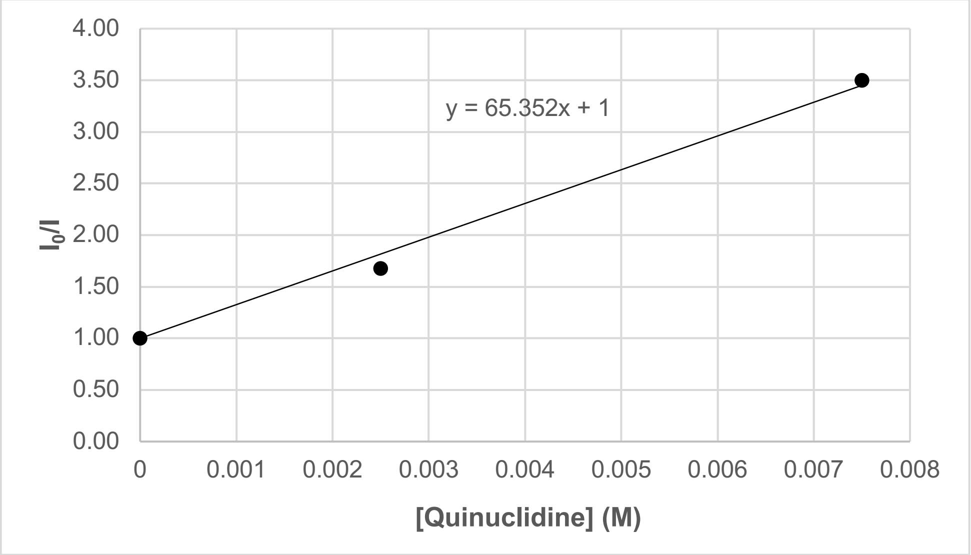

Figure S5. Stern-Volmer quenching experiment of $(Ir[dF(CF_3)ppy]_2(dtbpy))PF_6$ and quinuclidine.

The quenching of $(Ir[dF(CF_3)ppy]_2(dtbpy))PF_6$ by quinuclidine in CH_2Cl_2, excited at 450 nm, is described by a Stern-Volmer constant of K_{SV} of 65.352 M^{-1}.

Chain Length Approximations

Chain lengths were calculated using the following equation

$$\text{Chain Length} = \frac{\Phi}{Q} = \Phi\left(\frac{I_0}{I_0 - I}\right)$$

where Φ is 0.9 (calculated *vide supra*) and Q is the quenching fraction.

At 10 equiv. of quinuclidine relative to photocatalyst (10 mol% quinuclidine under standard reaction conditions), Q is 0.40 and the chain length is 2.25.

At 30 equiv. of quinuclidine relative to photocatalyst (30 mol% quinuclidine under standard reaction conditions), Q is 0.71 and the chain is length is 1.27.

Kinetic Isotope Effect Experiments

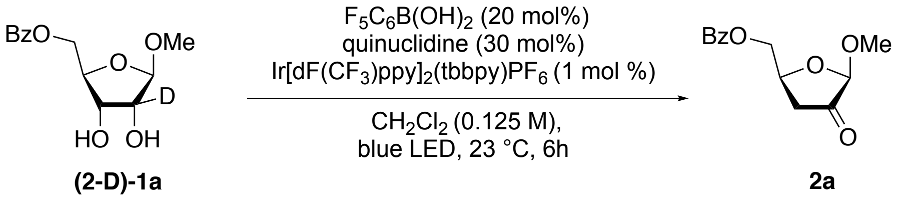

(2-D)-1a (26.9 mg, 0.1 mmol, 1 equiv.), (Ir[dF(CF_3)ppy]$_2$(dtbpy))PF_6 (1 mg, 0.001 mmol, 1 mol%), quinuclidine (3.3 mg, 0.03 mmol, 30 mol%), pentafluorophenyl boronic acid (4.2 mg, 0.02 mmol, 20 mol%) and a small magnetic stir bar were combined in a ½ dram vial. A small rubber septum was used to seal the vial, which was then evacuated and backfilled with argon three times on a Schlenk line. 0.8 mL of dry, degassed dichloromethane was added under a balloon of argon. The balloon was removed, the rubber septum was quickly replaced with the vial cap and sealed with Teflon tape. The vial was placed 5 inches from a blue LED Kessil lamp and stirred at 1050 rpm (the temperature under the light was monitored and remained at ~25 °C throughout the course of the reaction). After 6 hours, the crude reaction mixture was concentrated under reduced pressure, and analyzed by ^{1}H NMR spectroscopy (~8% $^2H_1$ content in product). The crude material was then subjected to flash chromatography on silica gel and the product was isolated in 50% yield (<5% $^2H_1$ content).

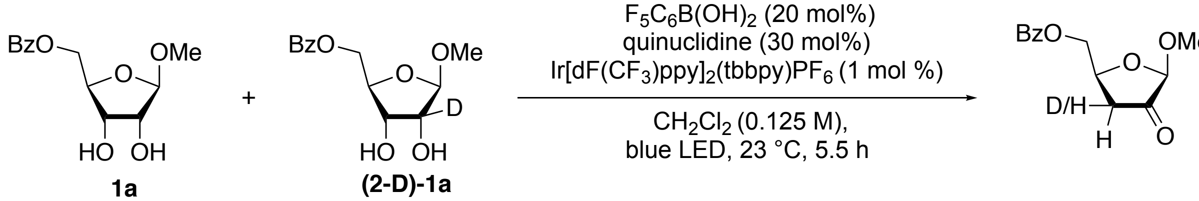

1a (16.8 mg, 0.0625 mmol, 1 equiv.) and **(2-D)-1a** (16.8 mg, 0.0625 mmol, 1 equiv.) were combined in a 1 dram vial, which was sealed with a rubber septum and evacuated and backfilled with argon three times. 1 mL of dry, degassed dichloromethane was added to the flask (solution ratio, 1.3:1 **1a**: (**2D)-1a**). (Ir[dF(CF_3)ppy]$_2$(dtbpy))PF_6 (1 mg, 0.001 mmol, 1 mol%), quinuclidine (3.3 mg, 0.03 mmol, 30 mol%), pentafluorophenyl boronic acid (4.2 mg, 0.02 mmol, 20 mol%), and a small magnetic stir bar were combined in a 0.5 dram vial. A small rubber septum was used to seal the vial, which was then evacuated and backfilled with argon three times on a Schlenk line. 0.8 mL of the solution of ribofuranosides was added to the 0.5 dram vial under a balloon of argon. The balloon was removed, the rubber septum was quickly replaced with the vial cap and sealed with Teflon tape. The vial was placed 5 inches from a blue LED Kessil lamp and stirred at 1050 rpm (the temperature under the light was monitored and remained at ~25 °C throughout the course of the reaction). After 6 hours, the crude reaction mixture was concentrated under reduced pressure, and analyzed by ^{1}H NMR spectroscopy. The crude material was then subjected to flash chromatography on silica gel and the starting material was recovered (16%, 1:1.2, **1a**: (**2D)-1a**). The apparent KIE was calculated to be 1.14.

H/D Exchange Experiment

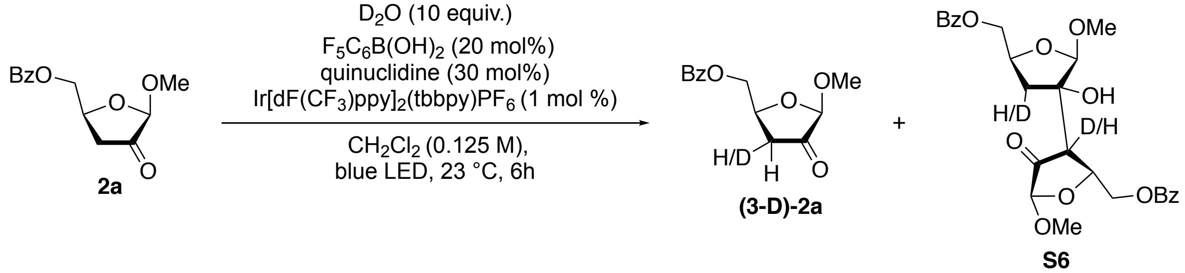

2a (25.0 mg, 0.1 mmol, 1 equiv.), (Ir[dF(CF_3)ppy]$_2$(dtbpy))PF_6 (1 mg, 0.001 mmol, 1 mol%), quinuclidine (3.3 mg, 0.03 mmol, 30 mol%), pentafluorophenyl boronic acid (4.2 mg, 0.02 mmol, 20 mol%), and a small magnetic stir bar were combined in a ½ dram vial. A small rubber septum was used to seal the vial, which was then evacuated and backfilled with argon three times on a Schlenk line. 0.8 mL of dry, degassed dichloromethane was added under a balloon of argon along with deuterium dioxide (10 equiv.). The balloon was removed, the rubber septum was quickly replaced with the vial cap and sealed with Teflon tape. The vial was placed 5 inches from a blue LED Kessil lamp and stirred at 1050 rpm (the temperature under the light was monitored and remained at ~25 °C throughout the course of the reaction). After 6 hours, the crude reaction mixture was concentrated under reduced pressure, and analyzed by ^{1}H NMR, ^{2}H NMR spectroscopy and mass spectrometry. Analysis of the crude ^{1}H NMR spectrum indicated a mixture of ketone and dimer illustrated above. Multiple signals in the ^{2}H NMR (^{1}H decoupled) spectrum (Figure S6) in the region of 2.7-2.4 ppm suggests α-deuteration of **2a** as well as the aldol dimer (**S6**). The presence of **(3-D)-2a** (2a: mono-D: di-D, 1.97:2.04:1) as well as **S6** (non-D-S6, mono-D: di-D, 1.25:1.53:1) were also observed in low resolution mass spectra of the crude reaction mixture (Figure S7).

Figure S6: Crude ^{2}H NMR (500 MHz, CH_2Cl_2) for H/D exchange experiment between 2a and D_2O under reaction conditions.

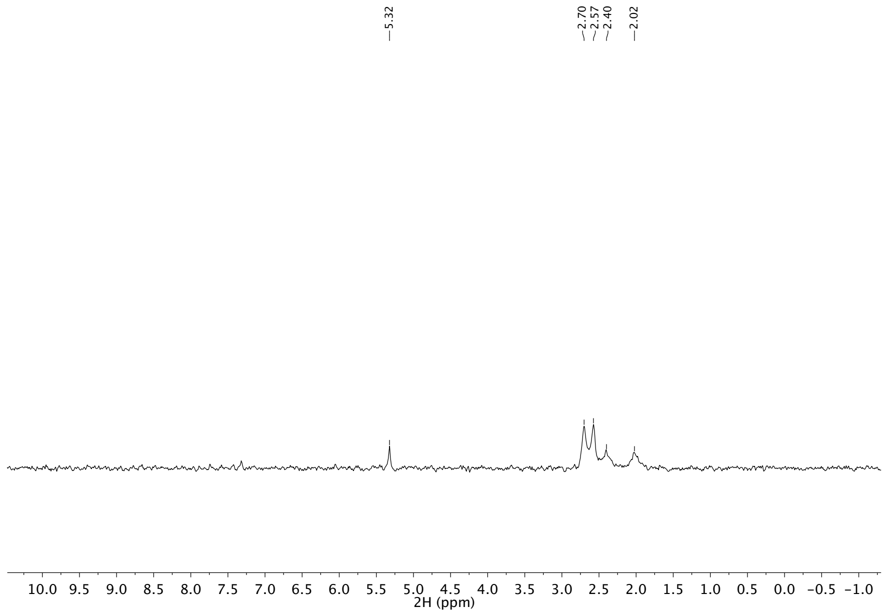

Figure S7: Low resolution mass spectra (DART ionization) of the crude reaction mixture for H/D exchange experiment between 2a and D_2O under reaction conditions.

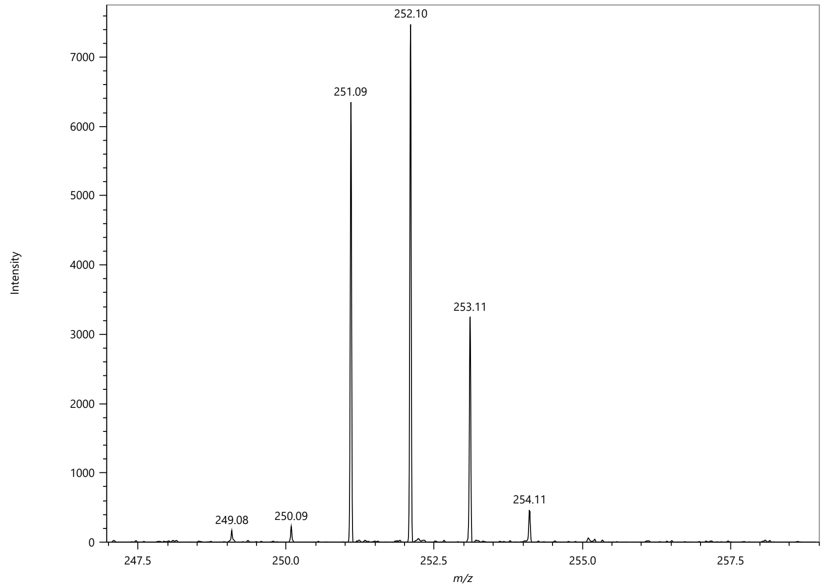

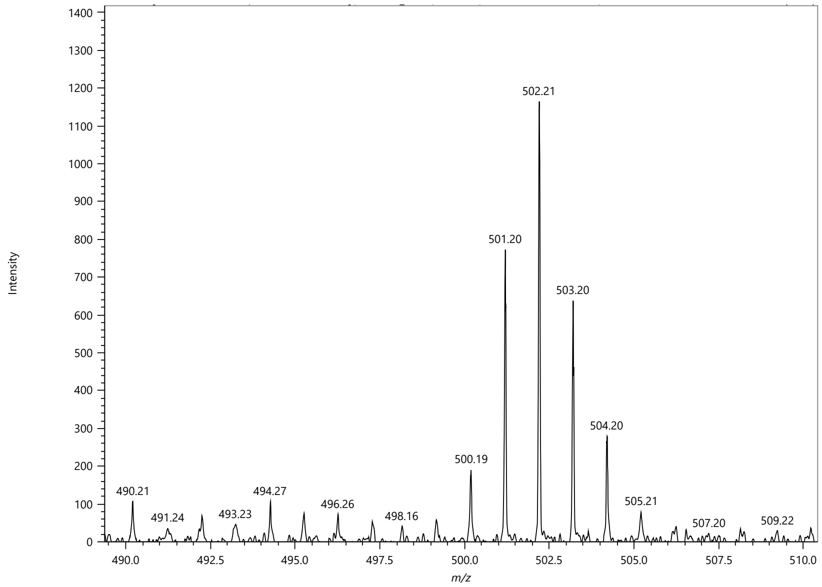

Computational Data

DFT calculations were carried out using the Gaussian 16 suite of programs[14] at the B97-D3/Def2-TZVP level of theory.[15,16] Vibrational frequency calculations were carried out for each stationary point to ensure they were either an energy minimum (no imaginary frequencies) or a transition state (one imaginary frequency) on the potential energy surface. The Intrinsic Reaction Coordinate (IRC) method was used to verify all transition states. Frequency calculations were carried out at 1 atm and 298.15 K. Structures were visualized using GaussView 5.0 and Avogadro 1.2.0.
Geometry optimizations and frequency calculations were carried out in the gas phase for all proposed reaction intermediates and transition states. The calculated energies of these species (depicted in Figure S8 and S9) are listed in Table S4.

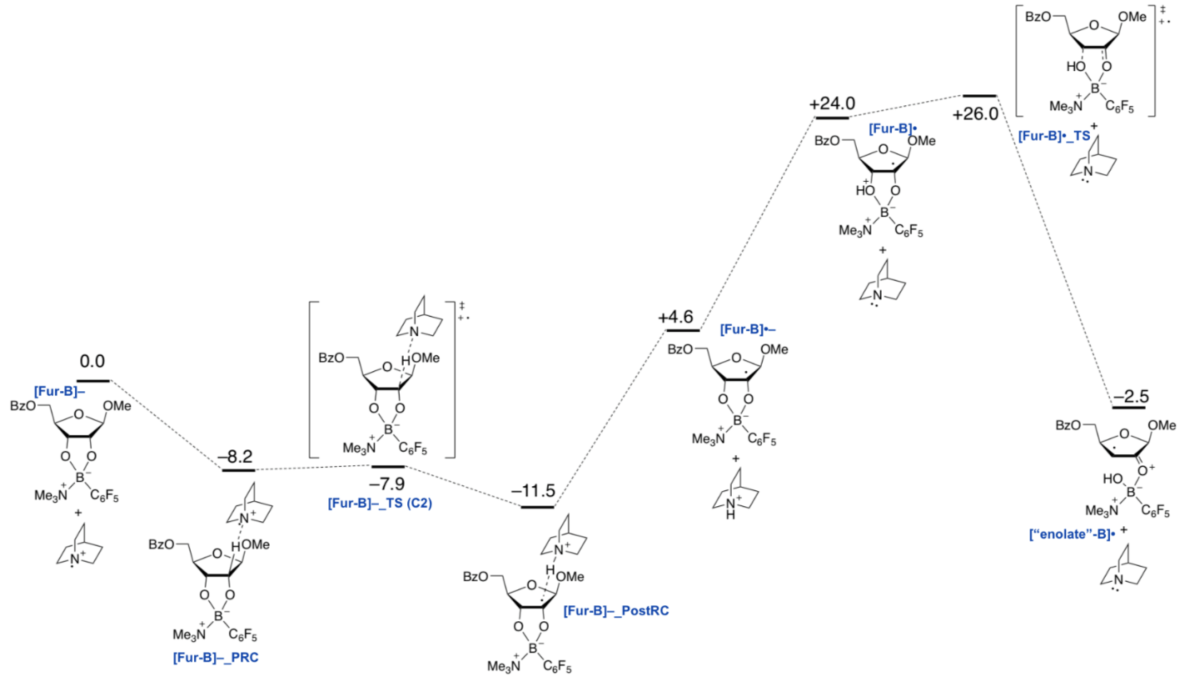

Figure S8. Calculated free energy profile for HAT at the C2 position of methyl 5-O-benzoyl β-D-ribofuranoside (gas phase).

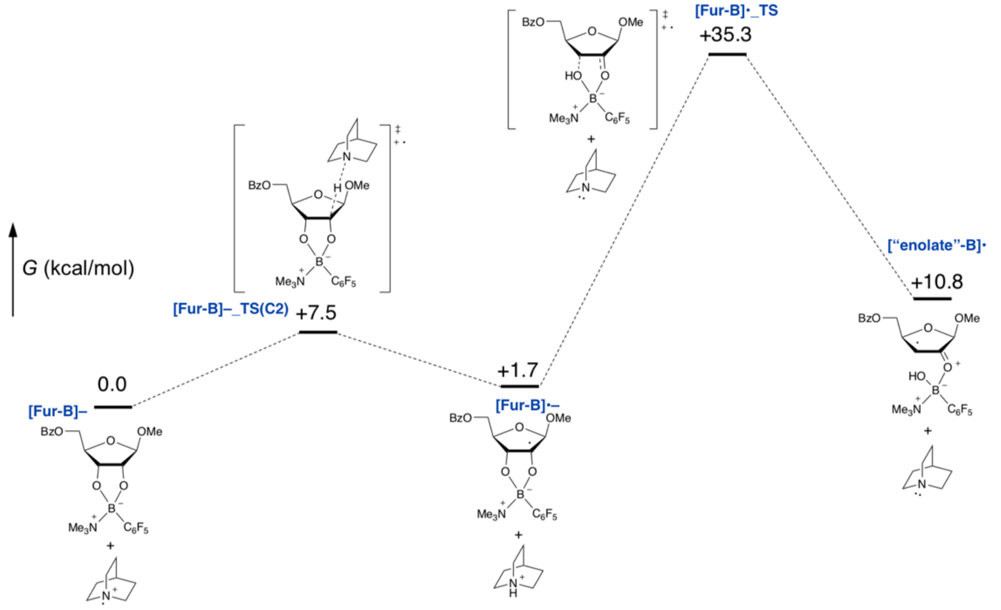

Figure S9. Calculated free energy profile for HAT at the C2 position of methyl 5-O-benzoyl β-D-ribofuranoside (SMD (dichloromethane)).

Table S4. Calculated energies of reaction intermediates depicted in Figure S5 and S6.

Species	Sum of electronic and zero-point energies[a]	Sum of electronic and thermal energies[a]	Sum of electronic and thermal enthalpies[a]	Sum of electronic and thermal free energies[a]	Electronic single-point energies SMD (CH_2Cl_2)	Free energy correction	SMD(CH_2Cl_2) free energies
**[Fur-B]⁻**	-1689.989736	-1689.959342	-1689.958398	-1690.053764	-1690.395872	0.31413	-1690.081742
**quinuclidine$^{•+}$**	-328.757904	-328.751037	-328.750093	-328.789853	-329.0365844	0.158914	-328.8776704
**[Fur-B]⁻ _PRC**	-2018.780998	-2018.742034	-2018.74109	-2018.856632	-2019.442952	0.494295	-2018.948657
**[Fur-B]⁻_TS (C2)**	-2018.782311	-2018.743793	-2018.742849	-2018.85622	-2019.440803	0.493358	-2018.947445
**[Fur-B]⁻ _PostRC**	-2018.786526	-2018.747562	-2018.746618	-2018.862021	-2019.452471	0.497351	-2018.95512
**[Fur-B]$^{•-}$**	-1689.339818	-1689.309462	-1689.308518	-1689.40372	-1689.734098	0.301524	-1689.432574
**Quinuclidine-H^{+}**	-329.401807	-329.395014	-329.39407	-329.432556	-329.6999333	0.175883	-329.5240503
**Quinuclidine**	-329.023652	-329.016939	-329.015995	-329.054531	-329.2248945	0.15998	-329.0649145
**[Fur-B]$^{•}$**	-1689.687109	-1689.656481	-1689.655537	-1689.750757	-1690.151313	0.314184	-1689.837129
**[Fur-B]$^{•}$_TS**	-1689.684105	-1689.653697	-1689.652753	-1689.747724	-1690.150823	0.312614	-1689.838209
**[“enolate”-B]$^{•}$**	-1689.726682	-1689.694879	-1689.693935	-1689.792995	-1690.185007	0.307656	-1689.877351

[a] Energies in Hartree, calculated in the gas phase.

Table S5. Calculated energies of transition states for HAT from C-2 versus C-3 in acetonitrile.

	C2	C3	ΔG (kcal/mol)
**None**	-2019.349578	-2019.354624	3.16618
**CPCM**	-2019.421395	-2019.420312	-0.67957
**PCM**	-2019.42102	-2019.419985	-0.64934
**SMD**	-2019.446305	-2019.444806	-0.94057

Table S6. Calculated energies of transition states for HAT from C-2 versus C-3 in dichloromethane.

	C2	C3	ΔG (kcal/mol)
**None**	-2019.349578	-2019.354624	3.16618
**CPCM**	-2019.414793	-2019.414375	-0.26278
**PCM**	-2019.413785	-2019.413526	-0.16289
**SMD**	-2019.440803	-2019.440247	-0.34886

Calculated energies and geometries.

Furanose

Energy	-764.459935

Zero-point correction	0.223555
Thermal correction to Energy	0.238894
Thermal correction to Enthalpy	0.239839
Thermal correction to Gibbs Free Energy	0.179673
Sum of electronic and zero-point Energies	-764.23638
Sum of electronic and thermal Energies	-764.22104
Sum of electronic and thermal Enthalpies	-764.220096
Sum of electronic and thermal Free Energies	-764.280261

Number of imaginary frequencies: 0

Geometry:

======
SM.log
======

C	-0.557441	-1.011538	-0.510038
C	-0.149436	-0.216596	0.745934
O	-1.263050	0.623078	1.077150
C	-2.369649	0.322557	0.212993
C	-2.084777	-1.061060	-0.371698
C	1.084771	0.645472	0.535767
O	2.134975	-0.278641	0.167480
H	-0.299061	-0.444108	-1.415955
H	0.028561	-0.920537	1.571019
H	-2.589929	-1.194999	-1.336845
H	1.347401	1.186060	1.451852
H	0.903002	1.363656	-0.272958
H	-3.272128	0.353227	0.838689
O	-2.466117	1.233291	-0.855422
C	-2.814336	2.554519	-0.444473
H	-2.034994	2.993062	0.191897
H	-2.922253	3.148194	-1.355410
H	-3.767956	2.554469	0.105776
C	3.410954	0.140165	-0.113591
C	3.706566	1.614565	0.041698
H	3.067388	2.215365	-0.614255
H	3.522383	1.945095	1.069675
H	4.752090	1.785327	-0.213359
O	4.215274	-0.686397	-0.461469
O	-0.030474	-2.328910	-0.569672
H	0.931395	-2.261000	-0.478817
O	-2.486002	-2.046094	0.568453
H	-1.862668	-2.782939	0.466984

[Fur-B]$^{-}$

Energy	-1690.367894
Zero-point correction	0.378158
Thermal correction to Energy	0.408552
Thermal correction to Enthalpy	0.409496
Thermal correction to Gibbs Free Energy	0.31413
Sum of electronic and zero-point Energies	-1689.989736
Sum of electronic and thermal Energies	-1689.959342
Sum of electronic and thermal Enthalpies	-1689.958398
Sum of electronic and thermal Free Energies	-1690.053764

Number of imaginary frequencies: 0

Geometry:

==============
BC6F5-N_SM.log
==============

C	-1.648651	-0.671221	0.264457
C	-2.072397	0.602422	-0.481968
O	-1.437960	1.691620	0.217327
C	-1.371960	1.361305	1.600347
C	-1.213433	-0.167591	1.673693
C	-3.585470	0.818050	-0.521945
O	-4.127106	-0.187504	-1.403441
O	-0.491174	-1.251665	-0.308906
O	0.140634	-0.541810	1.822392
H	-2.460637	-1.407386	0.289719
H	-1.677657	0.591340	-1.501633
H	-1.813648	-0.570654	2.496774
H	-3.793337	1.822818	-0.907678
H	-4.013814	0.726060	0.481223
H	-0.503400	1.896595	2.005610
O	-2.547019	1.714689	2.307096
C	-2.760538	3.123765	2.375735
H	-2.914567	3.555323	1.378414
H	-3.654537	3.277671	2.985353
H	-1.904172	3.625780	2.851673
C	-5.474320	-0.296412	-1.612635
C	-6.373022	0.708833	-0.922487
H	-6.281124	0.635671	0.166757
H	-6.108572	1.734083	-1.203024
H	-7.403283	0.505286	-1.213530

O	-5.876463	-1.177724	-2.331059
B	0.630649	-1.085076	0.577489
C	0.035543	-3.448888	1.536977
H	-0.295470	-2.931664	2.437527
H	-0.772409	-3.472399	0.805857
H	0.347111	-4.469397	1.783909
C	2.290724	-2.655661	1.942532
H	2.579466	-3.674224	2.224694
H	3.144721	-2.145076	1.496684
H	1.955881	-2.100336	2.818711
C	1.635394	-3.392143	-0.282670
H	2.501772	-2.867612	-0.686406
H	1.909333	-4.427168	-0.050216
H	0.826729	-3.369031	-1.013174
N	1.182332	-2.701082	0.953394
C	1.903942	-0.271408	-0.069509
C	2.765470	0.512801	0.705343
C	2.127798	-0.203492	-1.449218
C	3.763986	1.325041	0.165553
C	3.114075	0.594587	-2.031474
C	3.937507	1.368857	-1.216366
F	1.385075	-0.937733	-2.305828
F	3.283032	0.621705	-3.364057
F	4.891459	2.140201	-1.754788
F	4.561627	2.059545	0.959771
F	2.675125	0.512210	2.054467

quinuclidine•+

Energy	-328.948767
Zero-point correction	0.190863
Thermal correction to Energy	0.197729
Thermal correction to Enthalpy	0.198674
Thermal correction to Gibbs Free Energy	0.158914
Sum of electronic and zero-point Energies	-328.757904
Sum of electronic and thermal Energies	-328.751037
Sum of electronic and thermal Enthalpies	-328.750093
Sum of electronic and thermal Free Energies	-328.789853

Number of imaginary frequencies: 0

Geometry:

================
B97D3_Quin_rad.log
================

```
C   -0.825500   0.727705   1.200763
C    1.278302   0.001222  -0.005501
C    0.776047   0.752492   1.230398
H   -1.230691   1.739200   1.143990
H   -1.224337   0.209963   2.074476
H    1.107205   1.793764   1.231831
H    1.113533   0.278780   2.155384
C   -0.836046   0.671793  -1.225924
H   -1.243119   0.114941  -2.071343
H   -1.240059   1.685238  -1.211924
C    0.765234   0.693400  -1.271093
H    1.094119   0.175878  -2.175495
H    1.097014   1.733074  -1.325189
H    2.376792   0.002282  -0.010272
C    0.773039  -1.443521   0.030853
H    1.102897  -2.008572  -0.844248
H    1.109560  -1.966032   0.929533
C   -0.828382  -1.401978   0.035772
H   -1.234636  -1.898651  -0.846696
H   -1.228006  -1.857807   0.942974
N   -1.173777  -0.001247   0.005054
```

[Fur-B]⁻_PRC

Energy	-2019.350927
Zero-point correction	0.569929
Thermal correction to Energy	0.608893
Thermal correction to Enthalpy	0.609838
Thermal correction to Gibbs Free Energy	0.494295
Sum of electronic and zero-point Energies	-2018.780998
Sum of electronic and thermal Energies	-2018.742034
Sum of electronic and thermal Enthalpies	-2018.74109
Sum of electronic and thermal Free Energies	-2018.856632

Number of imaginary frequencies: 0

Geometry:

======================
BC6F5-N_C2_Hab-PRC.log
======================

C	-1.548793	-0.805437	0.148893
C	-2.989964	-1.248025	-0.164560
O	-3.117745	-1.221491	-1.610915
C	-2.262096	-0.221404	-2.136281
C	-1.046628	-0.224658	-1.196062
C	-4.066862	-0.368748	0.469209
O	-3.880220	-0.465203	1.887023
O	-0.674854	-1.875341	0.437223
O	-0.015938	-1.024108	-1.630772
H	-1.538123	-0.083288	0.973325
H	-3.132747	-2.284200	0.153984
H	-5.052696	-0.734742	0.161259
H	-3.954920	0.670101	0.135832
H	-2.017436	-0.523314	-3.163145
O	-2.814732	1.075817	-2.111215
C	-3.954042	1.225013	-2.973650
H	-4.776722	0.574852	-2.656665
H	-4.261509	2.270235	-2.904155
H	-3.684560	0.988917	-4.012414
C	-4.768129	0.109595	2.776670
C	-6.049865	0.676107	2.210668
H	-5.852432	1.441525	1.452208
H	-6.645080	-0.111939	1.735792
H	-6.622525	1.113822	3.027767
O	-4.472052	0.109353	3.940966

B	0.430206	-1.879869	-0.489889
C	1.656431	-3.509045	-2.124290
H	2.613514	-3.231102	-1.684058
H	1.711118	-4.533224	-2.502617
H	1.410576	-2.822462	-2.933723
C	-0.705094	-3.879799	-1.671583
H	-1.471886	-3.850059	-0.900076
H	-0.976097	-3.207520	-2.484460
H	-0.589526	-4.899070	-2.048778
C	0.946233	-4.344889	0.054500
H	0.990735	-5.372076	-0.316955
H	1.917938	-4.056493	0.455216
H	0.187185	-4.254119	0.830768
N	0.593408	-3.429052	-1.076715
C	1.853120	-1.337196	0.133292
C	2.789718	-0.628341	-0.627021
C	2.114183	-1.371435	1.509870
C	3.874187	0.055521	-0.077266
C	3.192157	-0.707904	2.103361
C	4.073446	0.021332	1.303565
F	2.664106	-0.547939	-1.974447
F	4.713038	0.754474	-0.856484
F	5.098525	0.675531	1.850724
F	3.386720	-0.761613	3.423779
F	1.312576	-2.059064	2.341185
H	-0.683387	0.854802	-1.058770
C	1.020358	2.571456	-1.703858
C	0.740550	2.120775	0.676794
C	-0.924274	3.425500	-0.519760
C	1.906660	3.813703	-1.337999
H	1.618885	1.668956	-1.831205
H	0.441755	2.739709	-2.614819
H	-0.012653	1.827751	1.411451
H	1.443277	1.298530	0.545940
C	1.468789	3.447228	1.094039
C	-0.150648	4.773310	-0.279039
H	-1.599288	3.193178	0.306990
H	-1.498422	3.438175	-1.447538
C	1.335891	4.449229	-0.061579
H	2.941760	3.496924	-1.180752
H	1.896473	4.523635	-2.169880
H	1.018935	3.847095	2.007340
H	2.519767	3.229922	1.305470
H	-0.571638	5.281695	0.593030
H	-0.277272	5.431651	-1.143521
H	1.885035	5.366247	0.175945

N	0.073533	2.358936	-0.605872

[Fur-B]⁻_TS (C2)

Energy	-2019.349578
Zero-point correction	0.567267
Thermal correction to Energy	0.605785
Thermal correction to Enthalpy	0.60673
Thermal correction to Gibbs Free Energy	0.493358
Sum of electronic and zero-point Energies	-2018.782311
Sum of electronic and thermal Energies	-2018.743793
Sum of electronic and thermal Enthalpies	-2018.742849
Sum of electronic and thermal Free Energies	-2018.85622

Number of imaginary frequencies: 1, -841.4819

Geometry:

====================
BC6F5-N_C2_Hab-TS.log
====================

```
C    -1.473034  -0.870533   0.139595
C    -2.886078  -1.380199  -0.205987
O    -3.023308  -1.261902  -1.649182
C    -2.229213  -0.177954  -2.110889
C    -1.024620  -0.187128  -1.167366
C    -4.011220  -0.613201   0.486046
O    -3.812419  -0.794020   1.892943
O    -0.522809  -1.890706   0.357587
O     0.066259  -0.837990  -1.641257
H    -1.508227  -0.203773   1.009128
H    -2.962274  -2.442651   0.040089
H    -4.973968  -1.016942   0.153064
H    -3.964551   0.450555   0.222328
H    -1.962756  -0.404406  -3.151742
O    -2.864247   1.074058  -2.014965
C    -3.999907   1.206498  -2.886144
H    -4.784124   0.489148  -2.621552
H    -4.371574   2.224861  -2.758211
H    -3.702549   1.051527  -3.932396
C    -4.729887  -0.335619   2.821770
C    -6.041450   0.199461   2.295461
H    -5.887643   1.025981   1.593104
H    -6.594848  -0.584218   1.765901
H    -6.634653   0.547138   3.140684
O    -4.431055  -0.405375   3.982688
```

B	0.576285	-1.764669	-0.567411
C	1.874308	-3.192784	-2.334881
H	2.817745	-2.909705	-1.869464
H	1.975014	-4.178304	-2.796937
H	1.597906	-2.453067	-3.085499
C	-0.466022	-3.712246	-1.923484
H	-1.237323	-3.771747	-1.158335
H	-0.762413	-2.998569	-2.690817
H	-0.303888	-4.696186	-2.370681
C	1.204846	-4.238469	-0.235116
H	1.299484	-5.227175	-0.691625
H	2.160944	-3.937773	0.192794
H	0.441771	-4.250390	0.542410
N	0.808393	-3.248823	-1.288169
C	1.975624	-1.209339	0.080264
C	2.870511	-0.400448	-0.629499
C	2.263002	-1.354163	1.444414
C	3.939817	0.272227	-0.039037
C	3.327556	-0.705446	2.076460
C	4.167301	0.122684	1.329835
F	2.712627	-0.206046	-1.962060
F	4.738106	1.065766	-0.766691
F	5.180153	0.760490	1.915206
F	3.549495	-0.868718	3.383027
F	1.501001	-2.141671	2.223083
H	-0.705947	1.029950	-0.889052
C	0.670498	2.681217	-1.656956
C	0.591905	2.084104	0.721183
C	-1.285934	3.254426	-0.306861
C	1.227024	4.105266	-1.374612
H	1.462426	1.936945	-1.746642
H	0.061589	2.645160	-2.563417
H	-0.115804	1.849625	1.519838
H	1.246424	1.224590	0.578393
C	1.387656	3.386606	1.012037
C	-0.654916	4.583707	0.203754
H	-1.986311	2.825070	0.413273
H	-1.806782	3.374201	-1.257152
C	0.874450	4.488655	0.071531
H	2.310583	4.111713	-1.523381
H	0.794030	4.827084	-2.073950
H	1.253883	3.672341	2.059211
H	2.457095	3.218672	0.850385
H	-0.933008	4.758236	1.247724
H	-1.042184	5.421240	-0.383693
H	1.333641	5.447111	0.328810

N	-0.196713	2.272000	-0.523296

[Fur-B]⁻_PostRC

Energy	-2019.359372
Zero-point correction	0.572847
Thermal correction to Energy	0.611811
Thermal correction to Enthalpy	0.612755
Thermal correction to Gibbs Free Energy	0.497351
Sum of electronic and zero-point Energies	-2018.786526
Sum of electronic and thermal Energies	-2018.747562
Sum of electronic and thermal Enthalpies	-2018.746618
Sum of electronic and thermal Free Energies	-2018.862021

Number of imaginary frequencies: 0

Geometry:

==========================
BC6F5-N_C2_Hab-PostRC.log
==========================

C	-1.487543	-0.942694	0.120711
C	-2.941746	-1.401989	-0.125181
O	-3.151503	-1.367824	-1.564325
C	-2.264523	-0.412101	-2.149746
C	-1.058706	-0.437798	-1.249878
C	-3.992474	-0.523455	0.551962
O	-3.706377	-0.544289	1.956875
O	-0.575134	-1.987200	0.414035
O	0.049102	-1.087060	-1.657251
H	-1.459908	-0.198106	0.927532
H	-3.057907	-2.440035	0.198355
H	-4.988506	-0.922300	0.331581
H	-3.928887	0.501010	0.163749
H	-2.070011	-0.751811	-3.175579
O	-2.788656	0.906041	-2.154767
C	-3.941477	1.057127	-2.998940
H	-4.771216	0.434877	-2.646289
H	-4.225480	2.110628	-2.954669
H	-3.699128	0.785478	-4.035752
C	-4.555599	0.035091	2.879533
C	-5.909088	0.493311	2.387133
H	-5.817789	1.221062	1.573381
H	-6.489400	-0.354526	2.006075
H	-6.443181	0.946674	3.221646
O	-4.171857	0.123997	4.014855

B	0.543304	-1.913860	-0.495725
C	1.910775	-3.455322	-2.096748
H	2.836935	-3.123905	-1.627861
H	2.036566	-4.471928	-2.478422
H	1.647321	-2.778807	-2.909148
C	-0.436853	-3.969236	-1.711317
H	-1.232581	-3.960298	-0.968808
H	-0.706778	-3.327163	-2.548686
H	-0.258512	-4.988544	-2.062968
C	1.191942	-4.341232	0.059903
H	1.315641	-5.361309	-0.313055
H	2.129443	-3.991666	0.492188
H	0.404448	-4.303658	0.811743
N	0.816005	-3.444250	-1.079340
C	1.911418	-1.266014	0.142491
C	2.796870	-0.480789	-0.604604
C	2.162862	-1.289698	1.521307
C	3.819452	0.281527	-0.040010
C	3.180942	-0.548712	2.129114
C	4.009604	0.252379	1.342050
F	2.678935	-0.402623	-1.952811
F	4.606984	1.051117	-0.806918
F	4.975417	0.981239	1.902271
F	3.367734	-0.596721	3.450687
F	1.409909	-2.043479	2.340316
H	-0.612685	1.359991	-0.935206
C	0.820387	2.608781	-1.801589
C	0.561930	2.146544	0.620809
C	-1.202163	3.367298	-0.600400
C	1.401209	4.017964	-1.563050
H	1.578806	1.826373	-1.779719
H	0.268768	2.522389	-2.739980
H	-0.211699	2.010705	1.379956
H	1.152418	1.232324	0.563784
C	1.416099	3.407209	0.861965
C	-0.526626	4.663594	-0.100128
H	-1.977910	2.999201	0.073840
H	-1.637225	3.464623	-1.596116
C	1.001906	4.486567	-0.153243
H	2.489567	3.987051	-1.663262
H	1.023783	4.720040	-2.312775
H	1.268133	3.760865	1.885855
H	2.479065	3.173572	0.747973
H	-0.841673	4.887183	0.923726
H	-0.836823	5.504443	-0.726583
H	1.496213	5.432239	0.081573

N -0.156478 2.289595 -0.697746

[Fur-B]•−

Energy	-1689.705243
Zero-point correction	0.365425
Thermal correction to Energy	0.395781
Thermal correction to Enthalpy	0.396725
Thermal correction to Gibbs Free Energy	0.301524
Sum of electronic and zero-point Energies	-1689.339818
Sum of electronic and thermal Energies	-1689.309462
Sum of electronic and thermal Enthalpies	-1689.308518
Sum of electronic and thermal Free Energies	-1689.40372

Number of imaginary frequencies: 0

Geometry:

==================
BC6F5-N_C2_Hab.log
==================

```
C   -1.651892  -0.015725   0.474551
C   -2.196776  -0.026215  -0.973393
O   -1.825217   1.240819  -1.568803
C   -1.672398   2.220989  -0.520536
C   -1.207690   1.420430   0.658547
C   -3.713771  -0.173419  -1.048082
O   -4.036096  -1.501550  -0.587490
O   -0.470317  -0.793434   0.655826
O    0.116033   1.456236   0.970911
H   -2.424078  -0.360276   1.175323
H   -1.710400  -0.820330  -1.547055
H   -4.039402  -0.035604  -2.085745
H   -4.191403   0.585258  -0.418773
H   -0.931459   2.945265  -0.884642
O   -2.892861   2.864311  -0.210748
C   -3.377885   3.686822  -1.272340
H   -3.620854   3.092064  -2.162436
H   -4.280338   4.178654  -0.900787
H   -2.634314   4.450865  -1.547457
C   -5.334219  -1.910337  -0.452847
C   -6.420459  -0.931428  -0.848137
H   -6.384417  -0.032749  -0.222452
H   -6.304666  -0.608512  -1.888304
H   -7.386119  -1.420653  -0.722654
O   -5.548257  -3.019597  -0.030472
```

B	0.616787	0.070132	1.024292
C	1.453978	-1.634638	2.864941
H	1.594964	-1.853693	3.928724
H	0.718680	-2.313065	2.432189
H	2.403026	-1.745820	2.339955
C	1.992182	0.714867	3.208796
H	2.163963	0.537872	4.275980
H	2.921951	0.564513	2.660020
H	1.637353	1.732552	3.046648
C	-0.286641	-0.068910	3.487212
H	-0.668515	0.940880	3.336129
H	-1.018246	-0.796280	3.136799
H	-0.078009	-0.235204	4.549191
N	0.968204	-0.237158	2.700892
C	1.994961	-0.146930	0.173541
C	2.895235	0.890734	-0.094534
C	2.303808	-1.369066	-0.436469
C	4.010915	0.747307	-0.920909
C	3.408573	-1.555297	-1.269041
C	4.267802	-0.486149	-1.516472
F	1.533682	-2.456885	-0.226950
F	3.657270	-2.750821	-1.827336
F	5.333971	-0.644737	-2.310828
F	4.840552	1.779737	-1.144248
F	2.726780	2.111729	0.459238

Quinuclidine-H$^+$

Energy	-329.60844
Zero-point correction	0.206633
Thermal correction to Energy	0.213426
Thermal correction to Enthalpy	0.21437
Thermal correction to Gibbs Free Energy	0.175883
Sum of electronic and zero-point Energies	-329.401807
Sum of electronic and thermal Energies	-329.395014
Sum of electronic and thermal Enthalpies	-329.39407
Sum of electronic and thermal Free Energies	-329.432556

Number of imaginary frequencies: 0

Geometry:

===============
B97D3_QuinH.log
===============

```
C     0.745507  -1.439226   0.017604
C    -1.309803   0.000085  -0.000170
C    -0.787020  -1.434282   0.192033
H     1.065123  -1.881064  -0.928925
H     1.269639  -1.942150   0.832959
H    -1.237739  -2.109734  -0.539476
H    -1.056727  -1.803006   1.185797
C     0.745803   0.704447  -1.255009
H     1.065279   1.745114  -1.164235
H     1.270191   0.249908  -2.098130
C    -0.786640   0.550738  -1.338302
H    -1.237428   1.521871  -1.557811
H    -1.056027  -0.125667  -2.154488
H    -2.401926   0.000133  -0.000385
C    -0.787032   0.883640   1.145907
H    -1.056371   1.928676   0.968075
H    -1.238007   0.588202   2.096603
C     0.745401   0.734675   1.237755
H     1.269813   1.692084   1.265950
H     1.064513   0.135566   2.093622
H     2.253912  -0.000082   0.000347
N     1.232637  -0.000045   0.000170
```

[Fur-B]⁻_TS (C3)

Energy	-2019.354624
Zero-point correction	0.567716
Thermal correction to Energy	0.606065
Thermal correction to Enthalpy	0.607009
Thermal correction to Gibbs Free Energy	0.495309
Sum of electronic and zero-point Energies	-2018.786908
Sum of electronic and thermal Energies	-2018.748559
Sum of electronic and thermal Enthalpies	-2018.747615
Sum of electronic and thermal Free Energies	-2018.859315

Number of imaginary frequencies: 1, -1189.5705

Geometry:

=====================
BC6F5-N_C3_Hab-TS.log
=====================

```
C     1.156775  -0.877497  -0.159521
C     2.501033  -1.455397  -0.632794
O     2.550080  -2.739414   0.011672
C     1.911133  -2.642995   1.302143
C     3.757098  -0.648870  -0.317420
O     3.644865   0.570480  -1.073191
O     0.142366  -1.045346  -1.051167
O    -0.485801  -2.189979   0.882661
H     2.454306  -1.622596  -1.713444
H     4.630453  -1.234353  -0.620248
H     3.828322  -0.425758   0.752602
H     1.547748  -3.653411   1.529318
O     2.770906  -2.169193   2.298075
C     3.825018  -3.084102   2.637247
H     4.488446  -3.257746   1.782004
H     4.383199  -2.622673   3.453681
H     3.406952  -4.042452   2.974177
C     4.697909   1.445339  -1.227374
C     6.077116   0.953003  -0.873519
H     6.133539   0.662086   0.181450
H     6.341726   0.074423  -1.472096
H     6.791608   1.752342  -1.067461
O     4.439080   2.547396  -1.642992
B    -1.020539  -1.703732  -0.369388
C    -2.516339  -3.794225  -0.587835
```

H	-3.402091	-3.163755	-0.513286
H	-2.753196	-4.696698	-1.157502
H	-2.167135	-4.056604	0.410283
C	-0.252920	-3.938175	-1.463144
H	0.533856	-3.400123	-1.988947
H	0.103184	-4.239618	-0.480625
H	-0.553123	-4.816623	-2.040132
C	-1.924935	-2.637926	-2.651103
H	-2.156126	-3.537736	-3.227301
H	-2.824323	-2.032985	-2.543481
H	-1.150253	-2.059687	-3.153518
N	-1.435569	-3.036815	-1.296010
C	-2.303376	-0.693520	-0.241912
C	-3.130405	-0.688745	0.888635
C	-2.545727	0.339341	-1.155456
C	-4.096648	0.291811	1.128189
C	-3.495296	1.341269	-0.955515
C	-4.274475	1.320908	0.202307
F	-3.021197	-1.653889	1.820518
F	-4.850047	0.254873	2.230954
F	-5.183245	2.271363	0.417065
F	-3.655522	2.323825	-1.851316
F	-1.819995	0.432187	-2.295538
H	1.217058	0.411953	0.122414
C	-0.278958	1.815310	1.170115
C	0.866461	2.399040	-0.922493
C	2.099344	2.328221	1.197485
C	-0.552607	3.297852	1.547003
H	-1.062976	1.405235	0.536481
H	-0.182360	1.179588	2.052984
H	1.852292	2.368896	-1.387012
H	0.174767	1.804939	-1.521805
C	0.365996	3.853019	-0.706316
C	1.867033	3.864013	1.295774
H	3.034301	2.099456	0.692557
H	2.092049	1.844529	2.177877
C	0.450031	4.183170	0.792236
H	-1.580551	3.557577	1.277137
H	-0.449501	3.440303	2.627225
H	0.987648	4.537302	-1.290459
H	-0.665054	3.959153	-1.058520
H	2.610805	4.389215	0.689398
H	1.993009	4.187418	2.333227
H	0.220683	5.239139	0.960378
N	0.993088	1.734403	0.405355
C	0.774115	-1.617681	1.139462

H	0.734641	-0.980952	2.031411

Quinuclidine

Energy	-329.214511
Zero-point correction	0.190858
Thermal correction to Energy	0.197571
Thermal correction to Enthalpy	0.198515
Thermal correction to Gibbs Free Energy	0.15998
Sum of electronic and zero-point Energies	-329.023652
Sum of electronic and thermal Energies	-329.016939
Sum of electronic and thermal Enthalpies	-329.015995
Sum of electronic and thermal Free Energies	-329.054531

Number of imaginary frequencies: 0

Geometry:

==============
B97D3_Quin.log
==============

C	-0.798858	-1.364751	0.231737
C	1.292054	-0.001525	-0.000961
C	0.760168	-1.425280	0.240855
H	-1.210616	-1.712260	1.186344
H	-1.211996	-2.006955	-0.554688
H	1.132723	-1.804929	1.200213
H	1.131454	-2.100108	-0.540187
C	-0.795634	0.883906	1.067722
H	-1.206806	1.884788	0.892325
H	-1.207070	0.524389	2.018018
C	0.763659	0.920481	1.112233
H	1.136971	1.940728	0.960397
H	1.136766	0.581233	2.086399
H	2.387466	-0.002813	-0.001812
C	0.761183	0.502348	-1.354846
H	1.134763	1.515336	-1.548711
H	1.132199	-0.139416	-2.163424
C	-0.797972	0.483486	-1.297660
H	-1.208819	1.486623	-1.460450
H	-1.211406	-0.168423	-2.075919
N	-1.291890	0.001402	0.001002

[Fur-B]•

Energy	-1690.064941
Zero-point correction	0.377832
Thermal correction to Energy	0.40846
Thermal correction to Enthalpy	0.409404
Thermal correction to Gibbs Free Energy	0.314184
Sum of electronic and zero-point Energies	-1689.687109
Sum of electronic and thermal Energies	-1689.656481
Sum of electronic and thermal Enthalpies	-1689.655537
Sum of electronic and thermal Free Energies	-1689.750757

Number of imaginary frequencies: 0

Geometry:

======================
BC6F5-N_C2_Hab-prot.log
======================

```
C    2.094047  -0.237080   0.465485
C    2.142364   0.384213  -0.944690
O    2.904227  -0.550456  -1.679447
C    2.552024  -1.924526  -1.202459
C    1.866741  -1.671042   0.118207
C    2.730326   1.783347  -0.948911
O    1.893118   2.447109   0.029934
O    0.549455  -1.992052   0.274718
H    2.970299  -0.002507   1.074155
H    1.103491   0.443159  -1.319026
H    2.651084   2.258384  -1.931083
H    3.776700   1.771340  -0.623924
H    1.873652  -2.365483  -1.950651
O    3.695659  -2.663423  -1.030814
C    4.328555  -3.070022  -2.260906
H    4.736334  -2.203489  -2.791870
H    5.135447  -3.746253  -1.976204
H    3.611007  -3.597838  -2.903581
C    2.045405   3.779951   0.407096
C    3.028032   4.600748  -0.380591
H    4.036454   4.177891  -0.314709
H    2.749361   4.627418  -1.439779
H    3.033916   5.614413   0.018778
O    1.368596   4.155622   1.323376
B   -0.215802  -0.989886   0.977633
```

C	-1.257174	-0.489225	3.308868
H	-1.503018	-0.911741	4.285512
H	-0.578246	0.354117	3.435192
H	-2.168645	-0.162479	2.809435
C	-1.536840	-2.706669	2.323033
H	-1.754105	-3.113904	3.312751
H	-2.457523	-2.356131	1.858019
H	-1.067926	-3.464192	1.696166
C	0.636371	-2.028463	3.196902
H	1.122682	-2.797152	2.597741
H	1.312892	-1.188912	3.354180
H	0.336879	-2.441422	4.162437
N	-0.592093	-1.545634	2.476801
H	0.673928	1.080235	1.070888
O	0.893716	0.133732	1.238792
C	-1.475486	-0.394856	0.177700
C	-2.228600	-1.188648	-0.699605
C	-1.857539	0.949073	0.241948
C	-3.283455	-0.685005	-1.462546
C	-2.897361	1.498680	-0.502789
C	-3.618719	0.668099	-1.365264
F	-1.958978	-2.500284	-0.826297
F	-3.971633	-1.481783	-2.278661
F	-4.617265	1.162890	-2.086342
F	-3.205793	2.790856	-0.401794
F	-1.182032	1.799178	1.067365

[Fur-B]•_TS

Energy	-1690.060338
Zero-point correction	0.376233
Thermal correction to Energy	0.406641
Thermal correction to Enthalpy	0.407585
Thermal correction to Gibbs Free Energy	0.312614
Sum of electronic and zero-point Energies	-1689.684105
Sum of electronic and thermal Energies	-1689.653697
Sum of electronic and thermal Enthalpies	-1689.652753
Sum of electronic and thermal Free Energies	-1689.747724

Number of imaginary frequencies: 1, -248.0240

Geometry:

======================
BC6F5-N_enolate-TS.log
======================

C	-1.780069	-0.347437	0.212364
C	-2.297416	0.691952	-0.763192
O	-1.633717	1.916671	-0.413116
C	-1.264906	1.883798	0.990868
C	-1.076555	0.409203	1.229407
C	-3.826963	0.819624	-0.674529
O	-4.329694	-0.458524	-1.069611
O	0.042258	-0.140831	1.708385
H	-2.440684	-1.176034	0.452131
H	-2.031782	0.444605	-1.797897
H	-4.145527	1.619519	-1.352319
H	-4.125983	1.074818	0.348640
H	-0.337292	2.466768	1.075062
O	-2.261185	2.369483	1.831386
C	-2.411934	3.802446	1.787767
H	-2.743338	4.130269	0.796132
H	-3.166793	4.051276	2.534663
H	-1.464184	4.295046	2.042771
C	-5.694284	-0.717094	-1.176742
C	-6.629680	0.449294	-0.983507
H	-6.499824	0.904603	0.004422
H	-6.443446	1.226091	-1.733595
H	-7.653648	0.090988	-1.084377
O	-6.019386	-1.844151	-1.423554
B	0.672152	-1.013700	0.695888

C	1.552112	-3.421859	0.402276
H	1.756540	-4.371403	0.901653
H	0.828412	-3.575160	-0.398320
H	2.476800	-3.011542	-0.003200
C	1.981841	-2.275041	2.514983
H	2.139509	-3.239049	3.003402
H	2.921250	-1.917186	2.096396
H	1.591424	-1.549420	3.227098
C	-0.269204	-3.045411	1.985159
H	-0.682647	-2.347609	2.712570
H	-0.983559	-3.230068	1.183357
H	-0.020787	-3.988782	2.475699
N	0.986131	-2.457254	1.402972
H	-0.205201	-1.101227	-1.241021
O	-0.462334	-1.284996	-0.319165
C	1.960212	-0.342869	-0.012785
C	2.849449	0.481593	0.693055
C	2.237669	-0.466783	-1.377822
C	3.934829	1.123314	0.093206
C	3.302924	0.152114	-2.024339
C	4.164882	0.957392	-1.274637
F	2.686756	0.683704	2.013575
F	4.749993	1.891513	0.813825
F	5.190378	1.562293	-1.859923
F	3.501061	-0.009260	-3.331710
F	1.414266	-1.229789	-2.167689

[“enolate”-B]•

Energy	-1690.100651
Zero-point correction	0.373969
Thermal correction to Energy	0.405772
Thermal correction to Enthalpy	0.406717
Thermal correction to Gibbs Free Energy	0.307656
Sum of electronic and zero-point Energies	-1689.726682
Sum of electronic and thermal Energies	-1689.694879
Sum of electronic and thermal Enthalpies	-1689.693935
Sum of electronic and thermal Free Energies	-1689.792995

Number of imaginary frequencies: 0

Geometry:

```
===================
BC6F5-N_enolate.log
===================

 C     1.415713  -0.210512   0.318678
 C     2.590374  -0.432646   1.177622
 O     2.803565   0.763793   1.924612
 C     1.965931   1.812863   1.420261
 C     1.008682   1.108531   0.471070
 C     3.884389  -0.762610   0.364970
 O     3.617630  -1.987476  -0.306659
 O     0.029468   1.753481  -0.047697
 H     1.018045  -0.940361  -0.370577
 H     2.434465  -1.268785   1.878160
 H     4.707765  -0.847671   1.082209
 H     4.081323   0.055047  -0.337160
 H     1.452602   2.257417   2.289044
 O     2.658604   2.773527   0.691333
 C     3.459708   3.654575   1.504509
 H     4.263249   3.101228   2.002166
 H     3.883787   4.391659   0.821357
 H     2.834170   4.156414   2.254935
 C     4.590357  -2.641514  -1.043796
 C     5.975173  -2.049814  -1.050255
 H     5.955755  -1.017955  -1.417868
 H     6.388584  -2.039662  -0.035287
 H     6.613888  -2.654412  -1.693290
 O     4.264797  -3.642174  -1.624372
 B    -0.960935   1.082436  -1.019948
```

C	-2.926053	1.963016	-2.446209
H	-3.522187	2.825130	-2.755095
H	-2.415923	1.539094	-3.310523
H	-3.575547	1.214511	-1.991599
C	-2.614986	3.011709	-0.275788
H	-3.185246	3.880854	-0.613727
H	-3.294897	2.275123	0.152520
H	-1.883074	3.315775	0.470821
C	-1.035460	3.449605	-2.083117
H	-0.326830	3.822651	-1.344484
H	-0.497230	2.993913	-2.913113
H	-1.666842	4.266818	-2.440675
N	-1.905927	2.405053	-1.444187
H	-0.586179	-0.113731	-2.593298
O	-0.221504	0.656399	-2.146031
C	-1.845826	-0.050663	-0.234445
C	-2.386565	-1.148939	-0.908875
C	-2.057147	-0.060927	1.153823
C	-3.069651	-2.193432	-0.281881
C	-2.721561	-1.088961	1.831013
C	-3.234674	-2.164411	1.107280
F	-2.248313	-1.256437	-2.256061
F	-3.546448	-3.216214	-0.985745
F	-3.876020	-3.148264	1.725090
F	-2.885550	-1.034850	3.152624
F	-1.637495	0.969516	1.919191

Furanose_Ox

Energy	-688.037584
Zero-point correction	0.195521
Thermal correction to Energy	0.209425
Thermal correction to Enthalpy	0.21037
Thermal correction to Gibbs Free Energy	0.152734
Sum of electronic and zero-point Energies	-687.842063
Sum of electronic and thermal Energies	-687.828159
Sum of electronic and thermal Enthalpies	-687.827215
Sum of electronic and thermal Free Energies	-687.88485

Number of imaginary frequencies: 0

Geometry:

=====
P.log
=====

C	-0.198665	-0.689874	1.047669
O	-1.387024	0.079235	1.389411
C	-2.260521	0.116432	0.278408
C	-1.990460	-1.205253	-0.479585
C	0.875023	0.252579	0.507147
O	2.074718	-0.531703	0.368498
O	-2.718969	-1.707664	-1.295600
H	0.155152	-1.132315	1.982670
H	1.028575	1.072174	1.219321
H	0.560975	0.668248	-0.456143
H	-3.284900	0.187165	0.670059
O	-1.994031	1.161625	-0.627727
C	-2.325512	2.455343	-0.113777
H	-1.717608	2.701948	0.766080
H	-2.121867	3.169378	-0.914982
H	-3.390483	2.503842	0.157318
C	3.212801	-0.003024	-0.185998
C	3.159023	1.437059	-0.649349
H	2.421370	1.562354	-1.449780
H	2.872307	2.108202	0.167315
H	4.144900	1.714916	-1.021225
O	4.185195	-0.708199	-0.276484
C	-0.662352	-1.743401	0.042238
H	0.036908	-1.907006	-0.782644
H	-0.840465	-2.714428	0.516717

[Fur-B]

Energy	-76.423158
Zero-point correction	0.020871
Thermal correction to Energy	0.023706
Thermal correction to Enthalpy	0.02465
Thermal correction to Gibbs Free Energy	0.003216
Sum of electronic and zero-point Energies	-76.402287
Sum of electronic and thermal Energies	-76.399451
Sum of electronic and thermal Enthalpies	-76.398507
Sum of electronic and thermal Free Energies	-76.419942

Number of imaginary frequencies: 0

Geometry:

```
=======
H2O.log
=======

 O     -0.000000    0.000000    0.118205
 H     -0.000000    0.763541   -0.472821
 H     -0.000000   -0.763541   -0.472821
```

References

[1] S. Manhas and M. S. Taylor, *J. Org. Chem.* 2017, **82**, 11406–11417.
[2] R. S. Mancini, J. B. Lee and M. S. Taylor, *Org. Biomol. Chem.* 2017, **15**, 132–143.
[3] K. P. Kaliappan and A. V. Subrahmanyam, *Org. Lett.* 2007, **9**,1121–1124.
[4] L. Lerner and G. Mennitt, *Carbohydr. Res.* 1994, **259**, 191–200.
[5] V. Dimakos, H. Y. Su, G. E. Garrett and M. S. Taylor, *J. Am. Chem. Soc.* 2019, **141**, 5149–5153.
[6] A. B. Lopes, P. Wagner, R. O. M. A. de Souza, N. L. Germain, J. Uziel, J-J. Bourguignon, M. Schmitt and L. S. Miranda, *J. Org. Chem.* 2016, **81**, 14540–4549.
[7] A. Földesi, M. K. Kundu, Z. Dinya and J. Chattopadhyaya, *Helv. Chim. Acta.* 2004, **87**, 742–757.
[8] M. Donnard, T. Tschamber, D. L. Nouën, S. Desrat, K. Hinsinger and J. Eustache, *Tetrahedron*, 2011, **67**, 339–357.
[9] M. Çayir, S. Demirci, S. Sezer and C. Tanyeli, *Tetrahedron: Asymmetry*, 2011, **22**, 1161–1168
[10] R. E. Harry-O'Kuru, J. M.Smith and M. S. Wolfe, *J. Org. Chem.* 1997, **62**, 1754–1759.
[11] M. Prévost, O. St-Jean and Y. Guindon, *J. Am. Chem. Soc.* 2010, **132**, 12433–12439.
[12] M. A. Cismesia and T. P. Yoon, *Chem. Sci.*, 2015, **6**, 5426– 5434.
[13] C. G. Hathchard and C. A. Parker, *Proc. Roy. Soc. A.* 1956, **235**, 518-536.
[14]Gaussian 16, Revision B.01, M. J. Frisch, G. W. Trucks, H. B. Schlegel, G. E. Scuseria, M. A. Robb, J. R. Cheeseman, G. Scalmani, V. Barone, G. A. Petersson, H. Nakatsuji, X. Li, M. Caricato, A. V. Marenich, J. Bloino, B. G. Janesko, R. Gomperts, B. Mennucci, H. P. Hratchian, J. V. Ortiz, A. F. Izmaylov, J. L. Sonnenberg, D. Williams-Young, F. Ding, F. Lipparini, F. Egidi, J. Goings, B. Peng, A. Petrone, T. Henderson, D. Ranasinghe, V. G. Zakrzewski, J. Gao, N. Rega, G. Zheng, W. Liang, M. Hada, M. Ehara, K. Toyota, R. Fukuda, J. Hasegawa, M. Ishida, T. Nakajima, Y. Honda, O. Kitao, H. Nakai, T. Vreven, K. Throssell, J. A. Montgomery Jr., J. E. Peralta, F. Ogliaro, M. J. Bearpark, J. J. Heyd, E. N. Brothers, K. N. Kudin, V. N. Staroverov, T. A. Keith, R. Kobayashi, J. Normand, K. Raghavachari, A. P. Rendell, J. C. Burant, S. S. Iyengar, J. Tomasi, M. Cossi, J. M. Millam, M. Klene, C. Adamo, R. Cammi, J. W. Ochterski, R. L. Martin, K. Morokuma, O. Farkas, J. B. Foresman, D. J. Fox, Gaussian, Inc., Wallingford CT, 2016.
[15]S. Grimme, S. Ehrlich, L. Goerigk, *J. Comput. Chem.* 2011, **32**, 1456–1465.
[16]F. Weigend, R. Ahlrichs, *Phys. Chem. Chem. Phys.* 2005, **7**, 3297–3305.

^{1}H, ^{13}C and 2D NMR Spectra

This page intentionally left blank

(2-D)-1a –^{1}H NMR (500 MHz, $CDCl_3$)

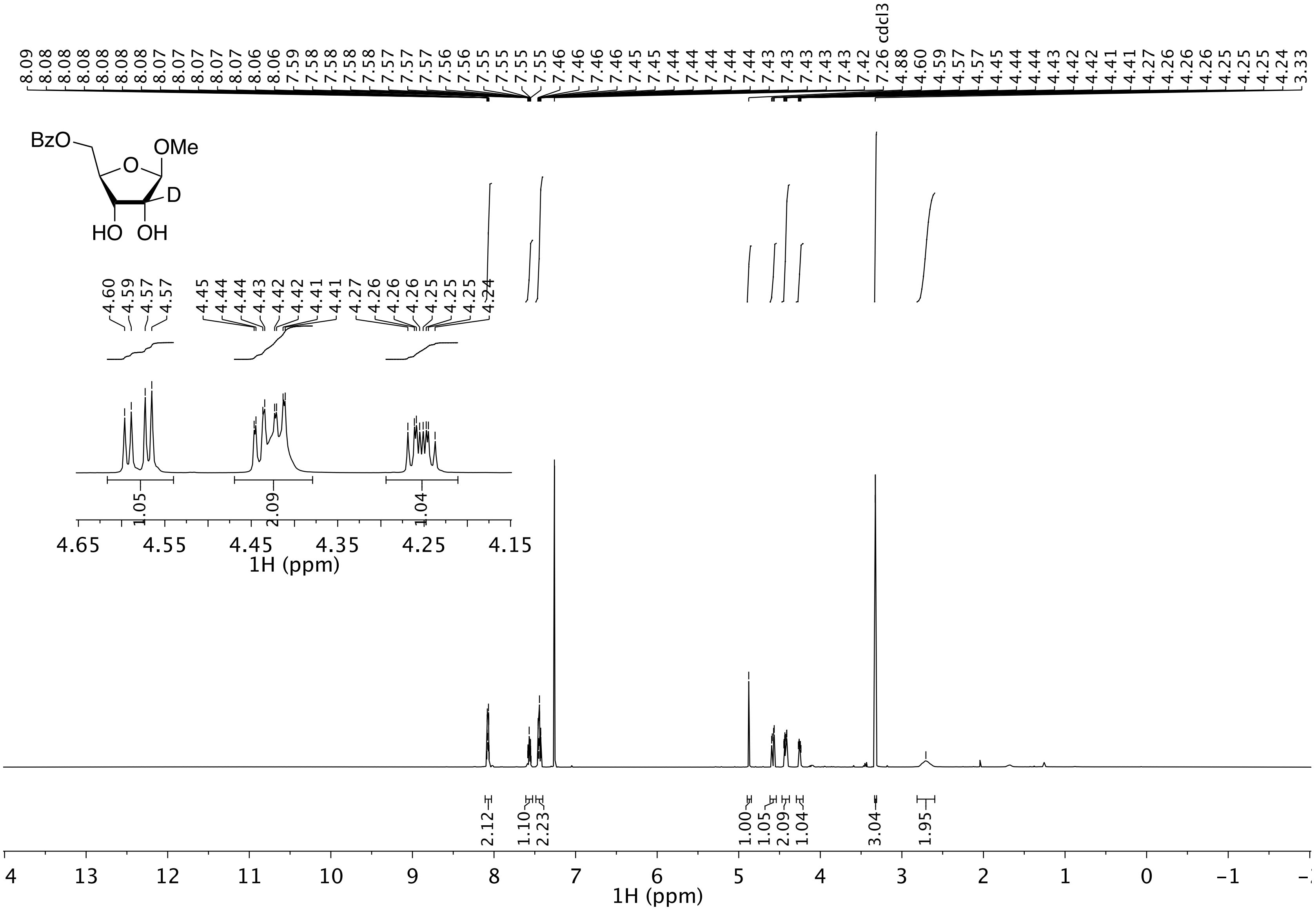

(2-D)-1a –^{13}C NMR (126 MHz, $CDCl_3$)

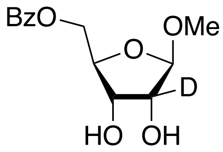

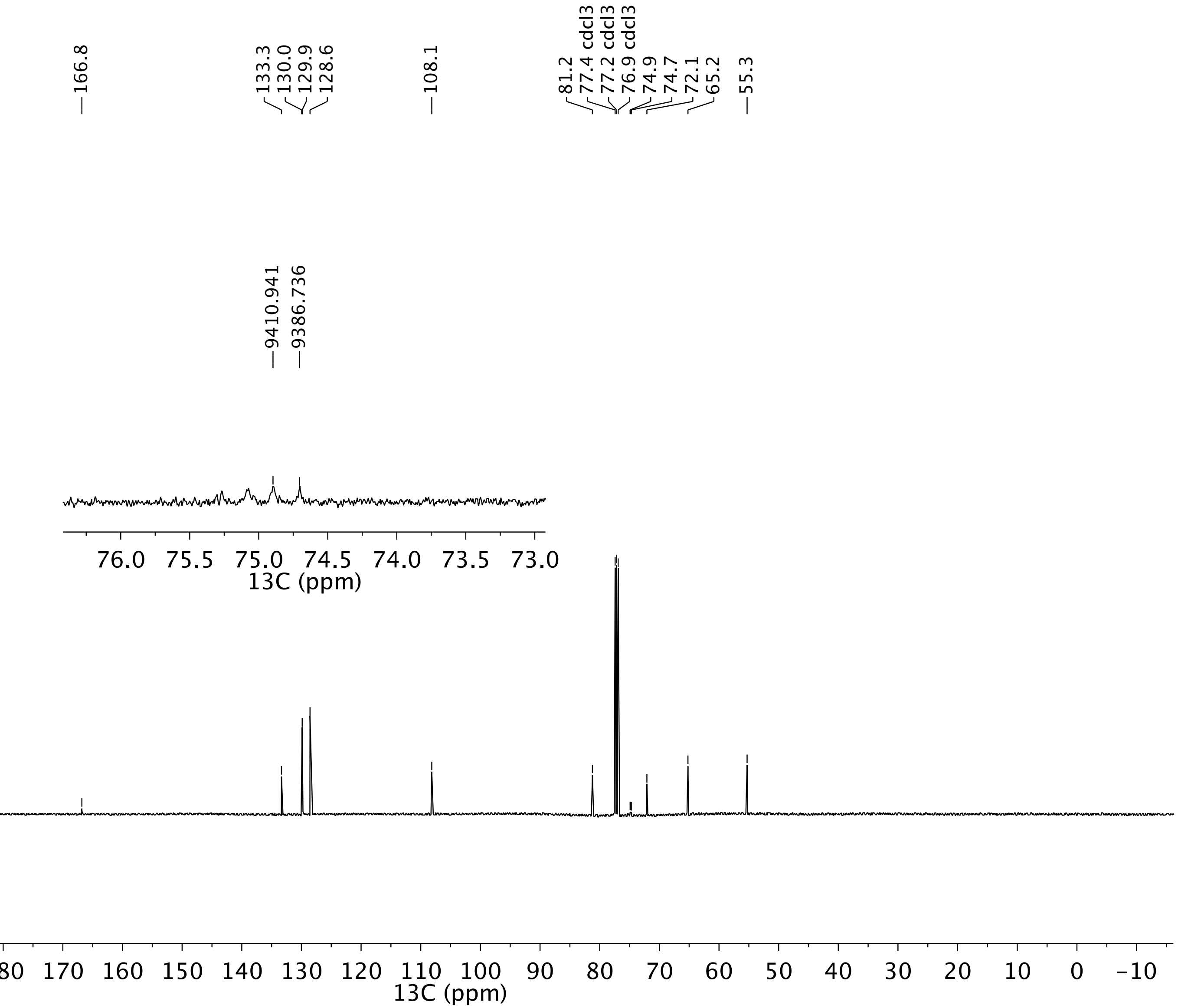

(2-D)-1a –COSY (400 MHz, CDCl$_3$)

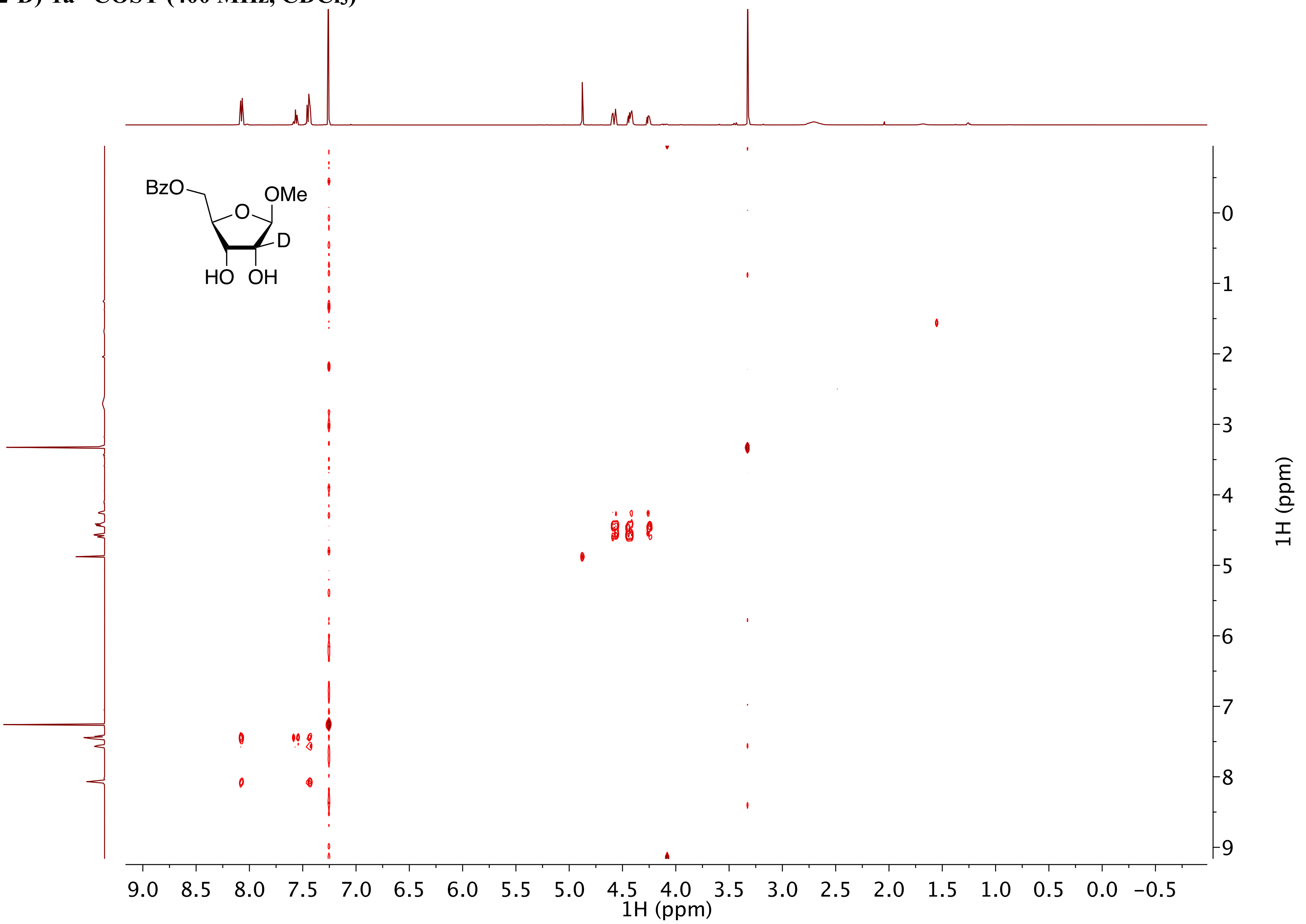

1b –¹H NMR (500 MHz, CDCl₃)

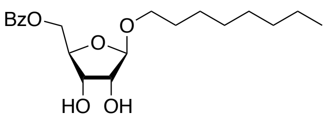

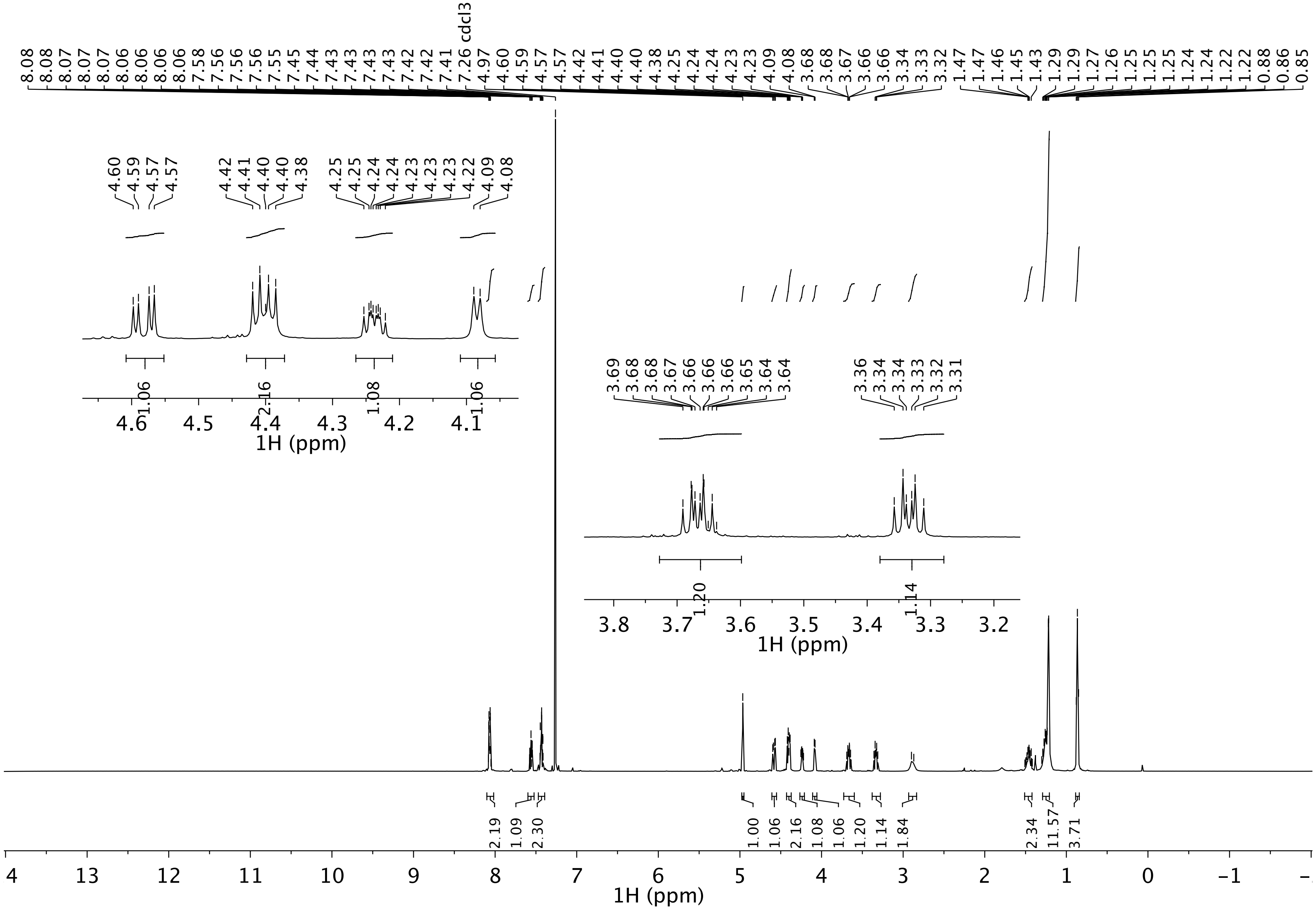

1b –^{1}H NMR (126 MHz, $CDCl_3$)

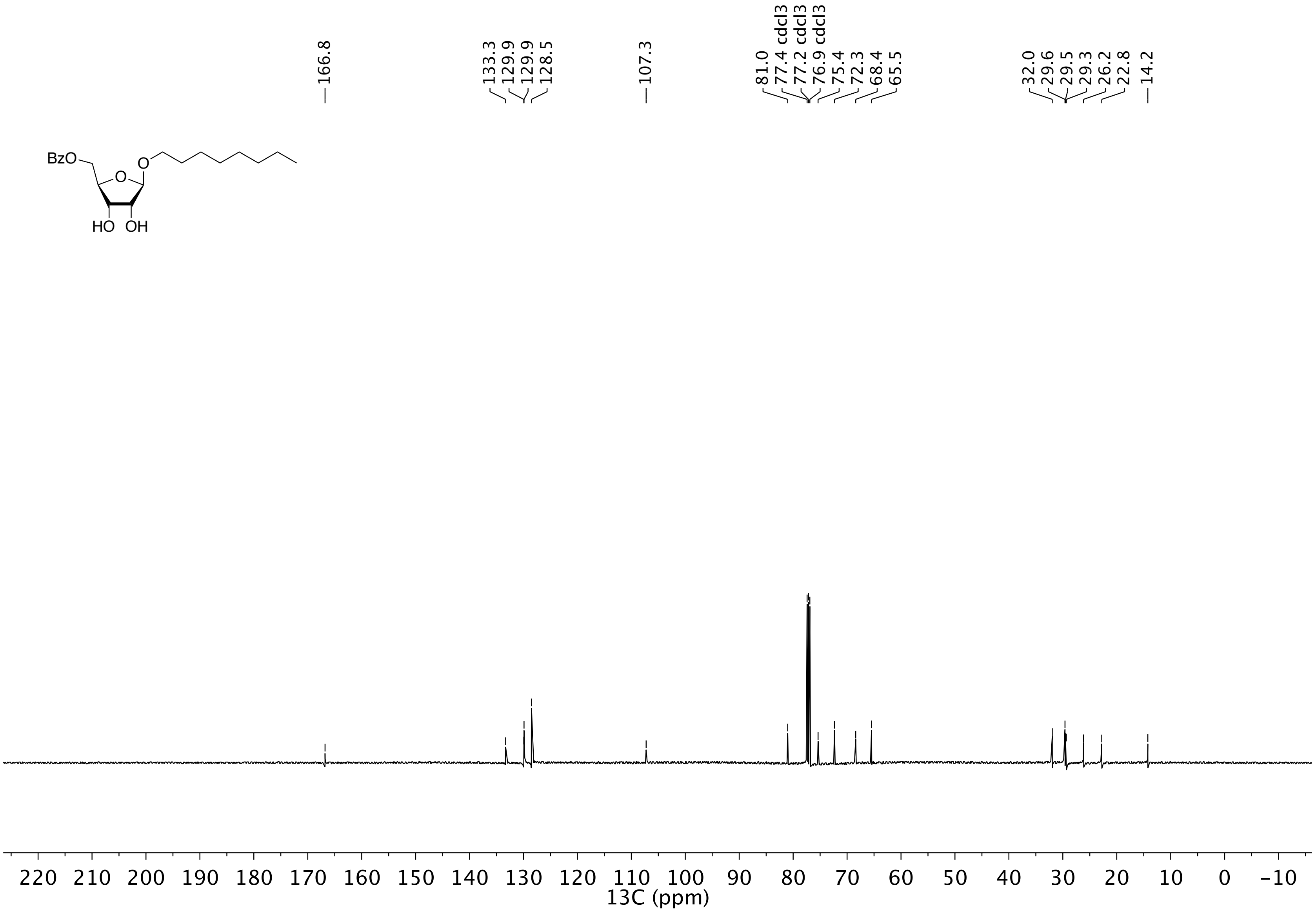

1b –COSY (500 MHz, CDCl$_3$

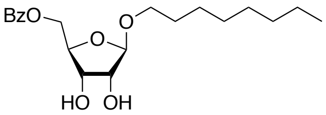

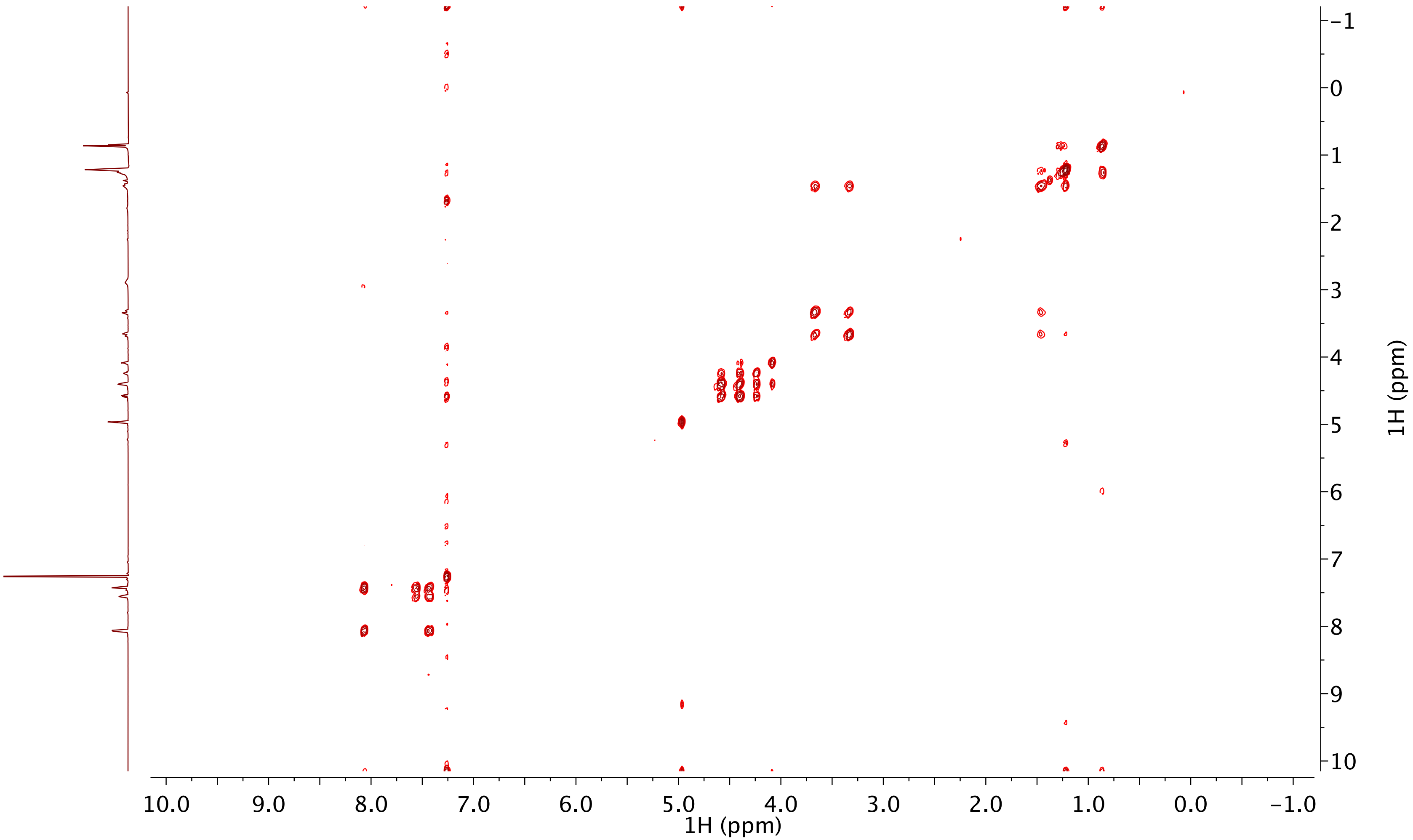

1c –^{1}H NMR (400 MHz, CDCl$_3$)

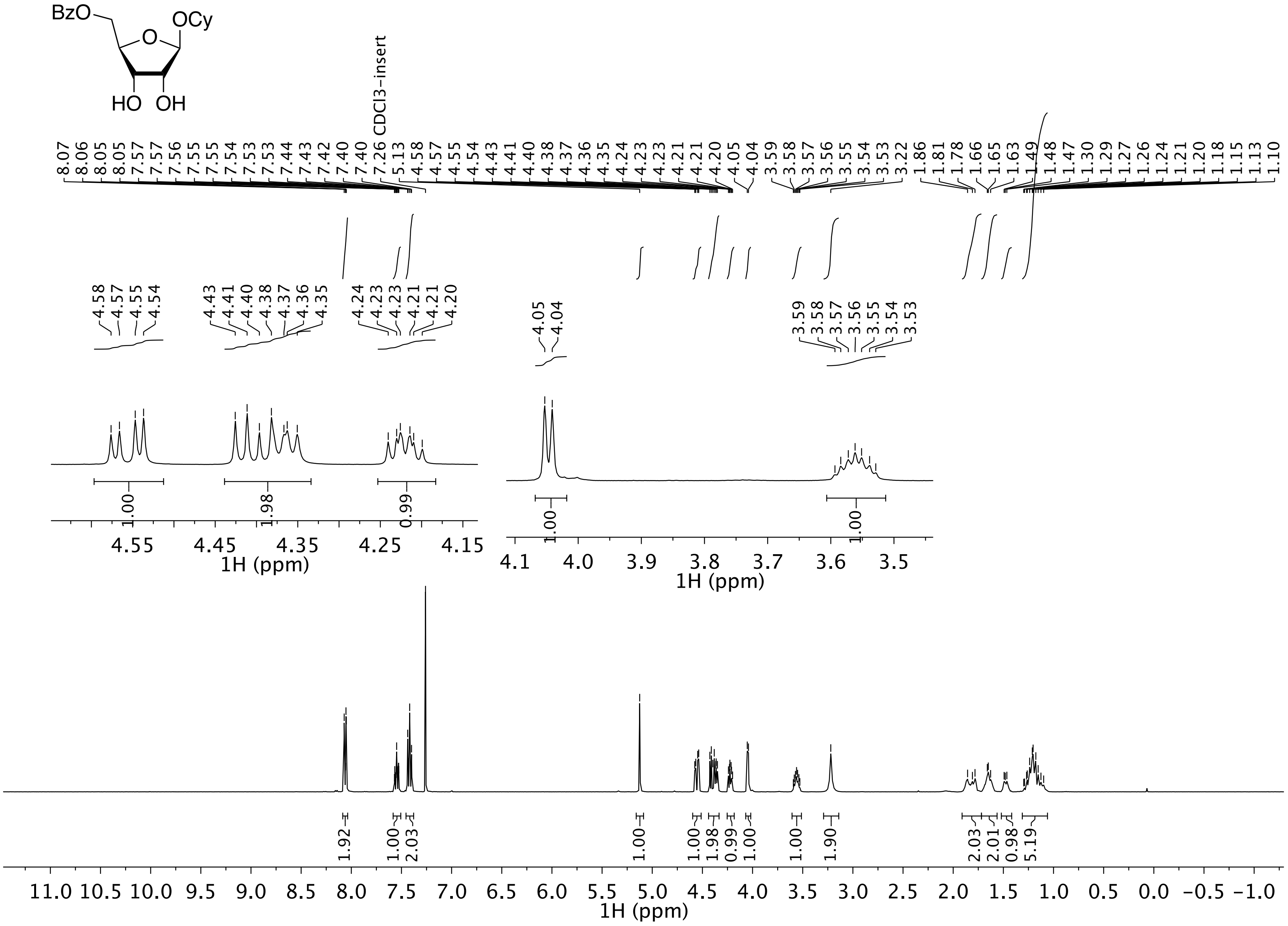

1c –^{13}C NMR (101 MHz, $CDCl_3$)

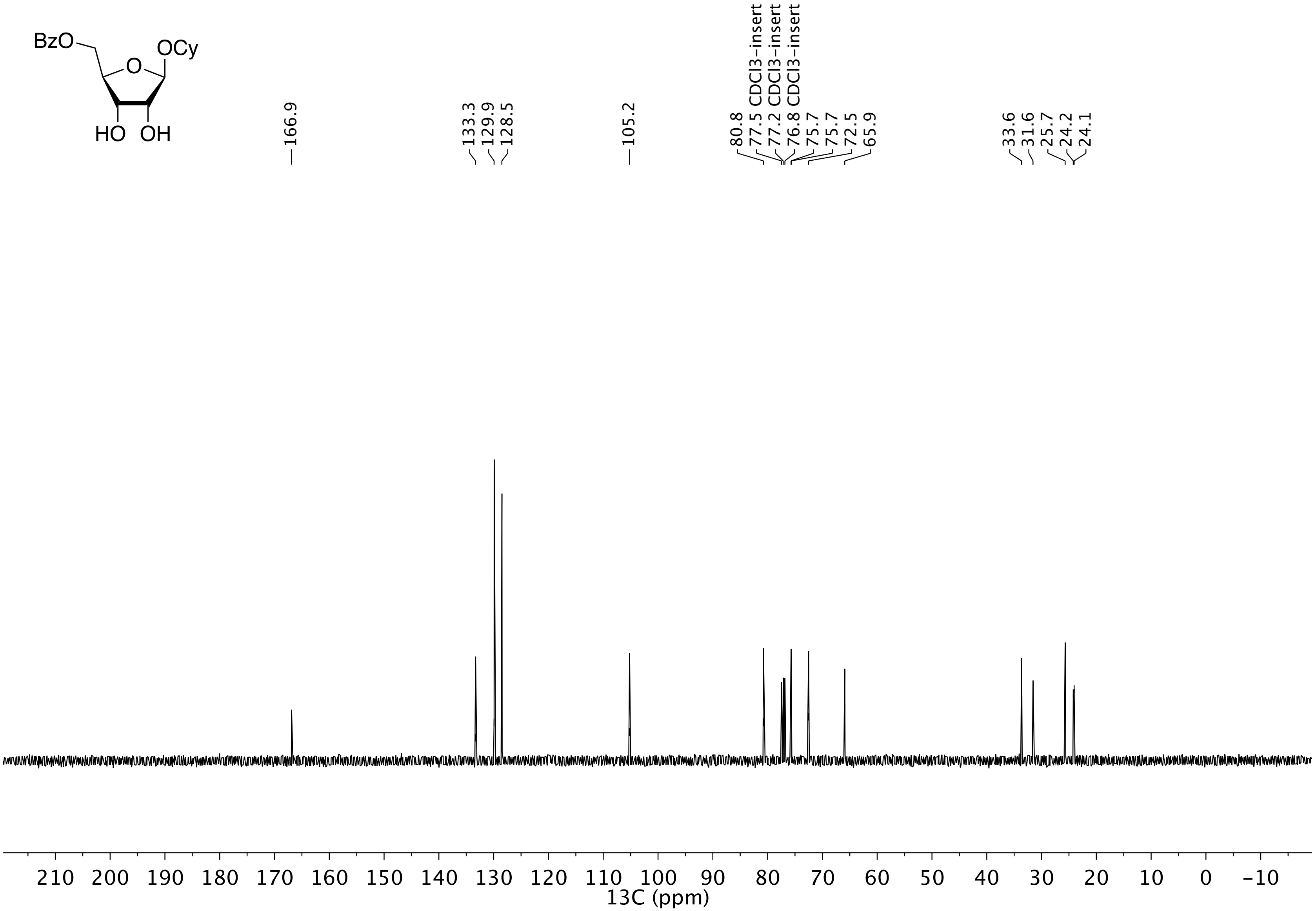

1c – COSY (400 MHz, $CDCl_3$)

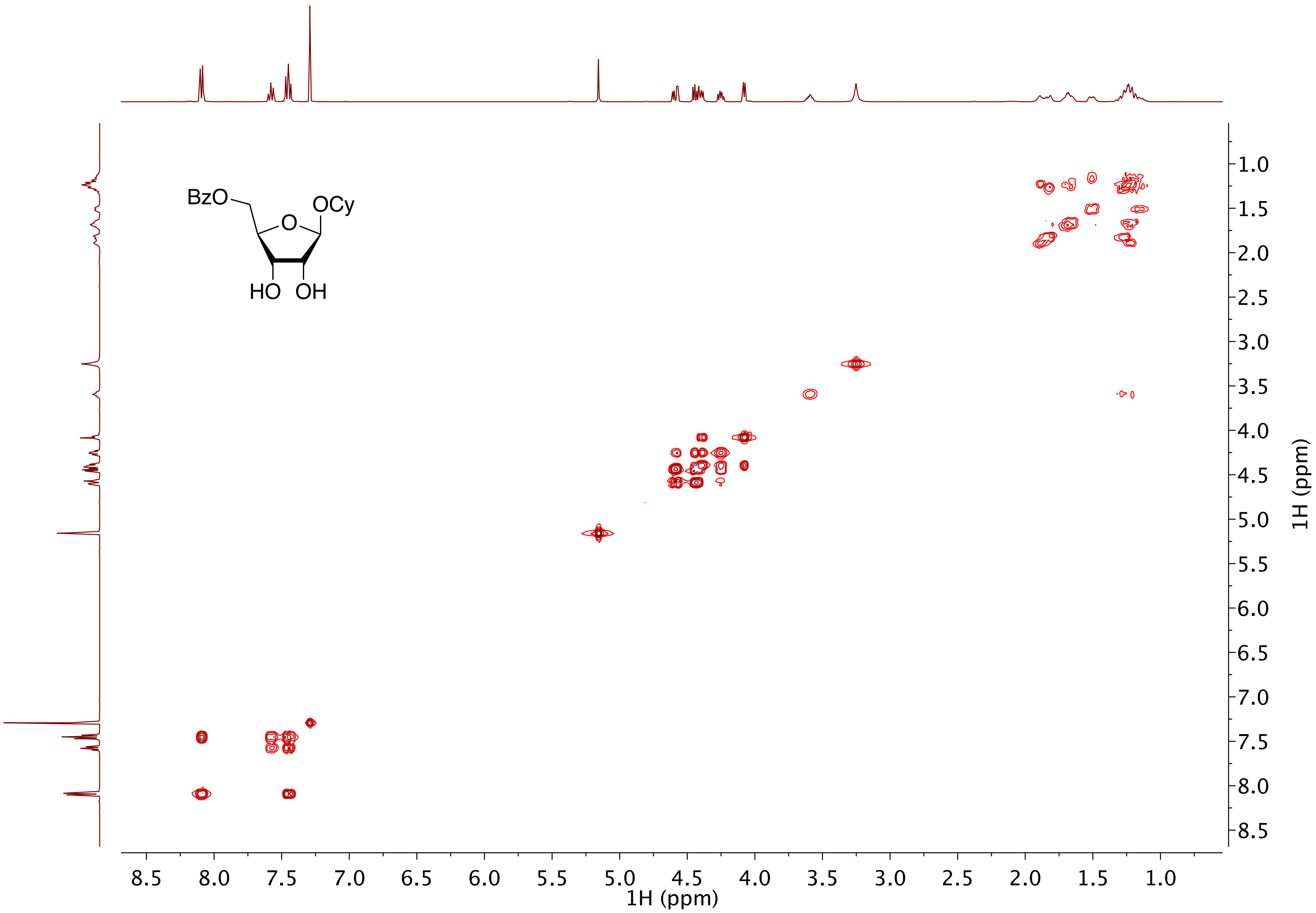

1d –^{1}H NMR (400 MHz, CDCl$_3$)

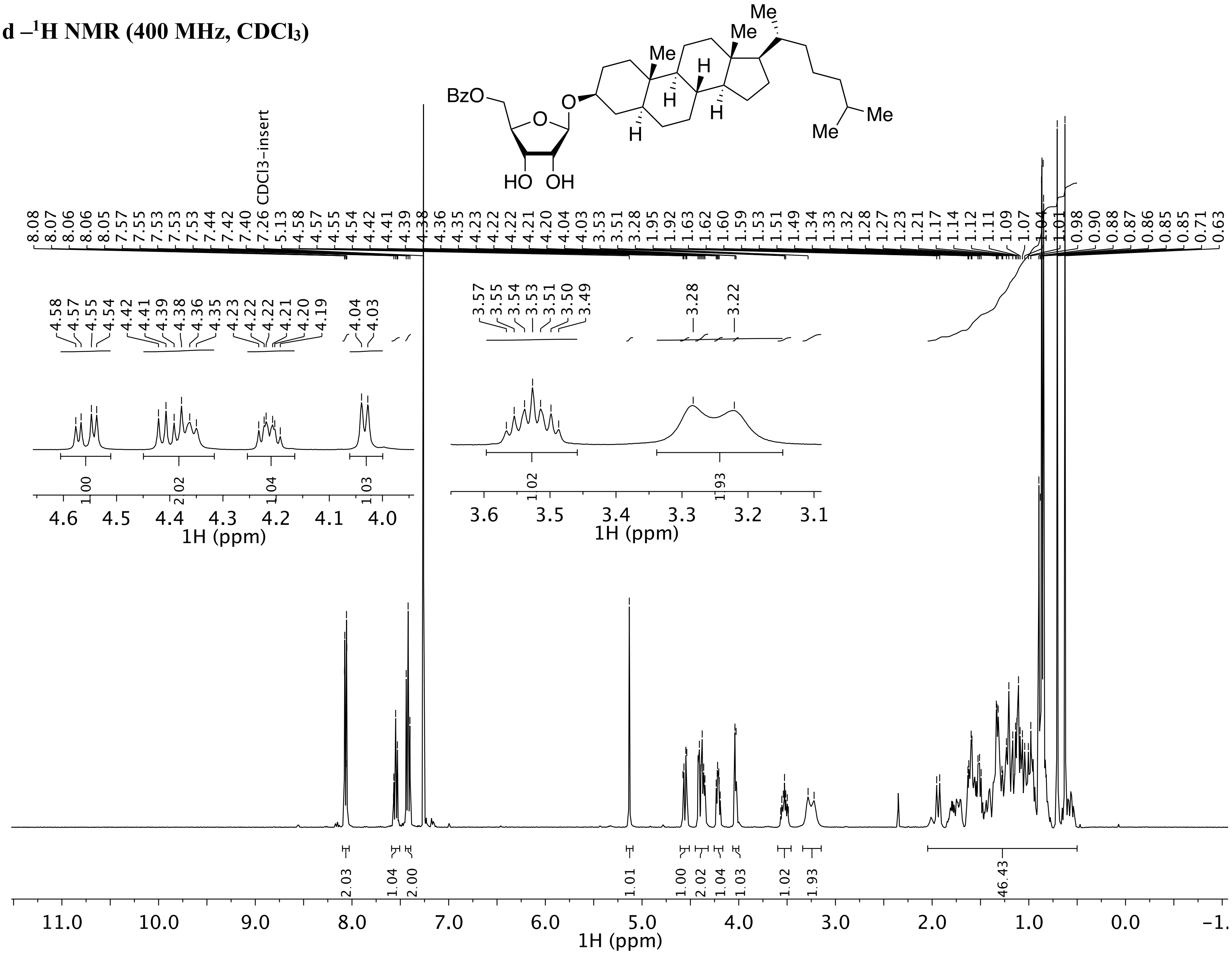

1d –^{13}C NMR (101 MHz, CDCl$_3$)

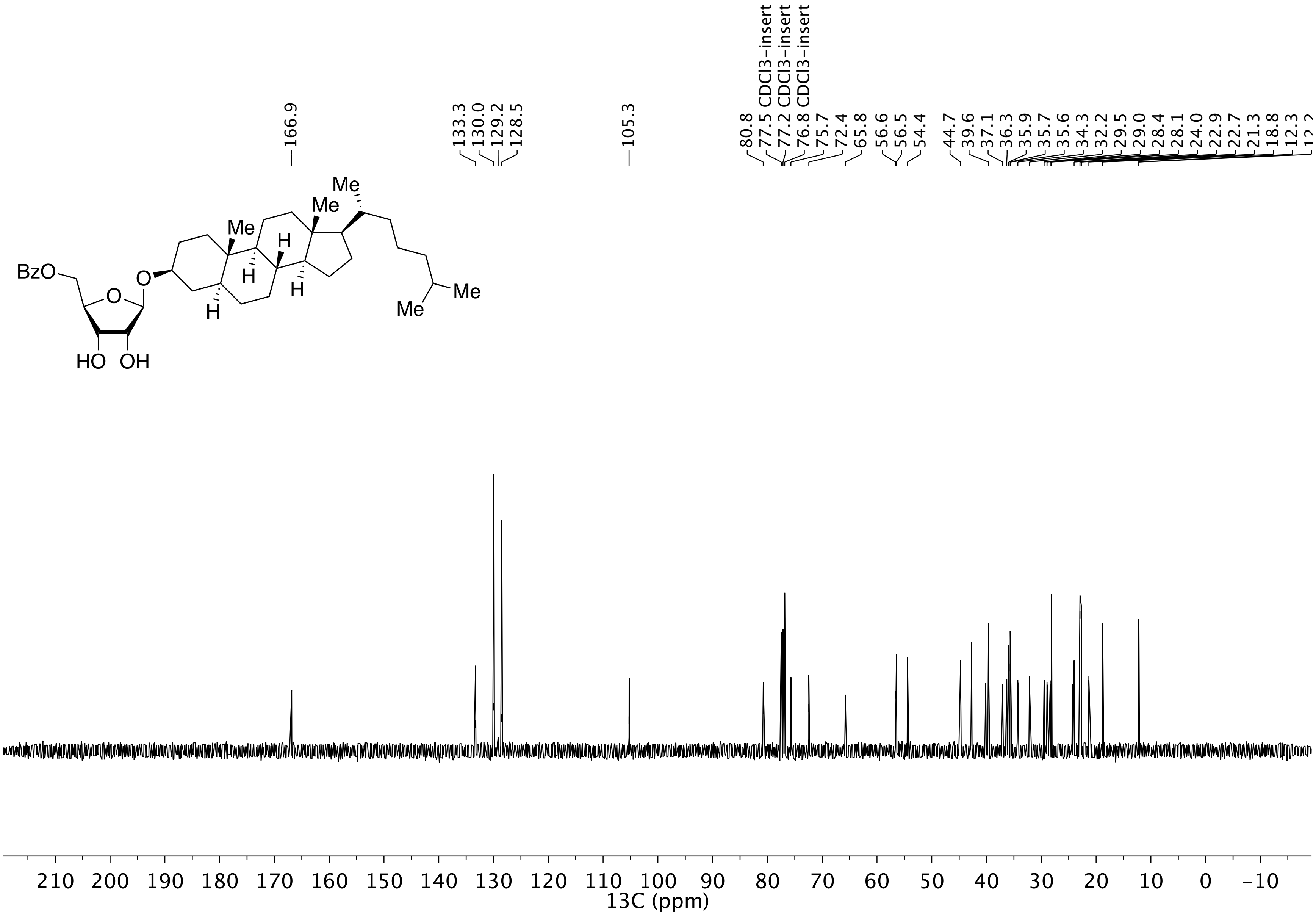

1d – COSY (400 MHz, $CDCl_3$)

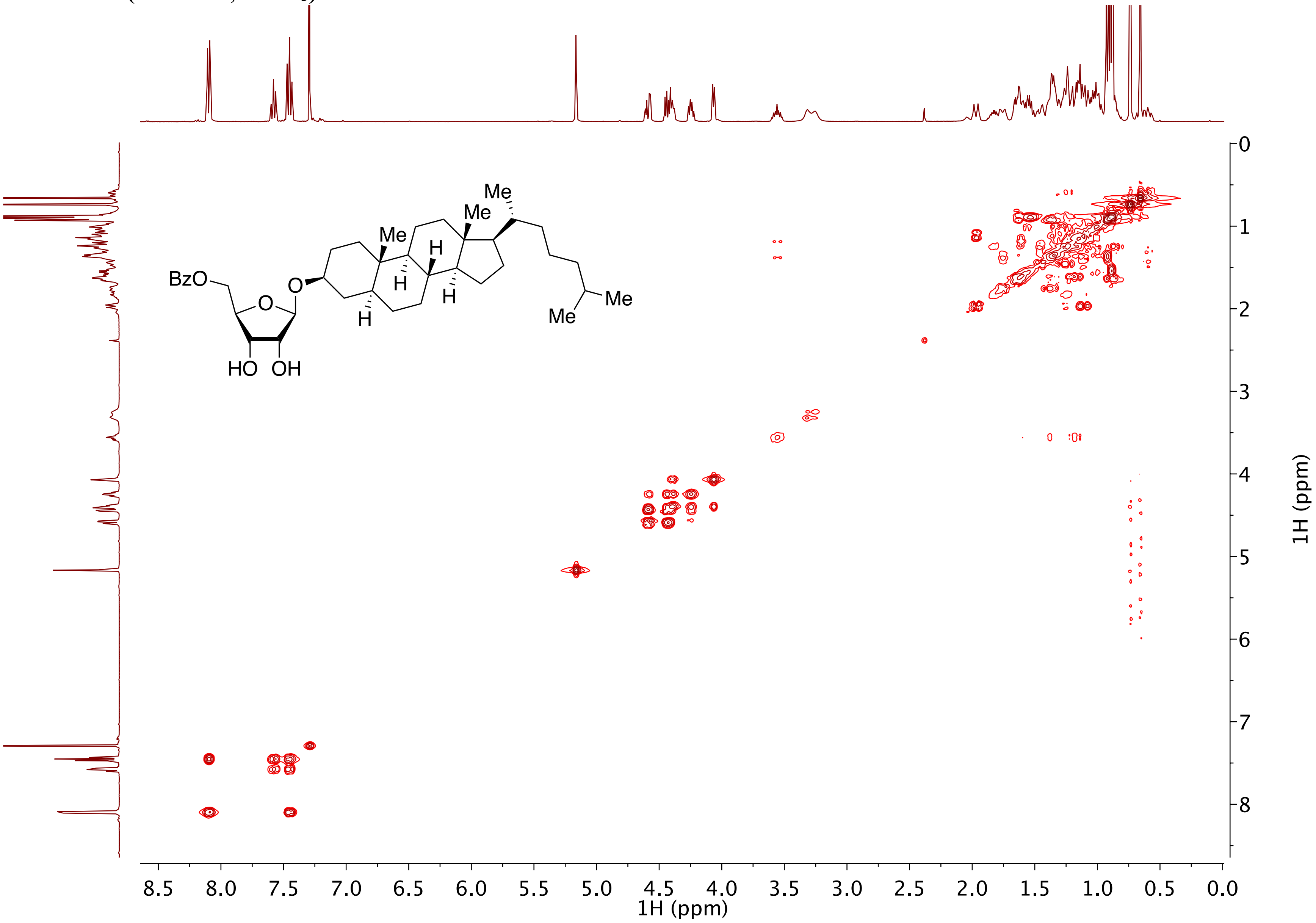

1e –^{1}H NMR (400 MHz, $CDCl_3$)

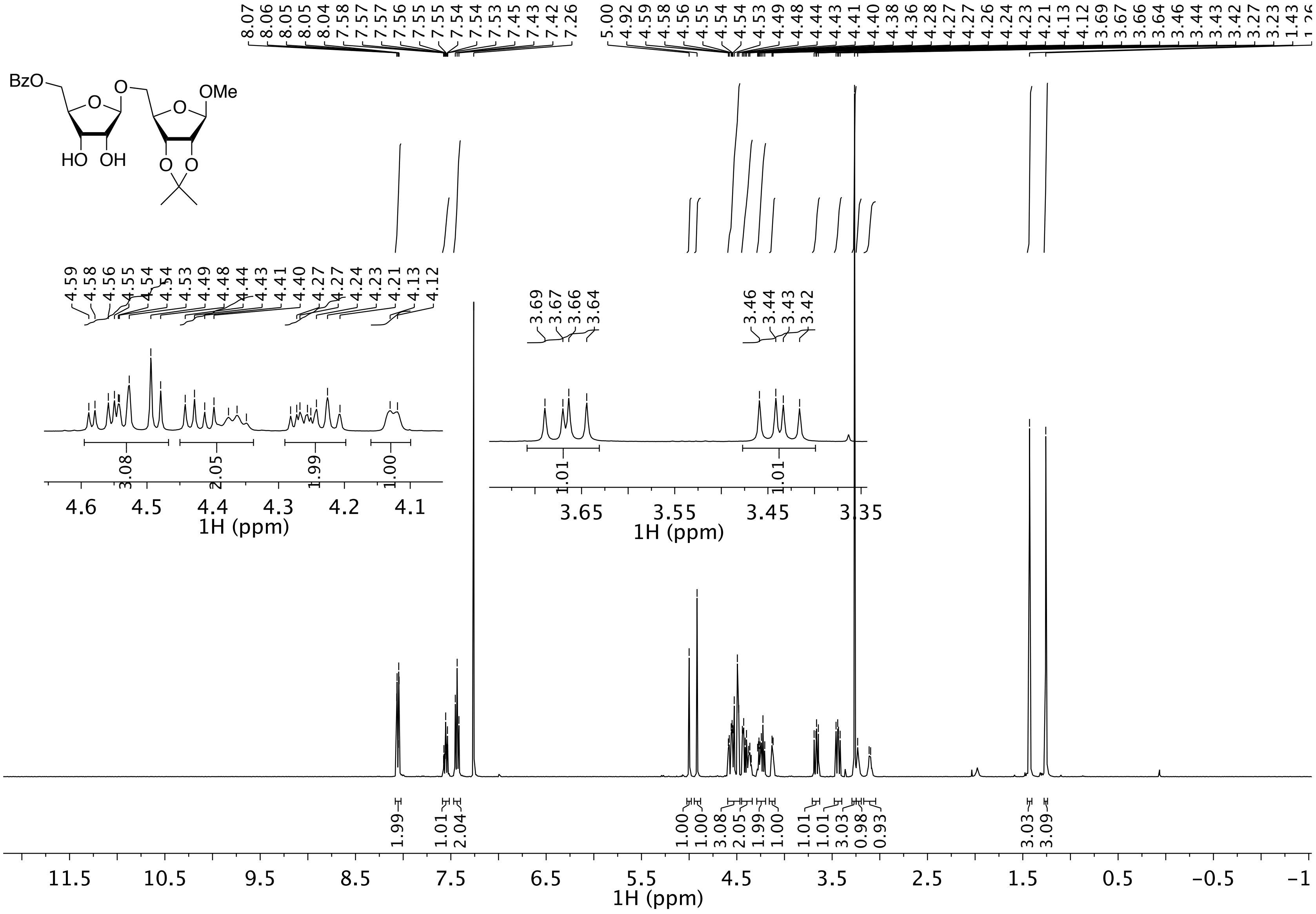

1e –^{13}C NMR (101 MHz, $CDCl_3$)

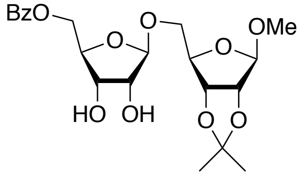

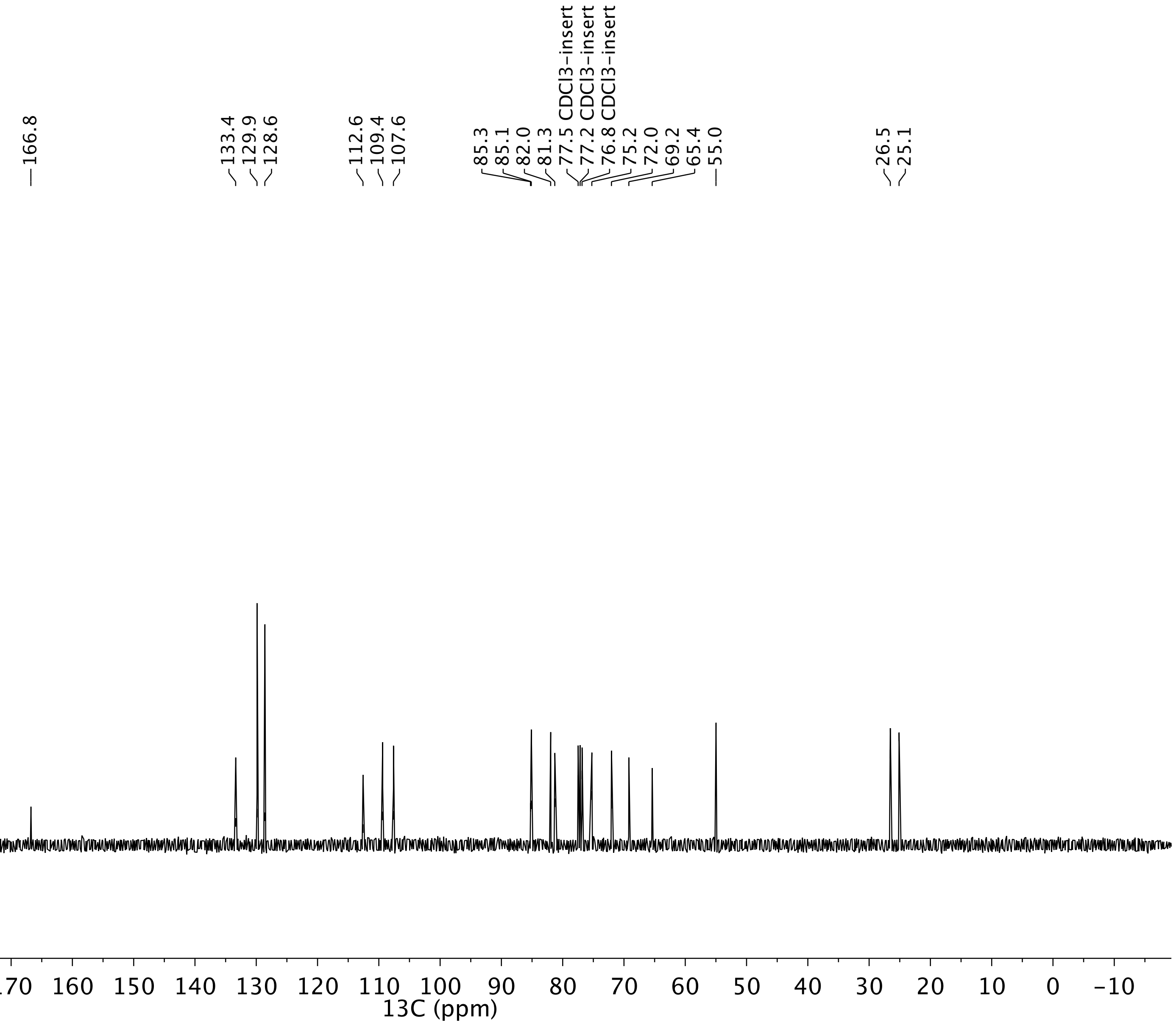

1e –COSY (400 MHz, CDCl3)

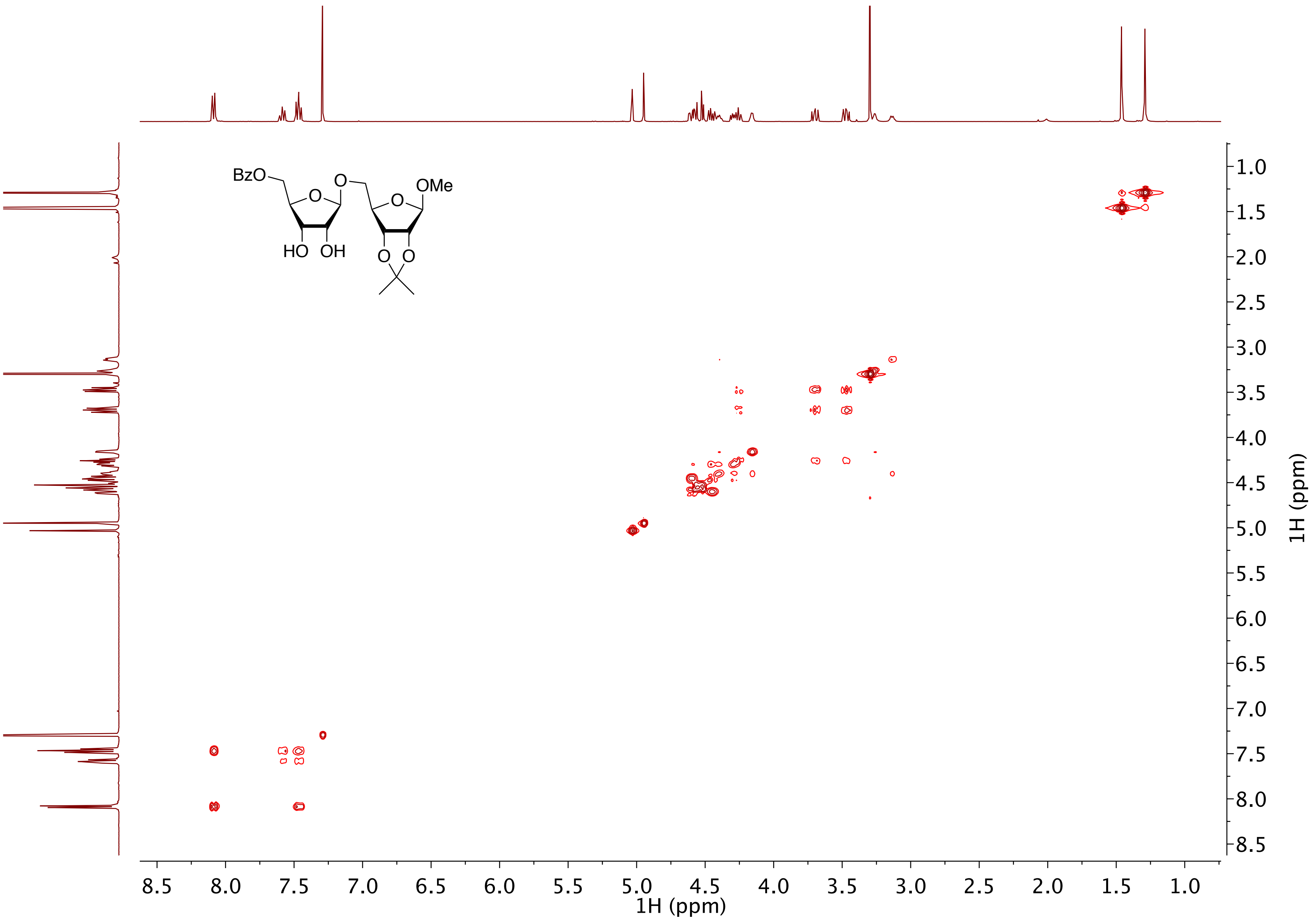

1f –^{1}H NMR (500 MHz, CDCl₃)

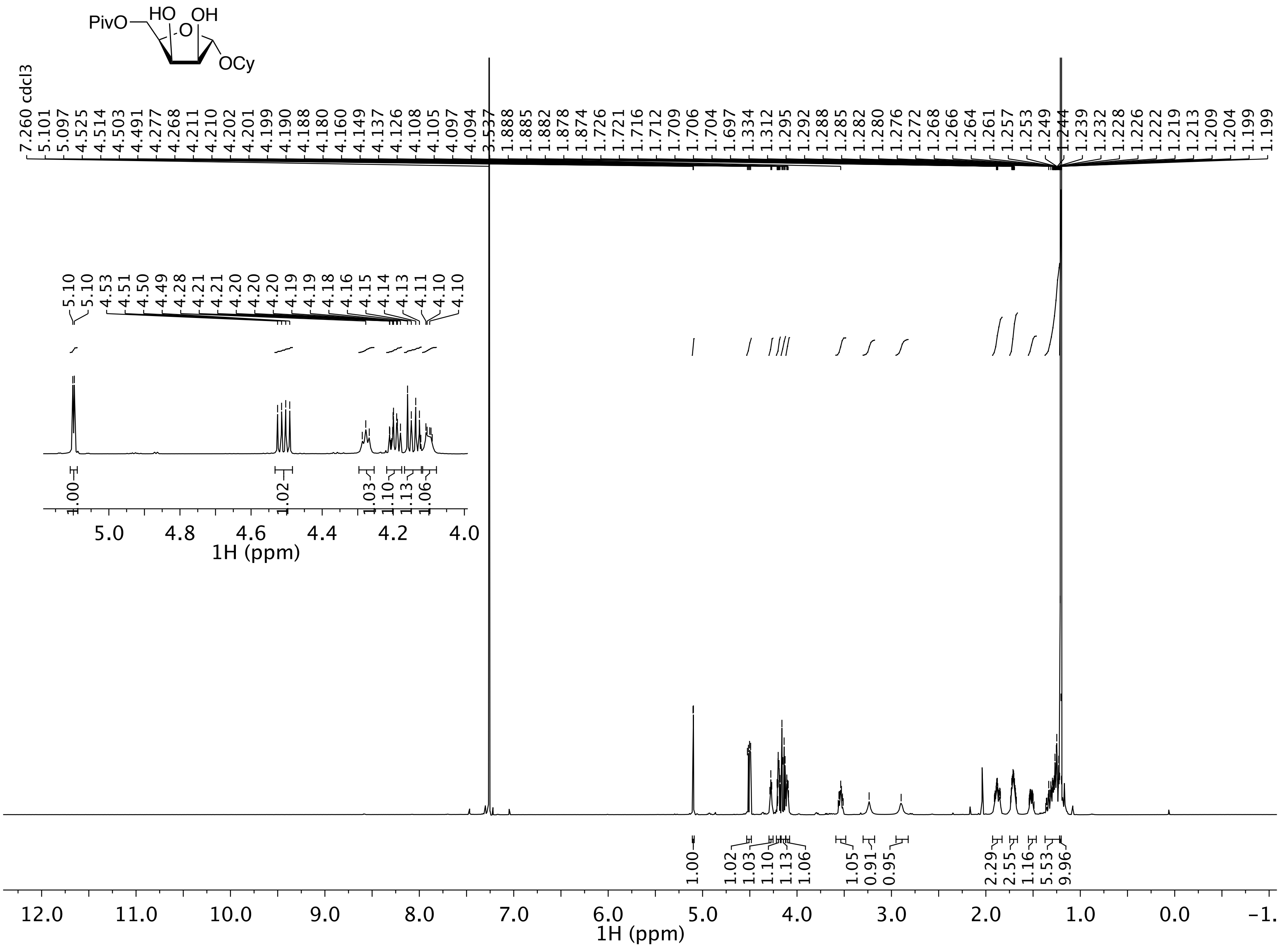

1f –^{13}C NMR (126 MHz, CDCl$_3$)

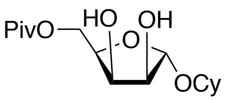

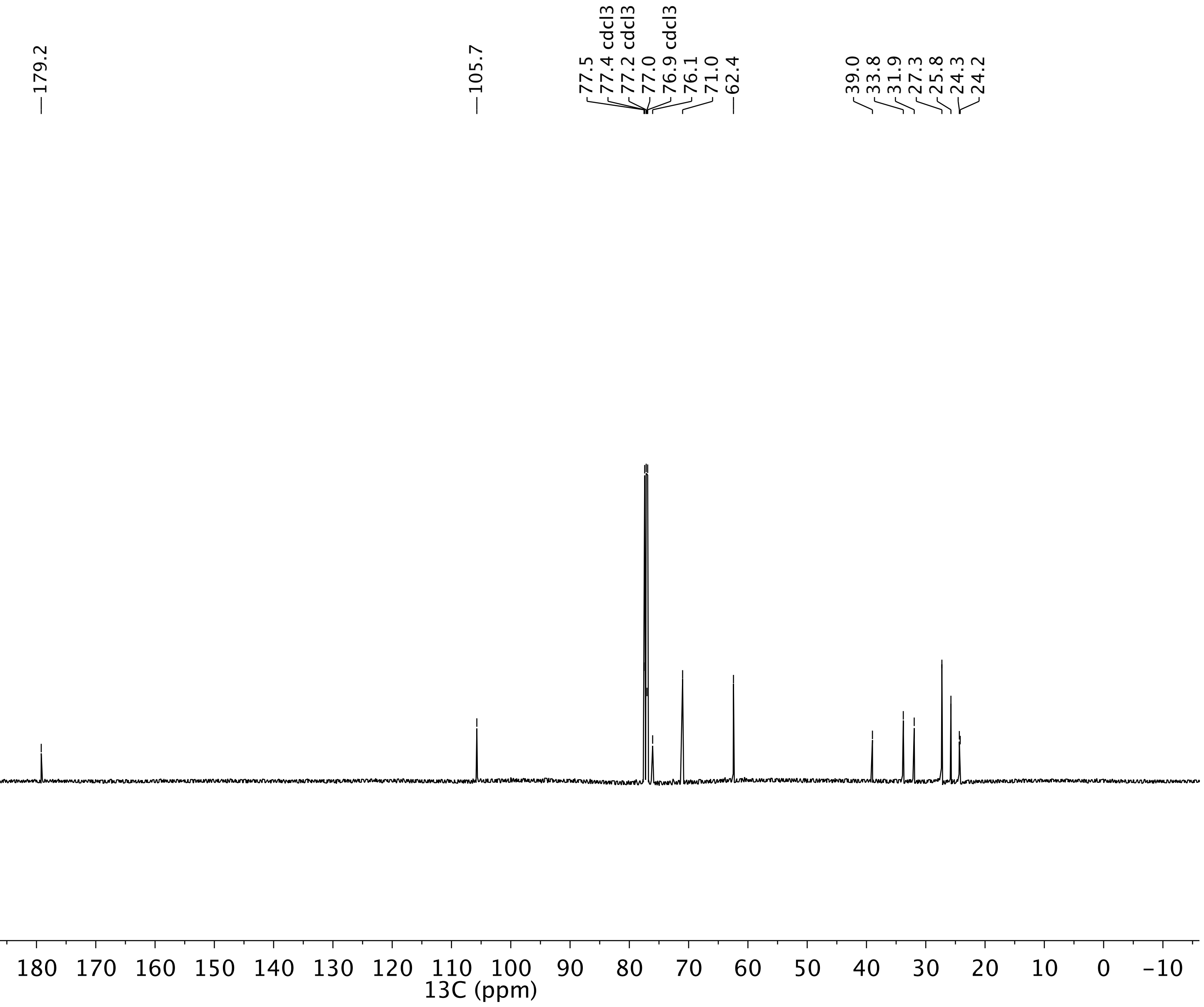

1f –COSY (500 MHz, $CDCl_3$)

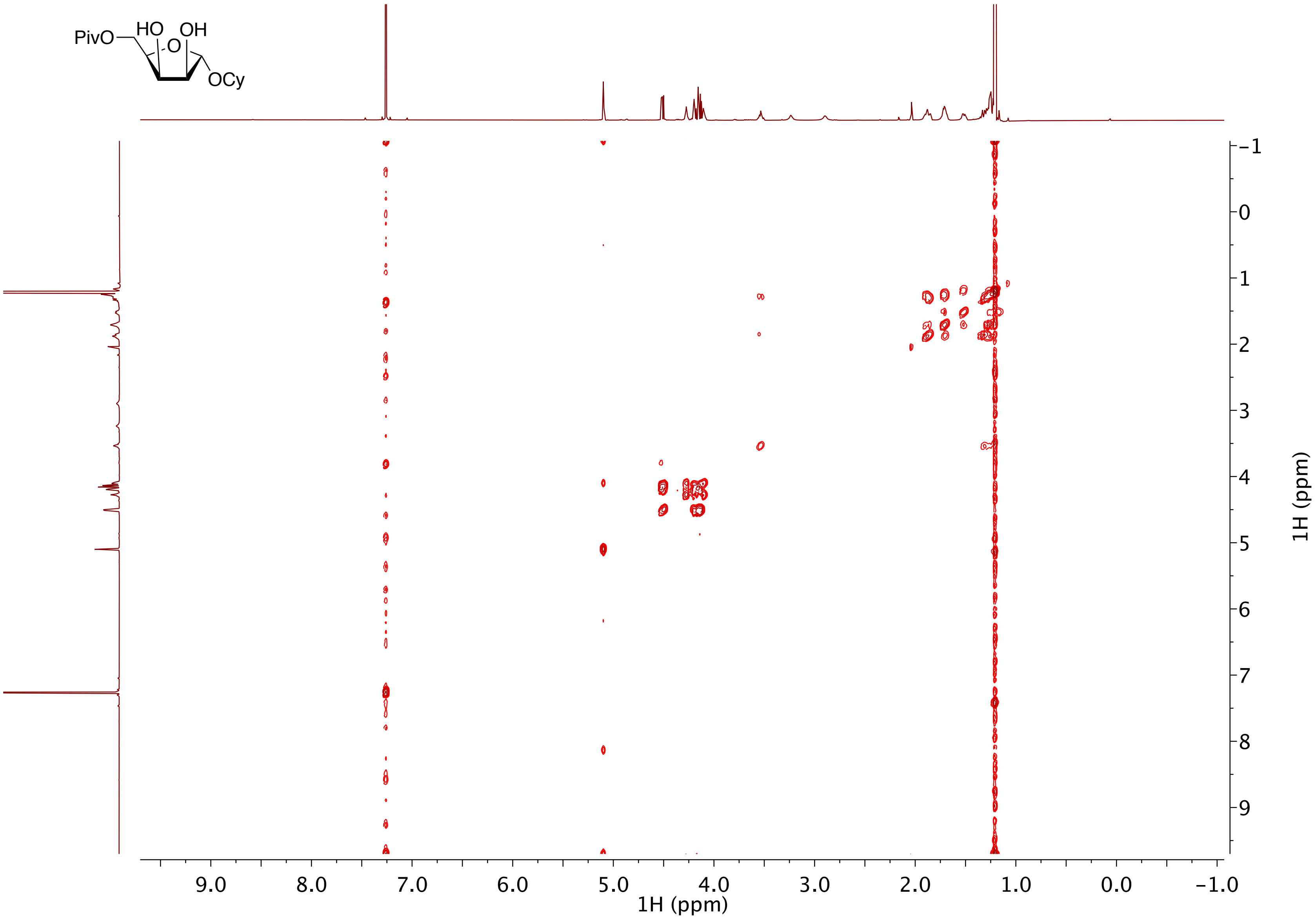

1g –^{1}H NMR (500 MHz, CDCl₃)

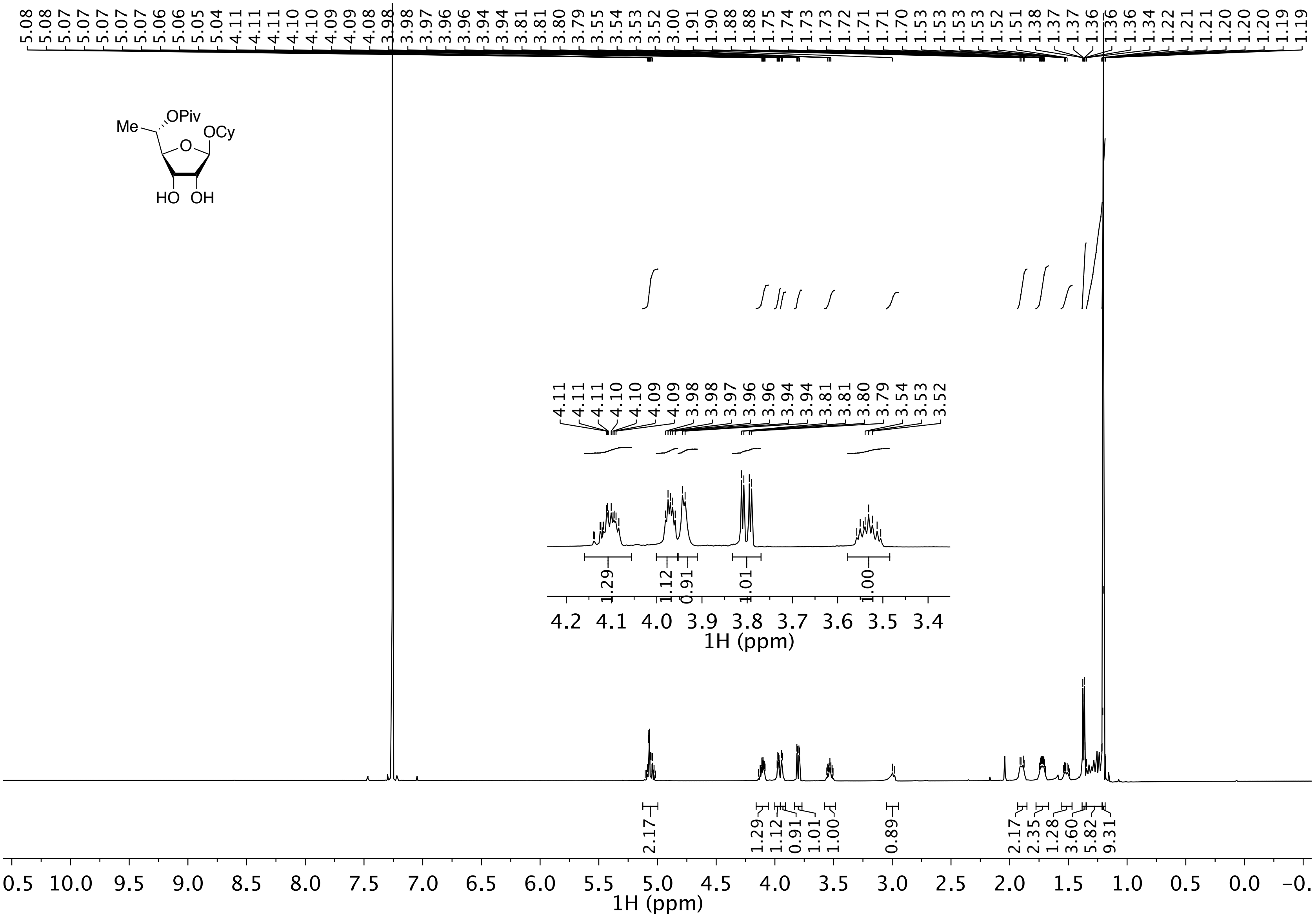

1g –^{13}C NMR (126 MHz, CDCl$_3$)

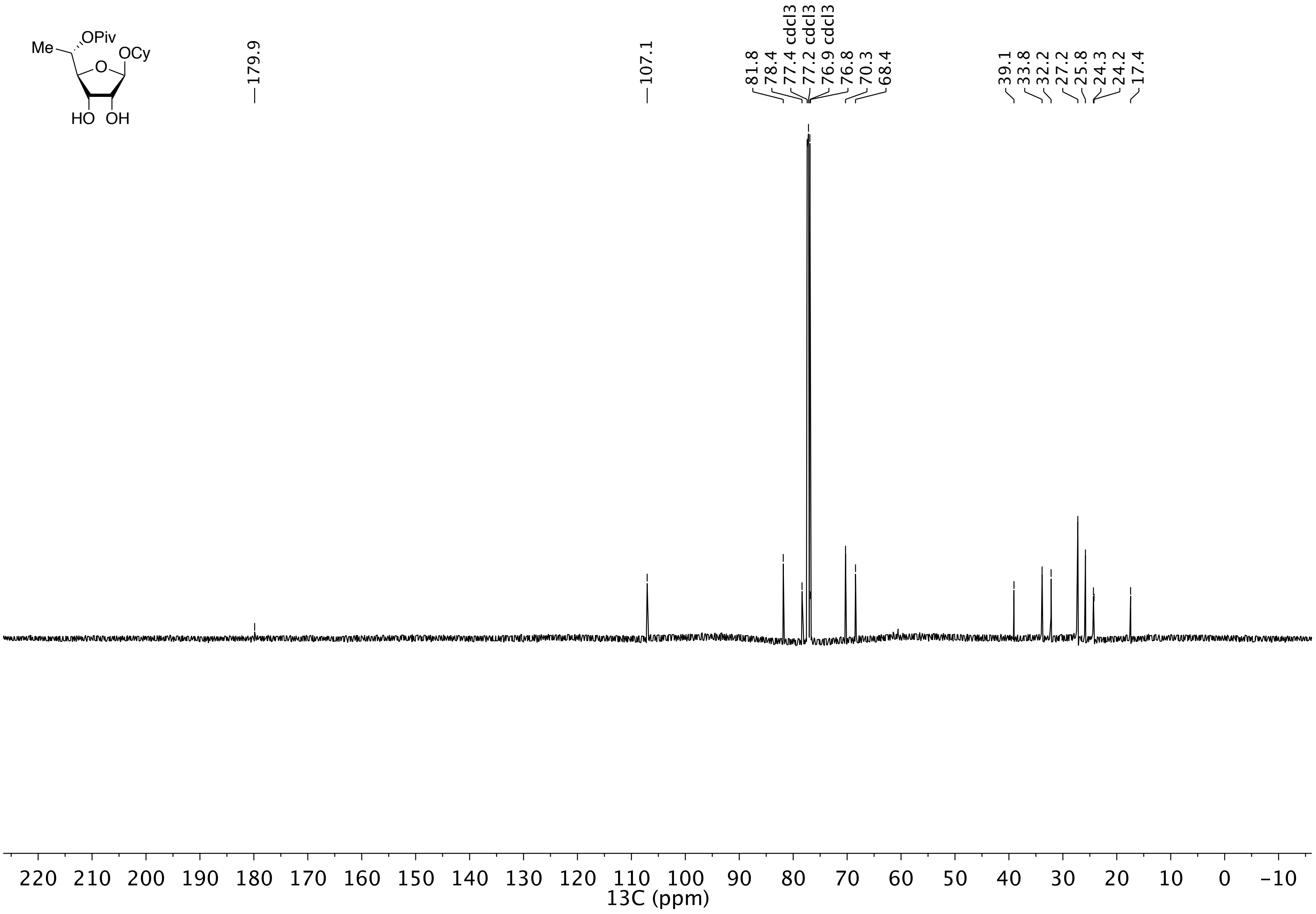

1g –COSY (500 MHz, CDCl₃)

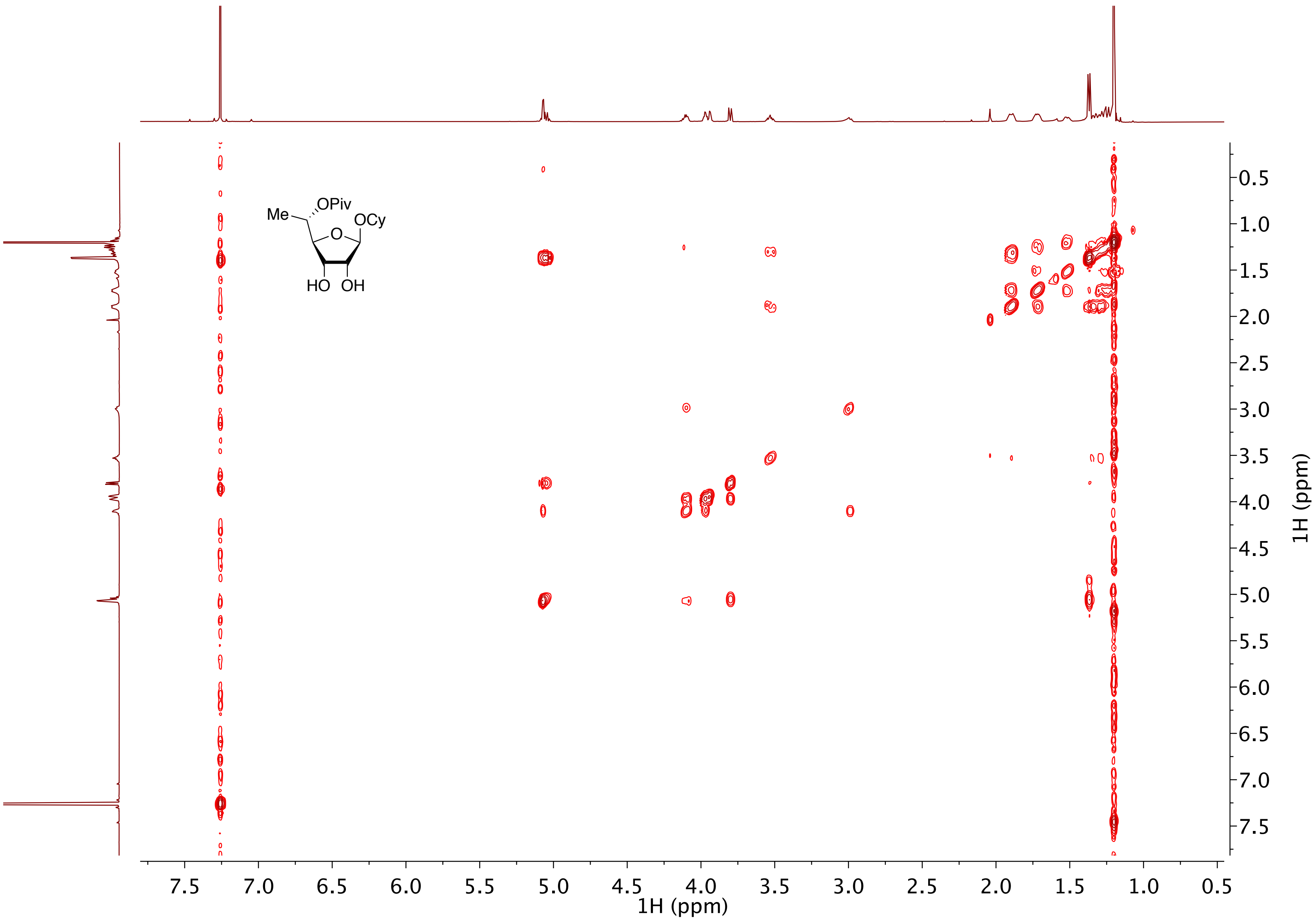

1h –^{1}H NMR (400 MHz, CDCl$_3$)

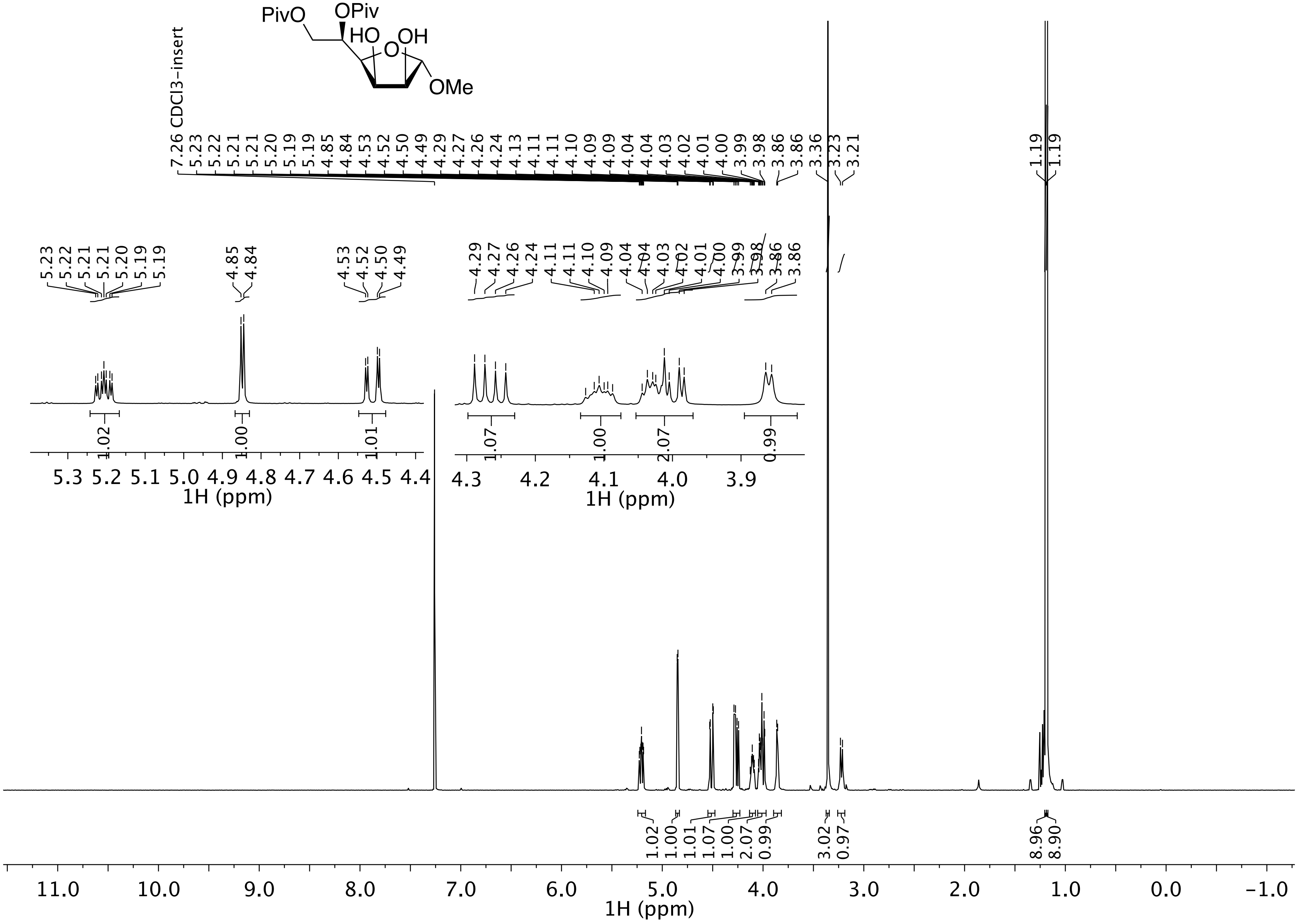

1h –^{13}CNMR (101 MHz, CDCl$_3$)

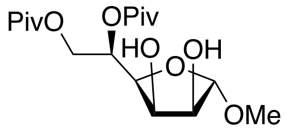

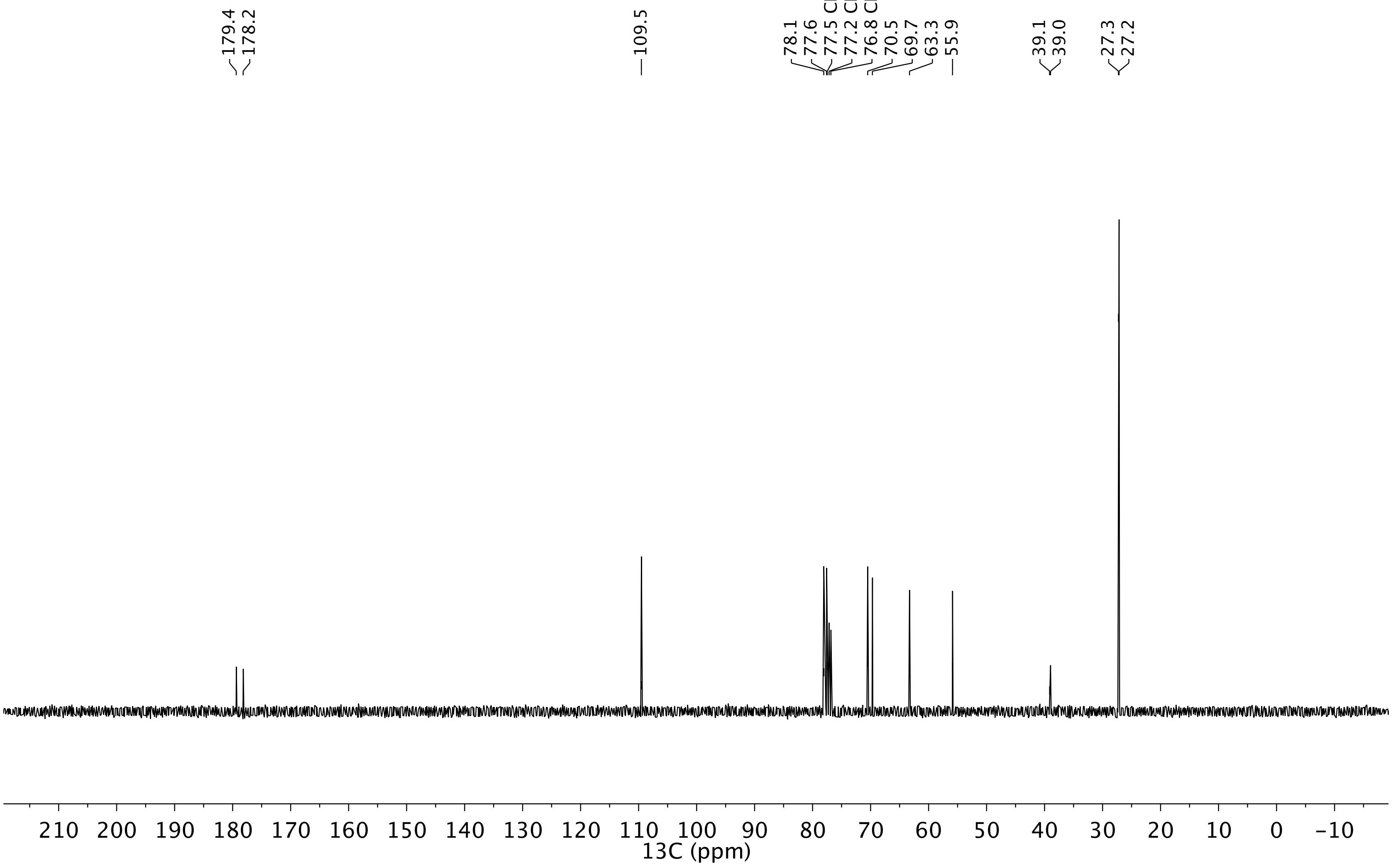

1h –COSY (400 MHz, $CDCl_3$)

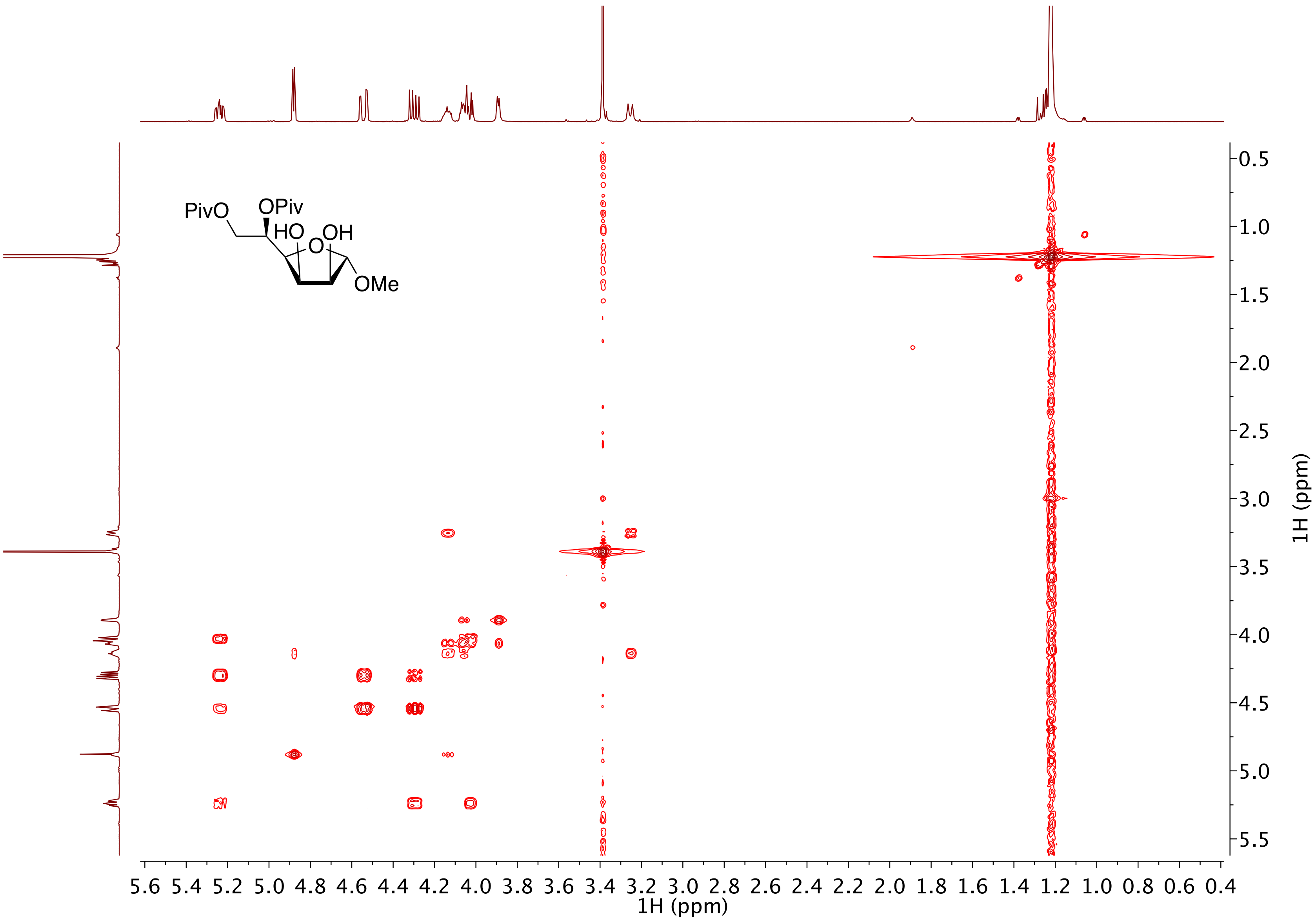

1i –^{1}H NMR (400 MHz, $CDCl_3$)

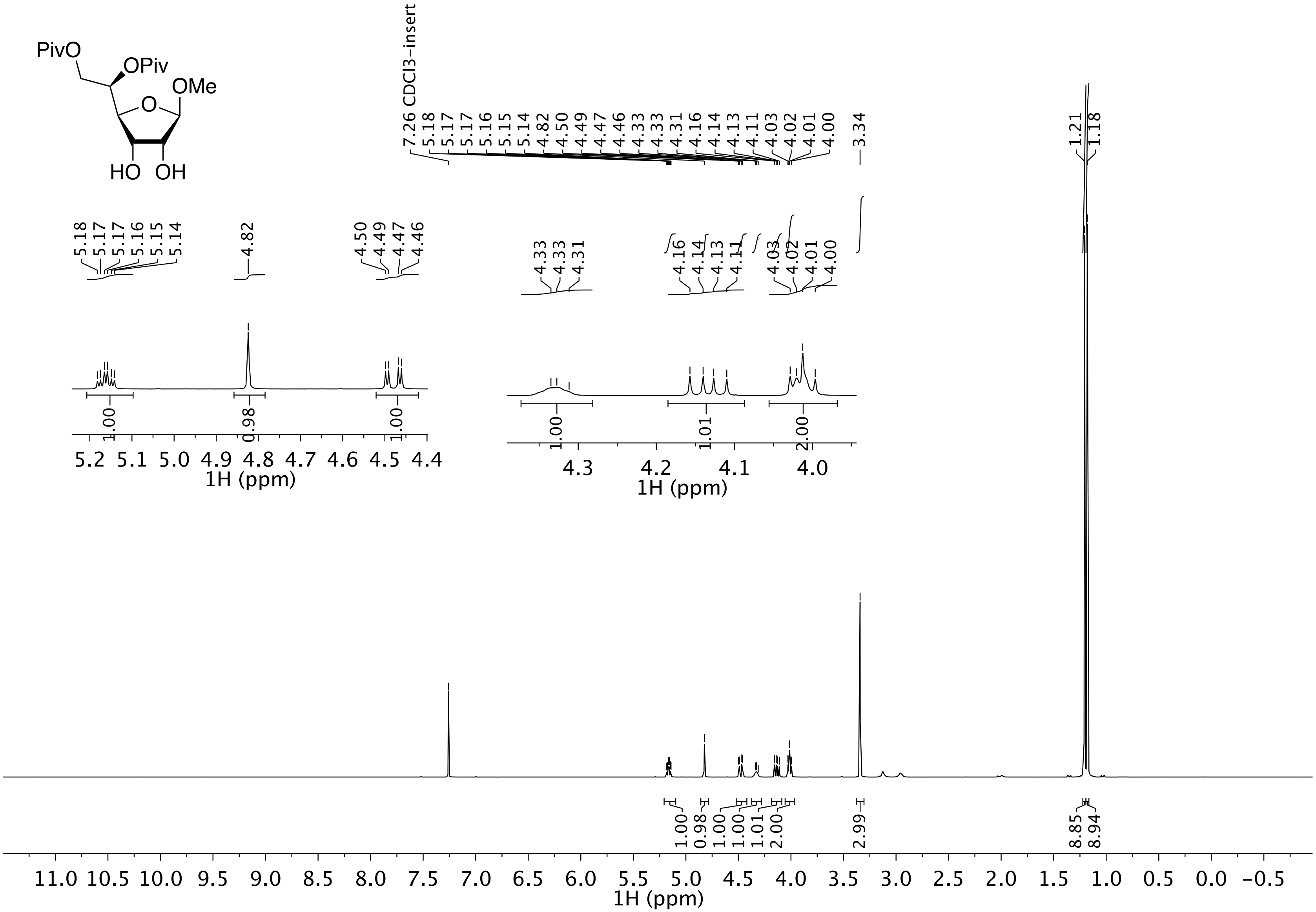

1i –^{13}C NMR (101 MHz, CDCl$_3$)

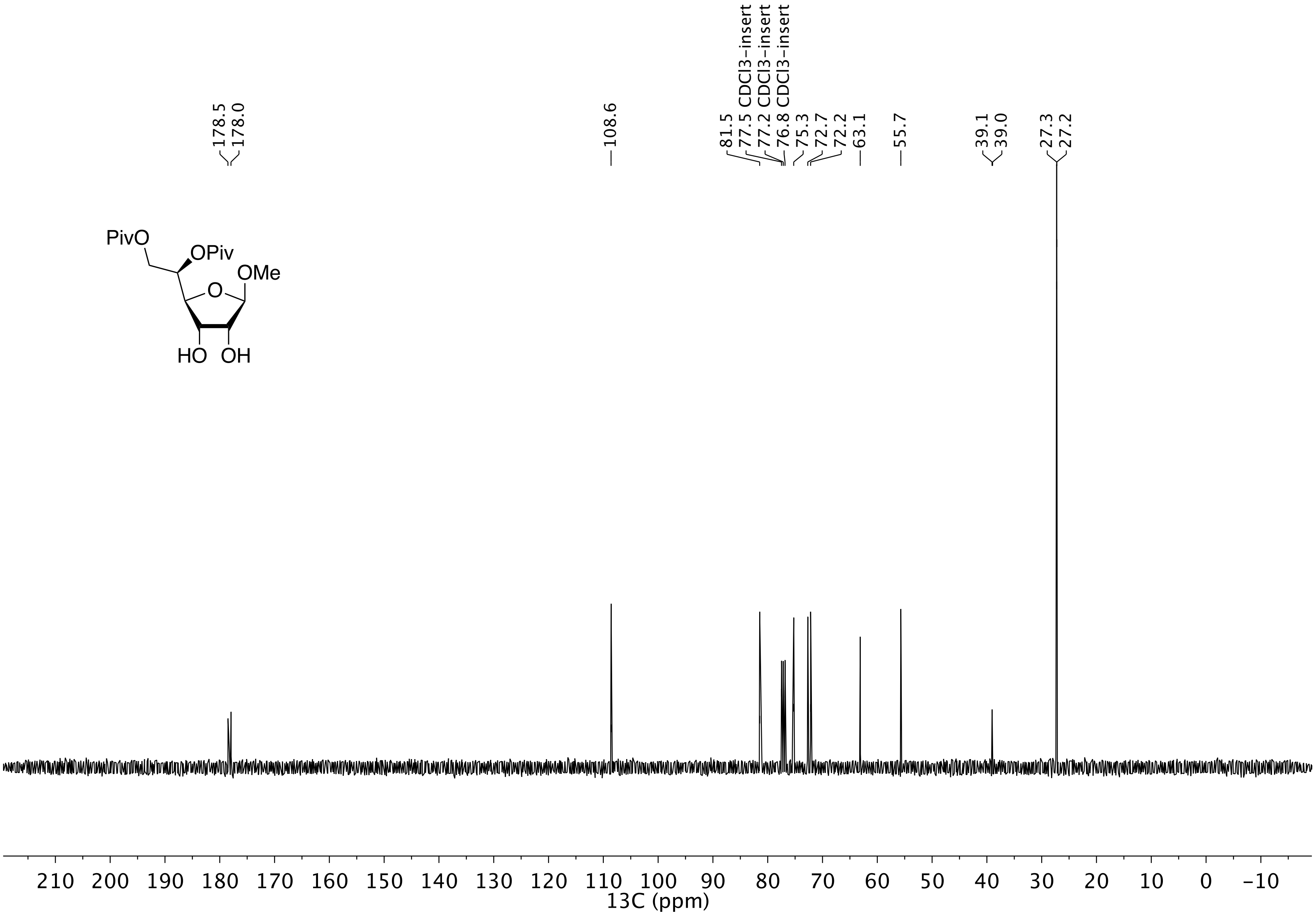

1i – COSY (400 MHz, $CDCl_3$)

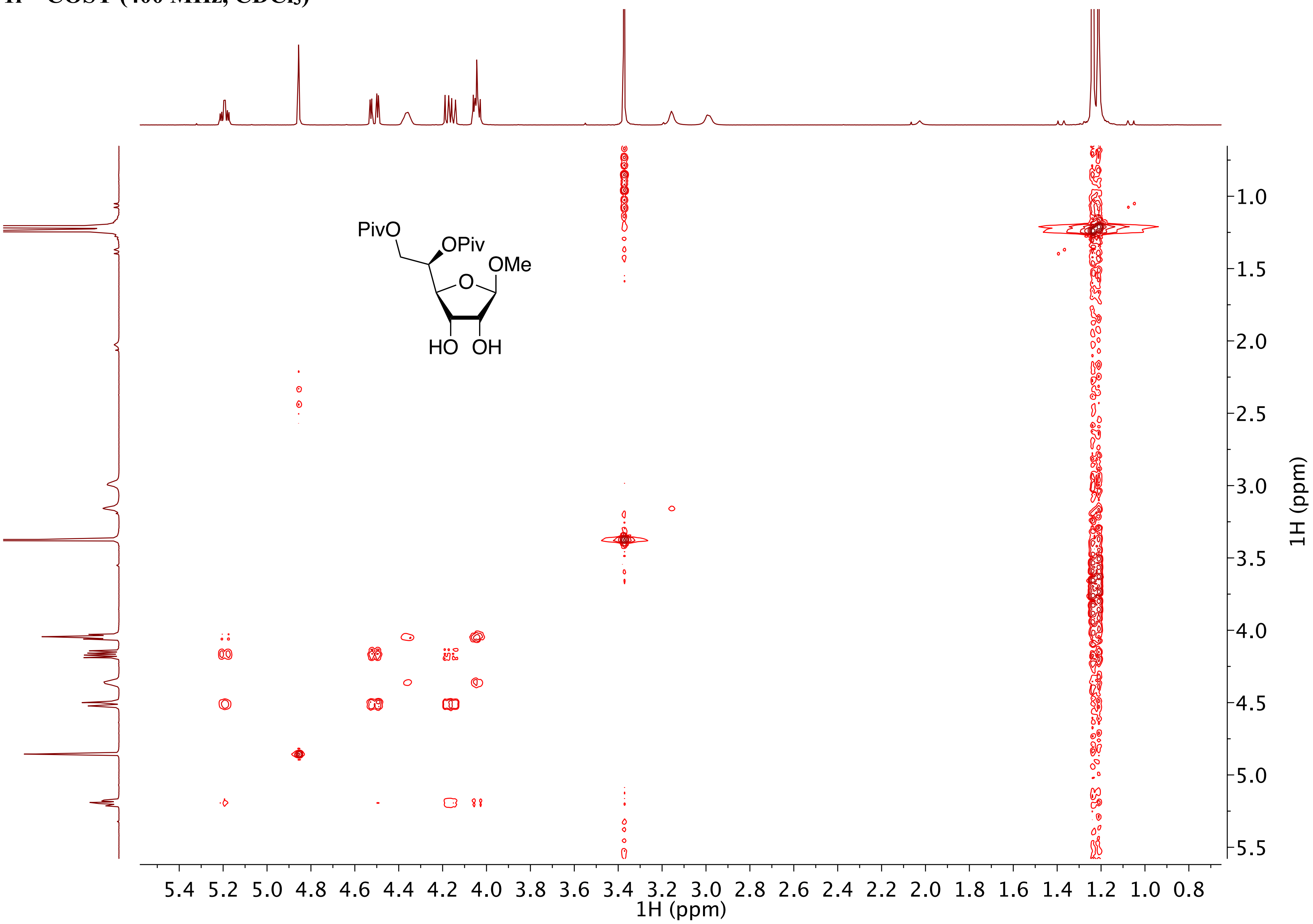

1m –^{1}H NMR (400 MHz, DMSO-d_6)

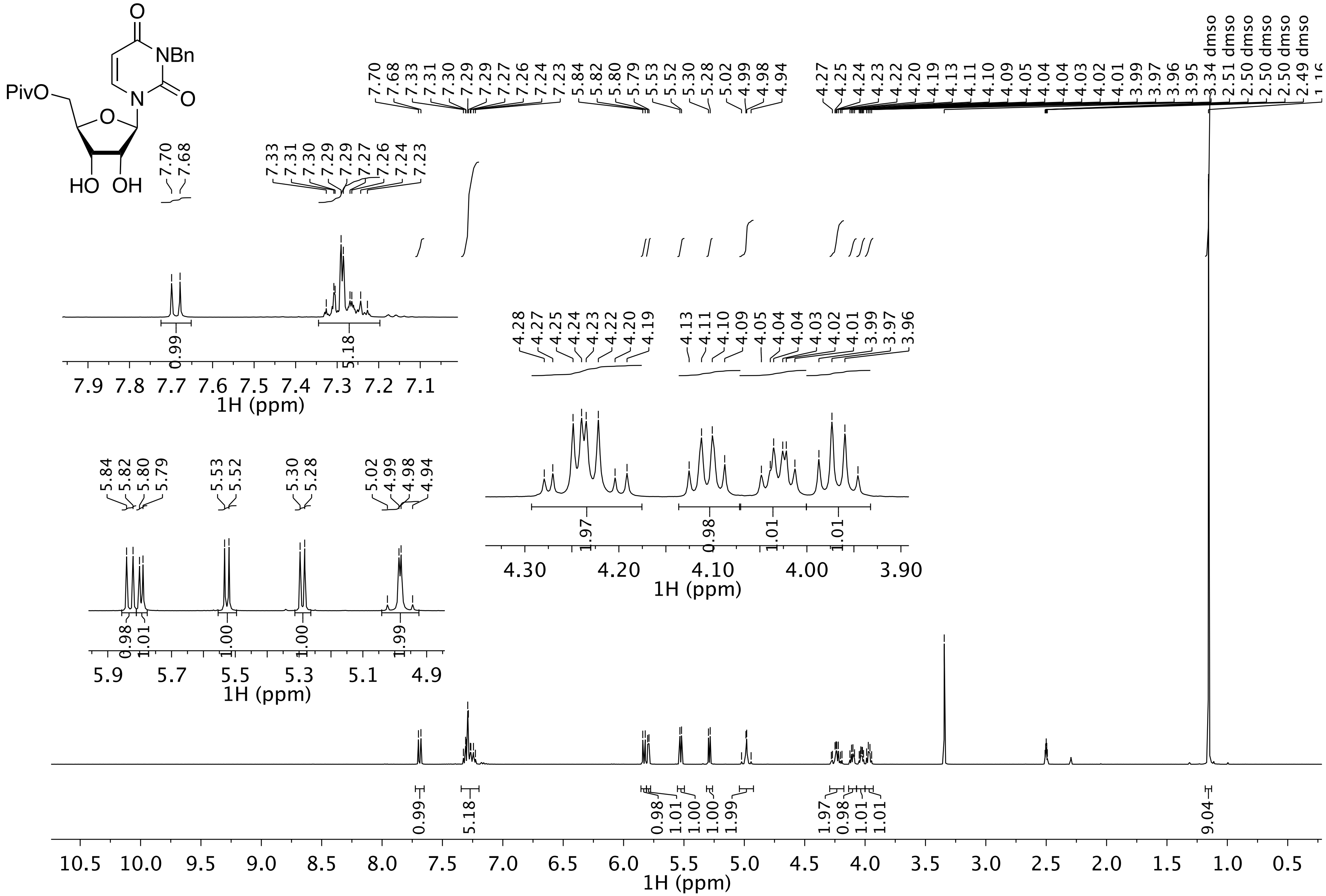

1m –^{13}C NMR (101 MHz, DMSO-*d*$_6$)

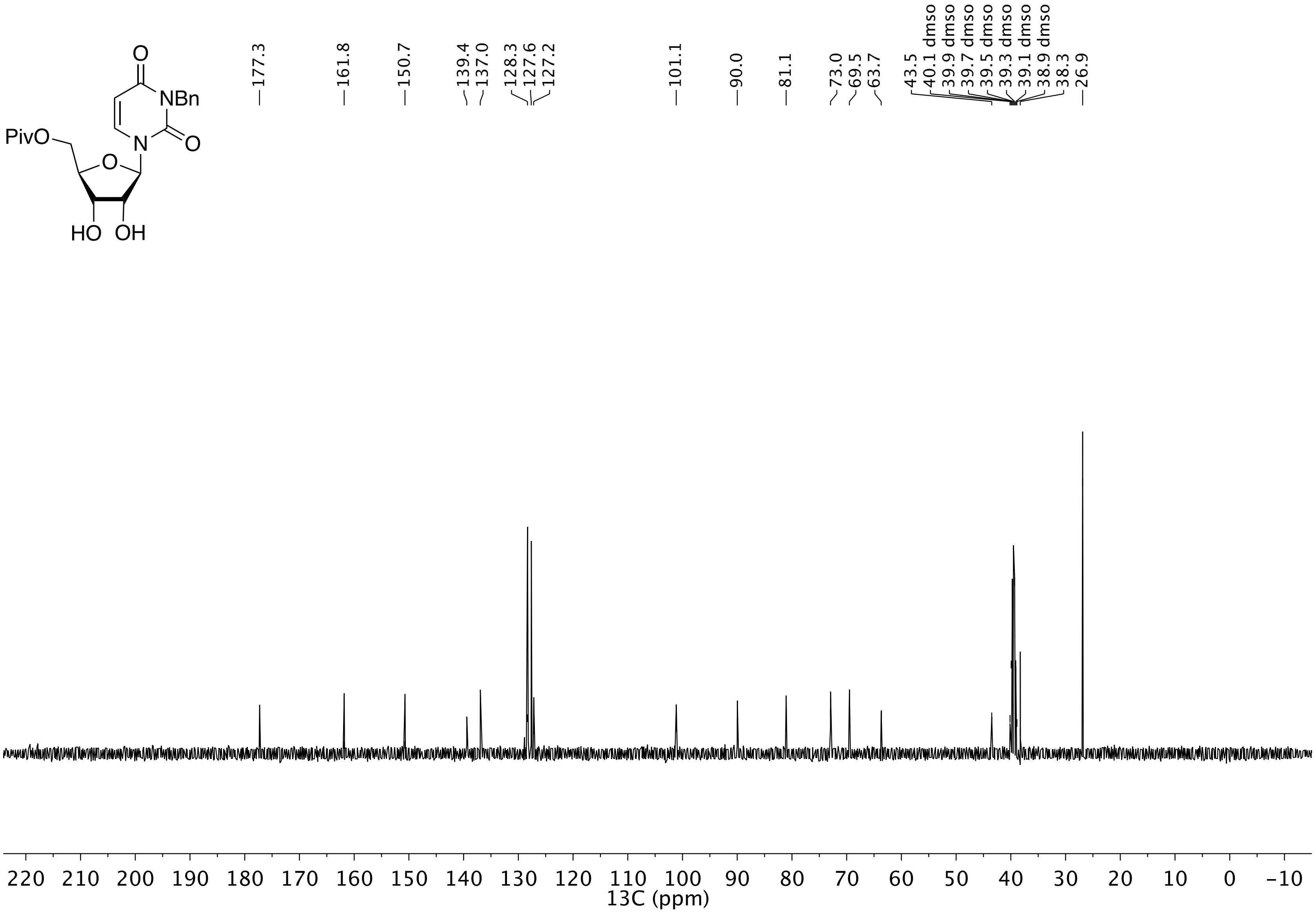

1m – COSY (400 MHz, DMSO-d_6)

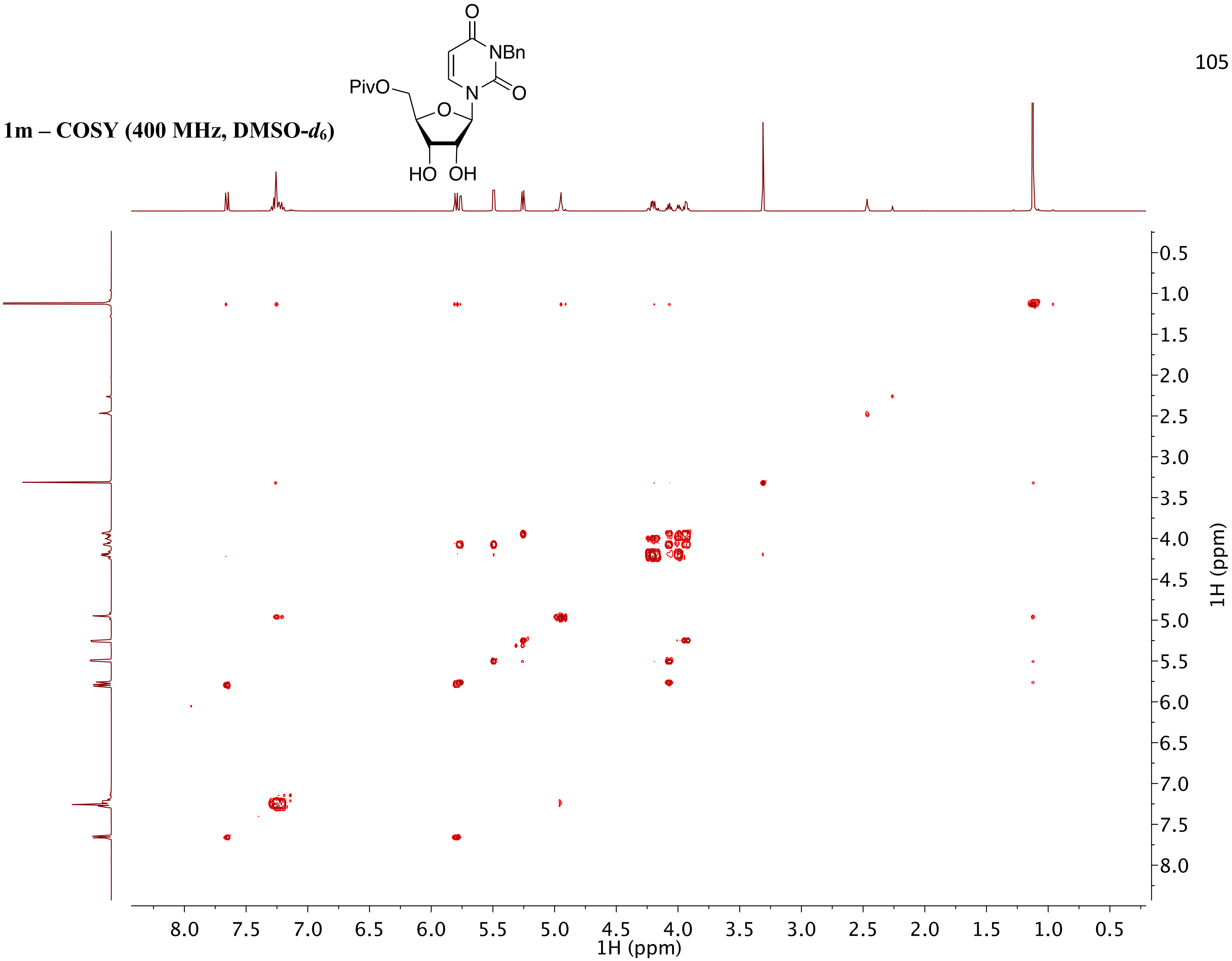

2a –^{1}H NMR (500 MHz, $CDCl_3$)

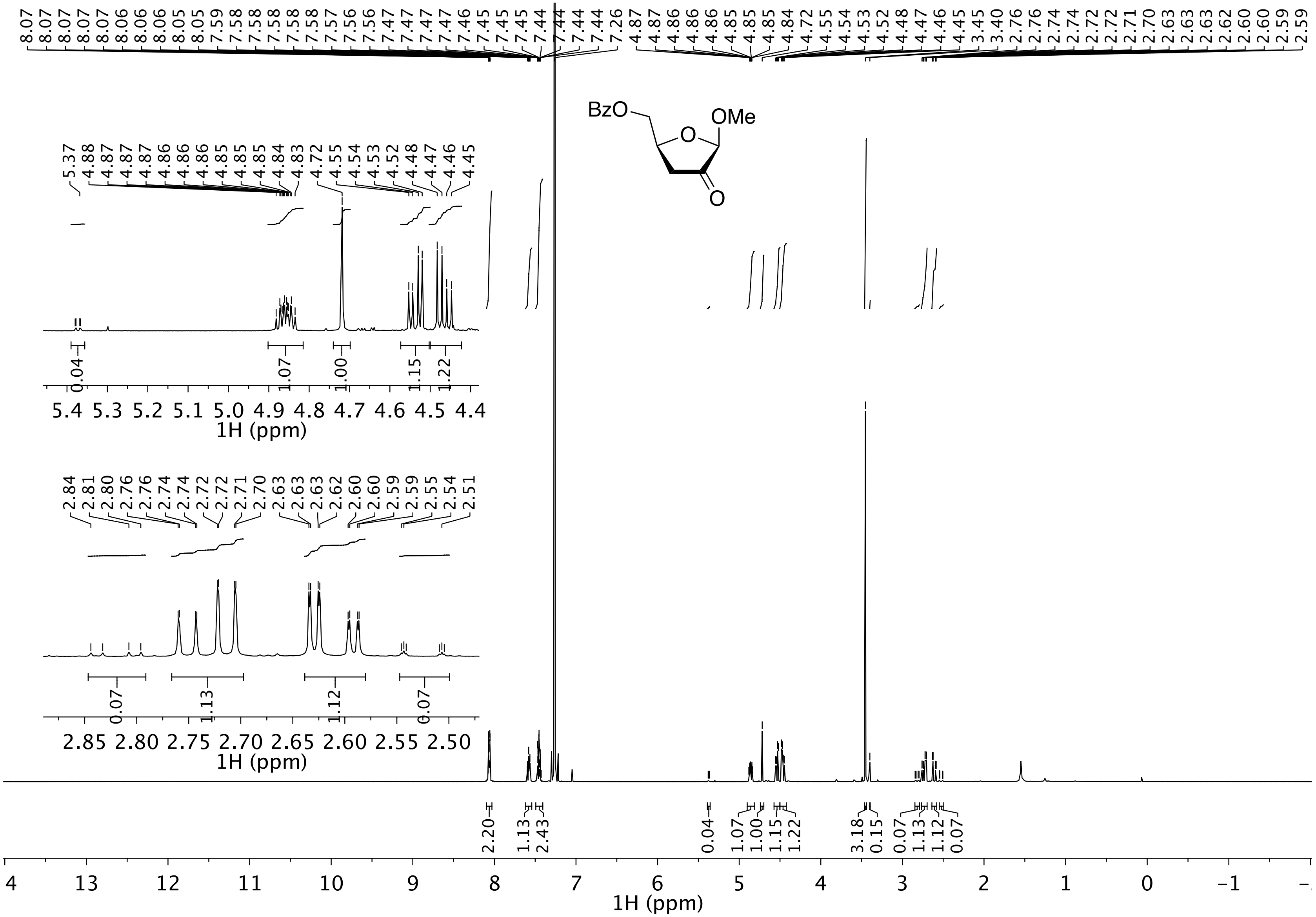

2a –^{13}C NMR (126 MHz, CDCl$_3$)

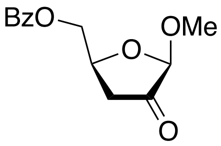

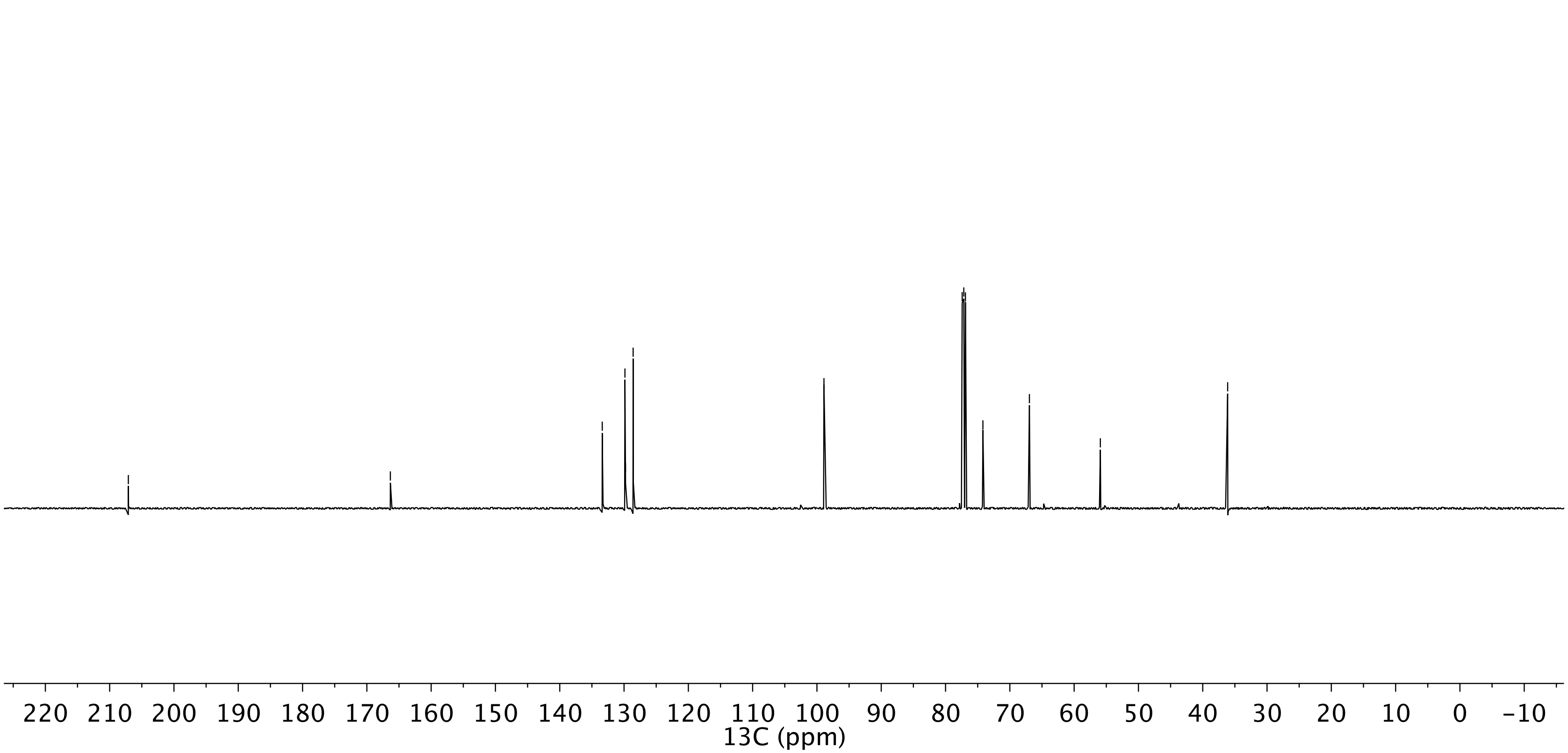

2a – COSY (500 MHz, $CDCl_3$)

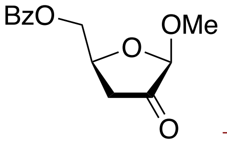

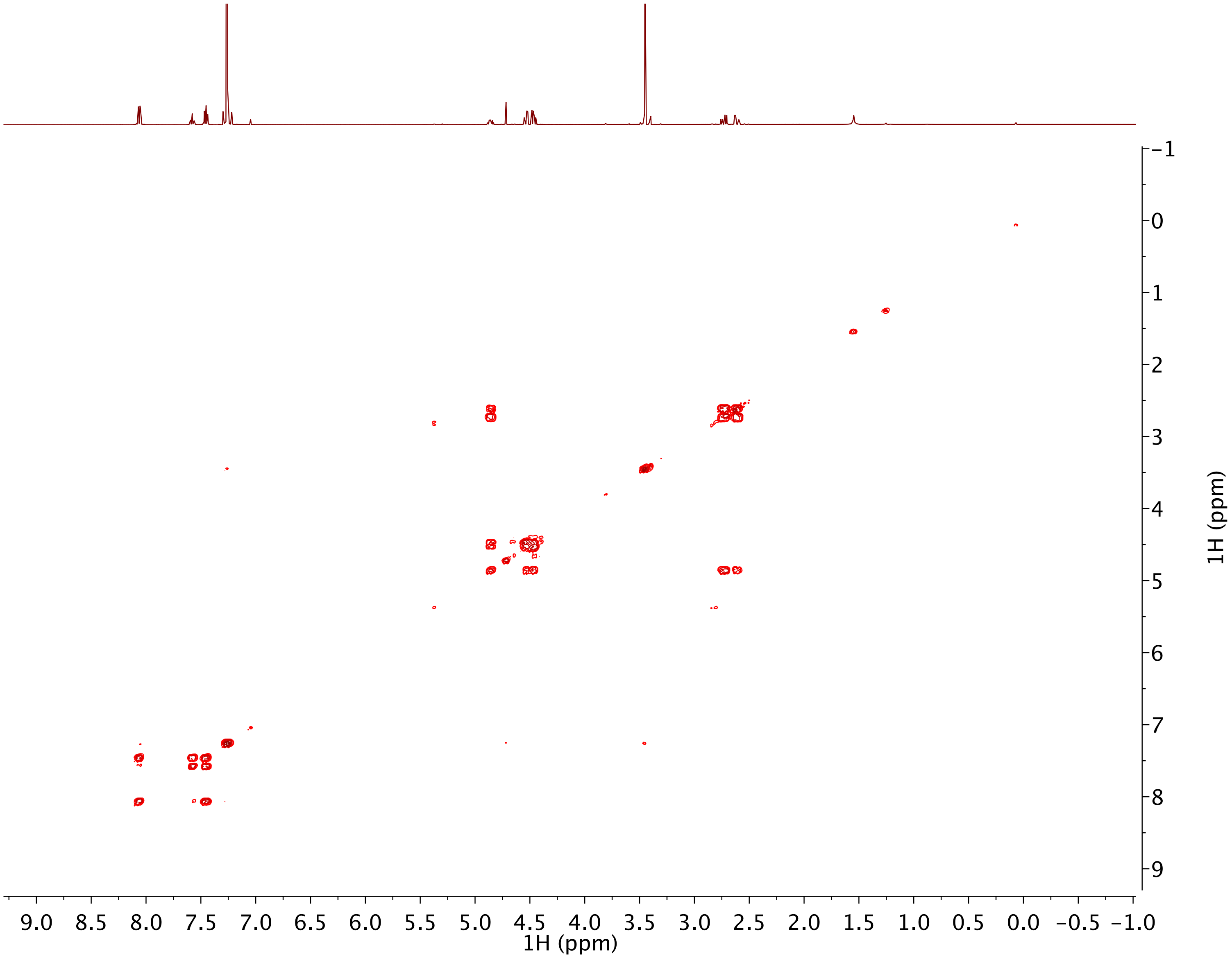

2b –^{1}H NMR (400 MHz, CDCl₃)

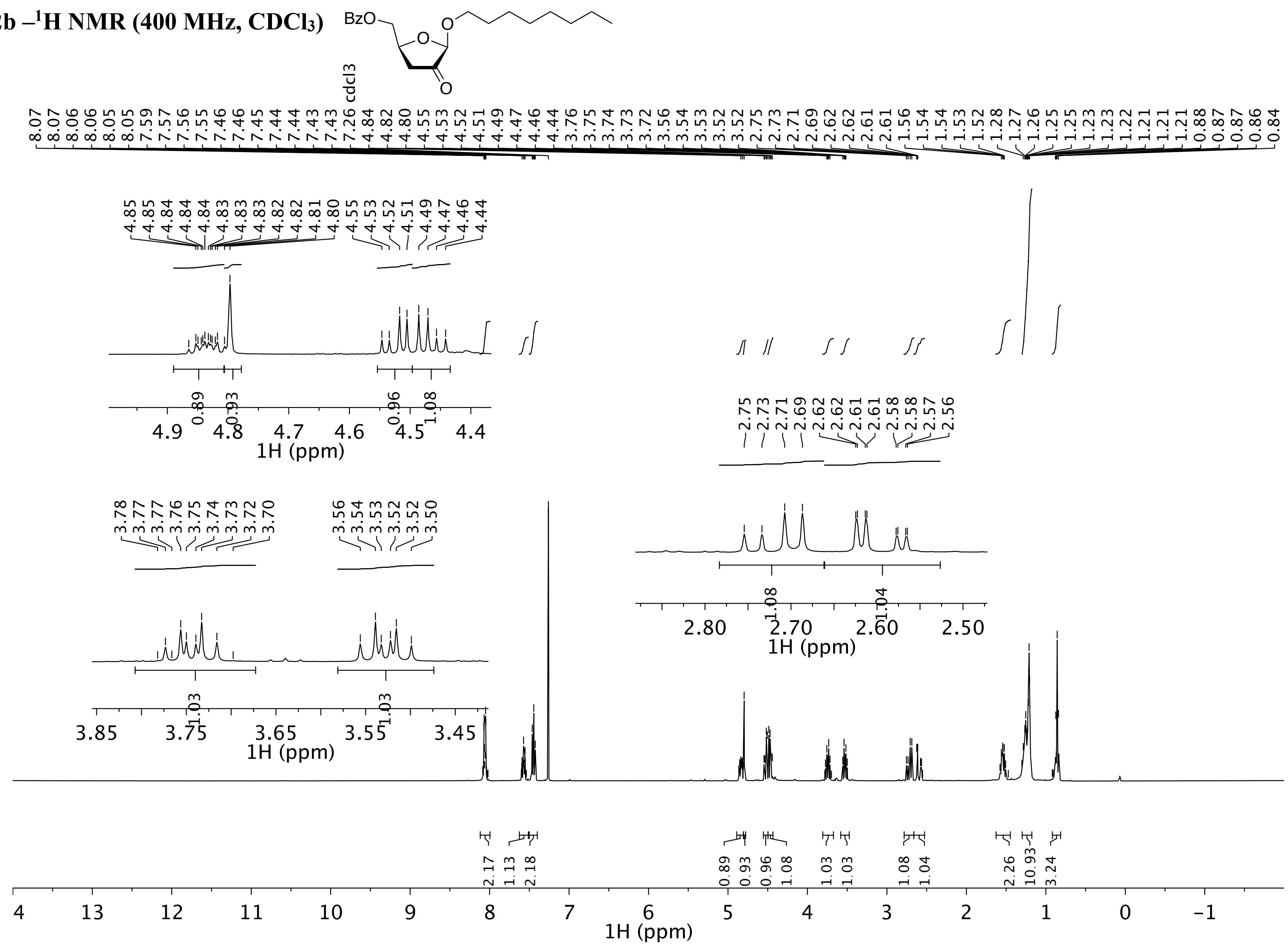

2b –^{13}C NMR (101 MHz, $CDCl_3$)

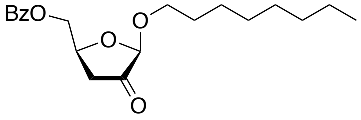

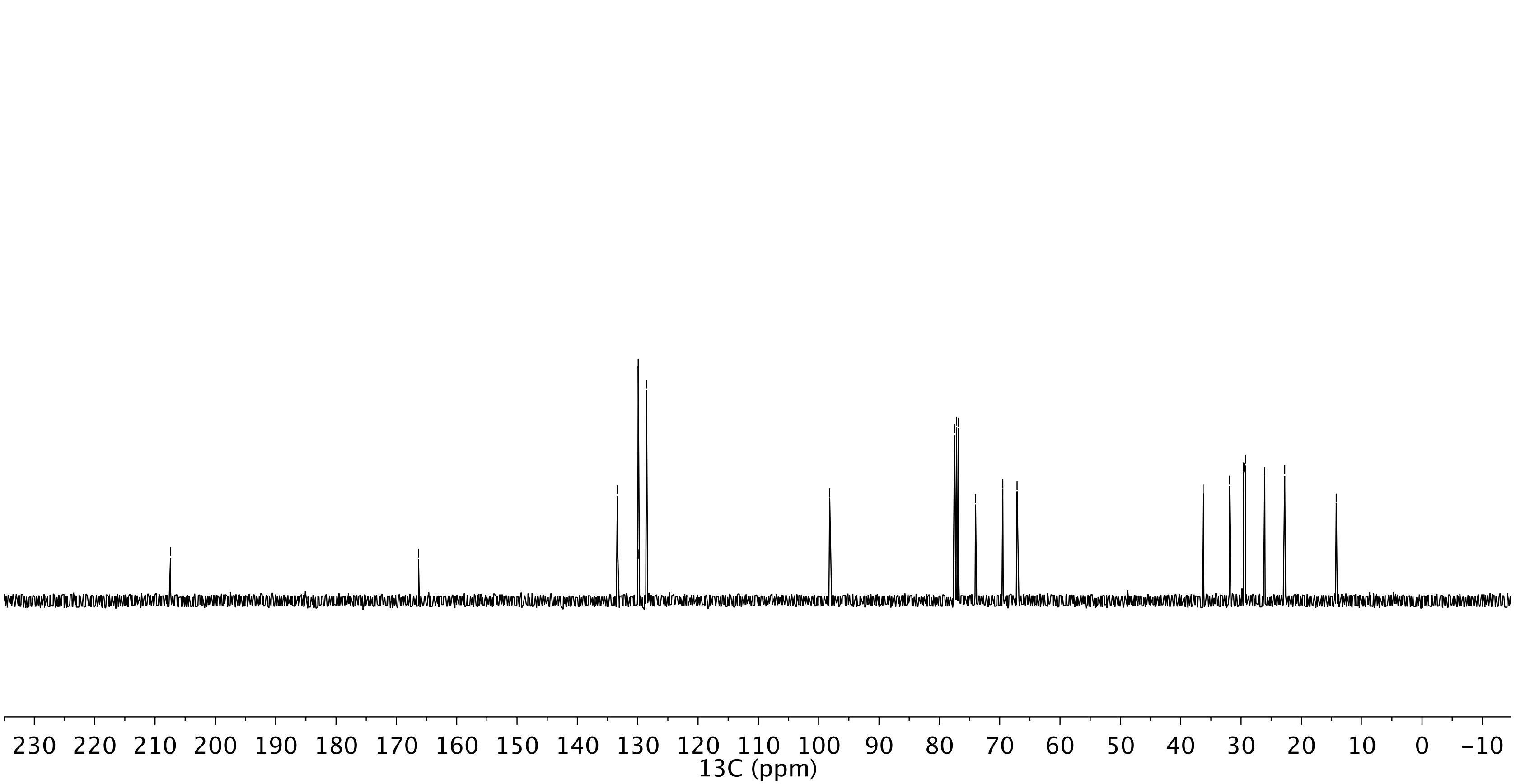

2b –COSY (400 MHz, CDCl3)

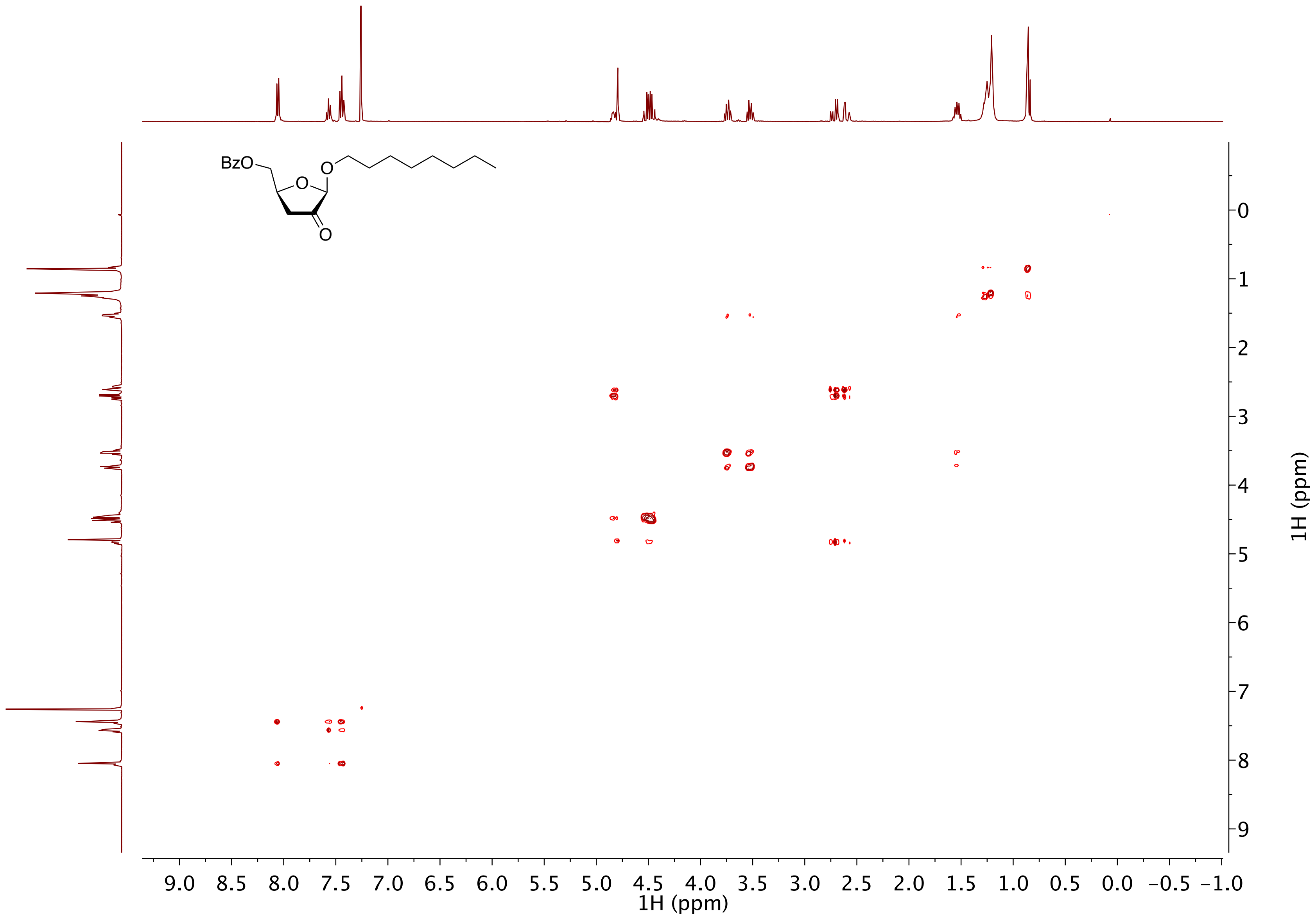

2b –HSQC (400 MHz, $CDCl_3$)

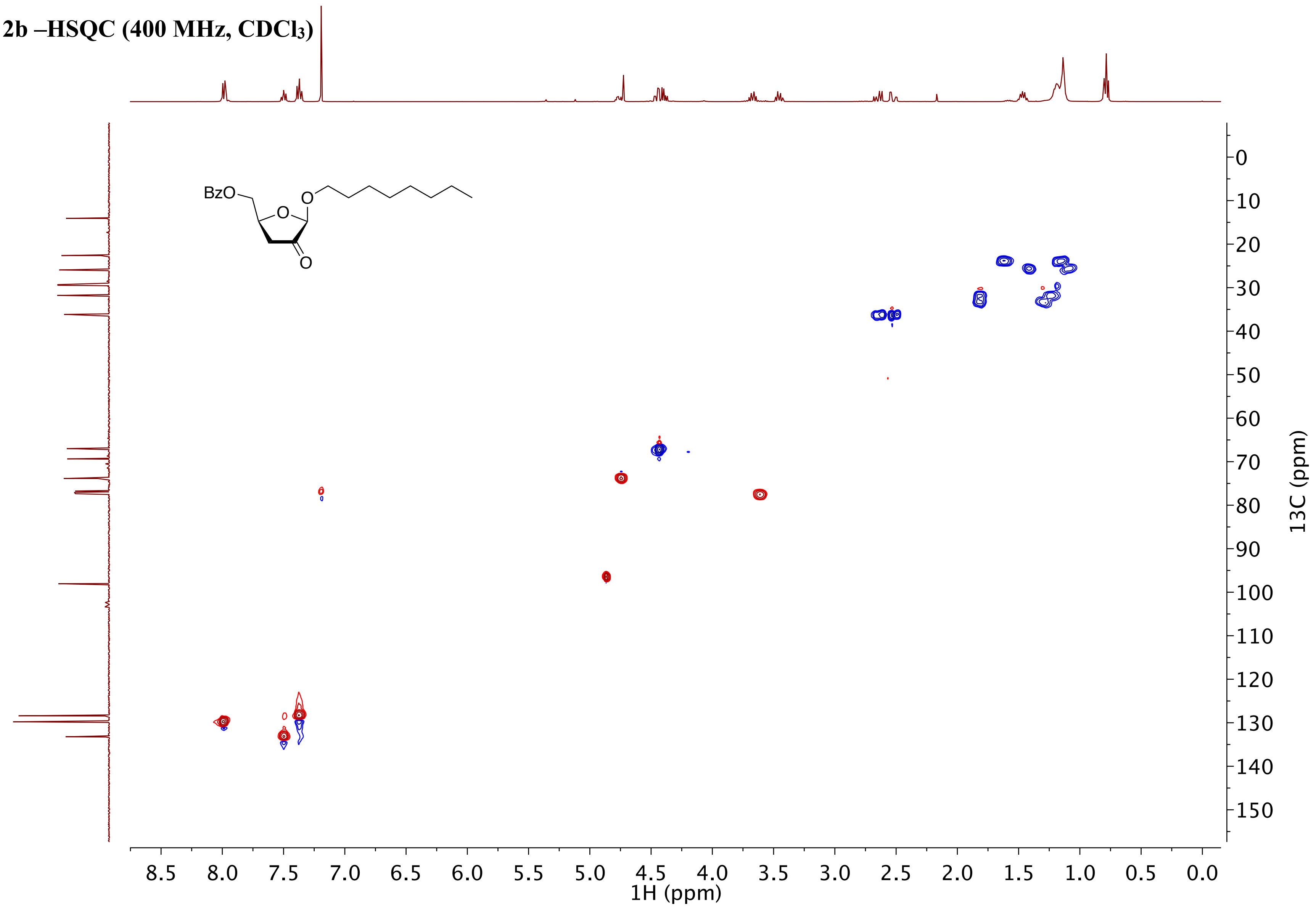

2b' –^{1}H NMR (400 MHz, CDCl₃)

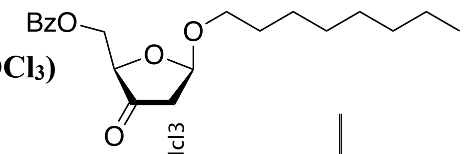

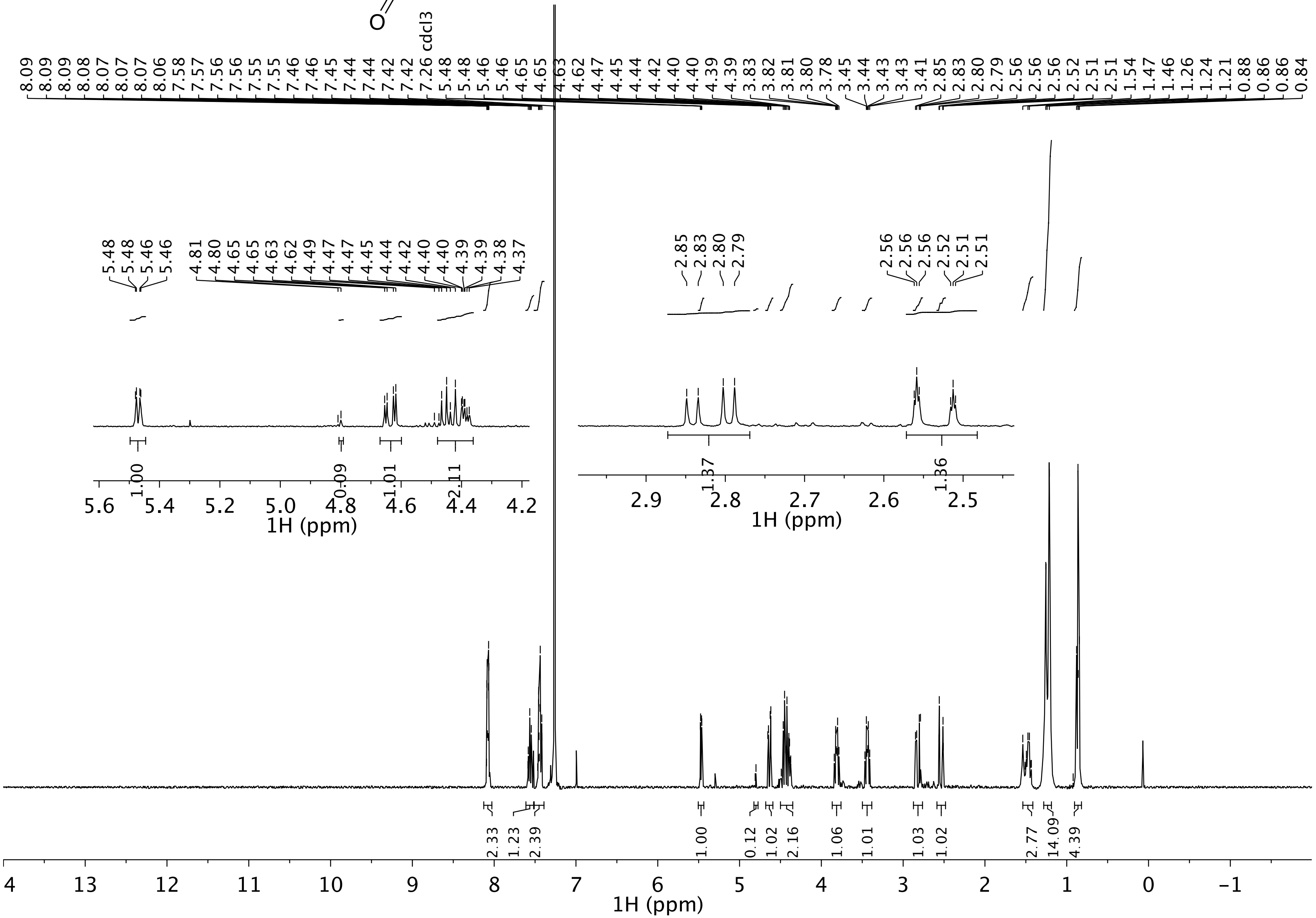

2b' – COSY (400 MHz, CDCl$_3$)

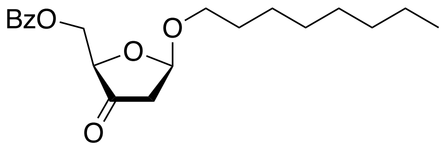

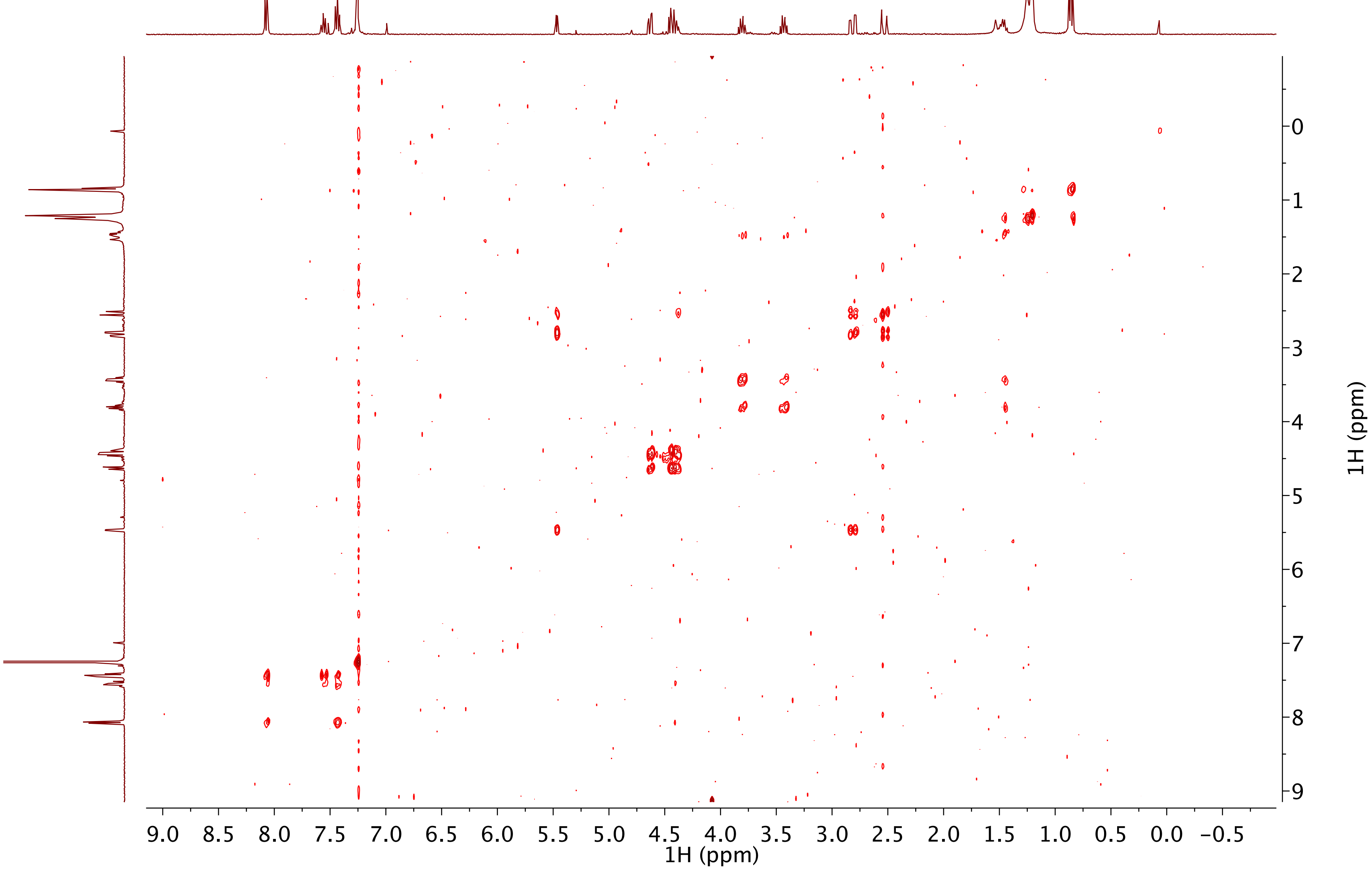

2c –^{1}H NMR (400 MHz, CDCl$_3$)

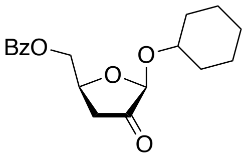

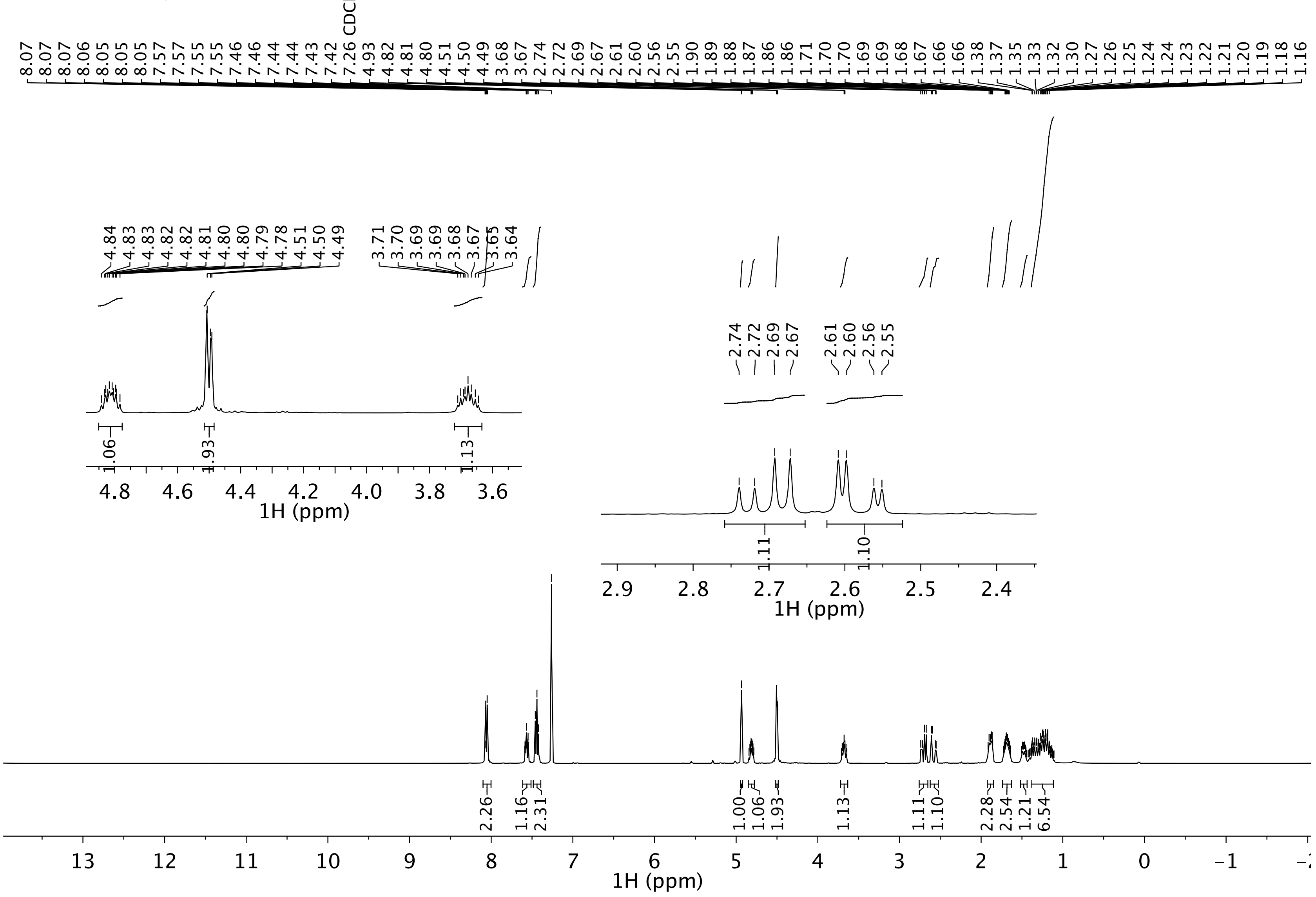

2c – ^{13}C NMR (101 MHz, CDCl$_3$)

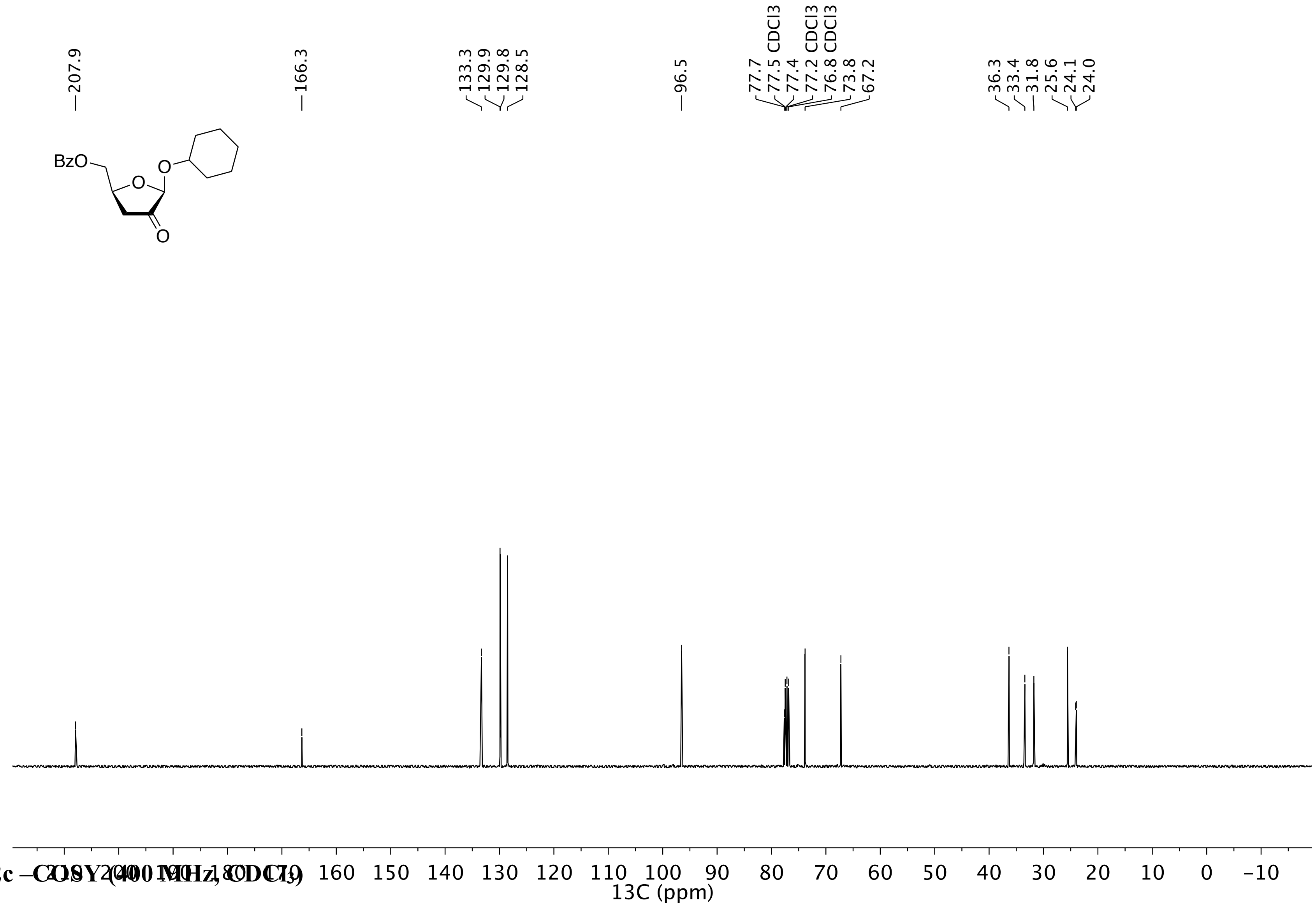

2c – COSY (400 MHz, CDCl$_3$)

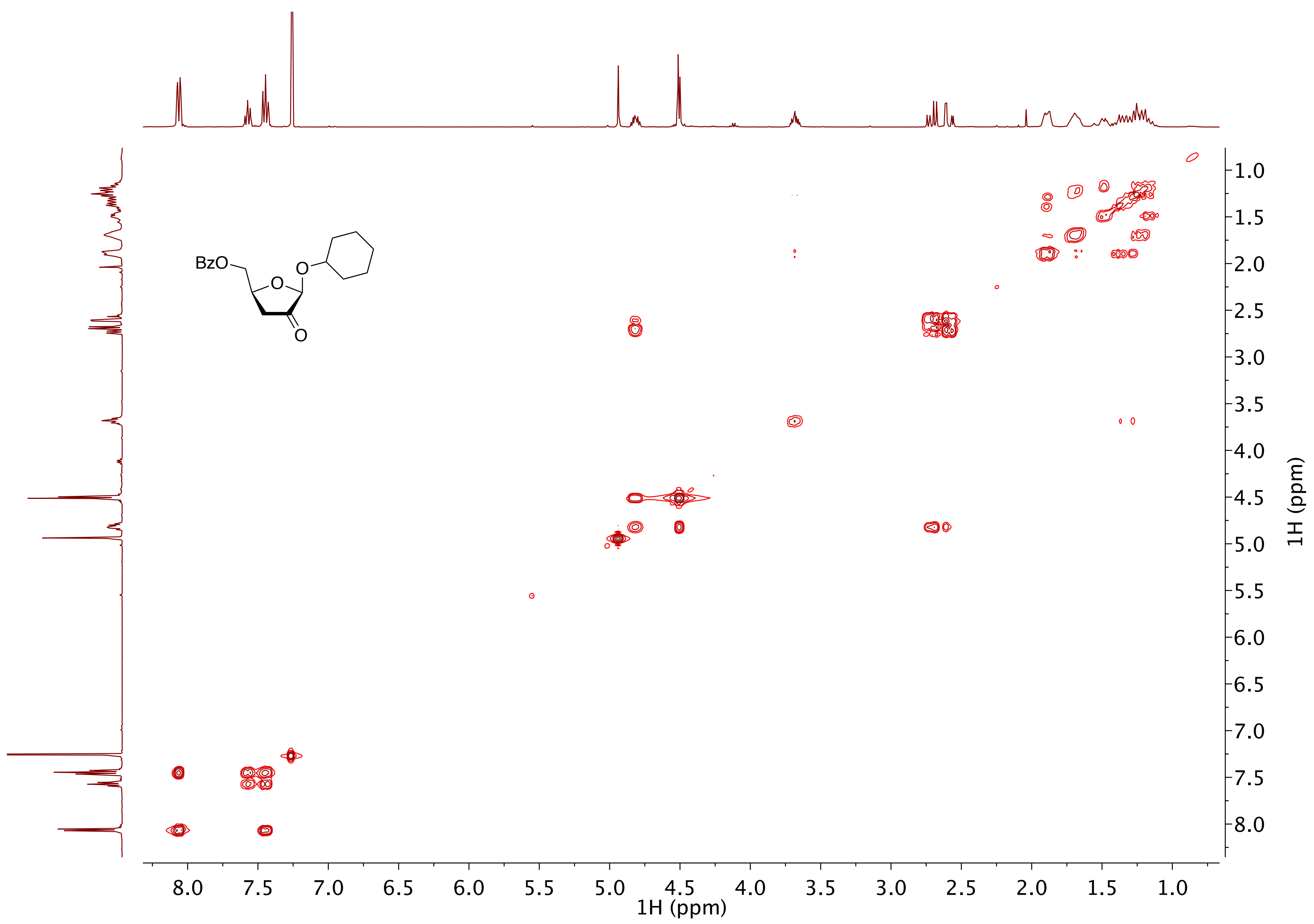
BzO
O
O
O
8.0
7.5
7.0
6.5
6.0
5.5
5.0
4.5
4.0
3.5
3.0
2.5
2.0
1.5
1.0
1H (ppm)
1H (ppm)

2c –HSQC (400 MHz, CDCl₃)

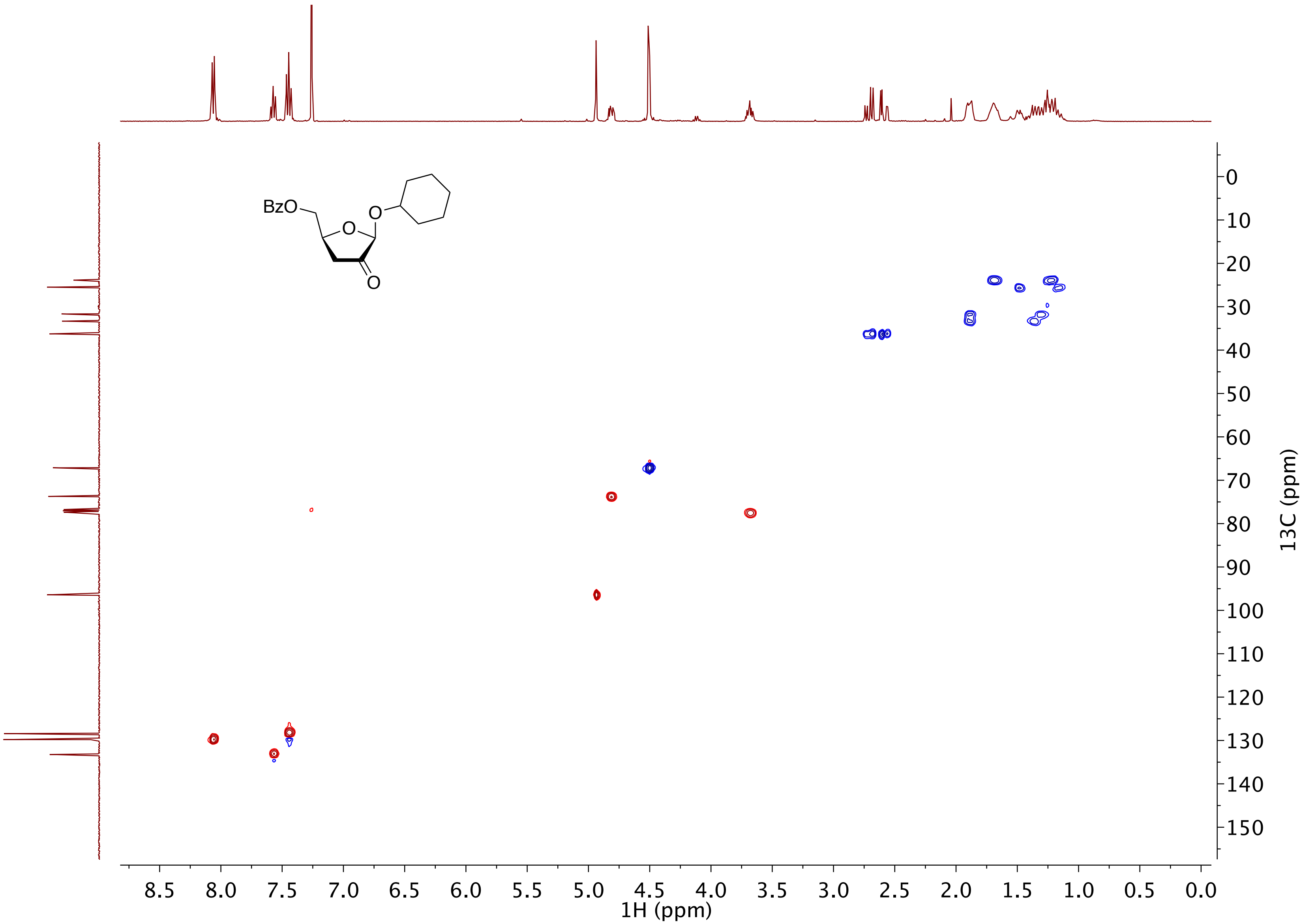

2d –^{1}H NMR (400 MHz, CDCl₃)

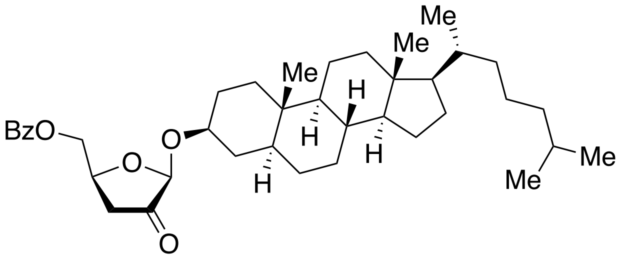

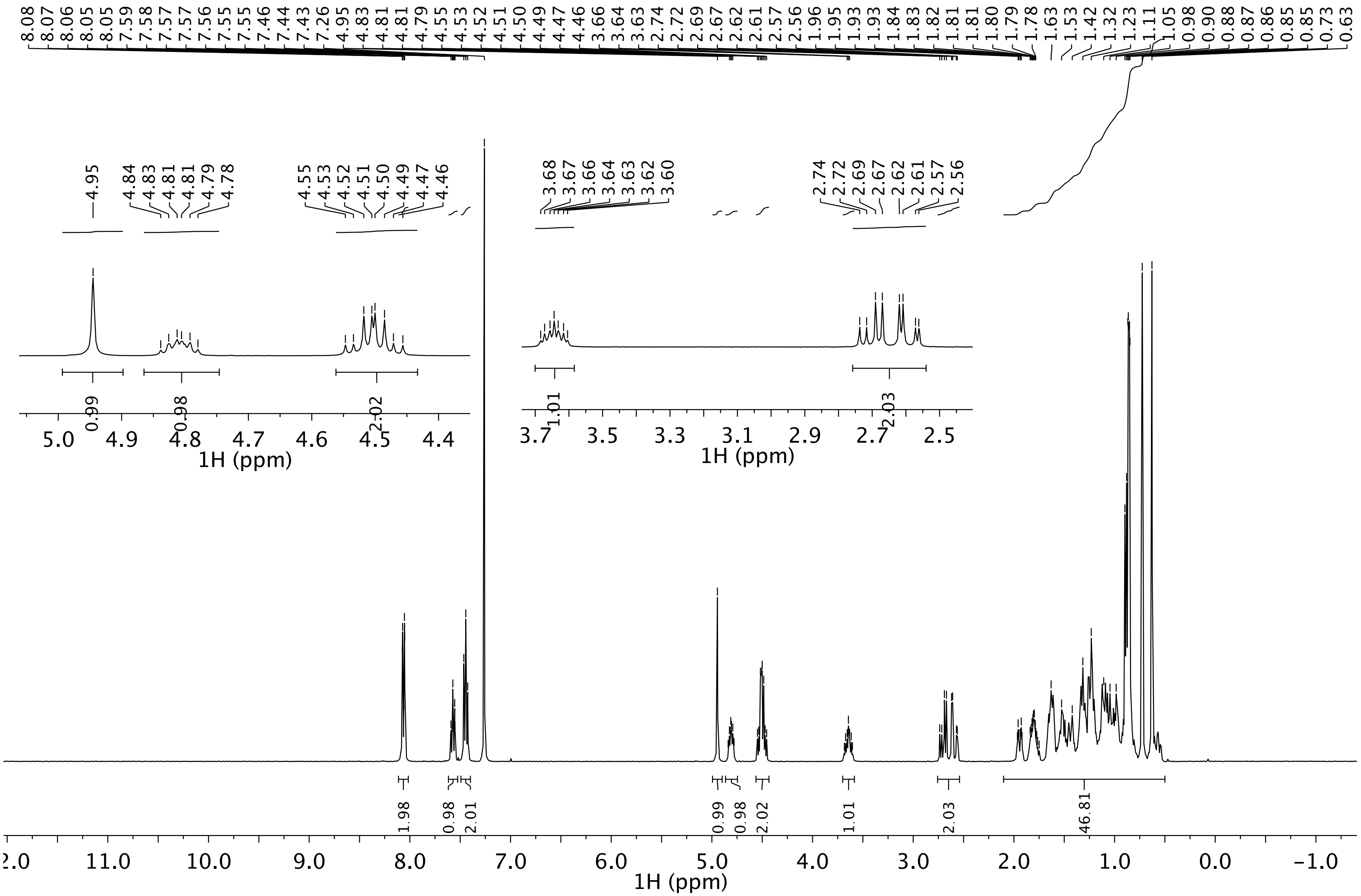

2d –^{13}C NMR (101 MHz, CDCl$_3$)

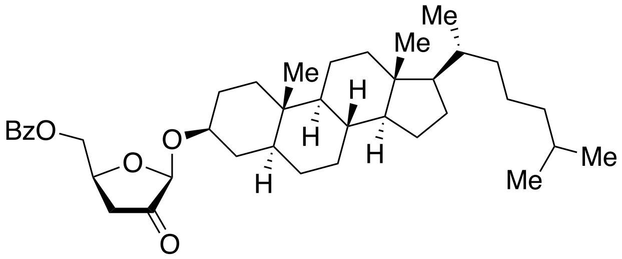

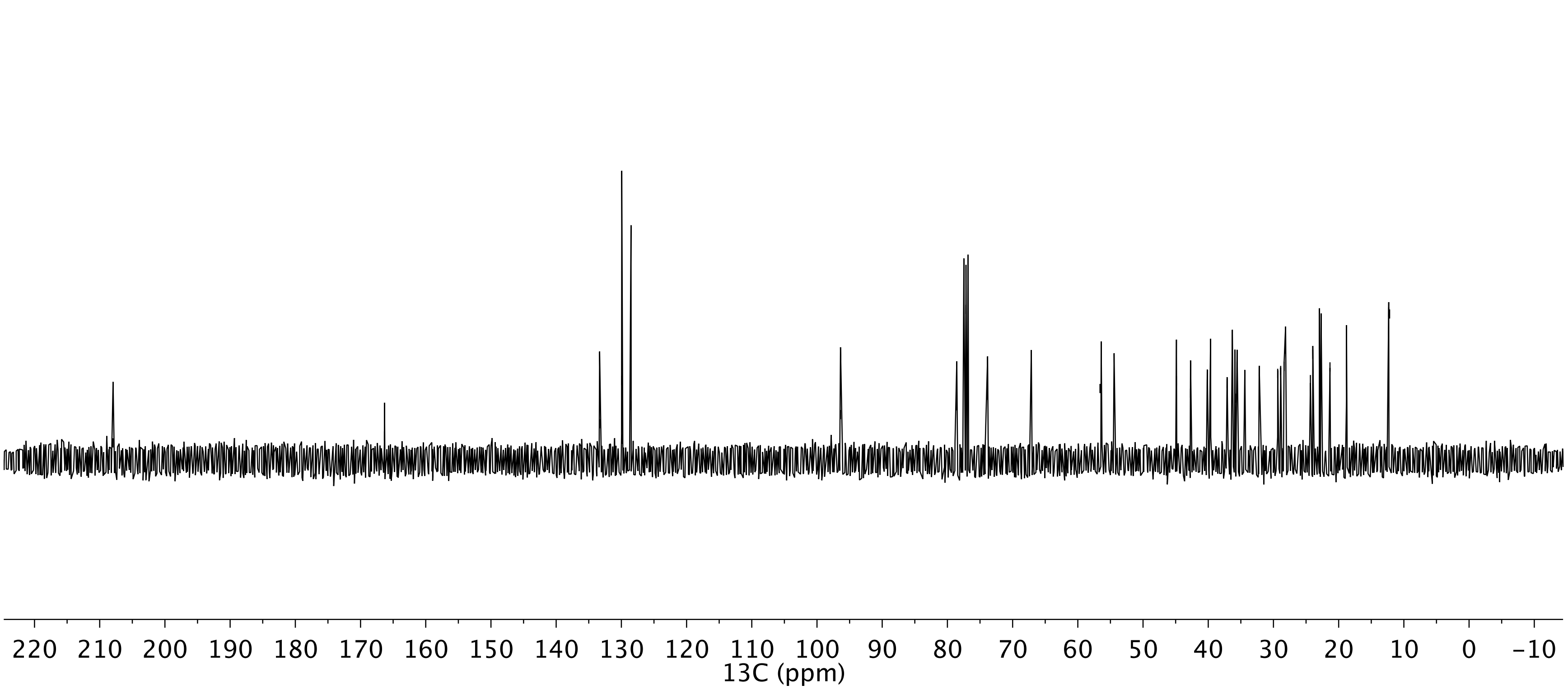

2d – COSY (400 MHz, CDCl₃)

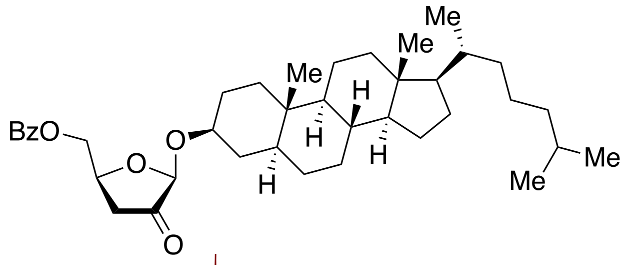

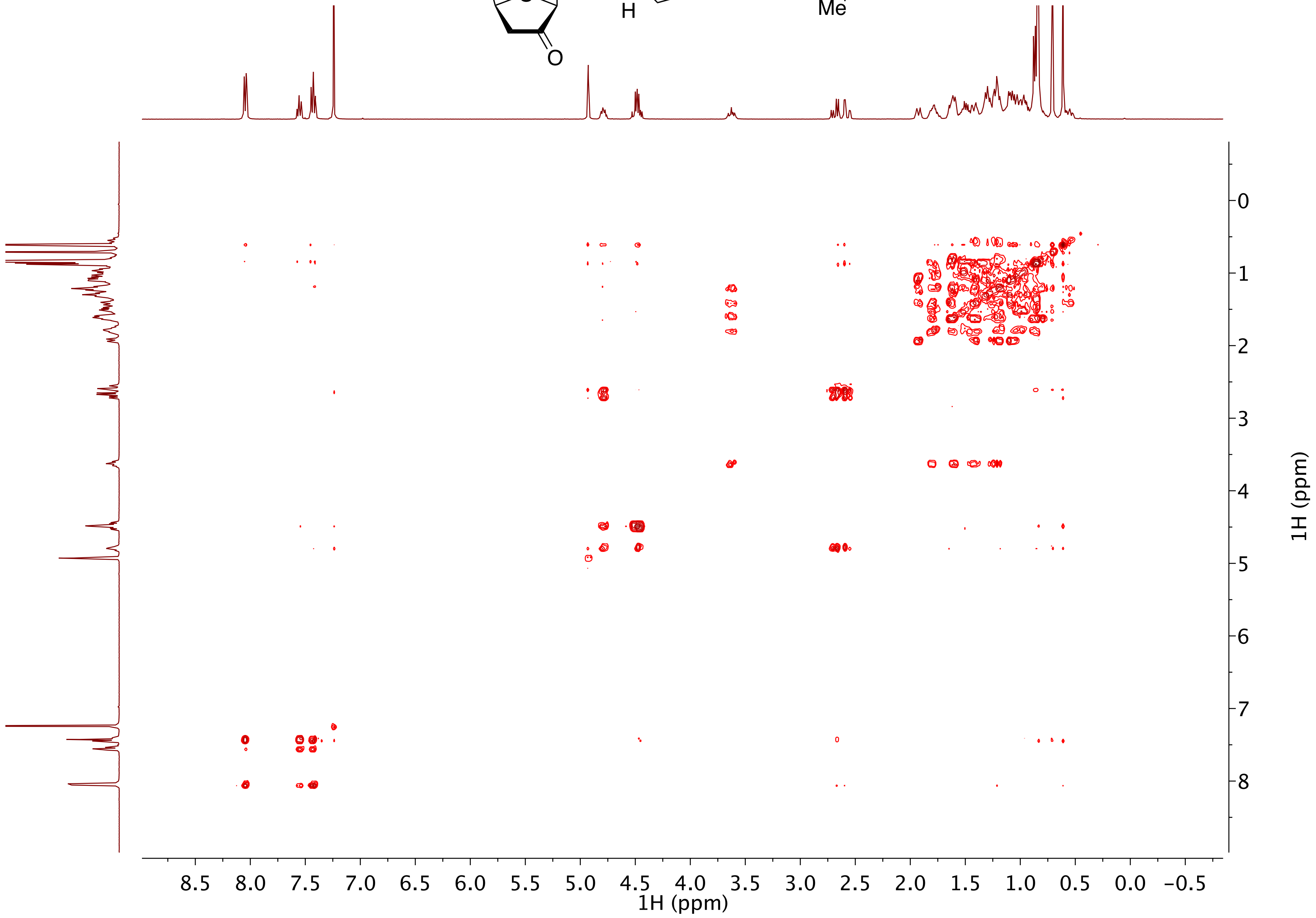

2d – HSQC (400 MHz, CDCl3)

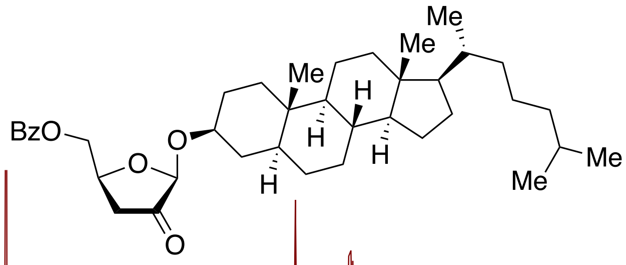

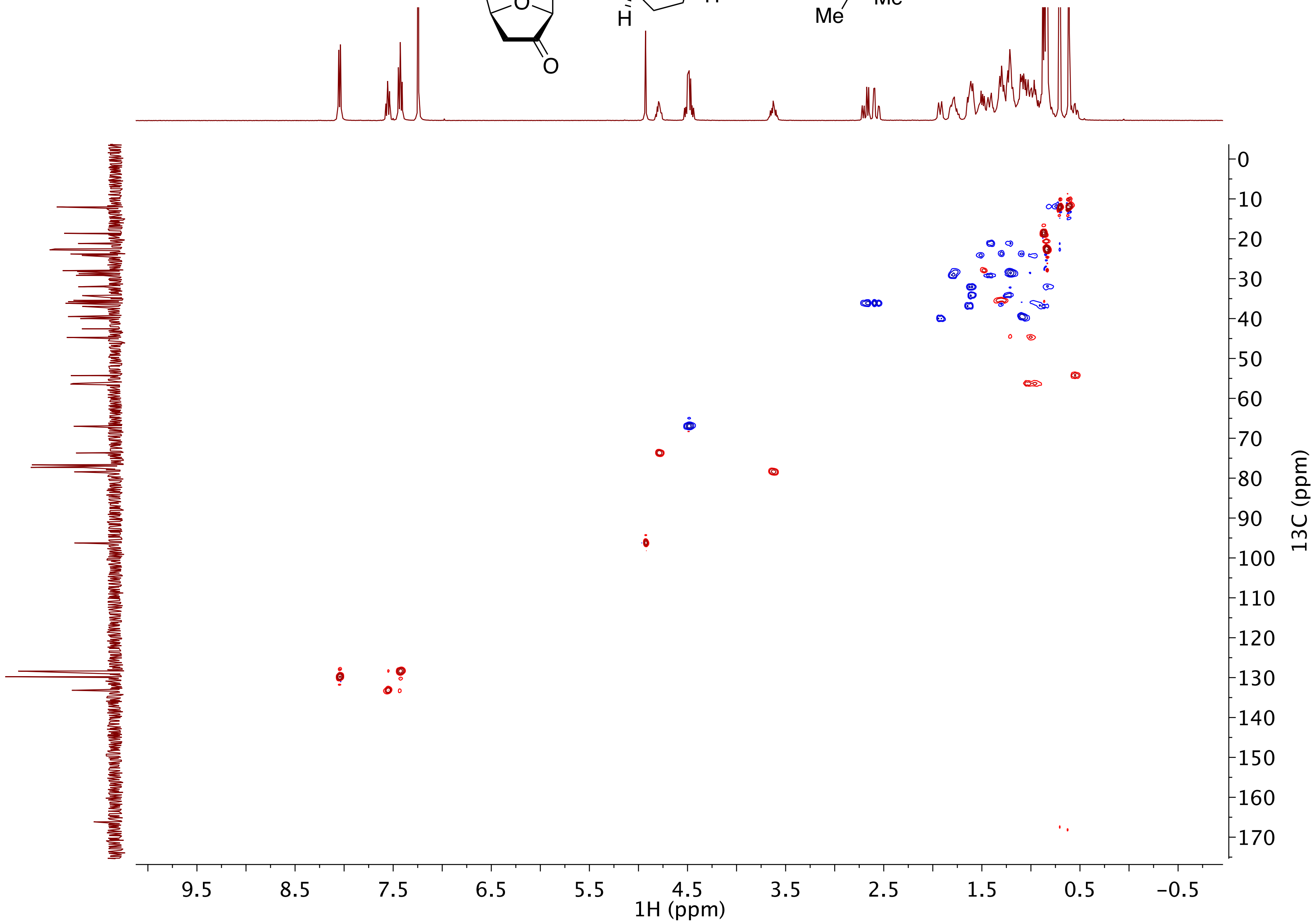

2e –^{1}H NMR (400 MHz, $CDCl_3$)

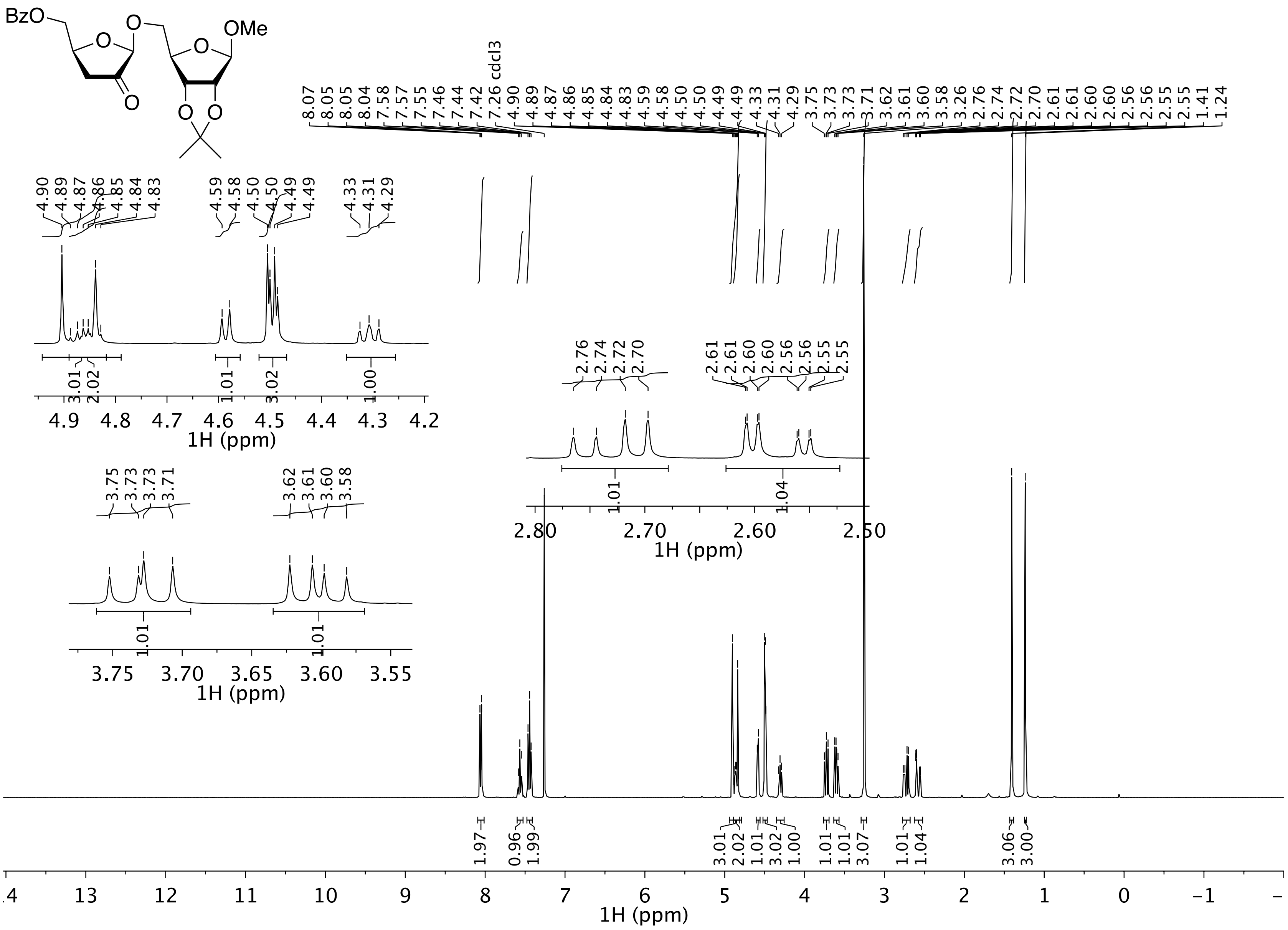

2e –^{13}C NMR (101 MHz, $CDCl_3$)

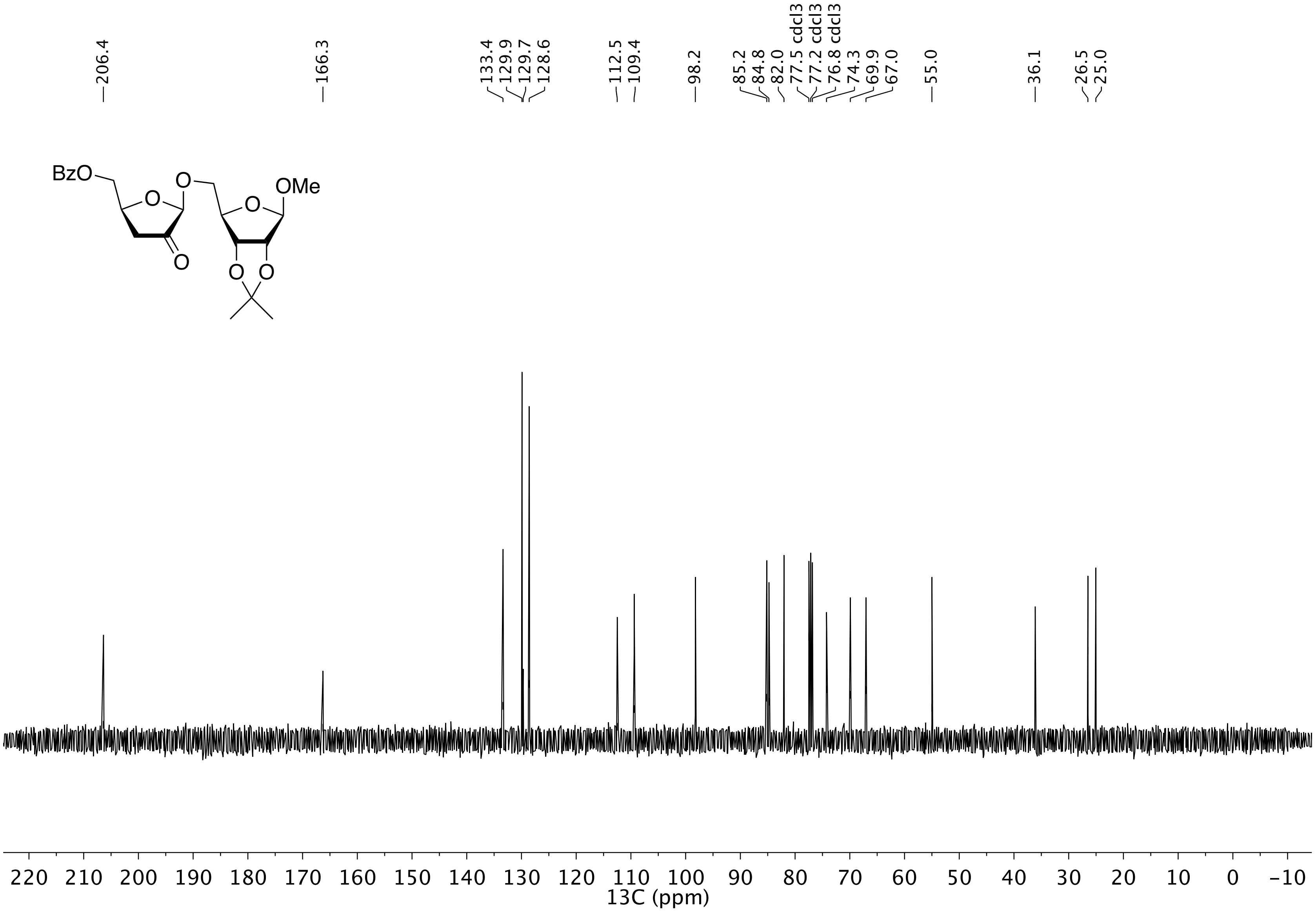

2e –COSY (400 MHz, CDCl₃)

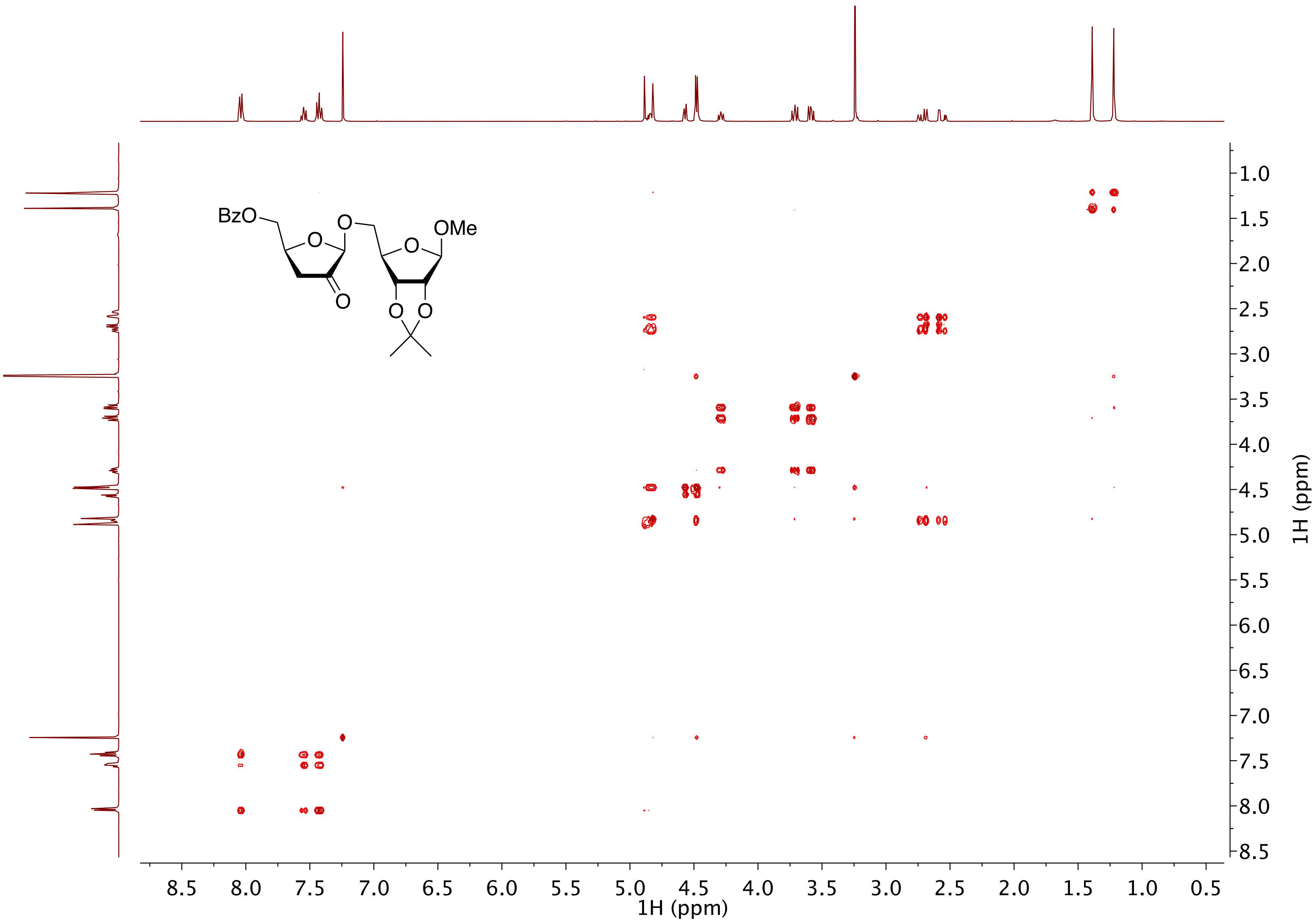

2e –HSQC (400 MHz, $CDCl_3$)

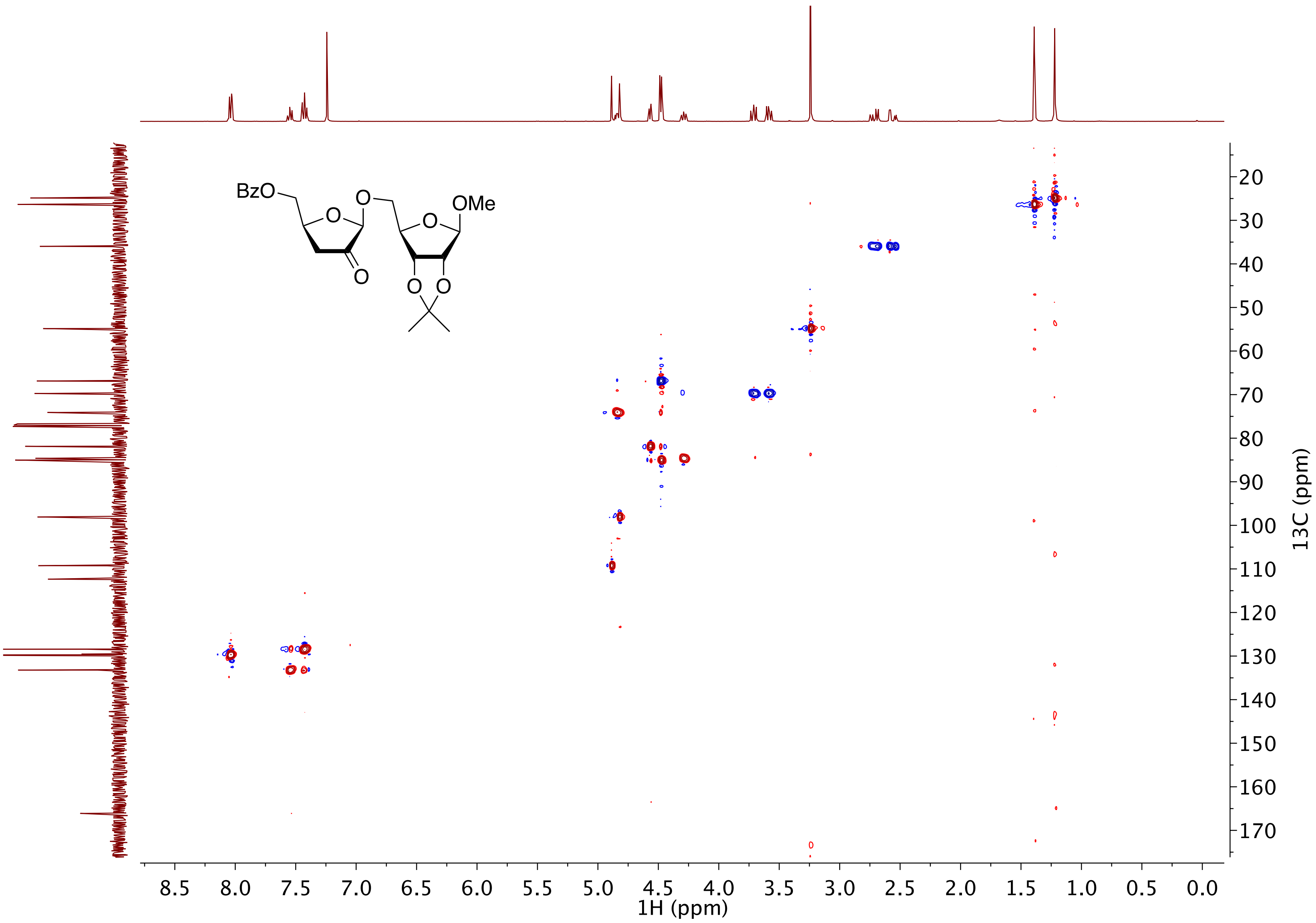

2f –^{1}H NMR (400 MHz, CDCl3)

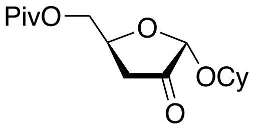

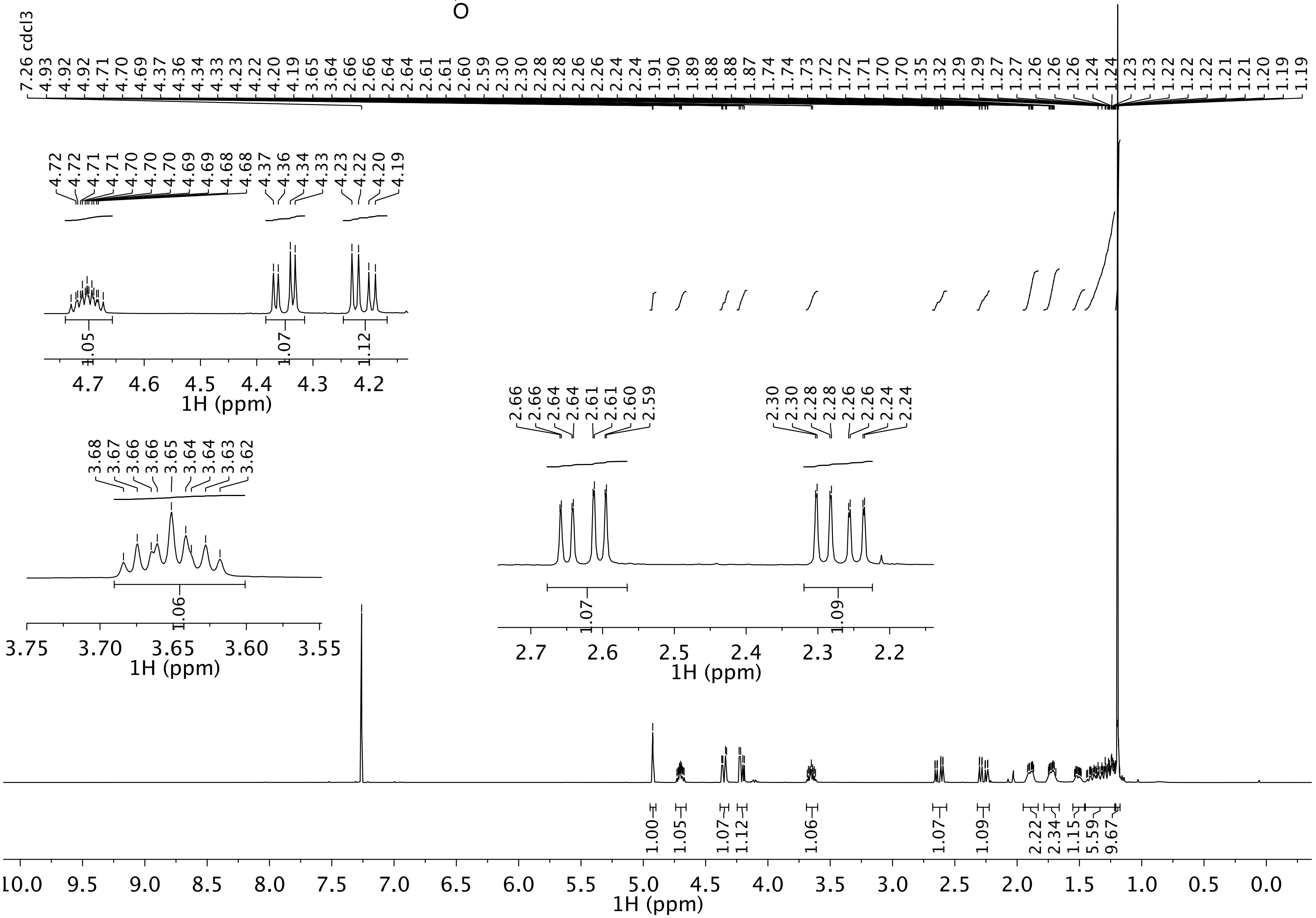

2f – ^{13}C NMR (101 MHz, $CDCl_3$)

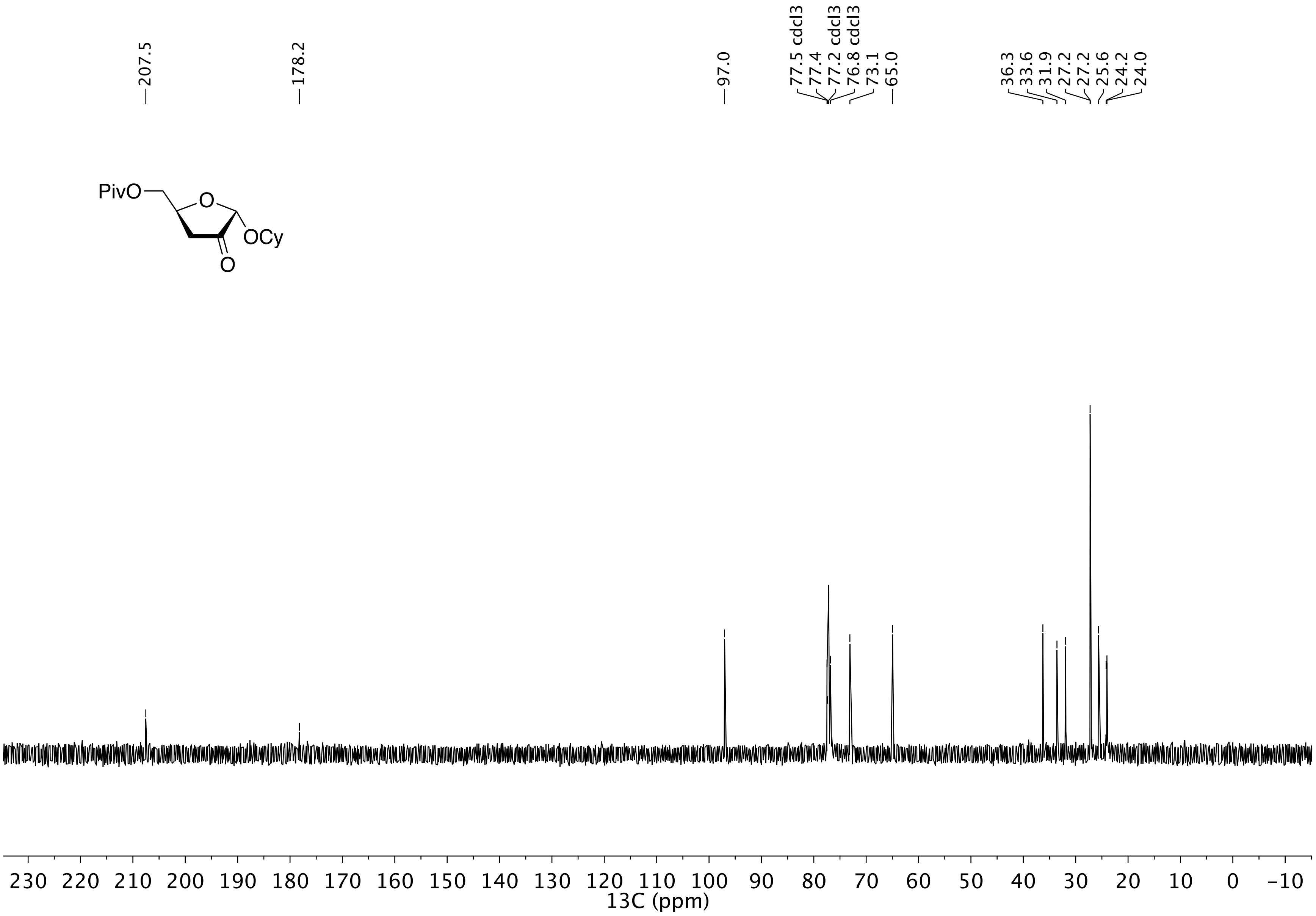

2f –COSY (400 MHz, $CDCl_3$)

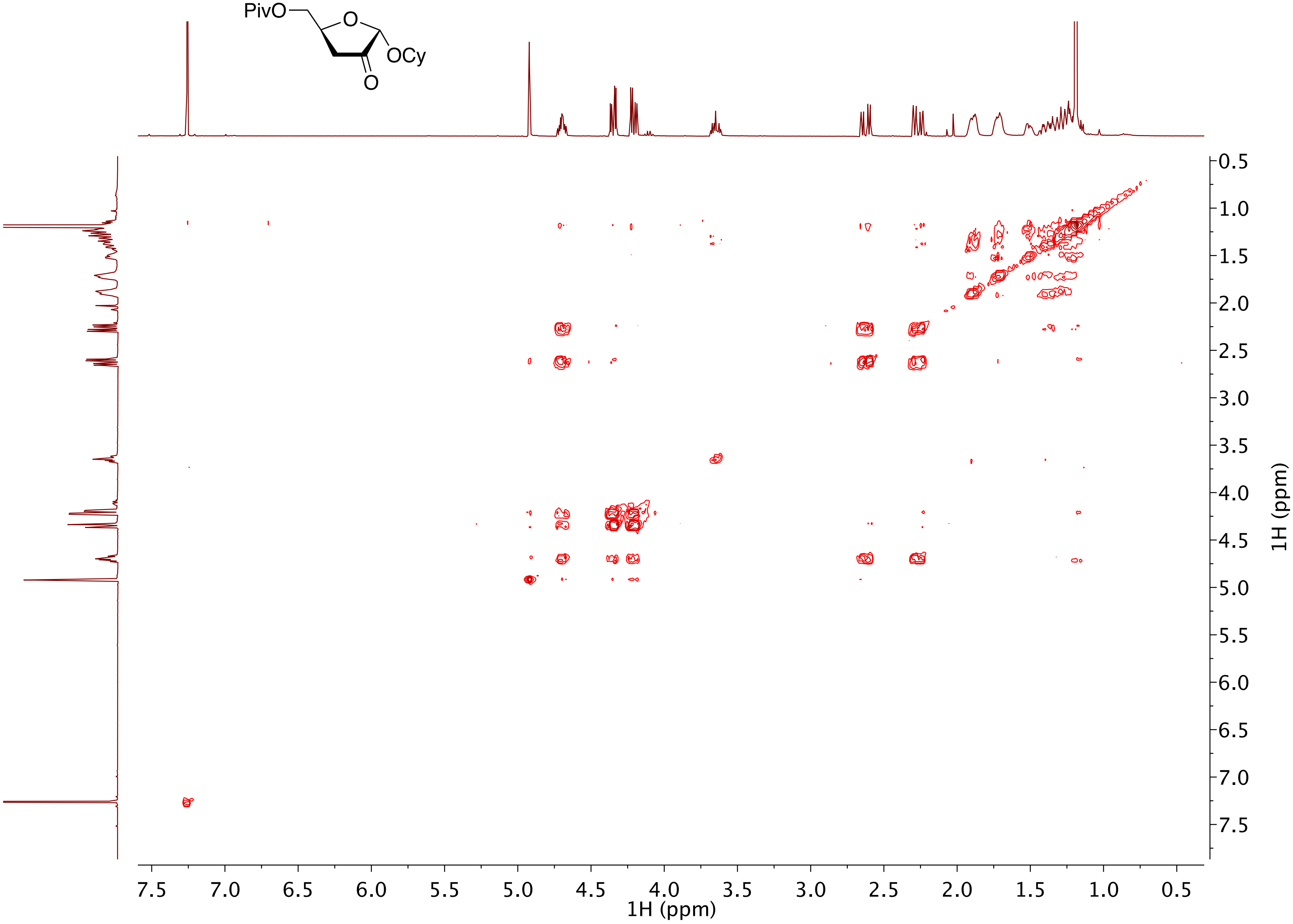

2f –HSQC (400 MHz, CDCl₃)

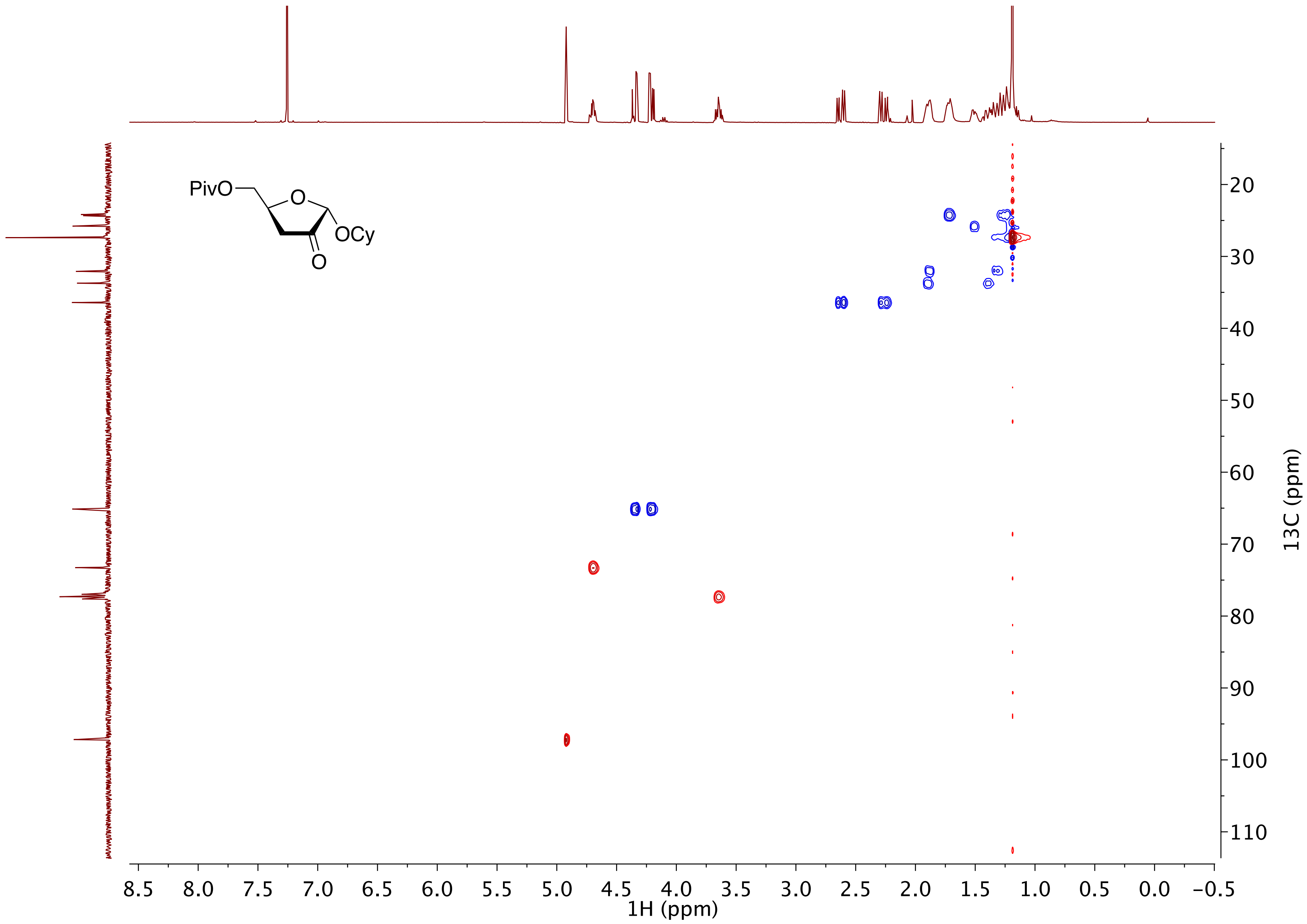

2f' –^{1}H NMR (400 MHz, CDCl₃)

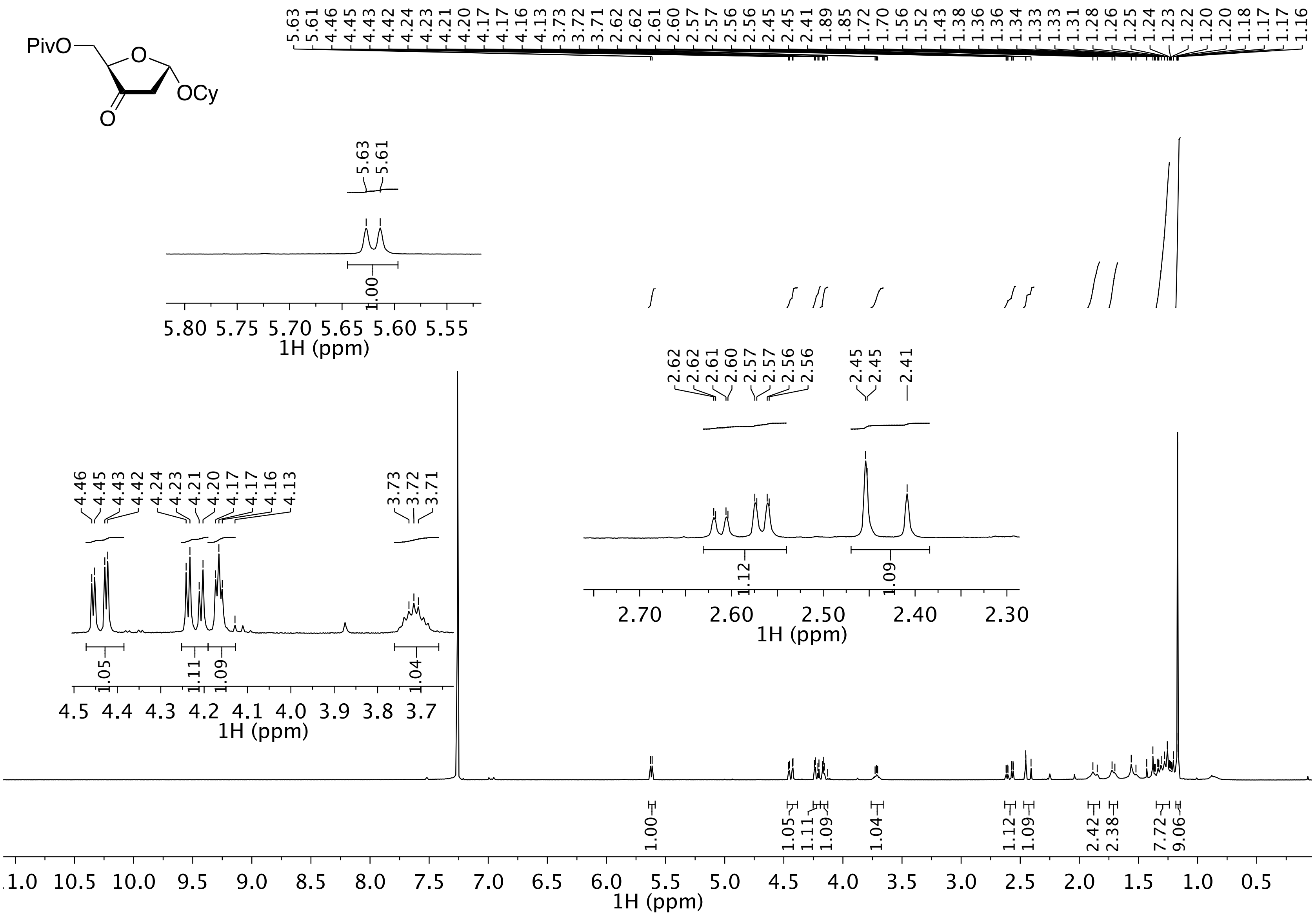

2f’ –^{13}C NMR (126 MHz, CDCl$_3$)

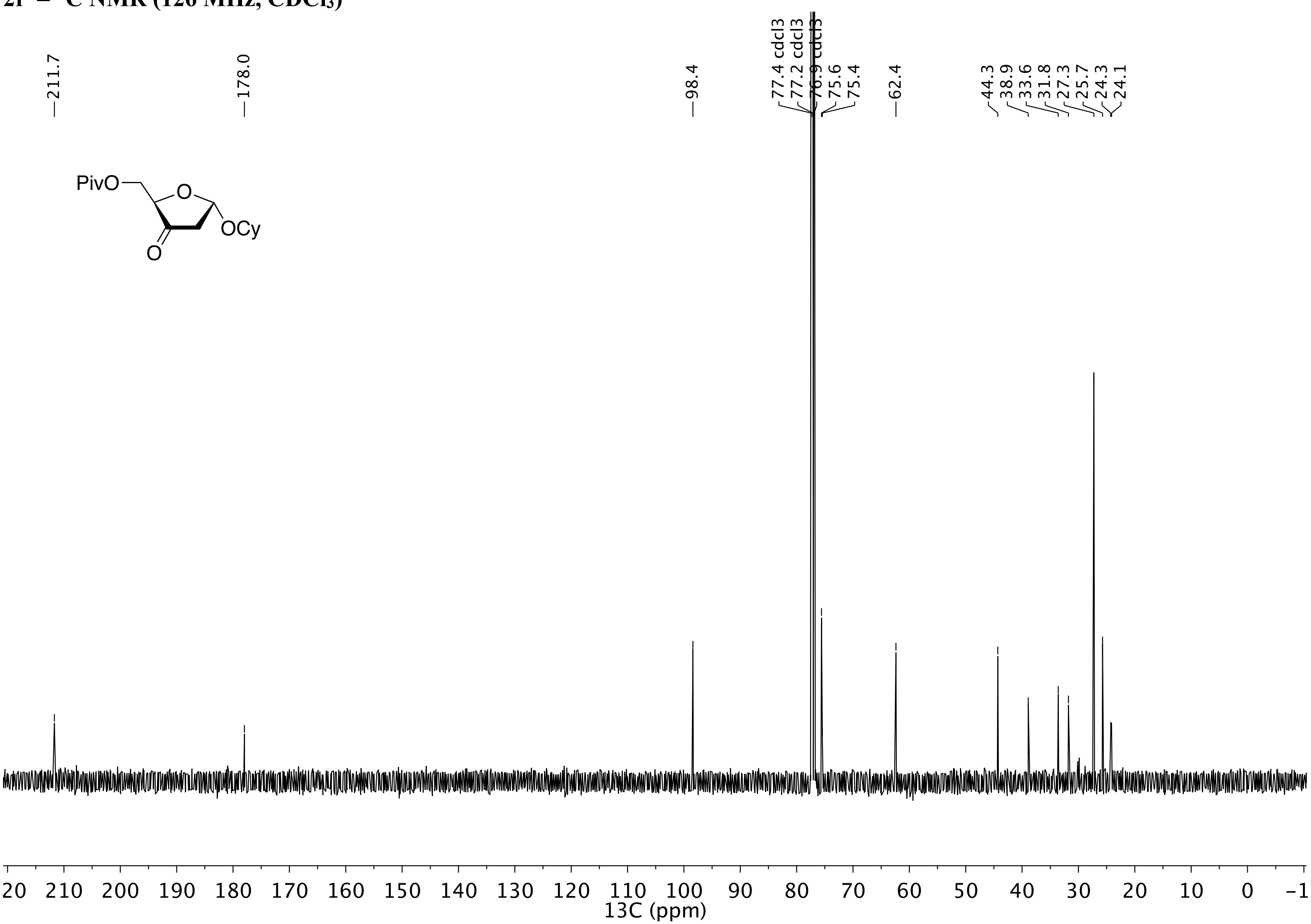

2f' – COSY (400 MHz, CDCl3)

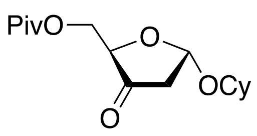

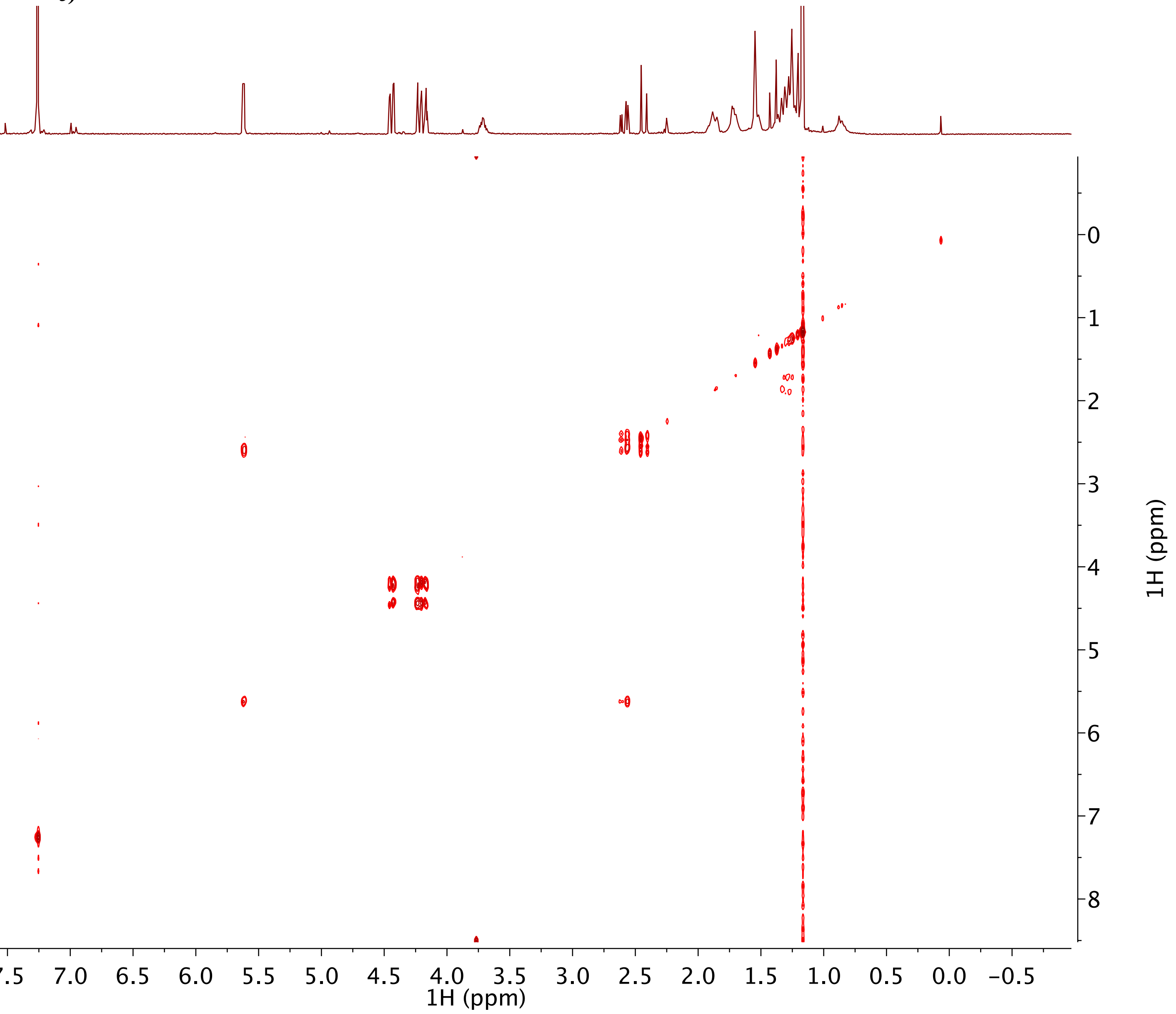

2f' – HSQC (400 MHz, CDCl₃)

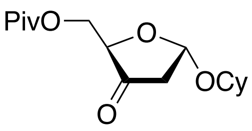

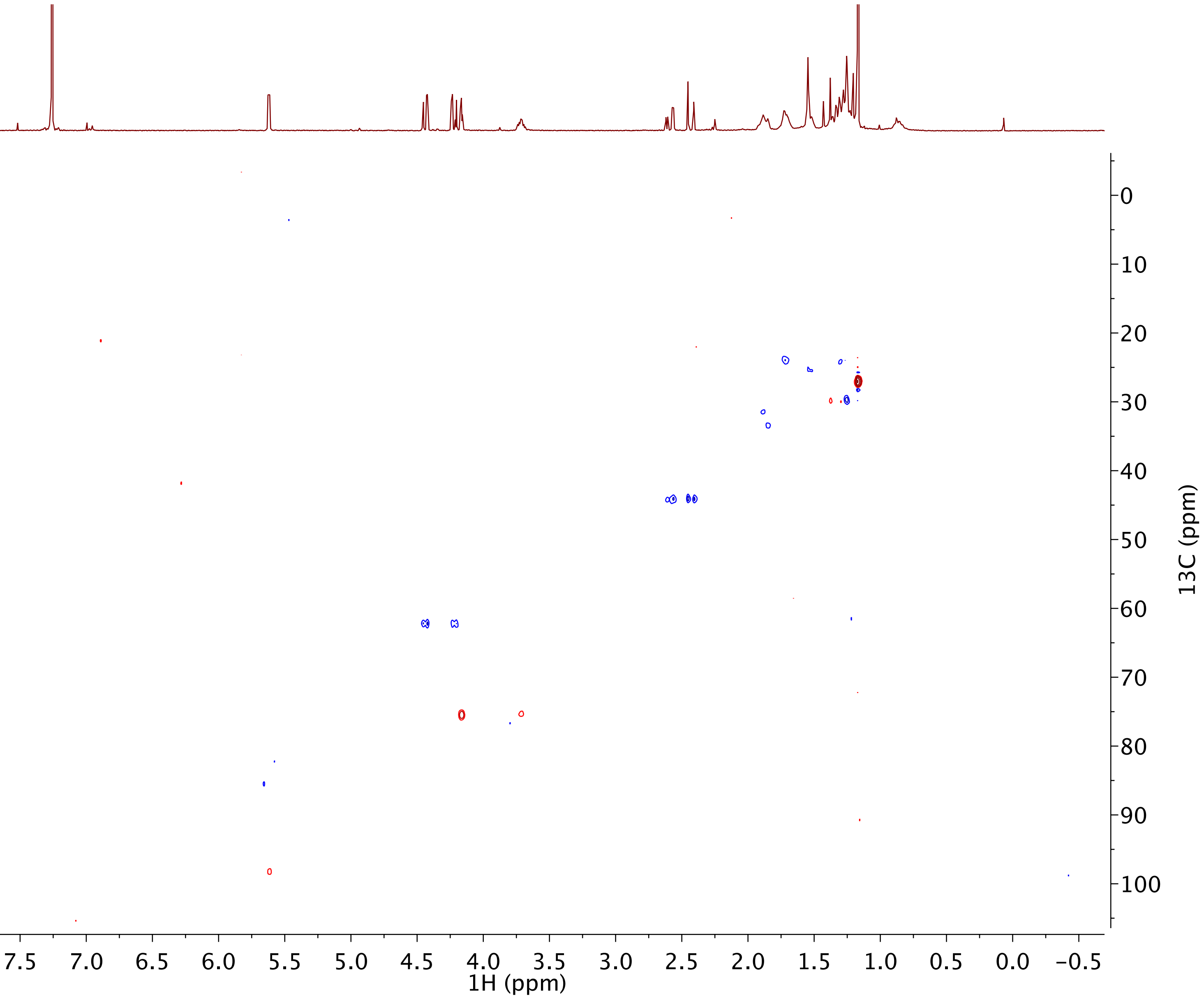

2g –^{1}H NMR (400 MHz, CDCl₃)

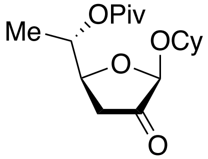

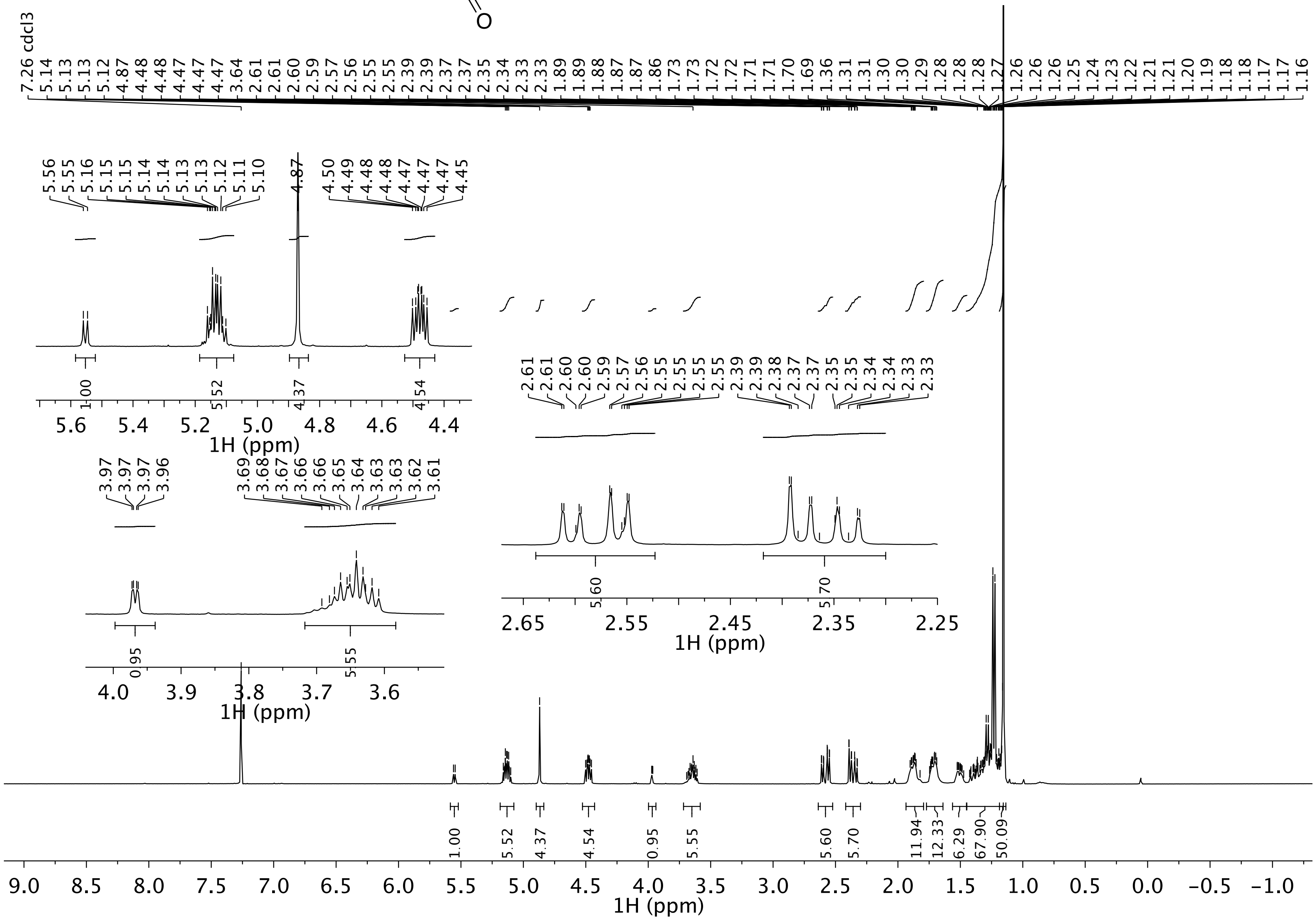

2g –^{13}C NMR (101 MHz, $CDCl_3$)

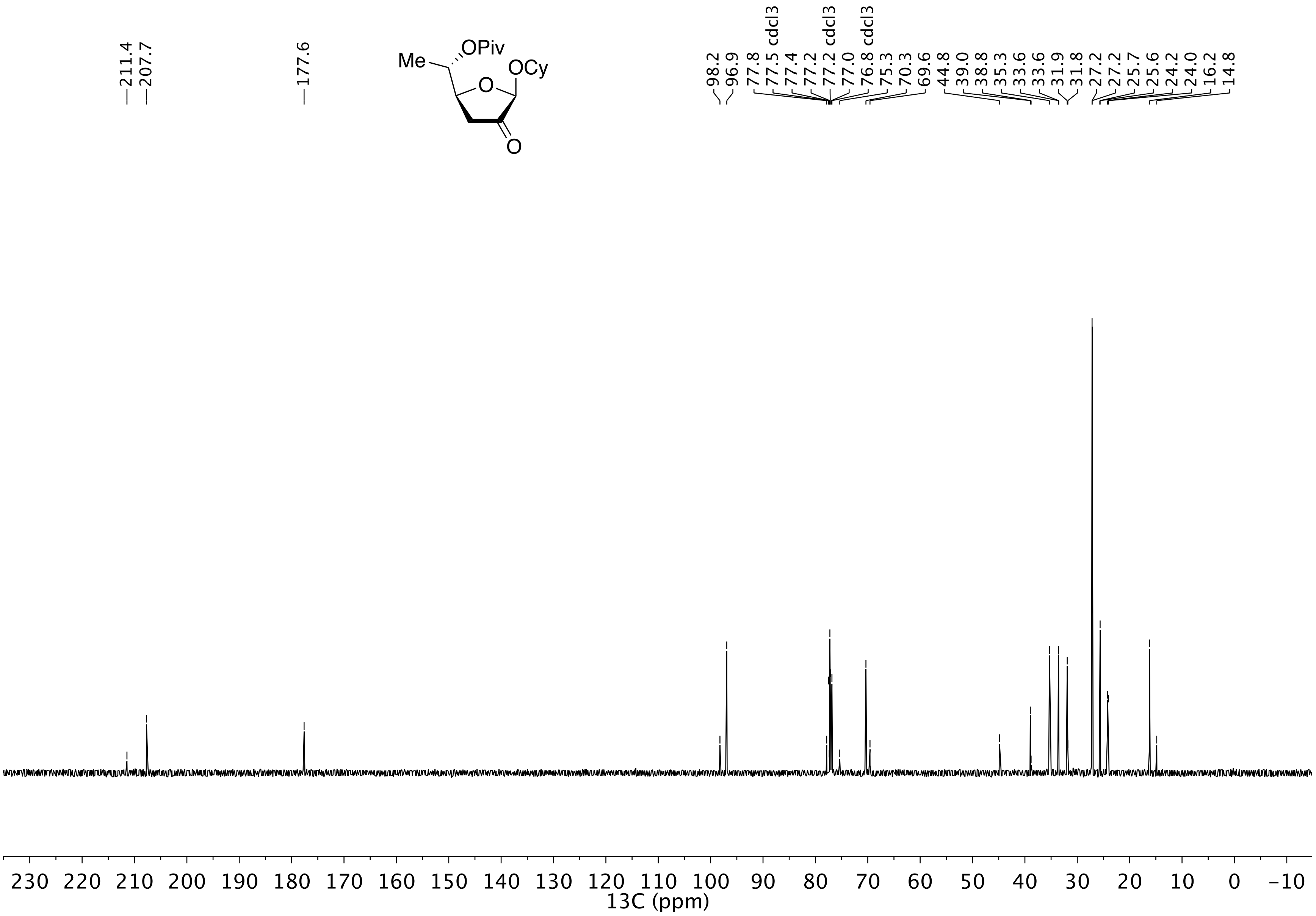

2g –COSY (400 MHz, CDCl$_3$)

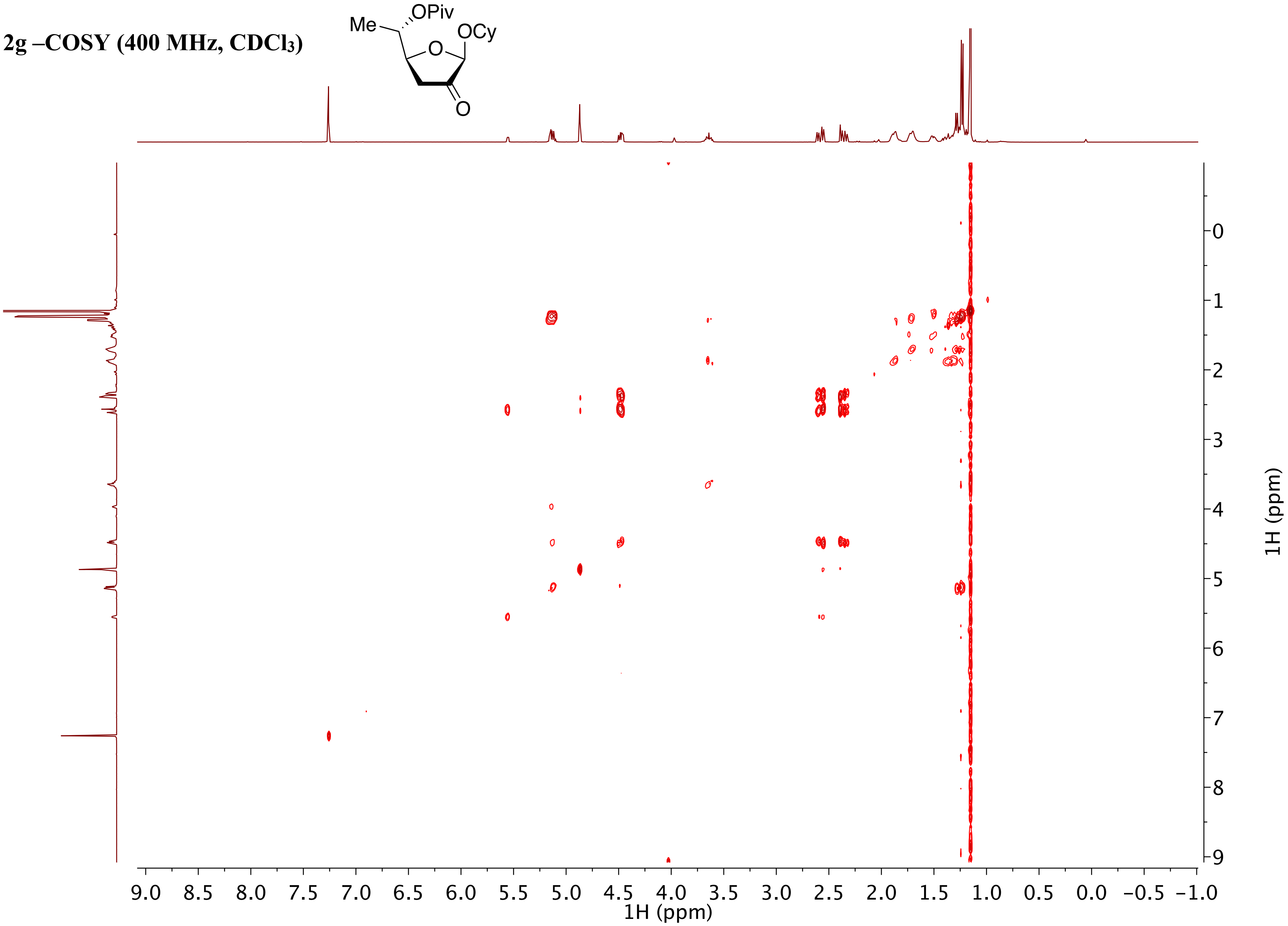

2g –HSQC (400 MHz, $CDCl_3$)

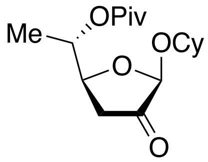

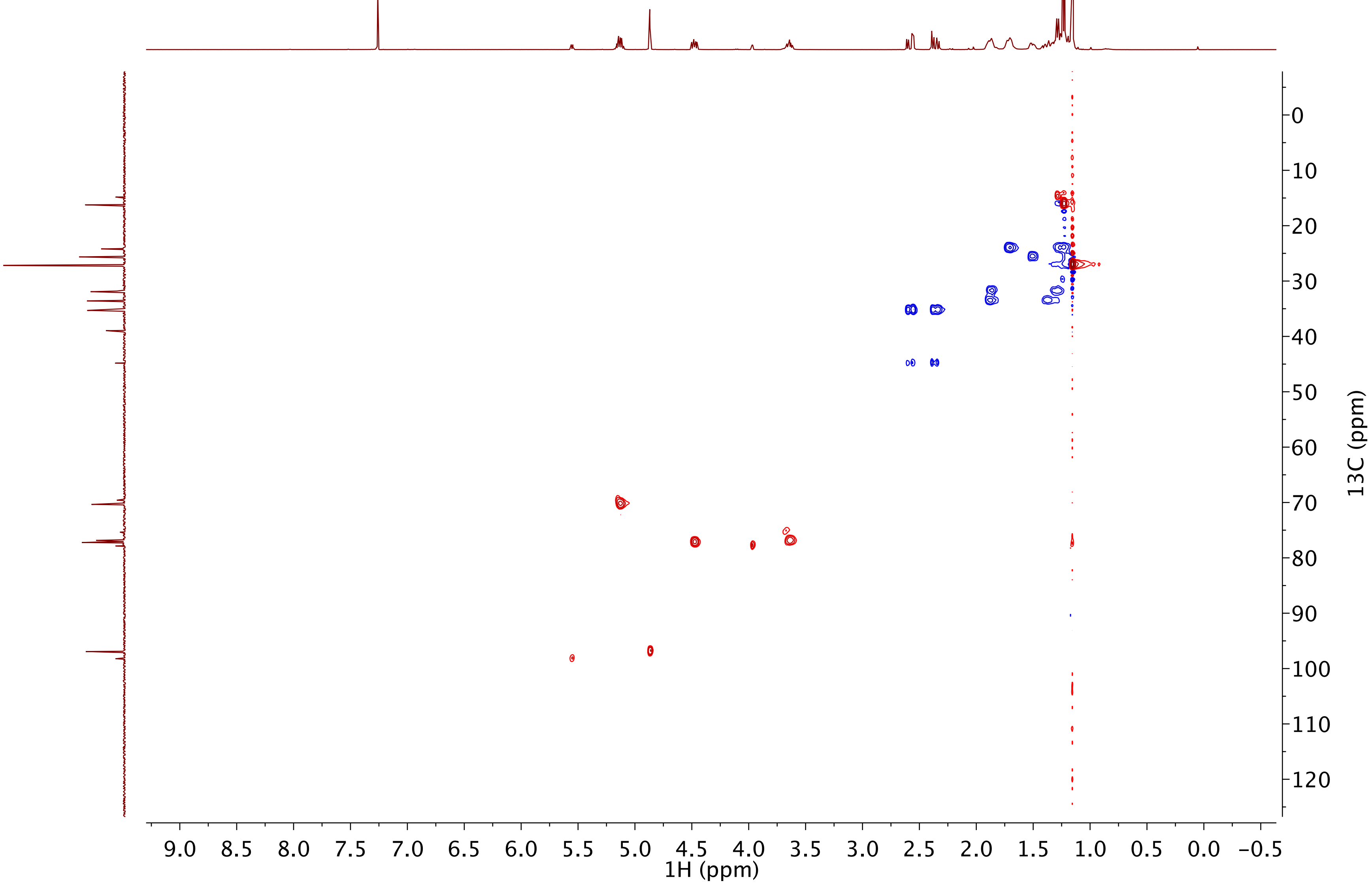

2h –^{1}H NMR (400 MHz, CDCl₃)

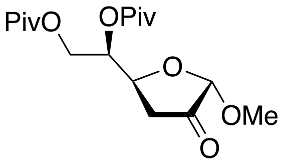

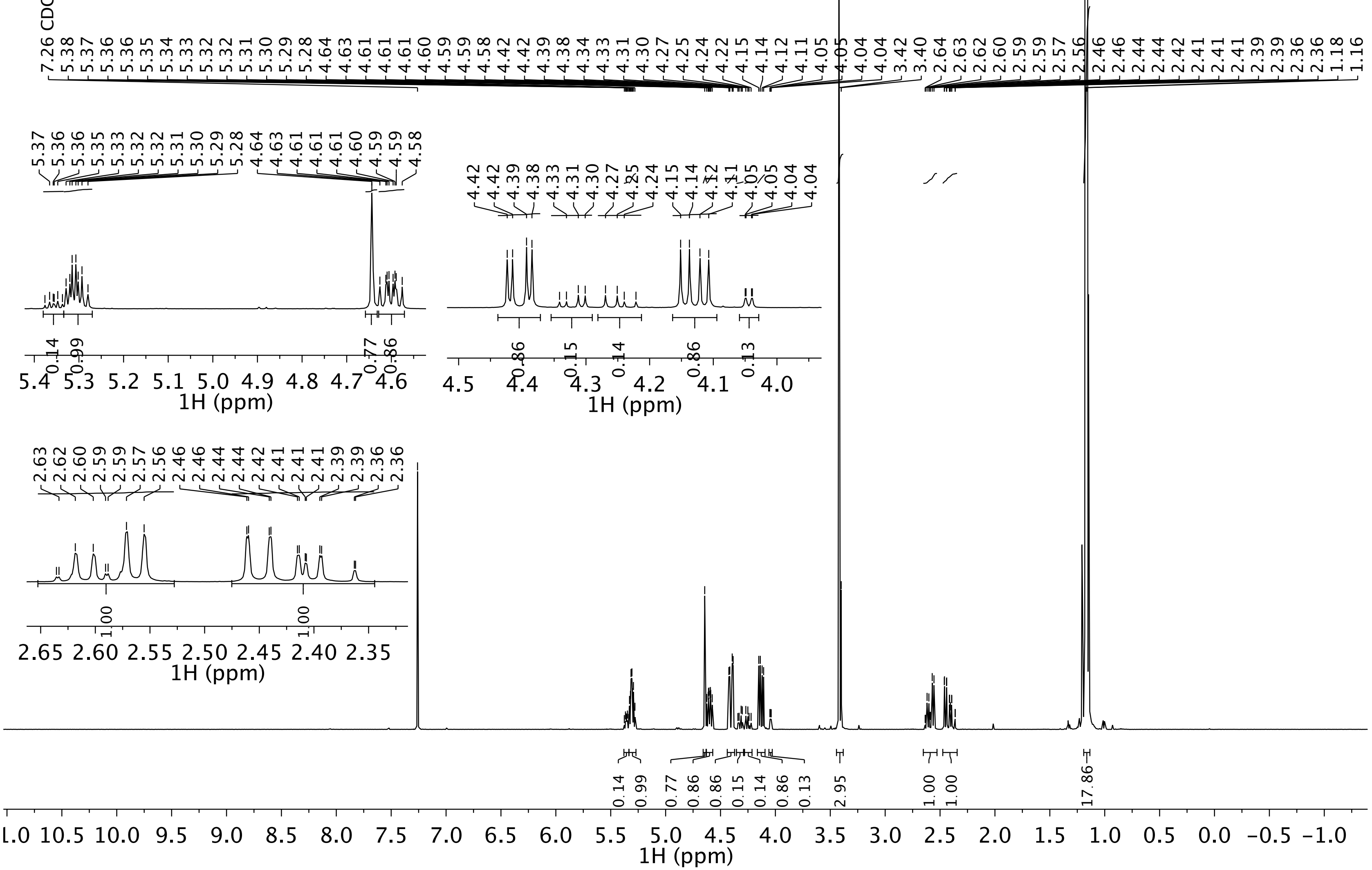

2h –^{13}C NMR (101 MHz, $CDCl_3$)

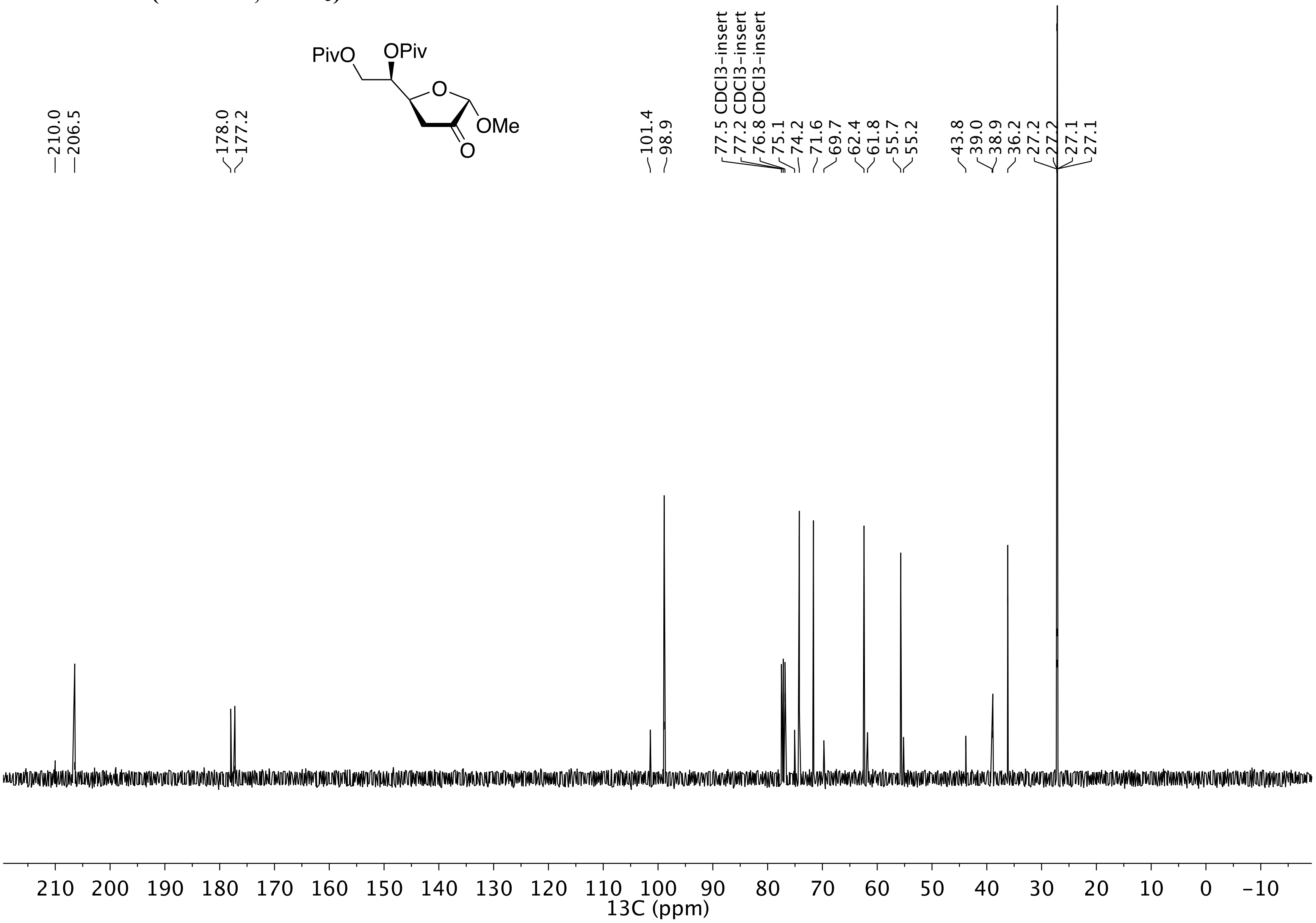

2h – COSY (400 MHz, CDCl$_3$)

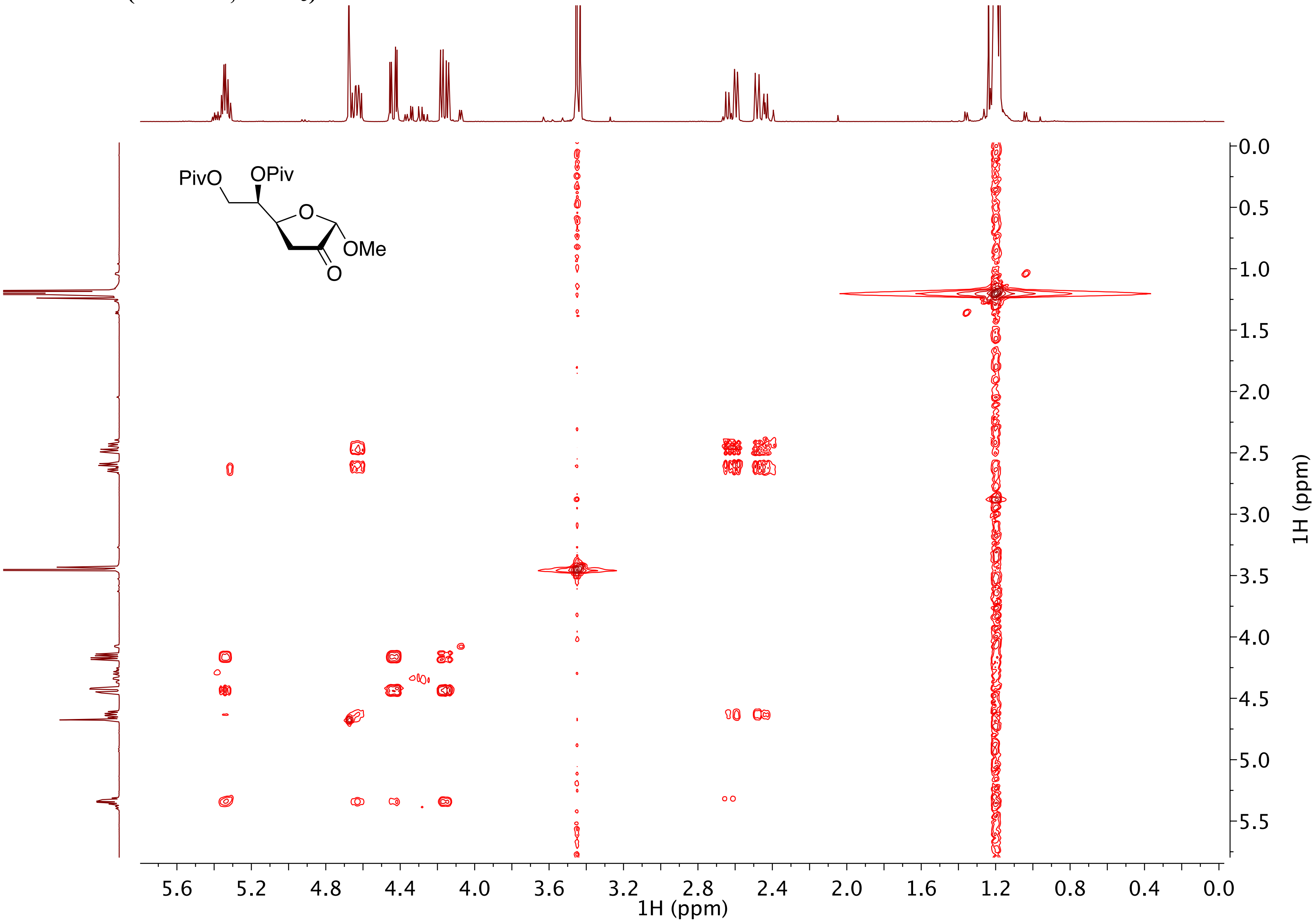

2h – HSQC (400 MHz, $CDCl_3$)

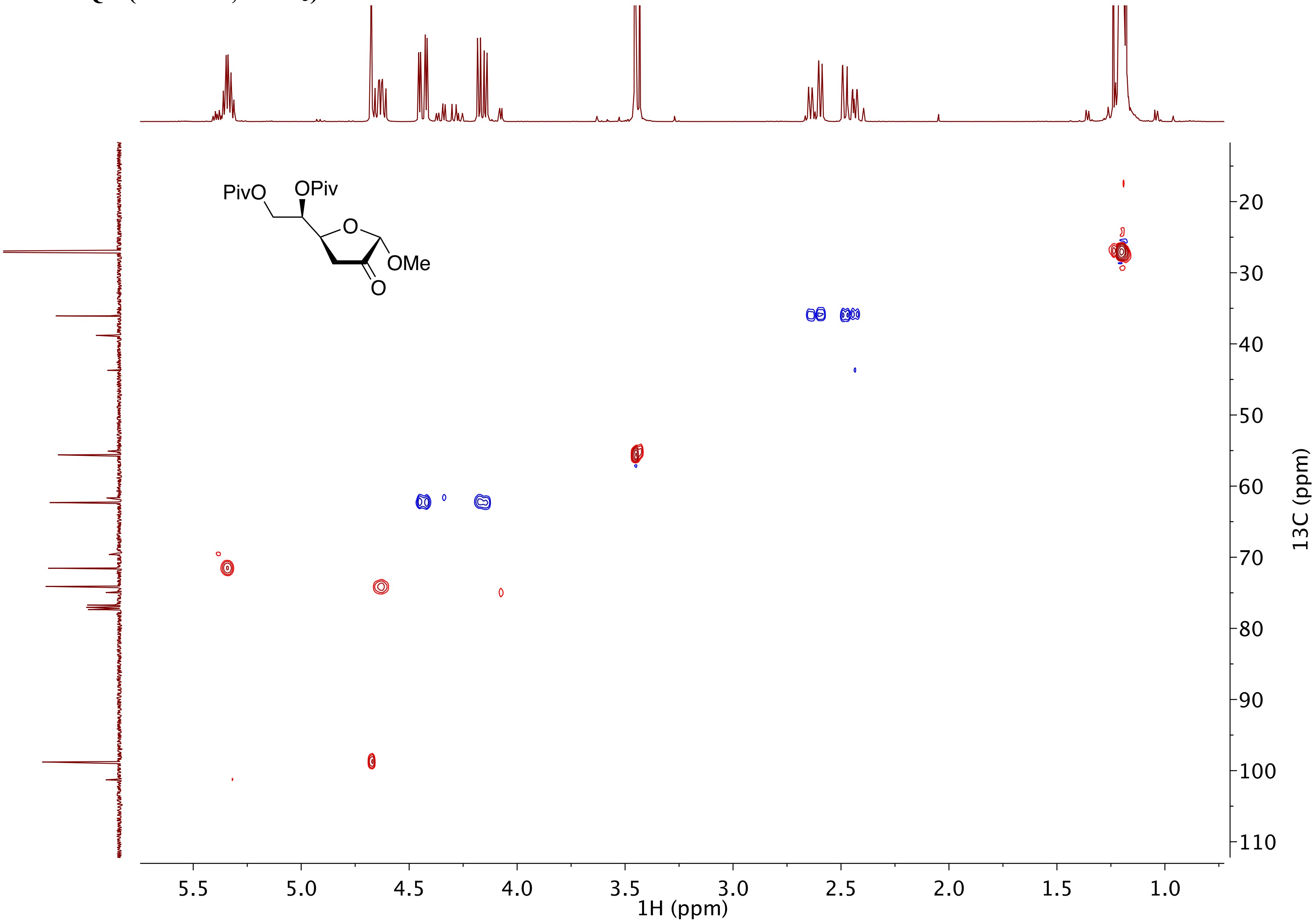

2i –^{1}H NMR (400 MHz, CH3CN)

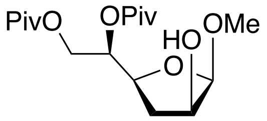

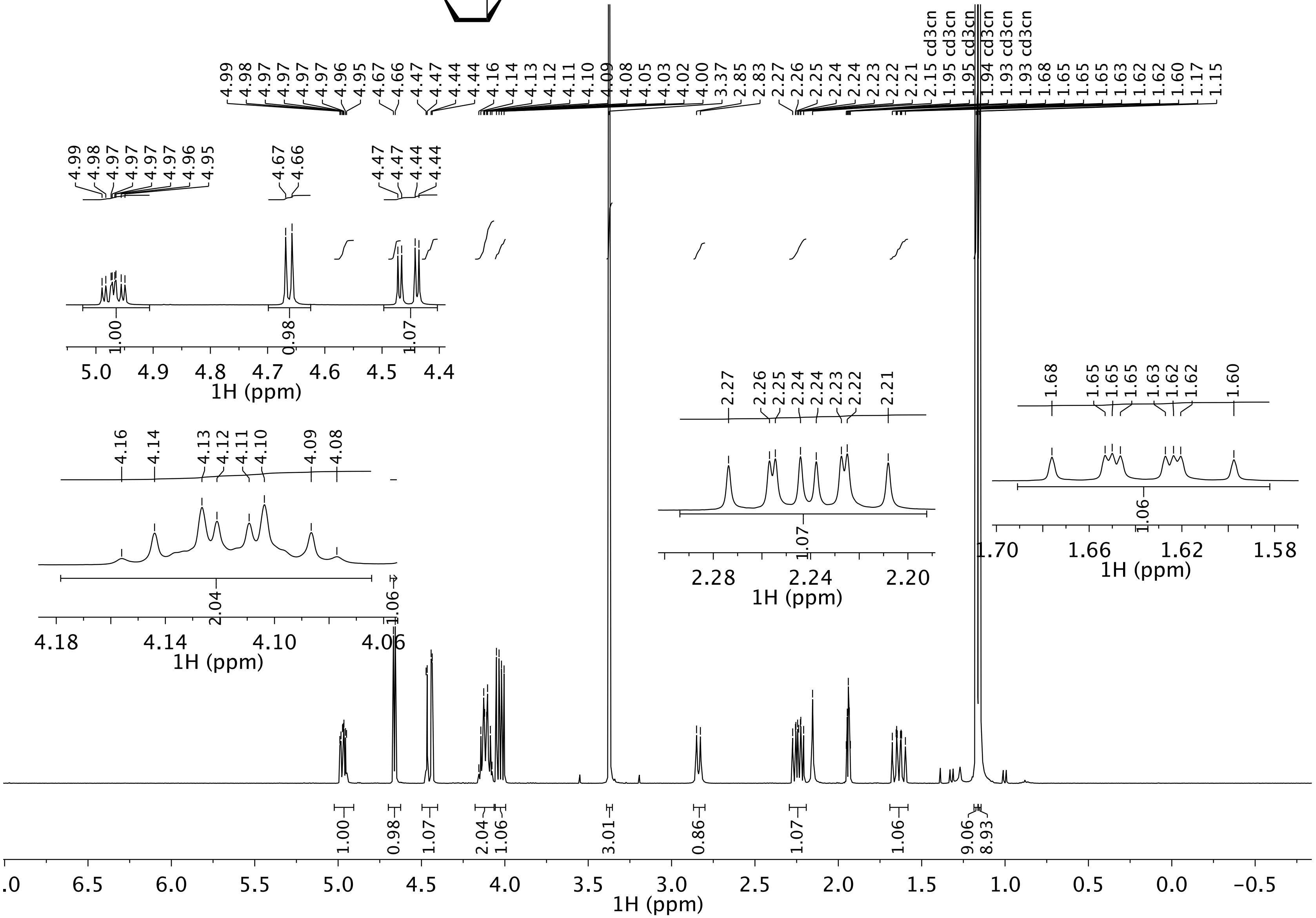

2i –^{13}C NMR (101 MHz, CH_3CN)

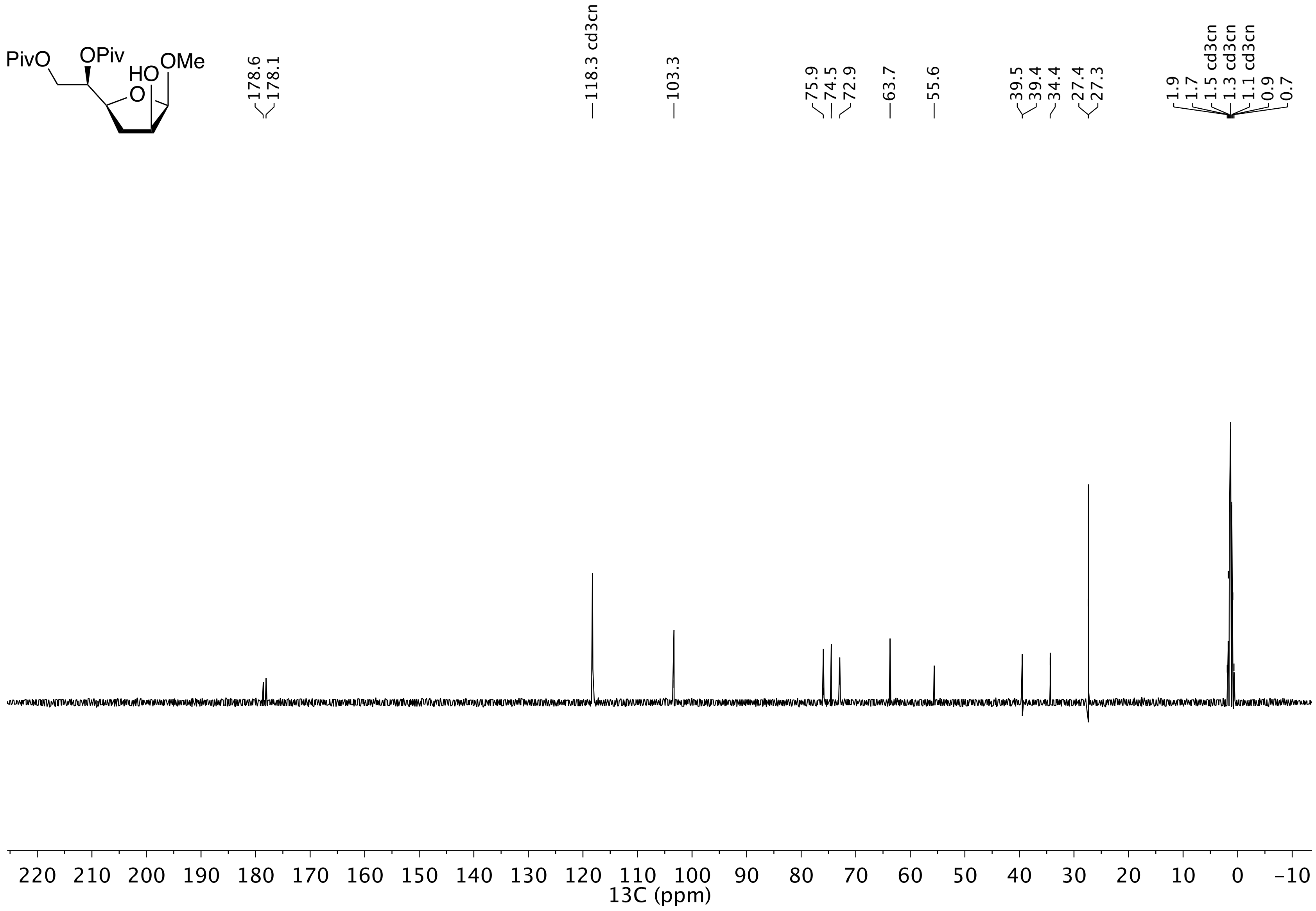

2i – COSY (400 MHz, CH_3CN)

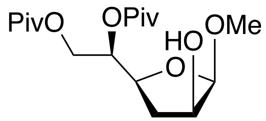

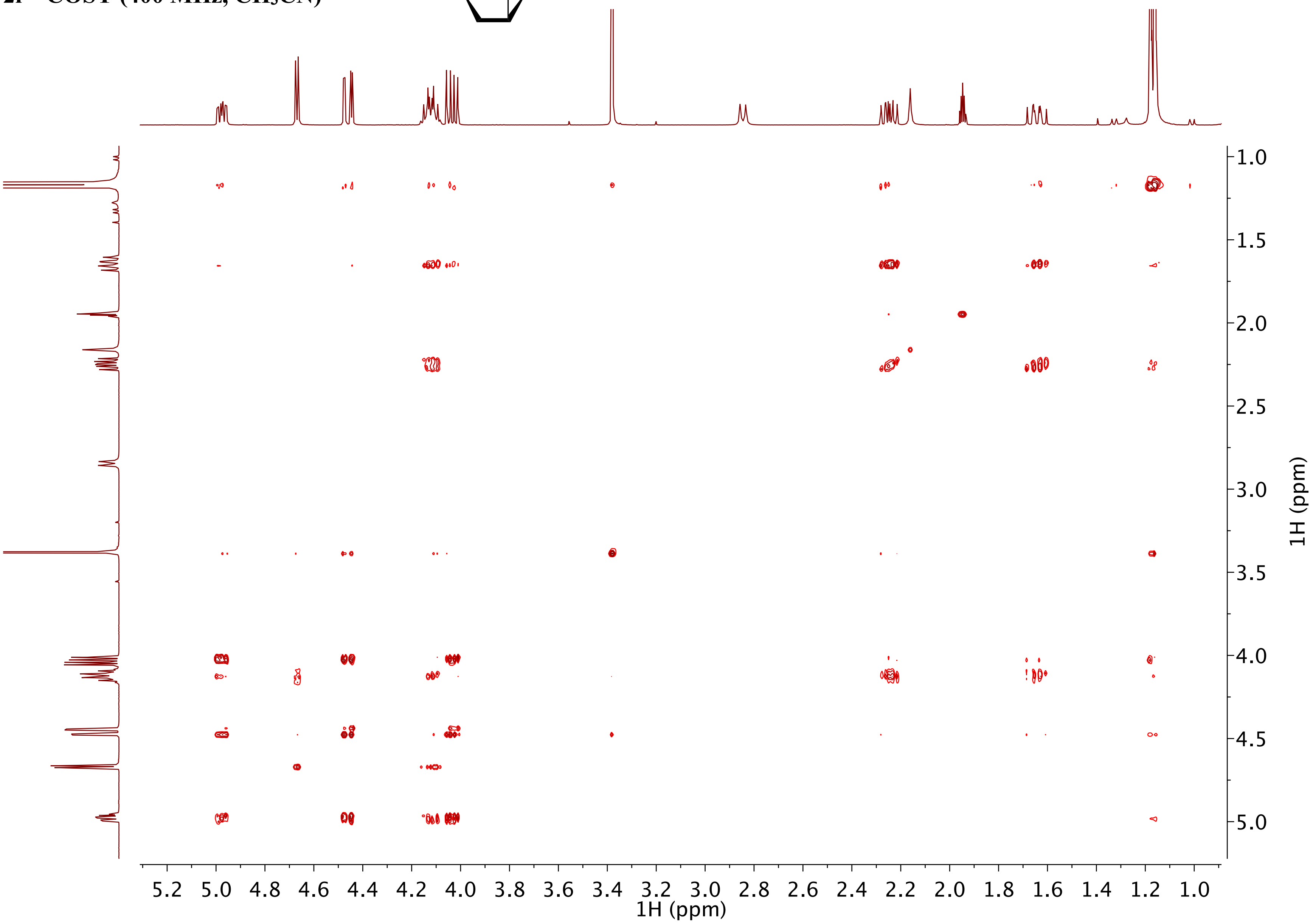

2i – HSQC (400 MHz, CH_3CN)

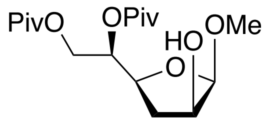

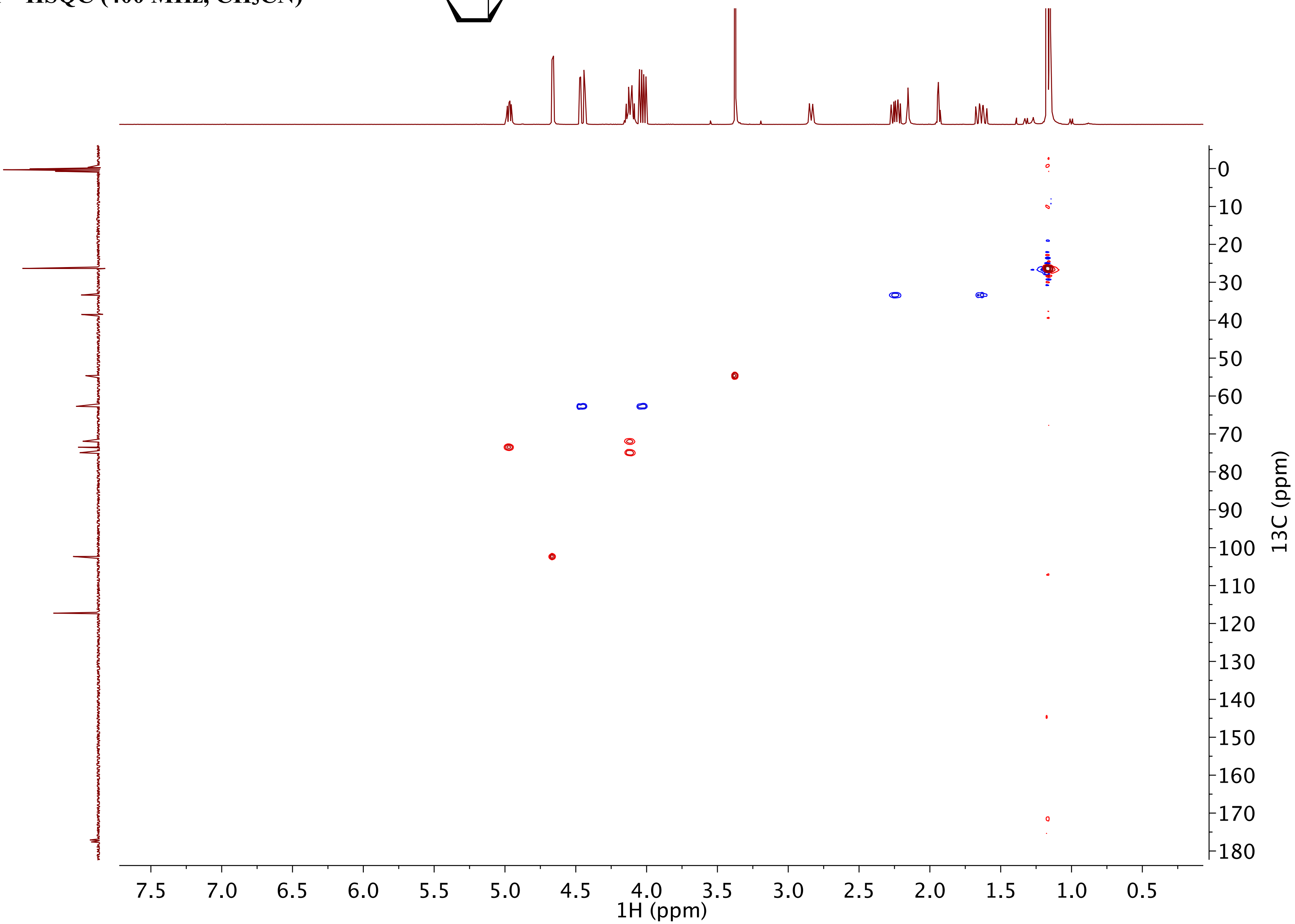

2m –¹H NMR (500 MHz, CDCl₃)

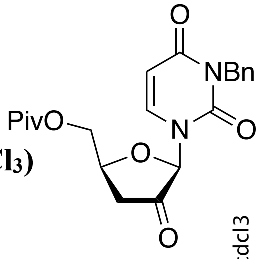

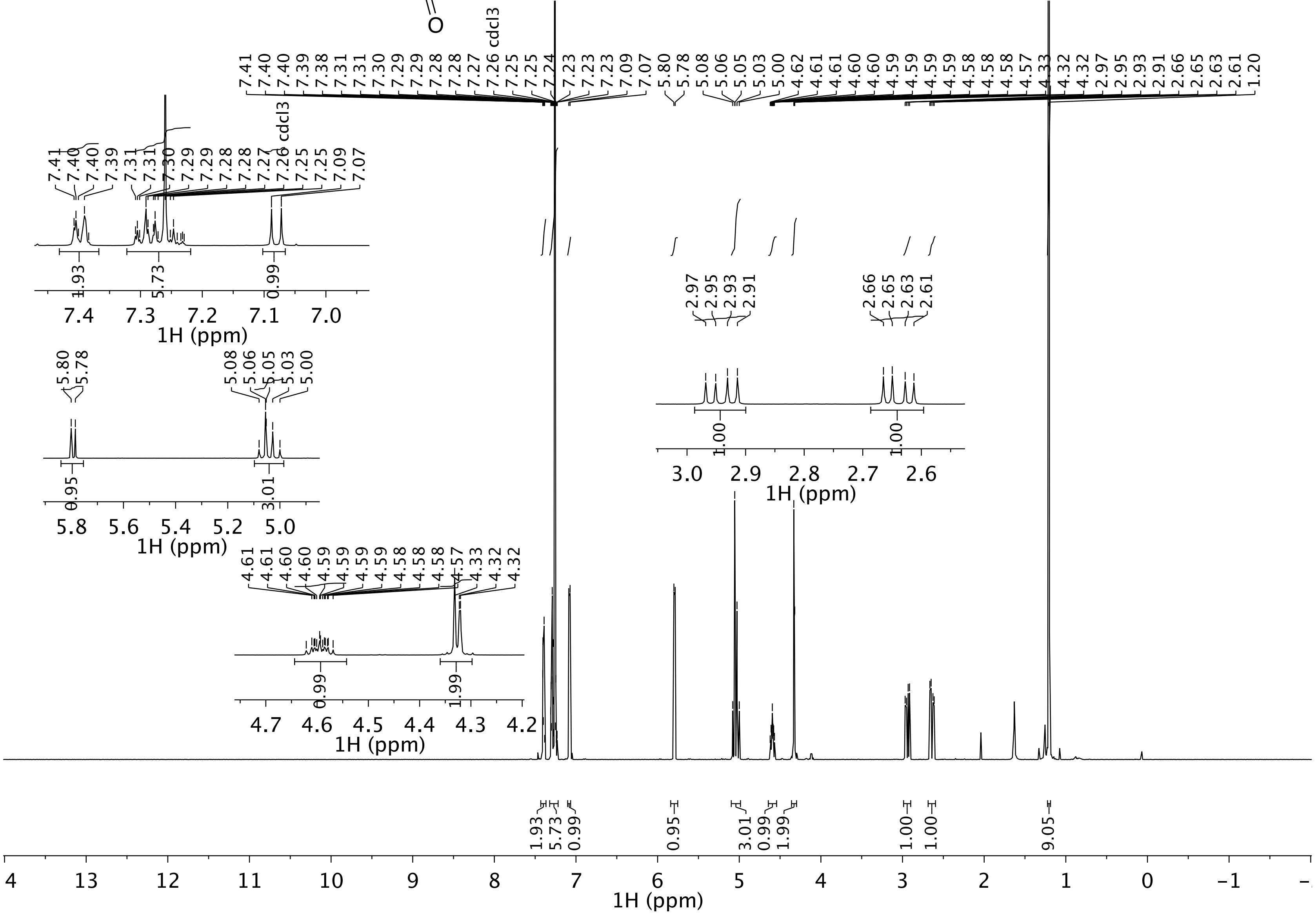

2m –^{13}C NMR (126 MHz, $CDCl_3$)

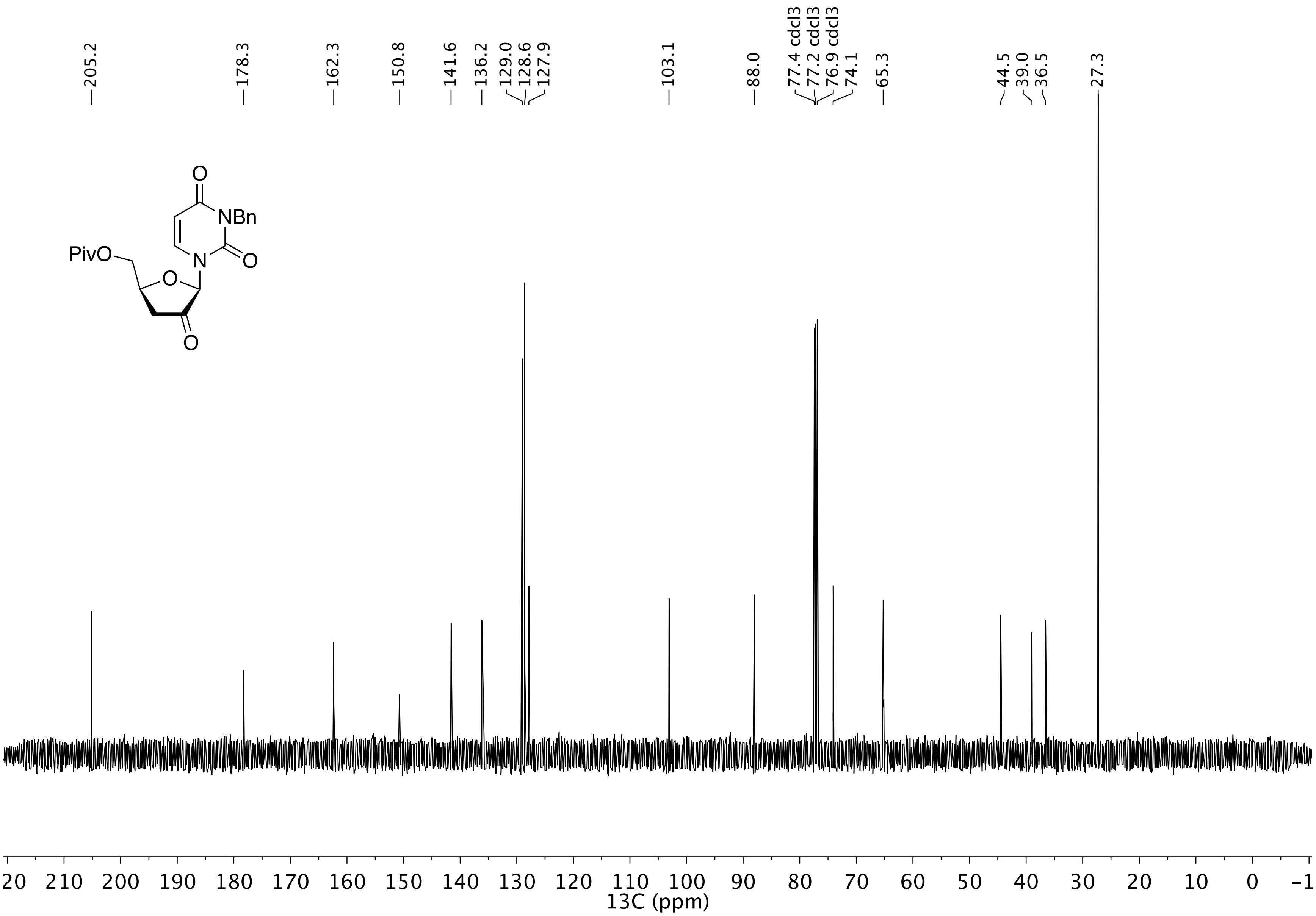

2m –COSY (500 MHz, $CDCl_3$)

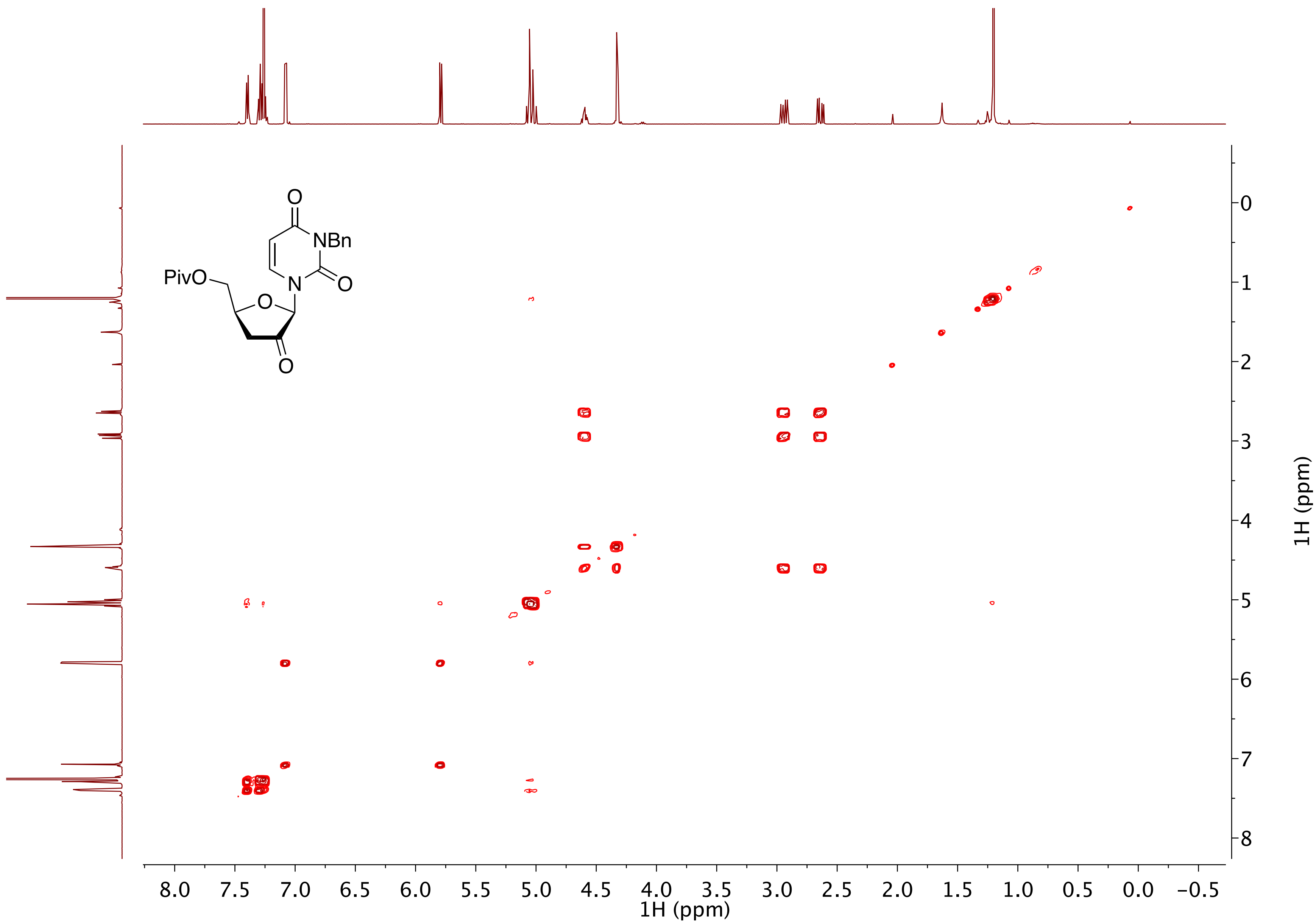

2m –HSQC(500 MHz, CDCl$_3$)

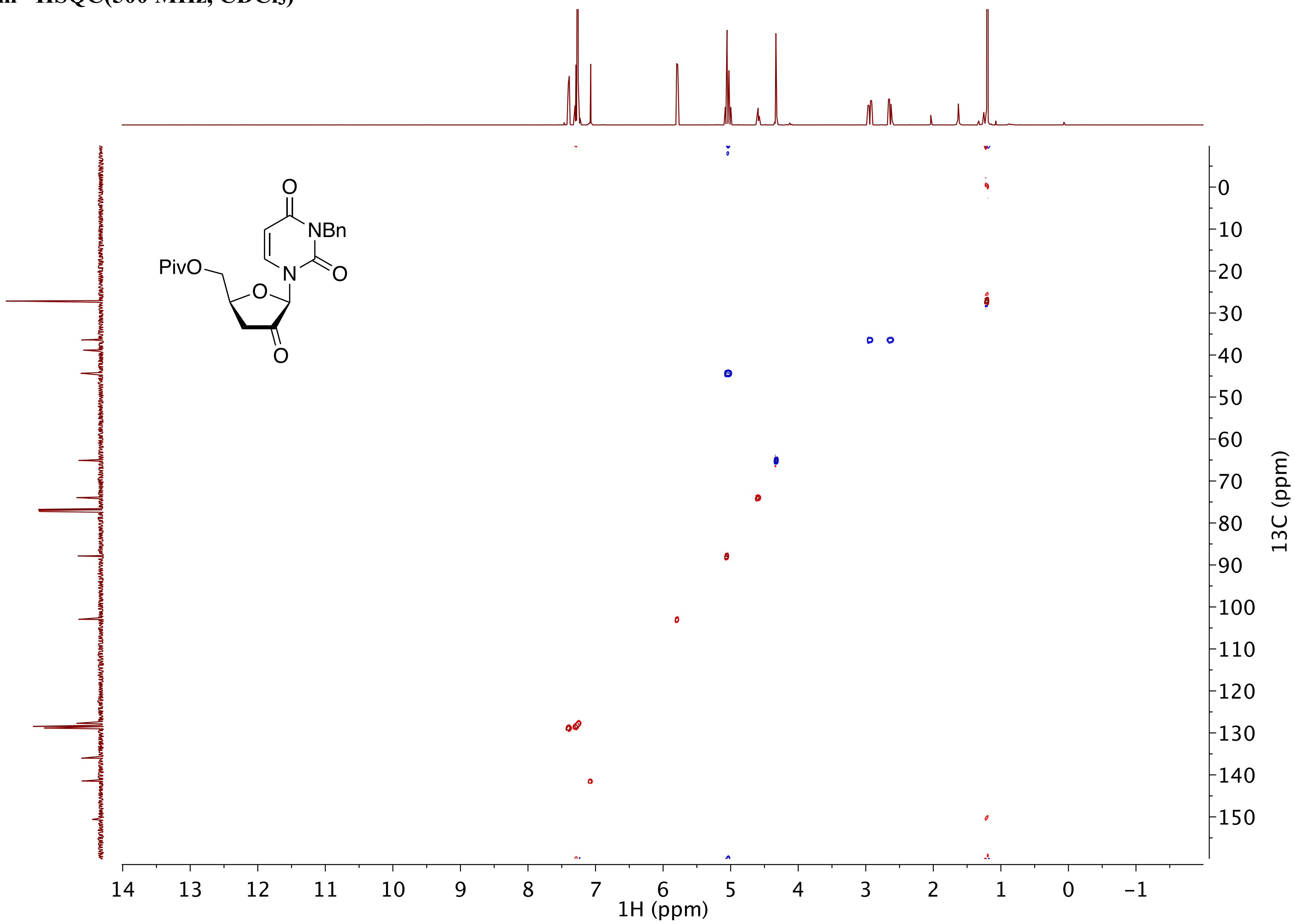

S1 –^{1}H NMR (400 MHz, $CDCl_3$)

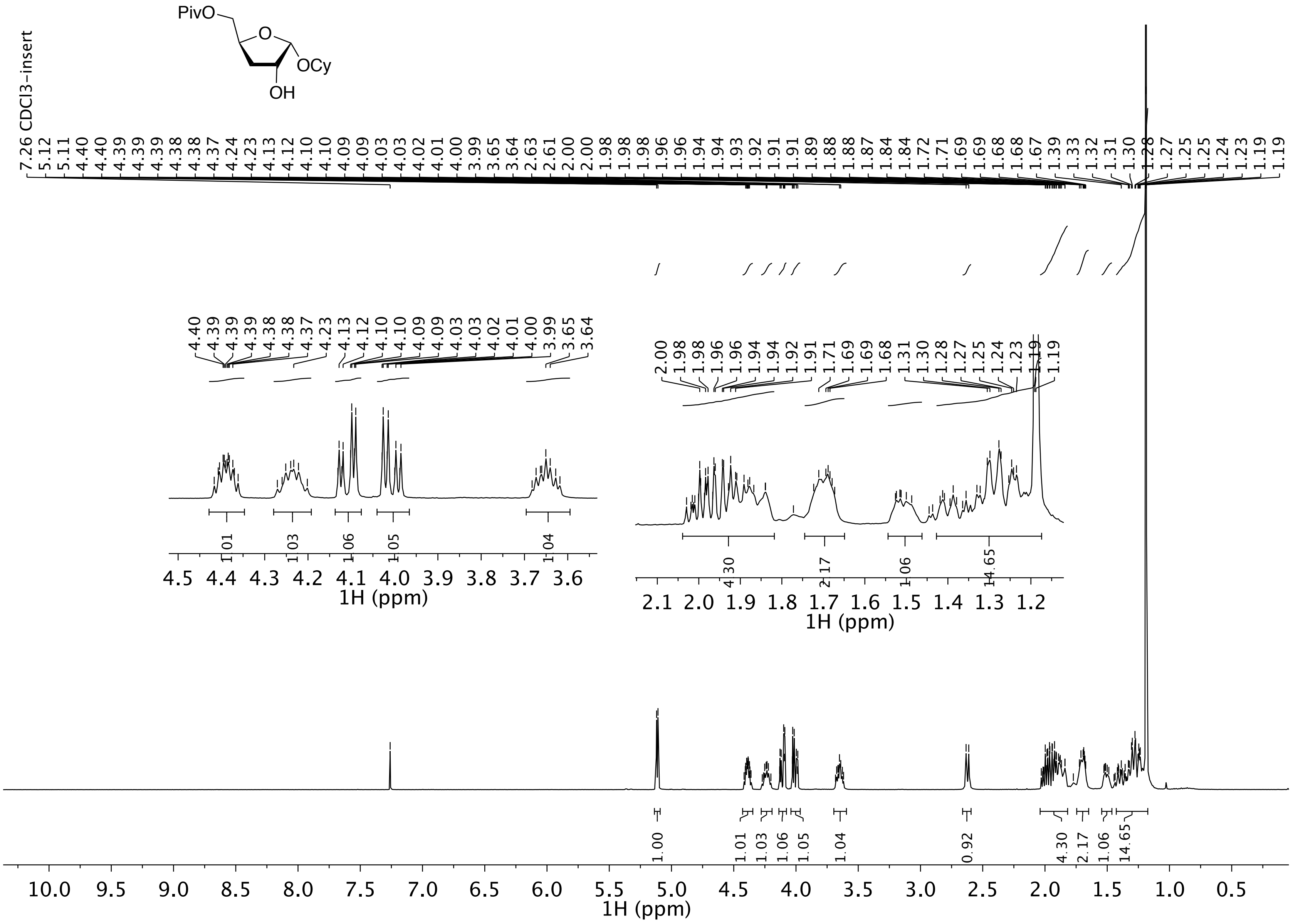

S1 –^{13}C NMR (101 MHz, $CDCl_3$)

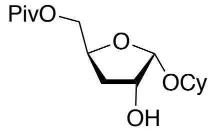

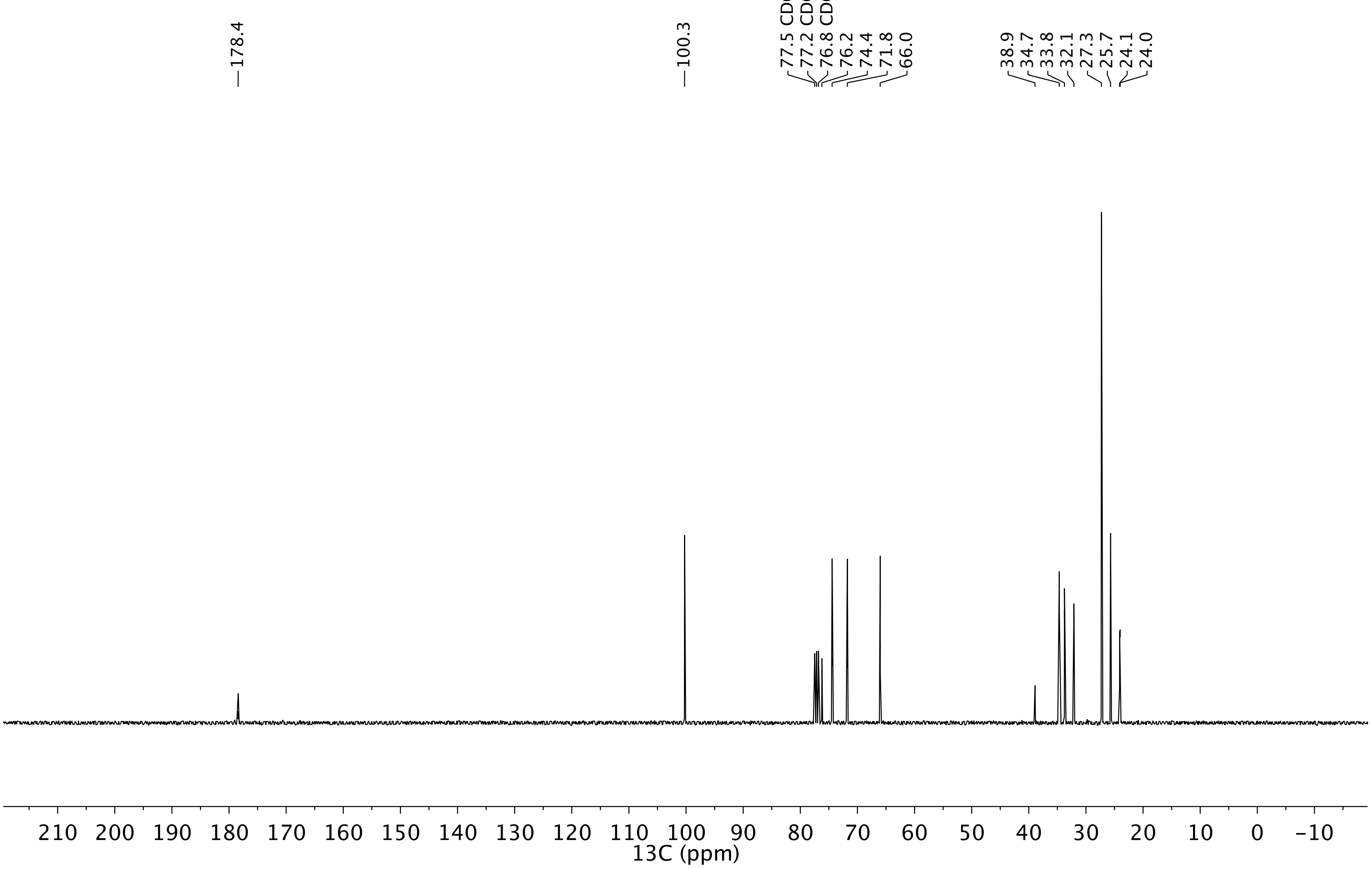

S1 – COSY (400 MHz, $CDCl_3$)

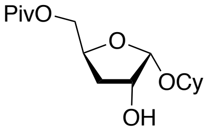

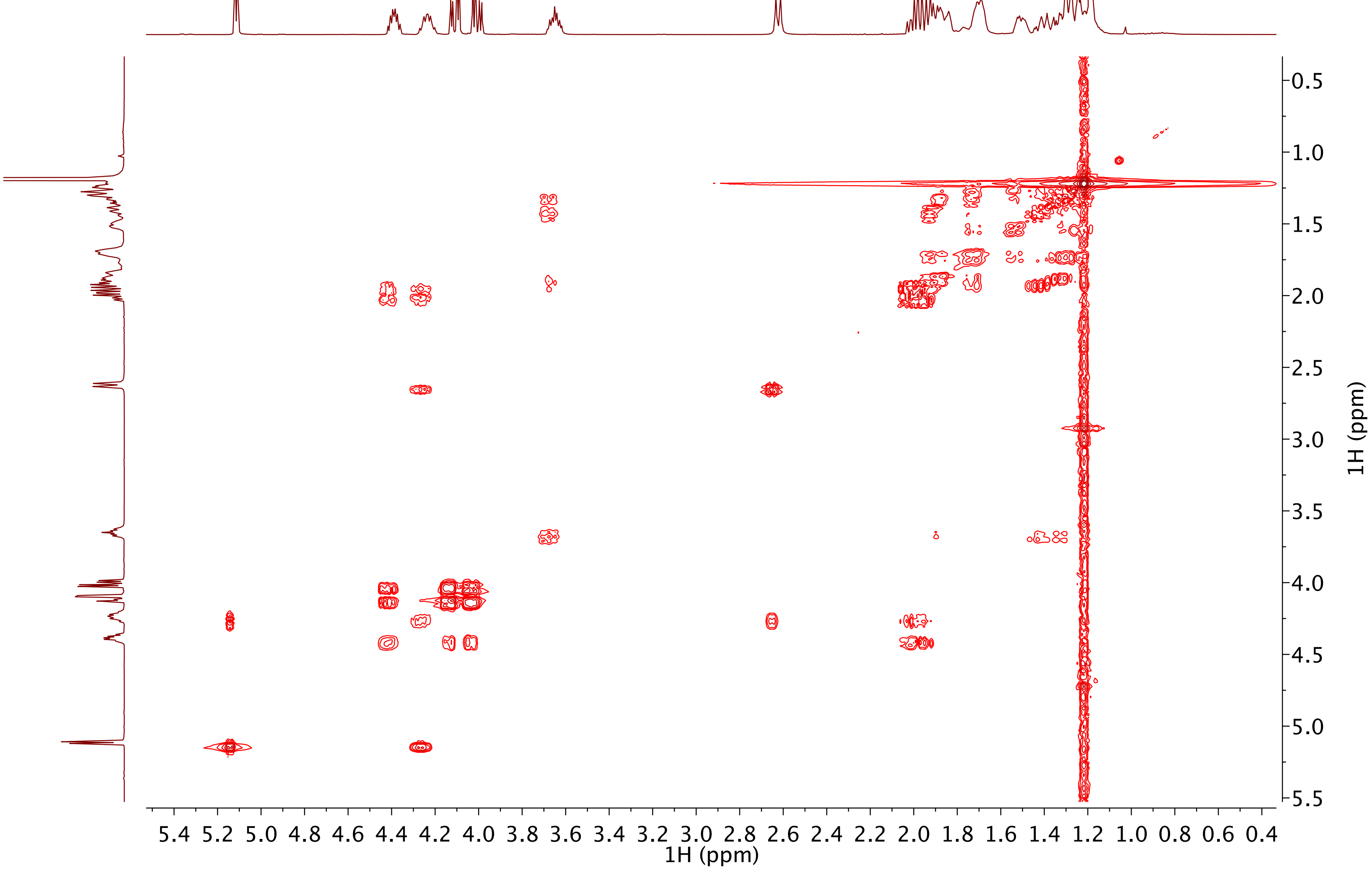

S2 –¹H NMR (400 MHz, CD_3CN)

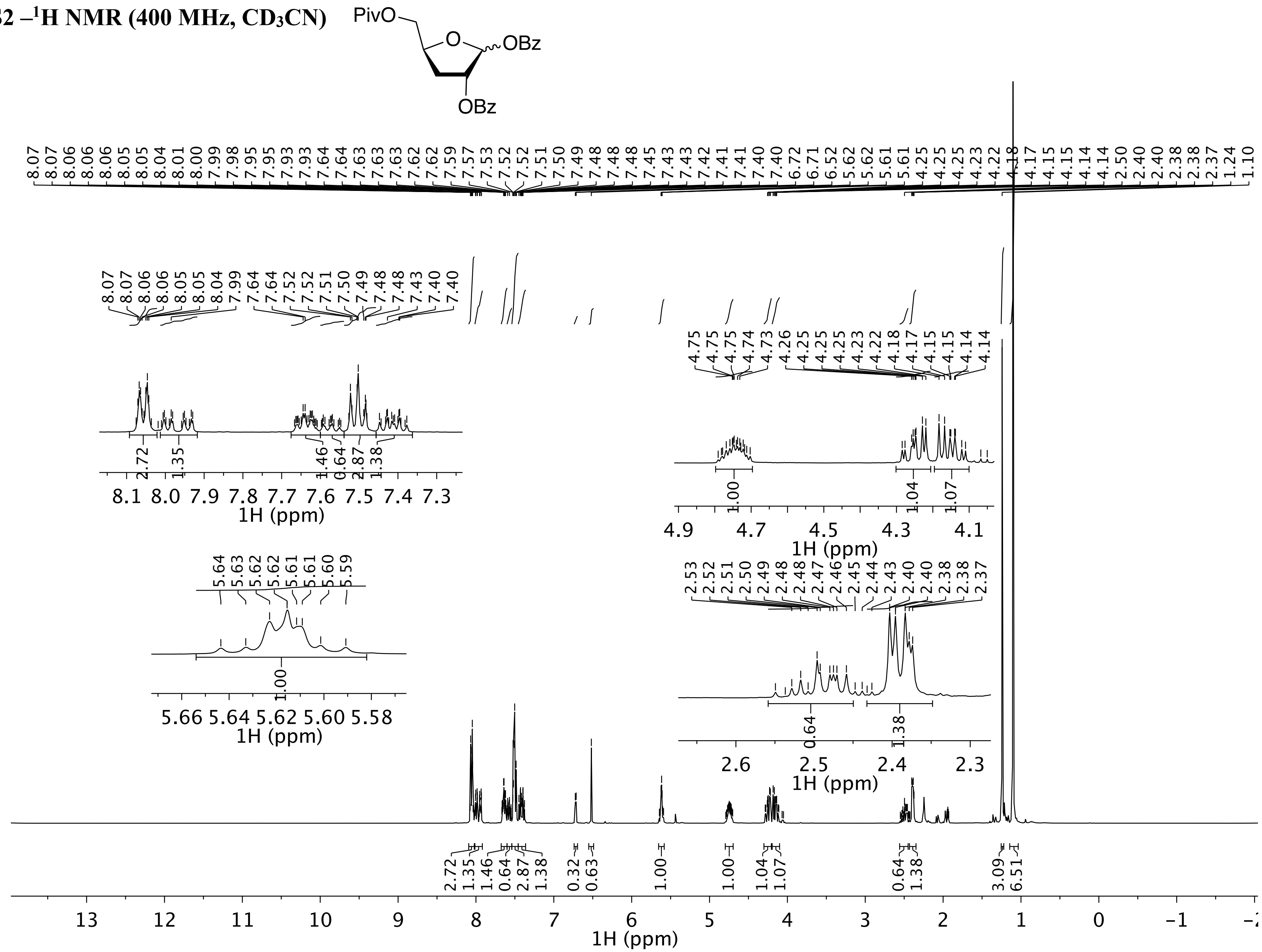

S2 –^{13}C NMR (101 MHz, CD_3CN)

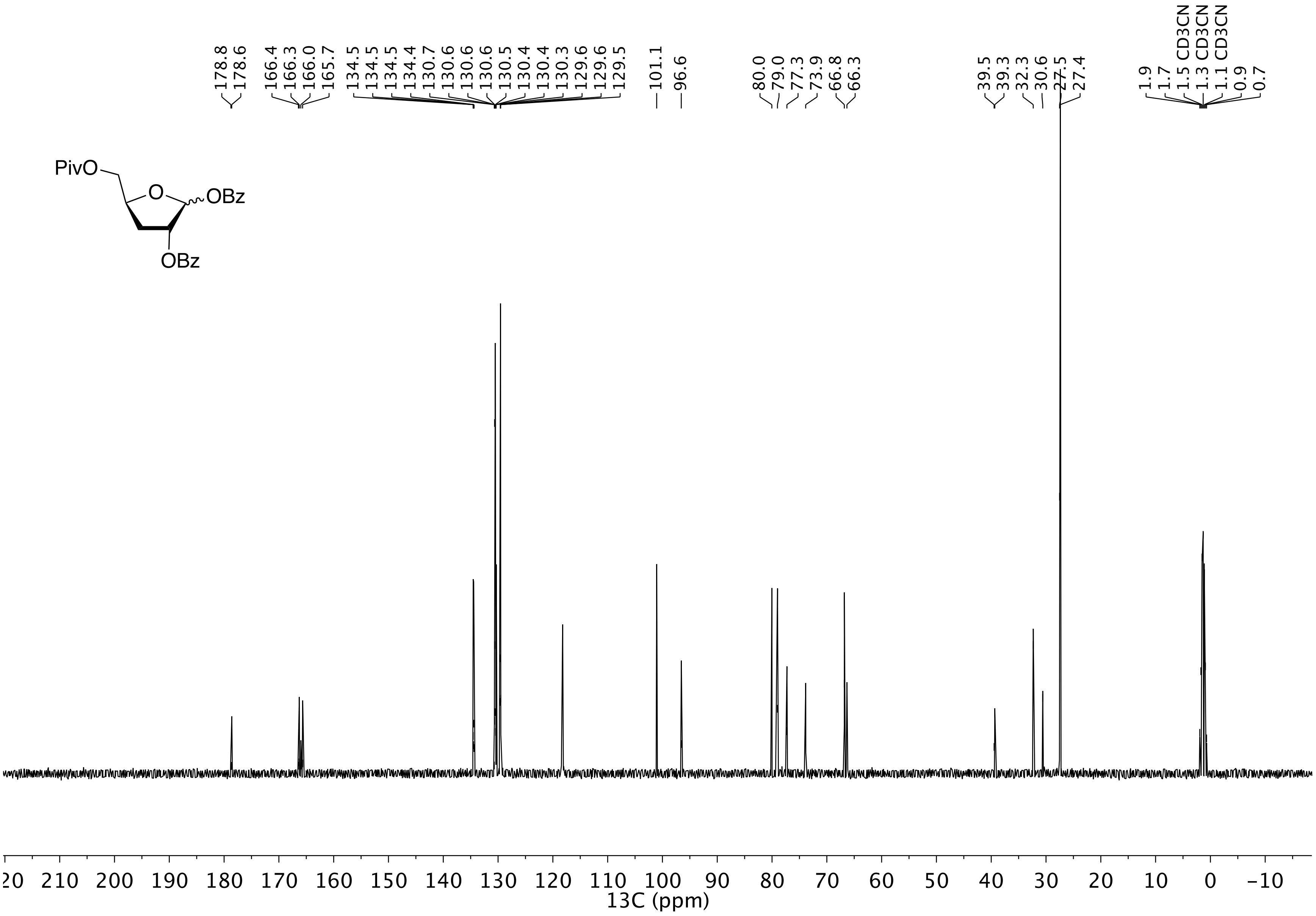

S2 –COSY (400 MHz, CD_3CN)

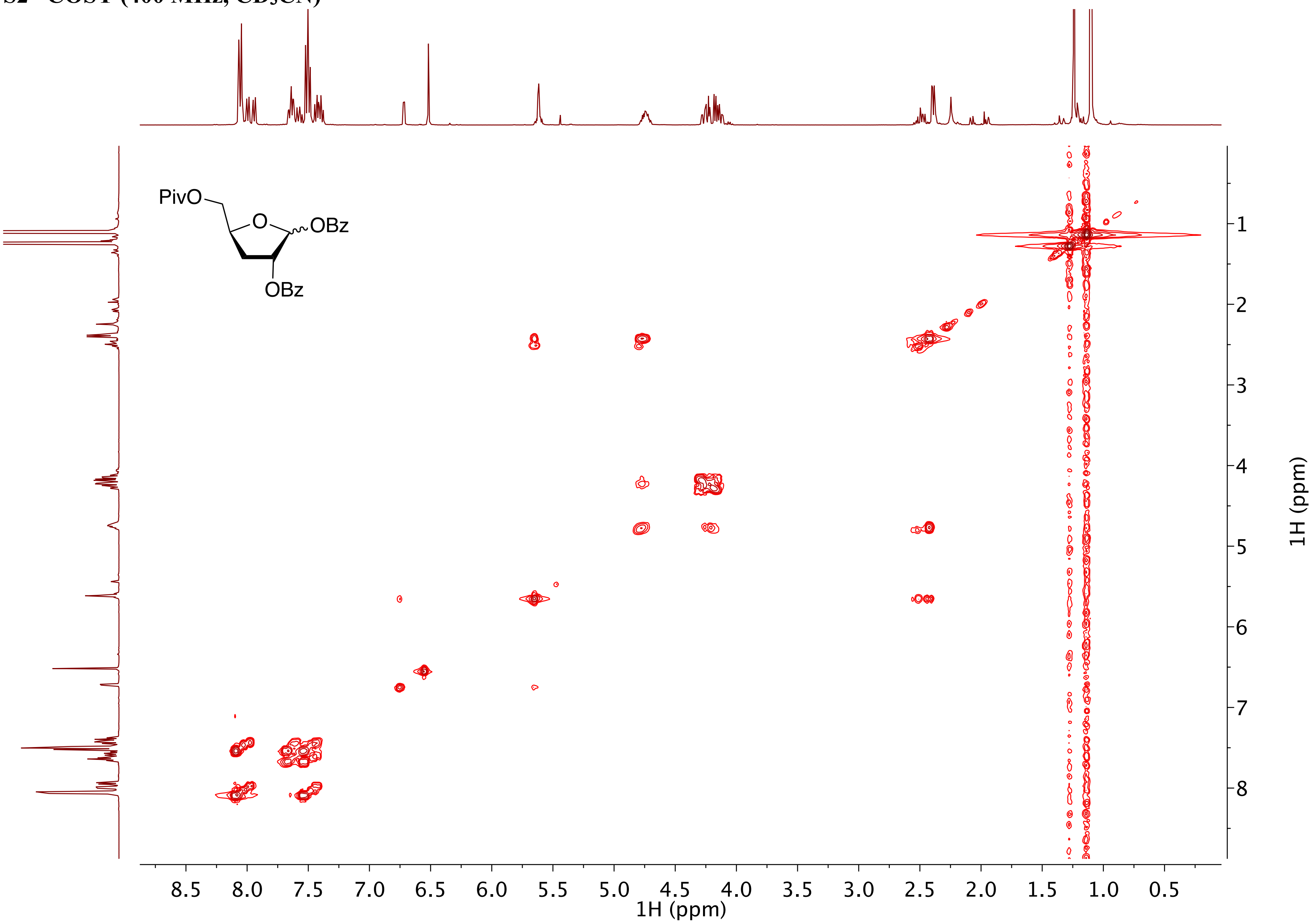

5 –^{1}H NMR (400 MHz, CD_3CN)

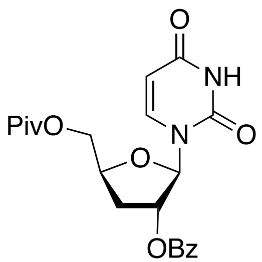

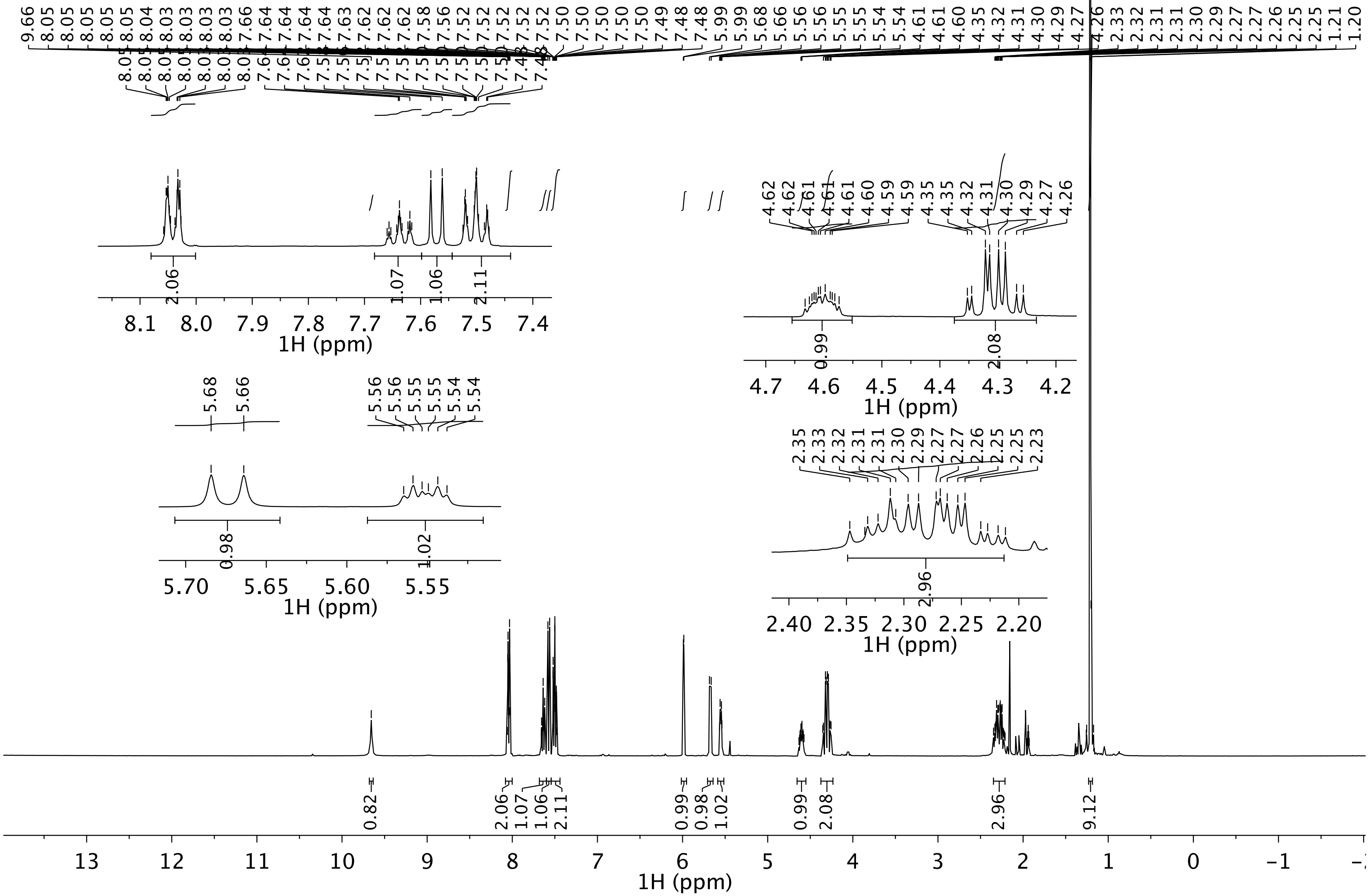

5 –^{13}C NMR (101 MHz, CD$_3$CN)

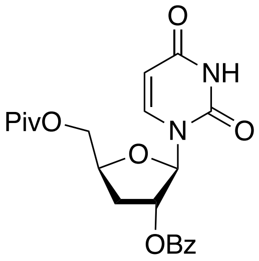

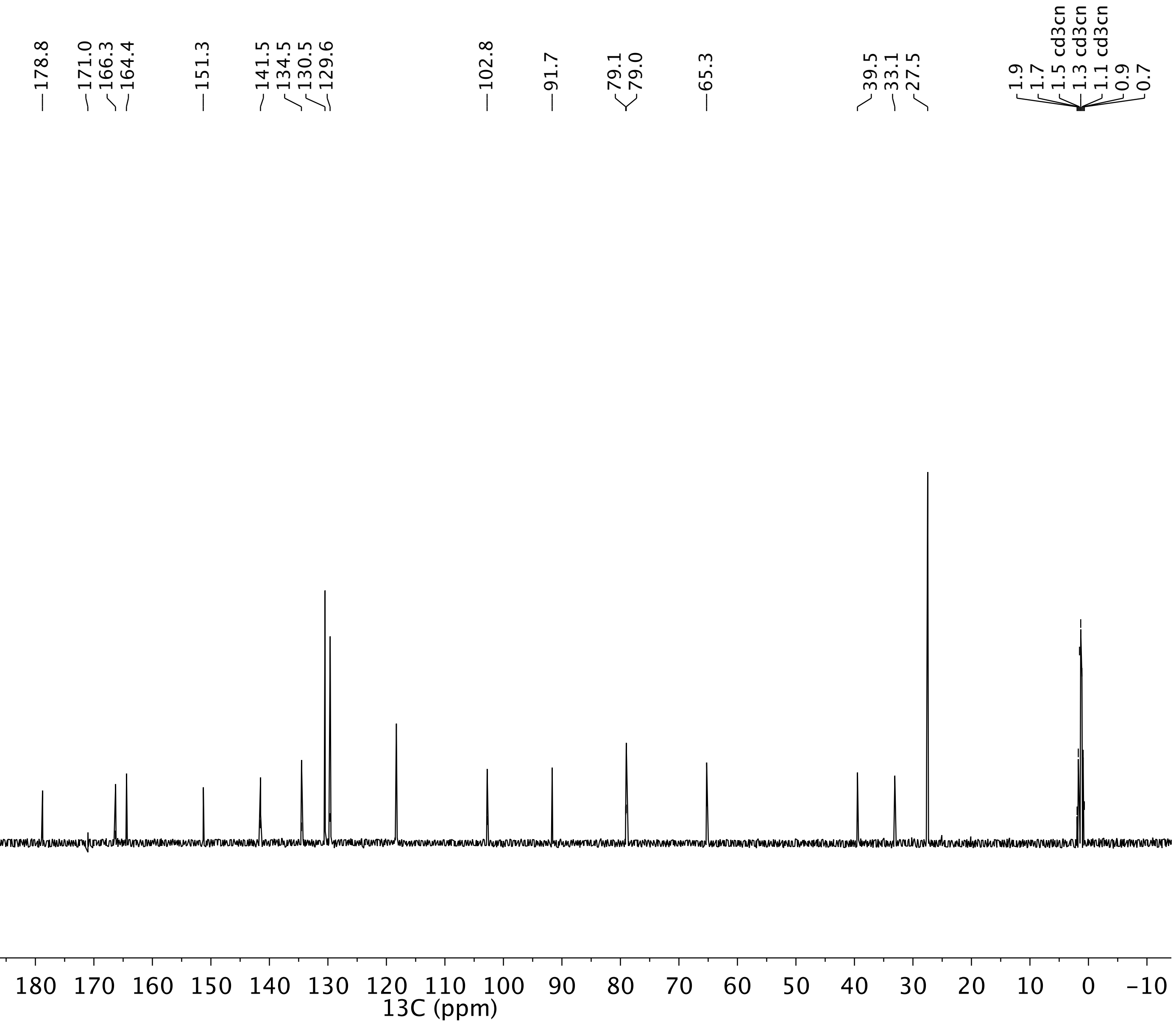

5 –COSY (400 MHz, CD_3CN)

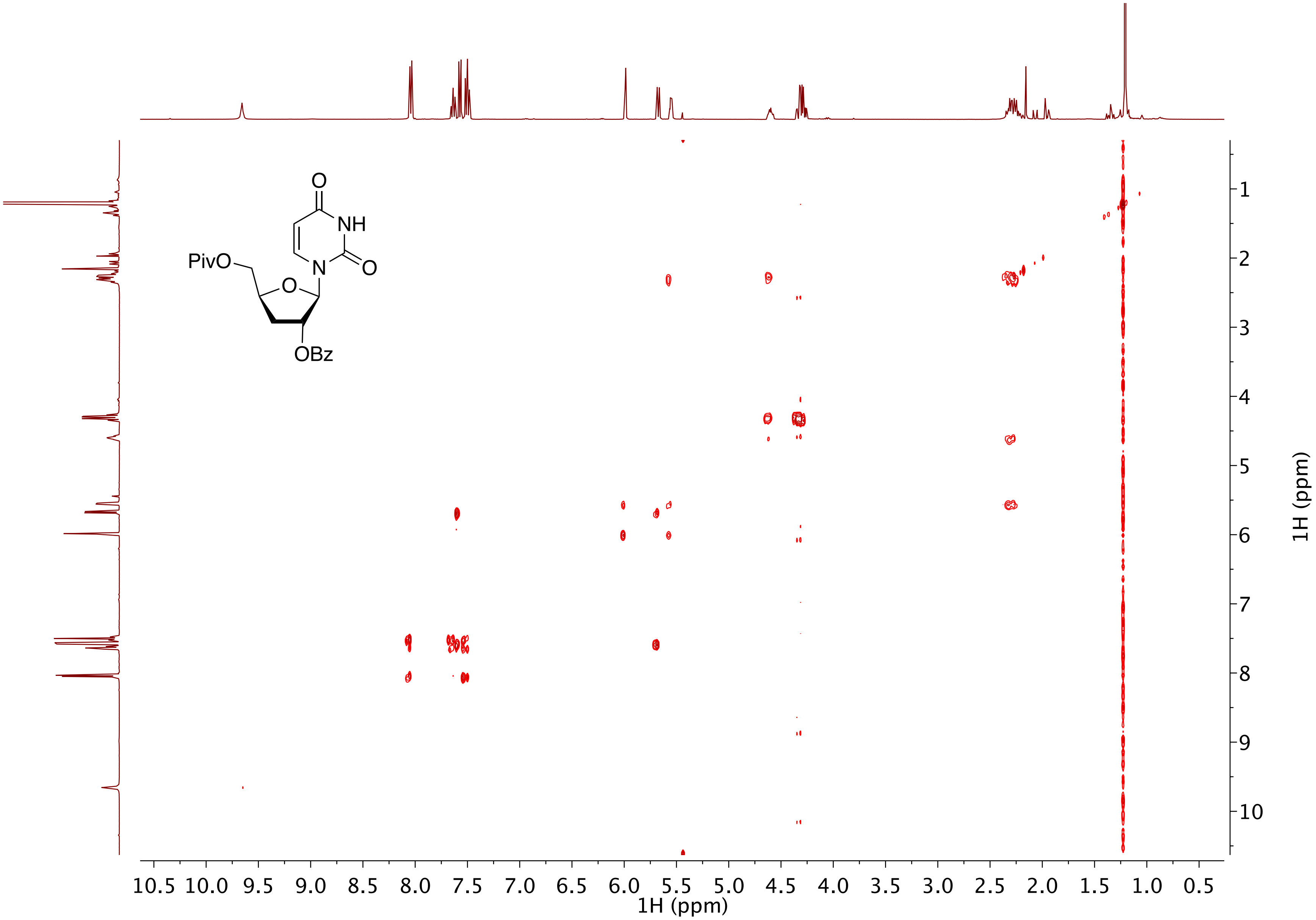

5 –HSQC (400 MHz, CD_3CN)

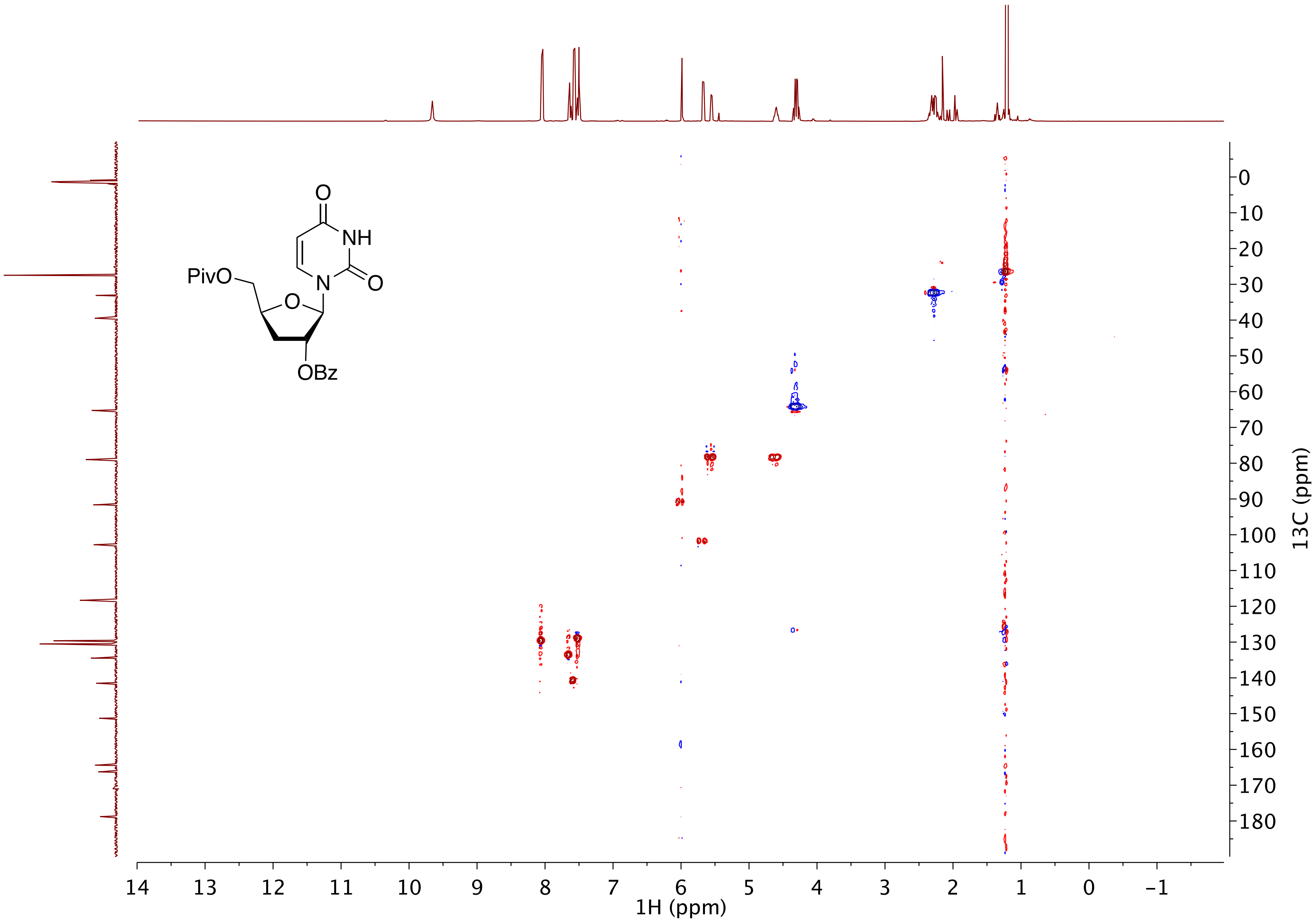

5 –HMBC (400 MHz, CD_3CN)

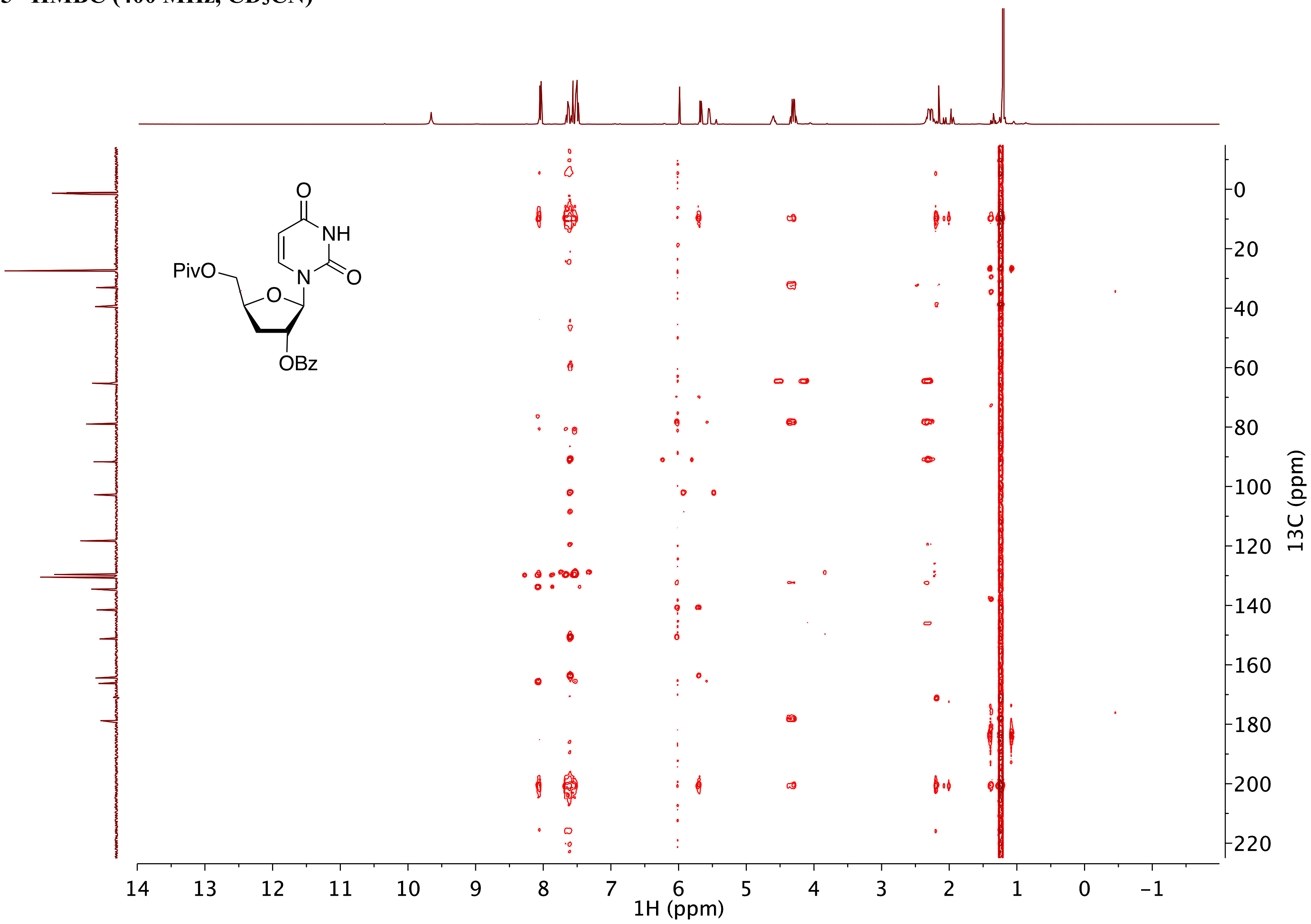

5 –NOESY (400 MHz, CD_3CN)

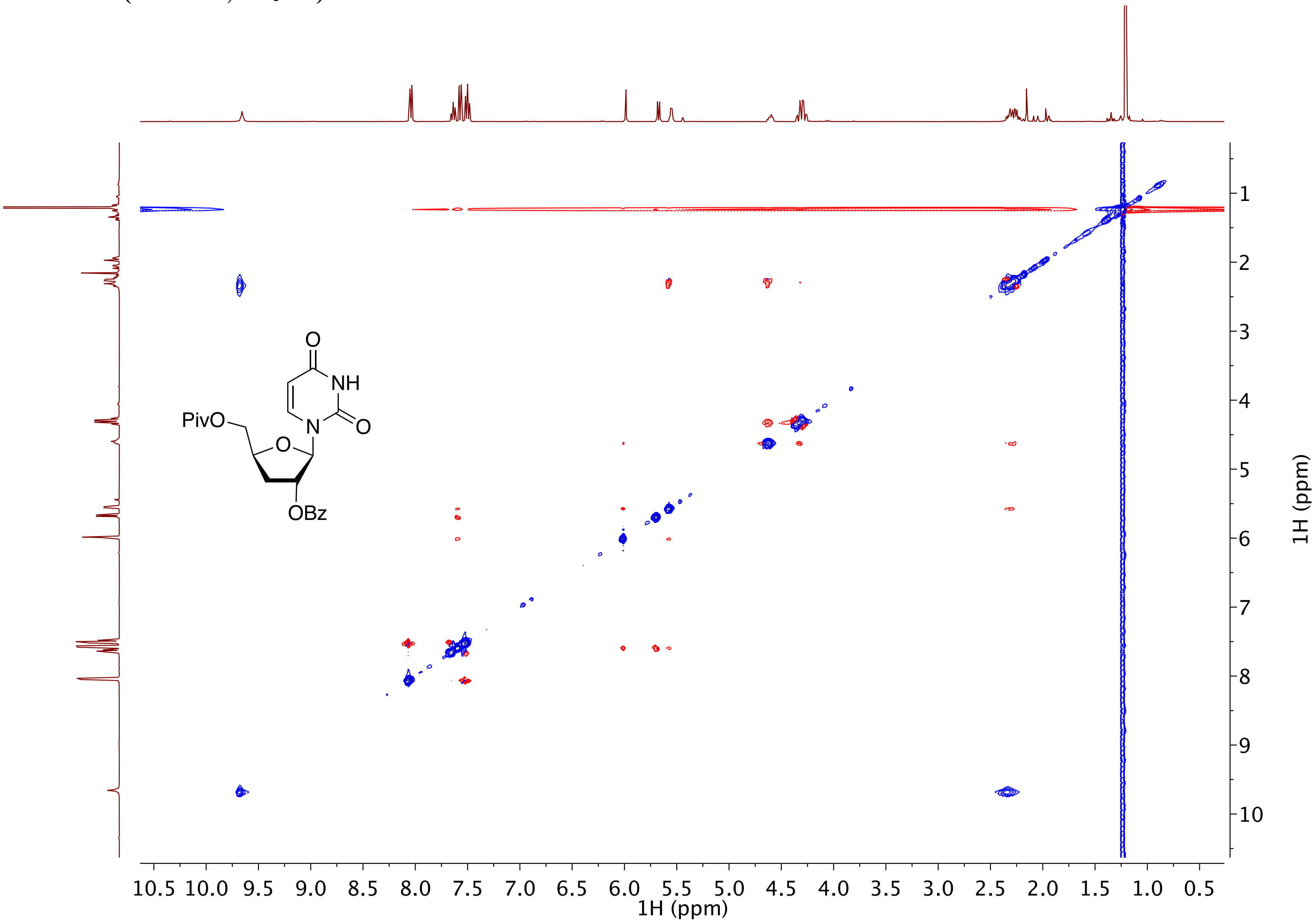

S3 –^{1}H NMR (400 MHz, $CDCl_3$)

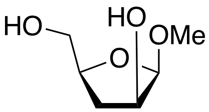

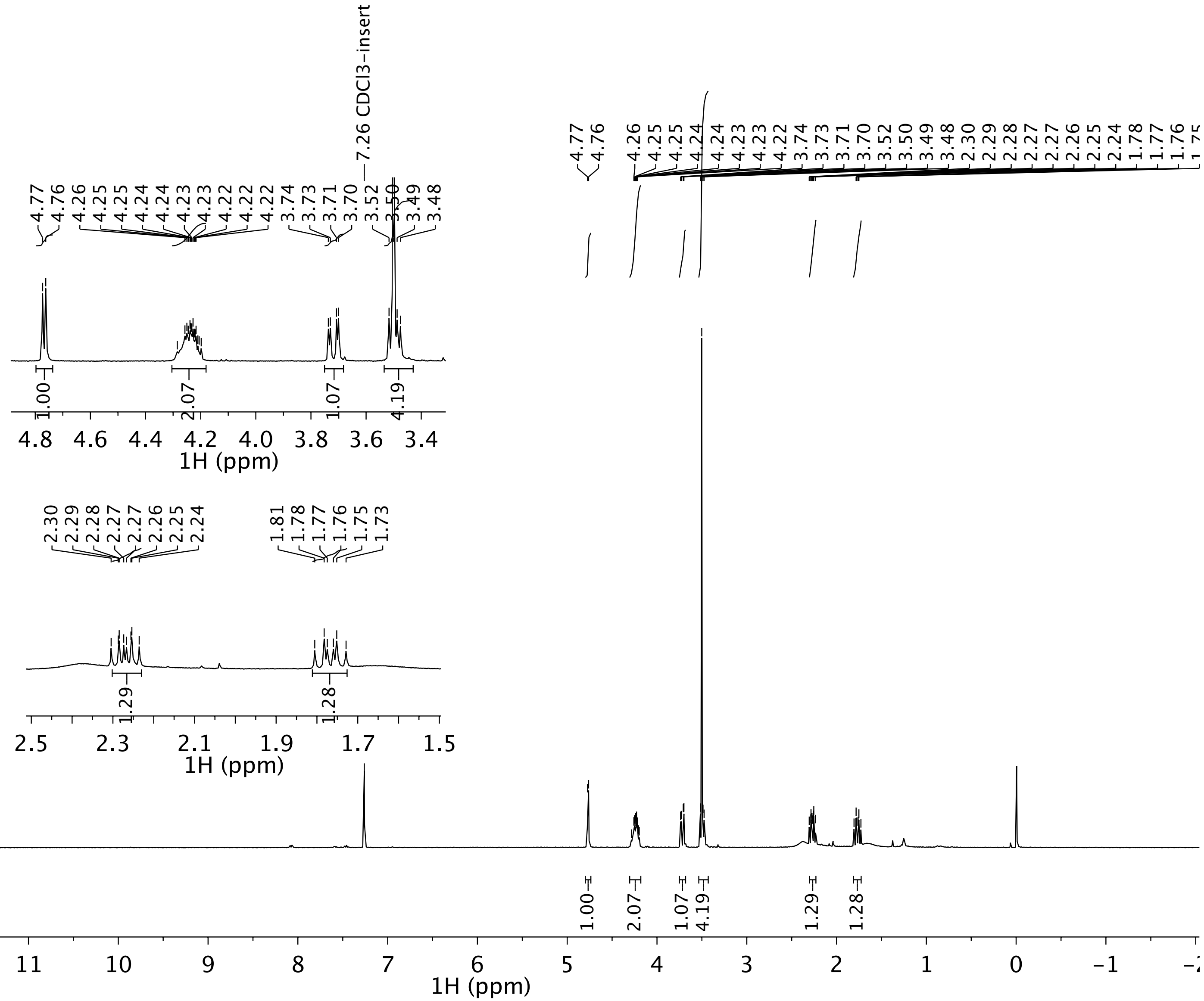

S3 –^{13}C NMR (101 MHz, $CDCl_3$)

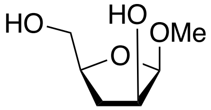

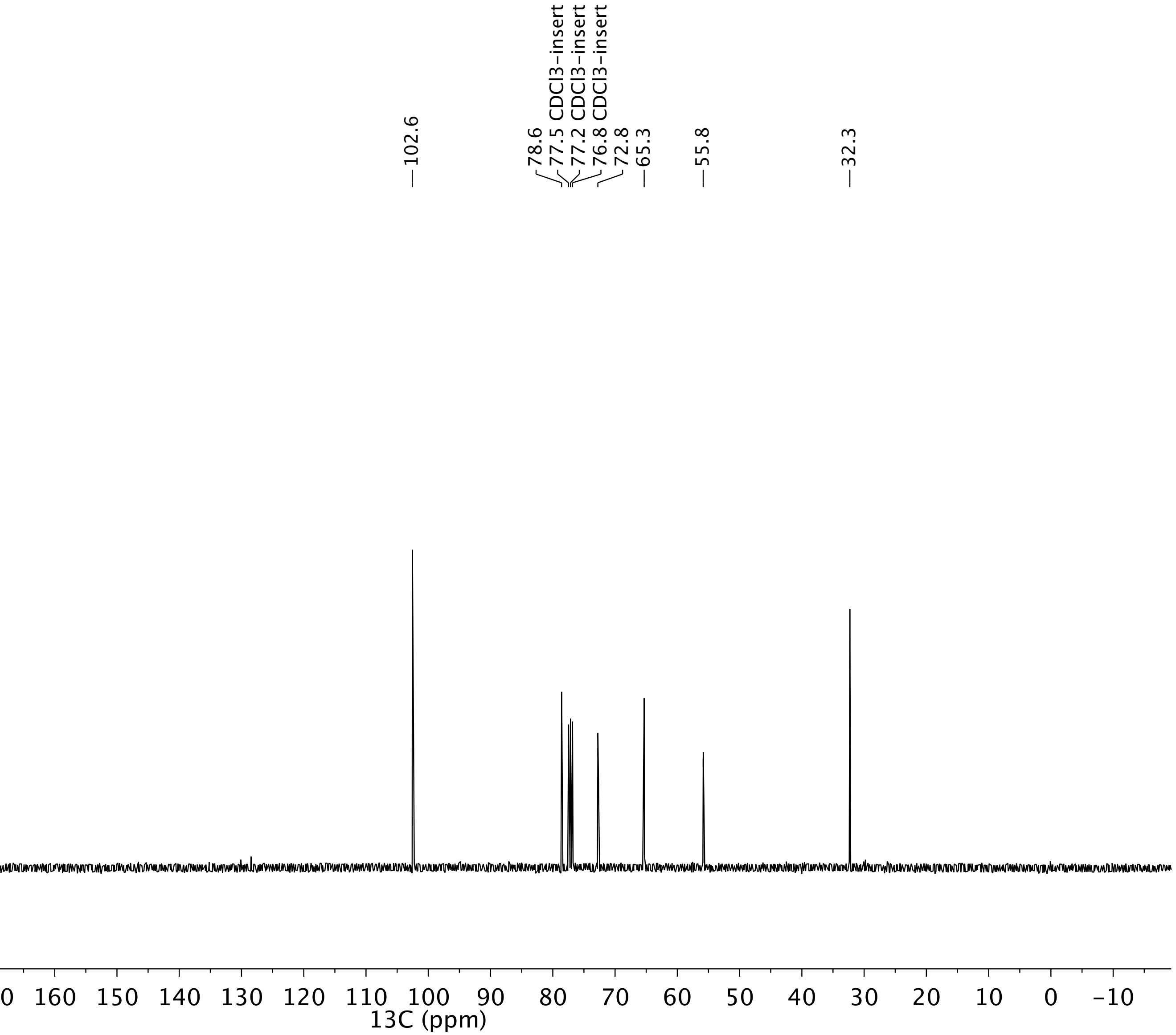

S3 – COSY (400 MHz, $CDCl_3$)

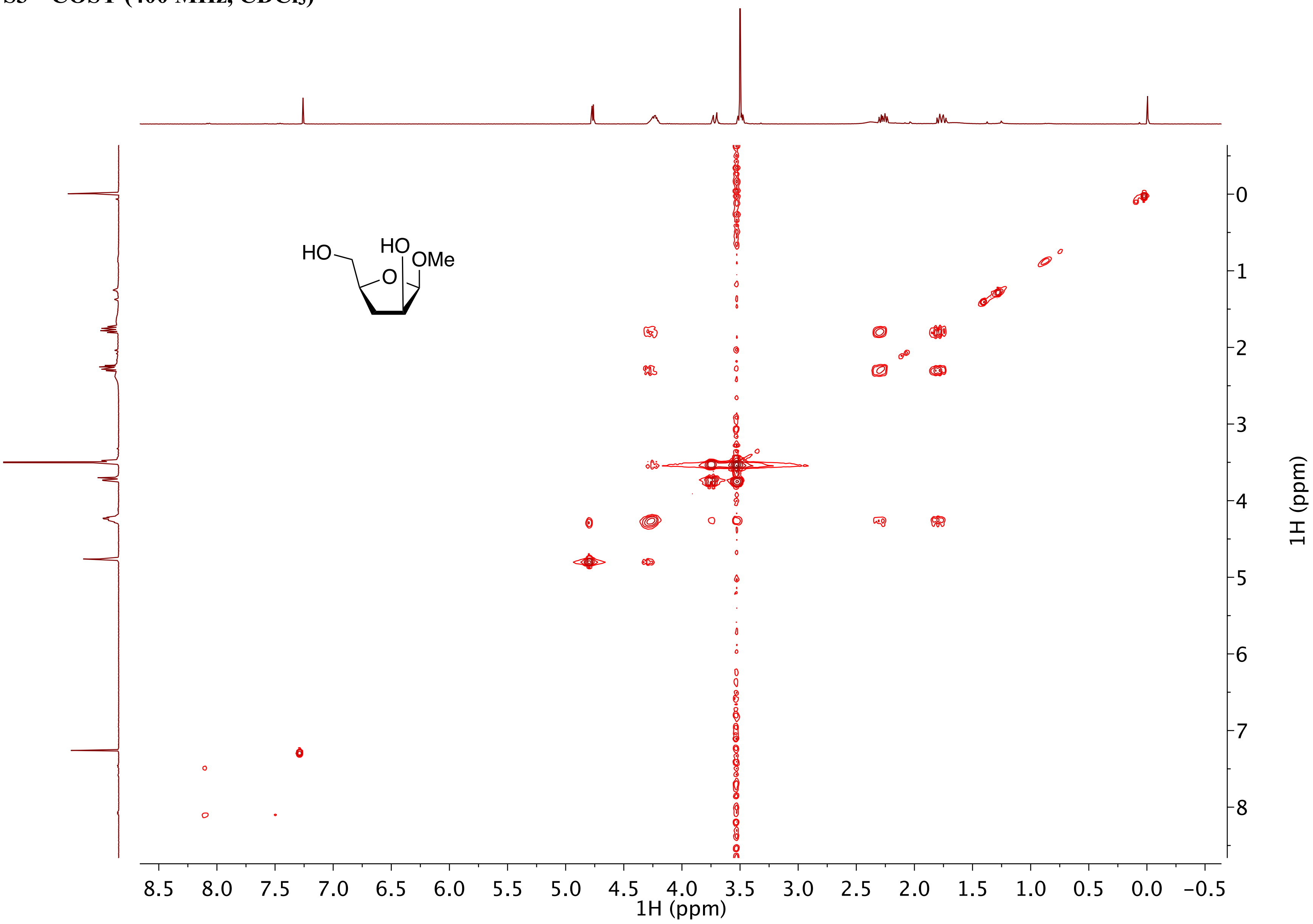

S4 –^{1}H NMR (400 MHz, CD3CN)

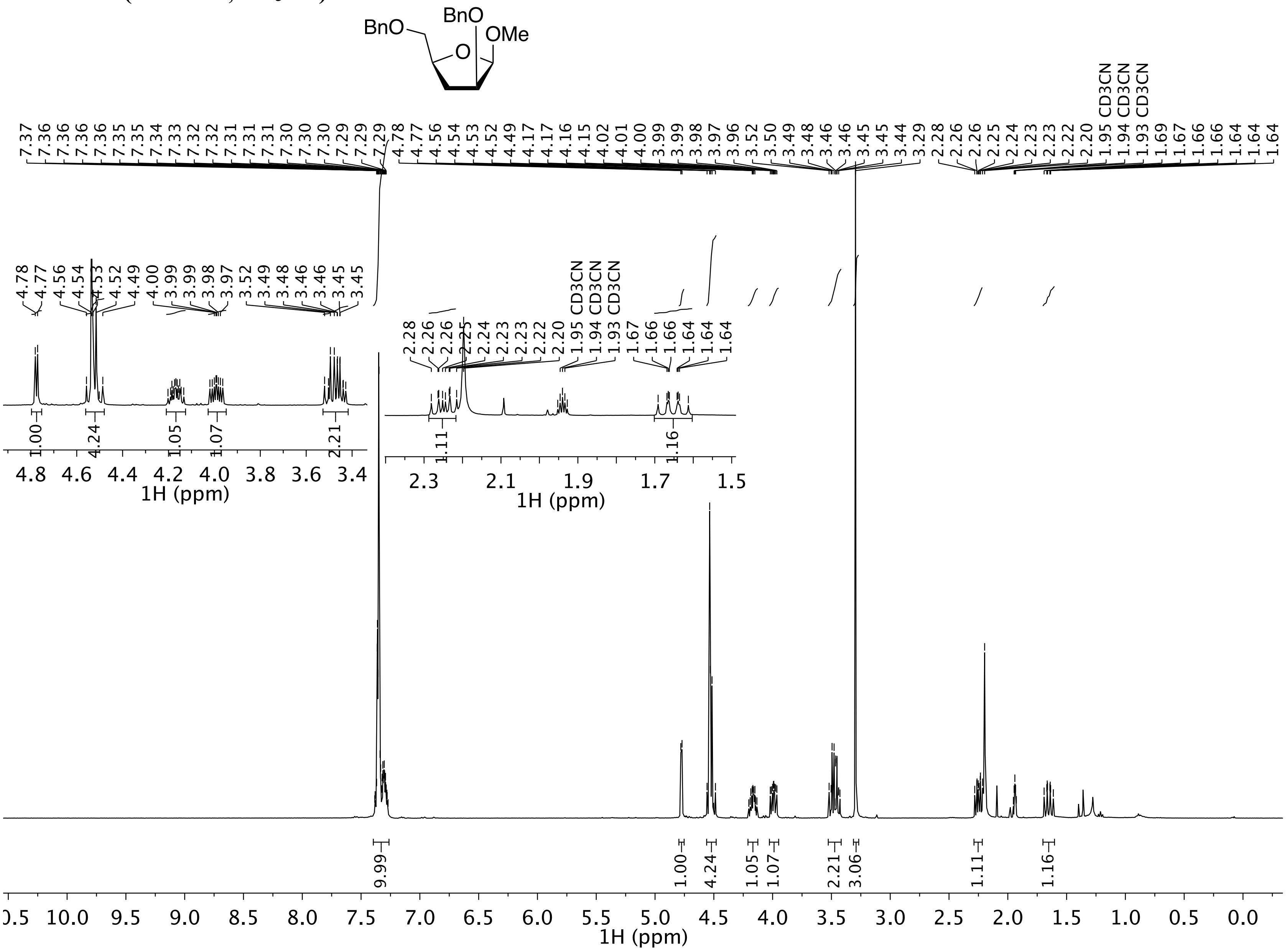

S4 –^{13}C NMR (101 MHz, CD_3CN)

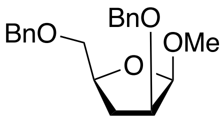

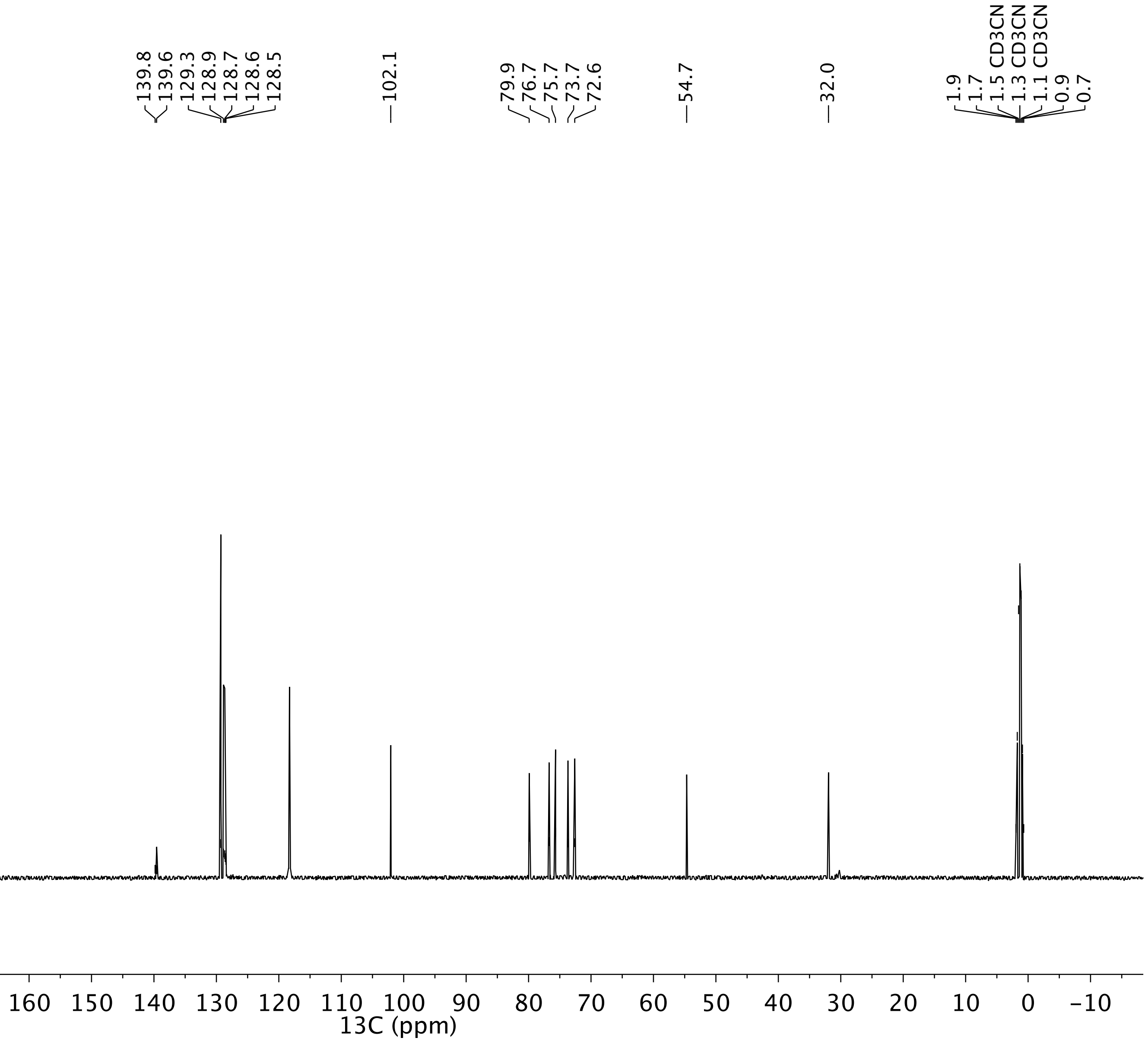

S4 –COSY (400 MHz, CD_3CN)

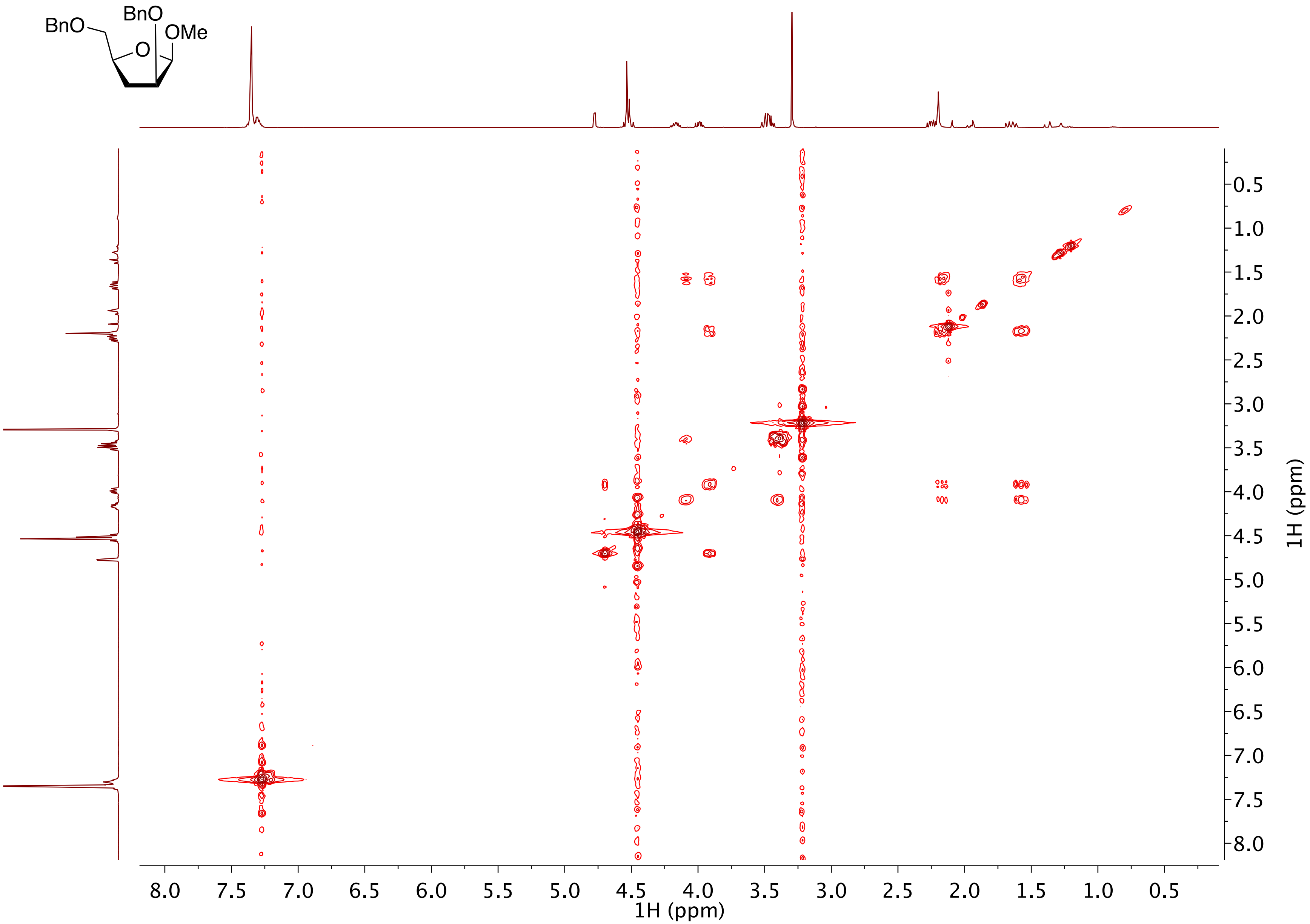

S5 –^{1}H NMR (400 MHz, CD_3CN)

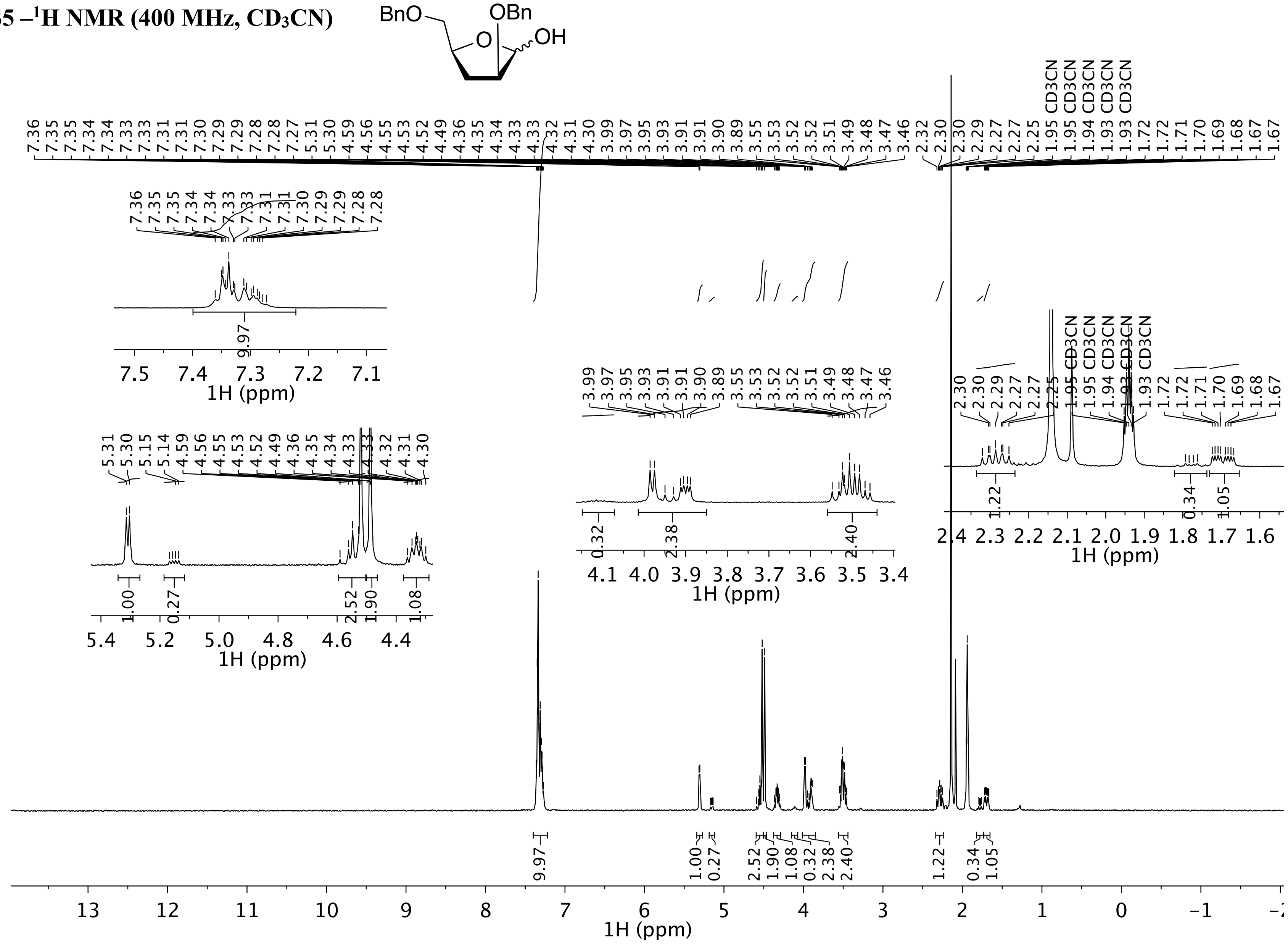

S5 –^{13}C NMR (101 MHz, CD_3CN)

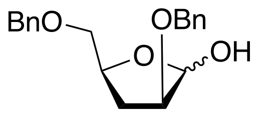

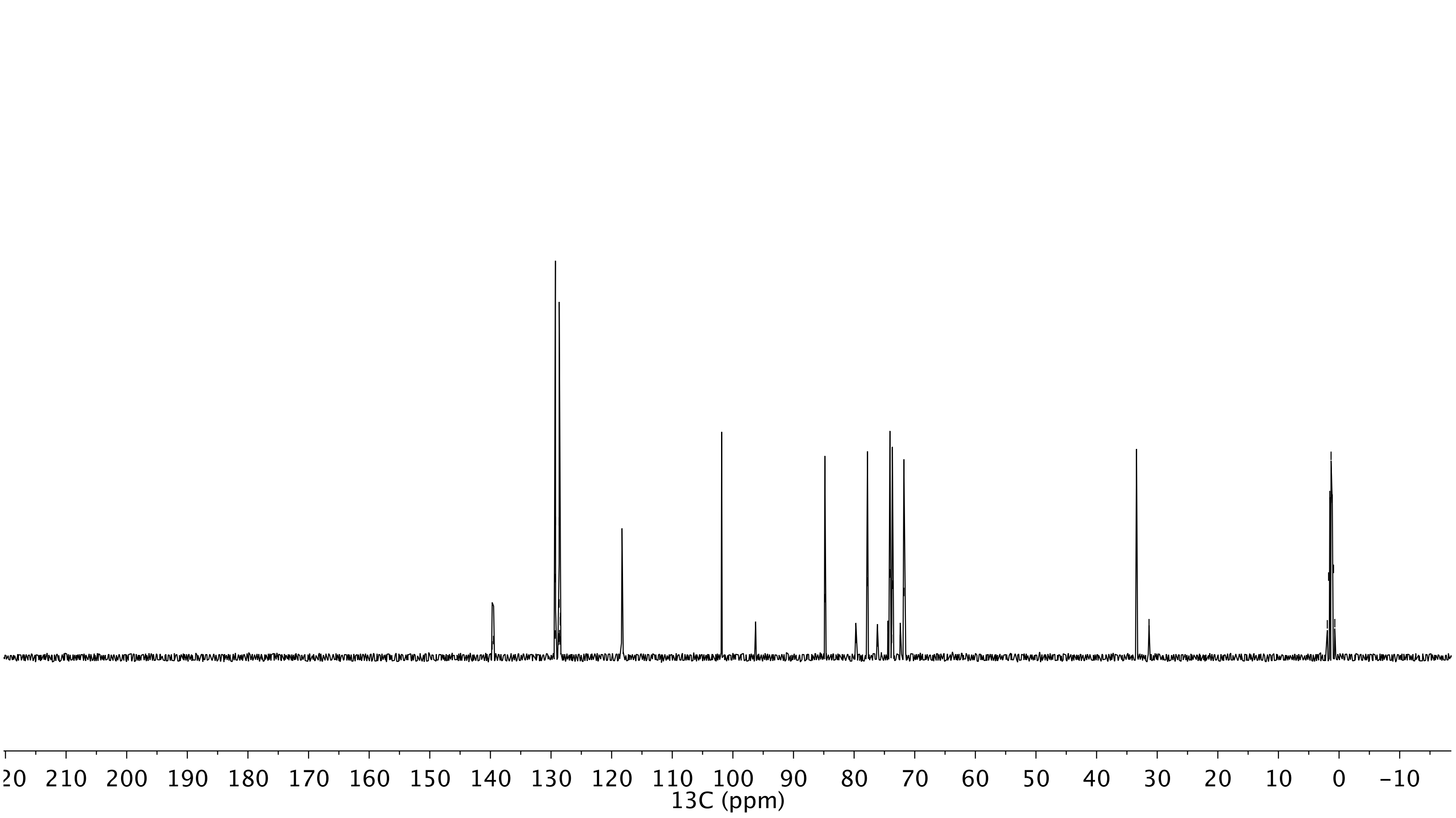

S5 –COSY (400 MHz, CD_3CN)

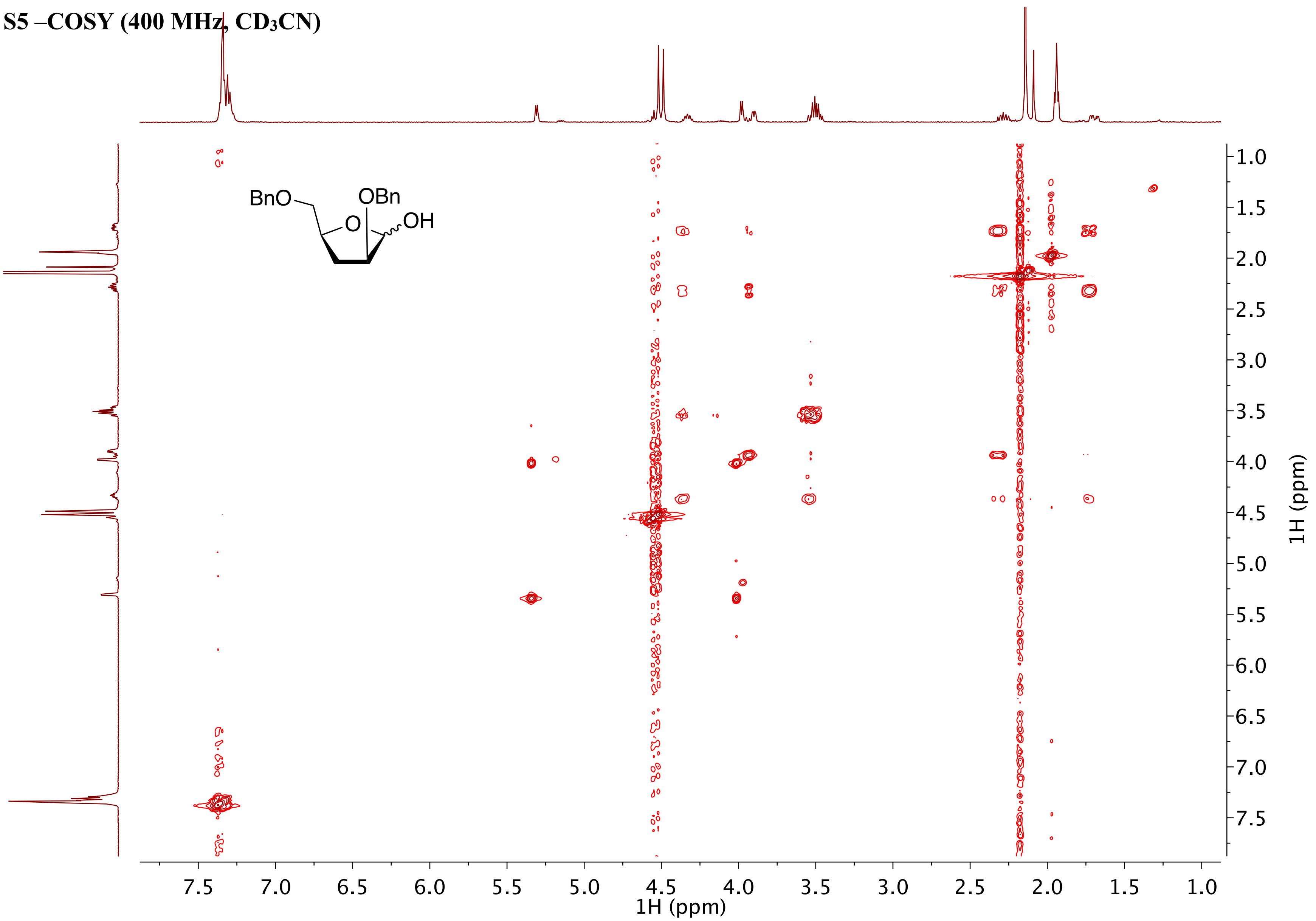

6 –^{1}H NMR (400 MHz, CD_3CN)

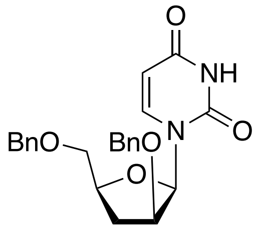

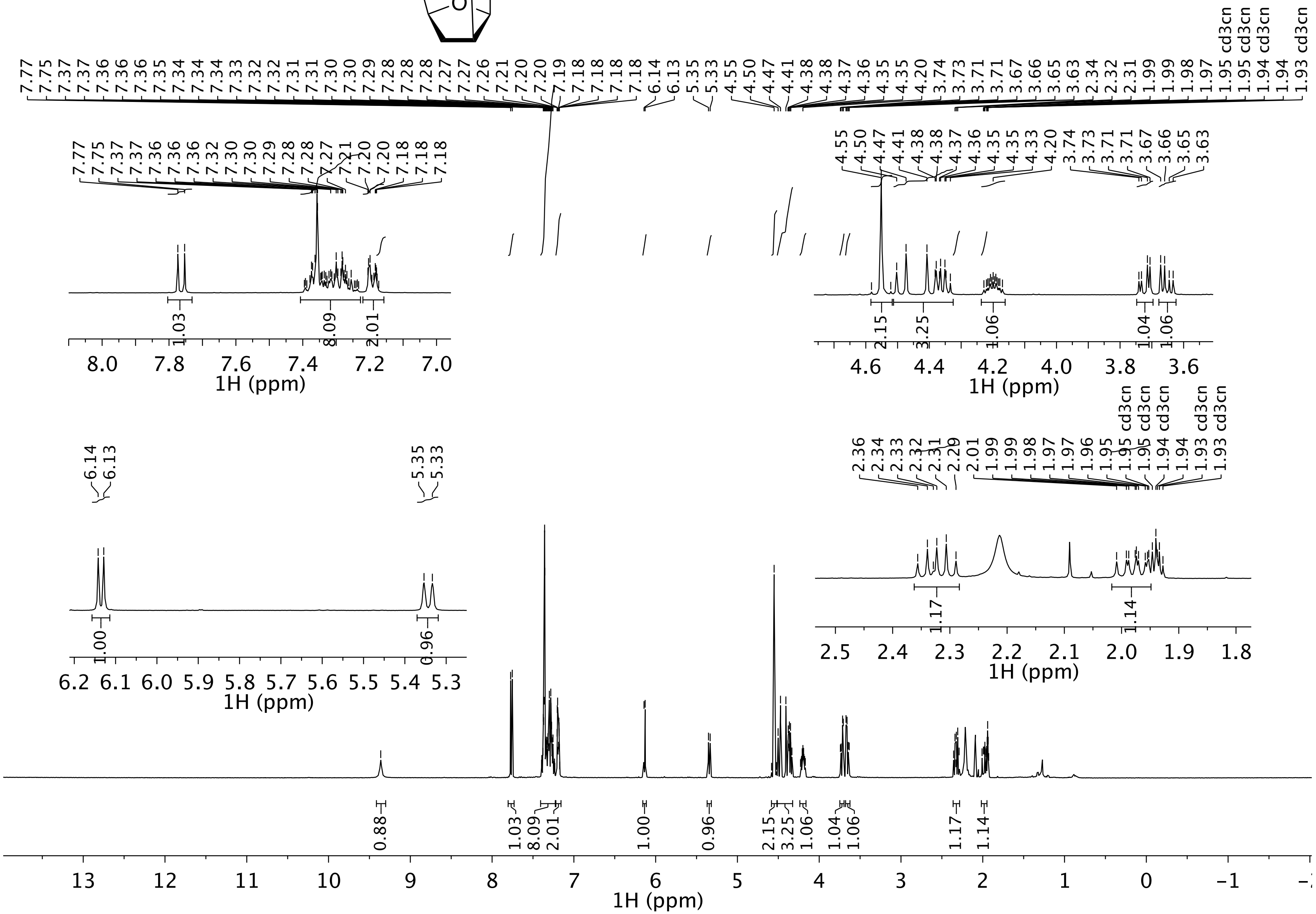

6 –^{13}C NMR (101 MHz, CD_3CN)

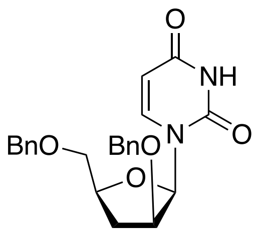

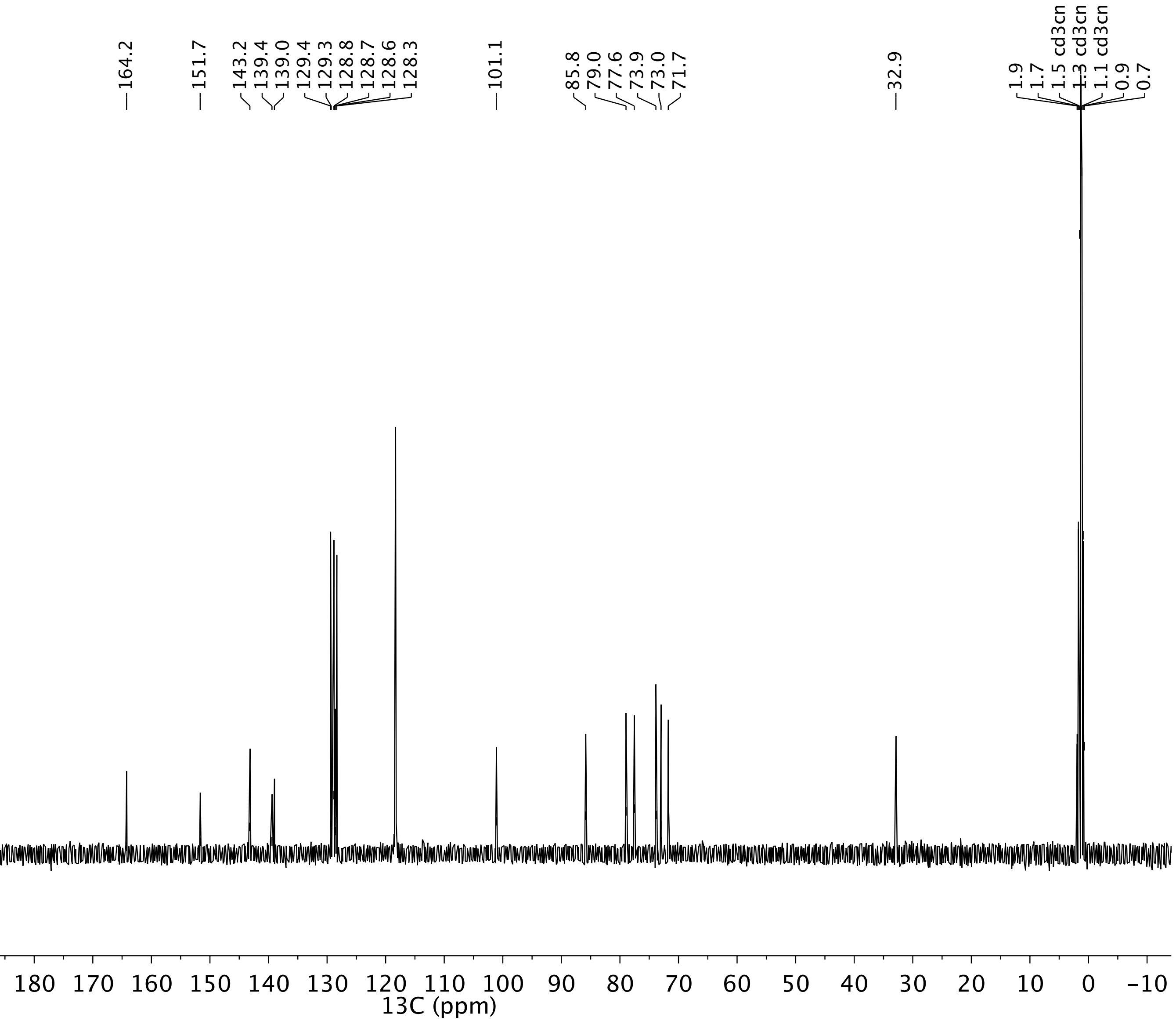

6 –COSY (400 MHz, CD_3CN)

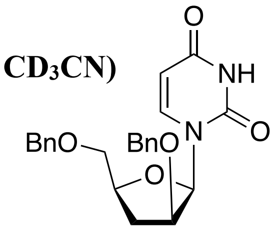

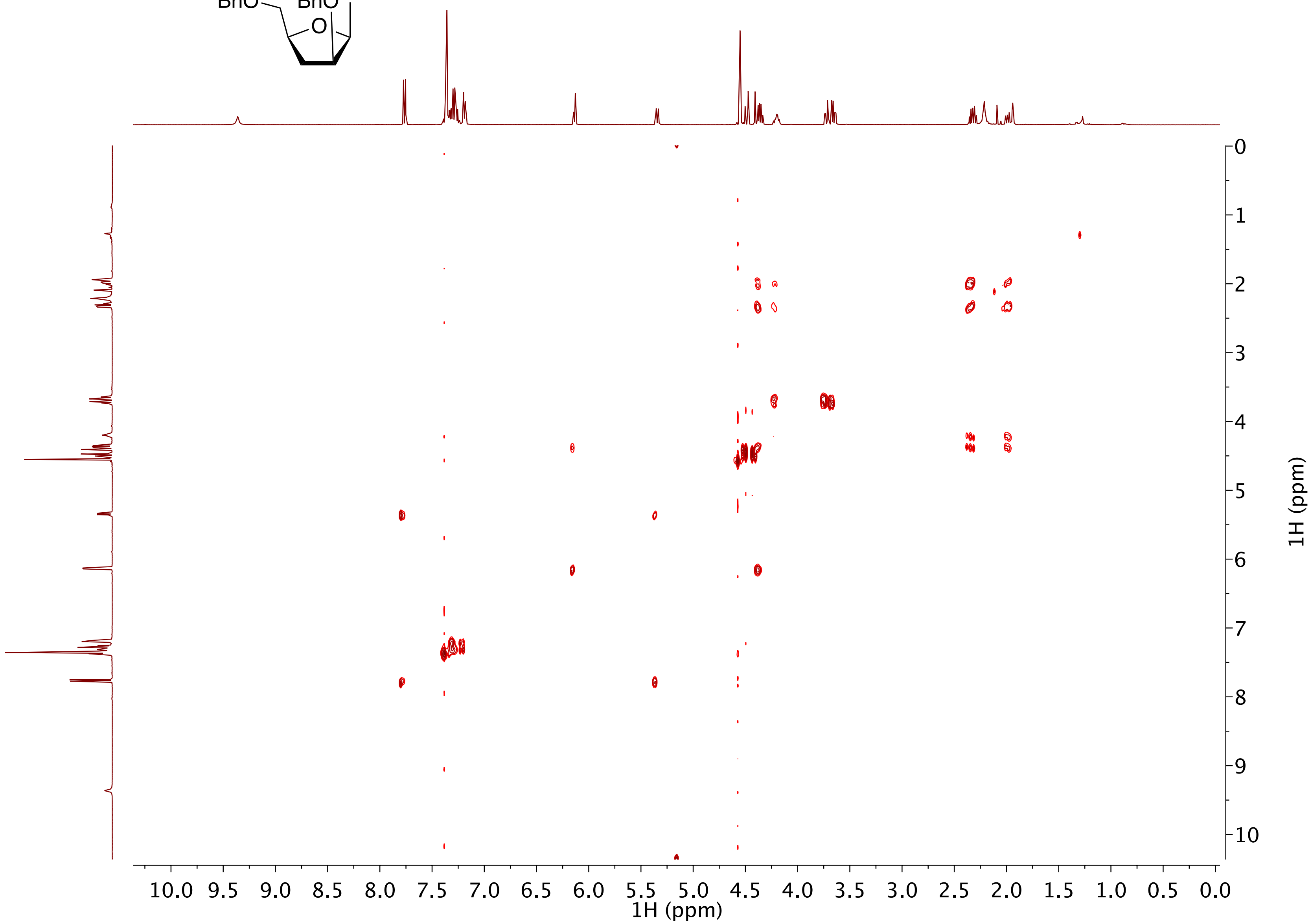

6 –HSQC (400 MHz, CD_3CN)

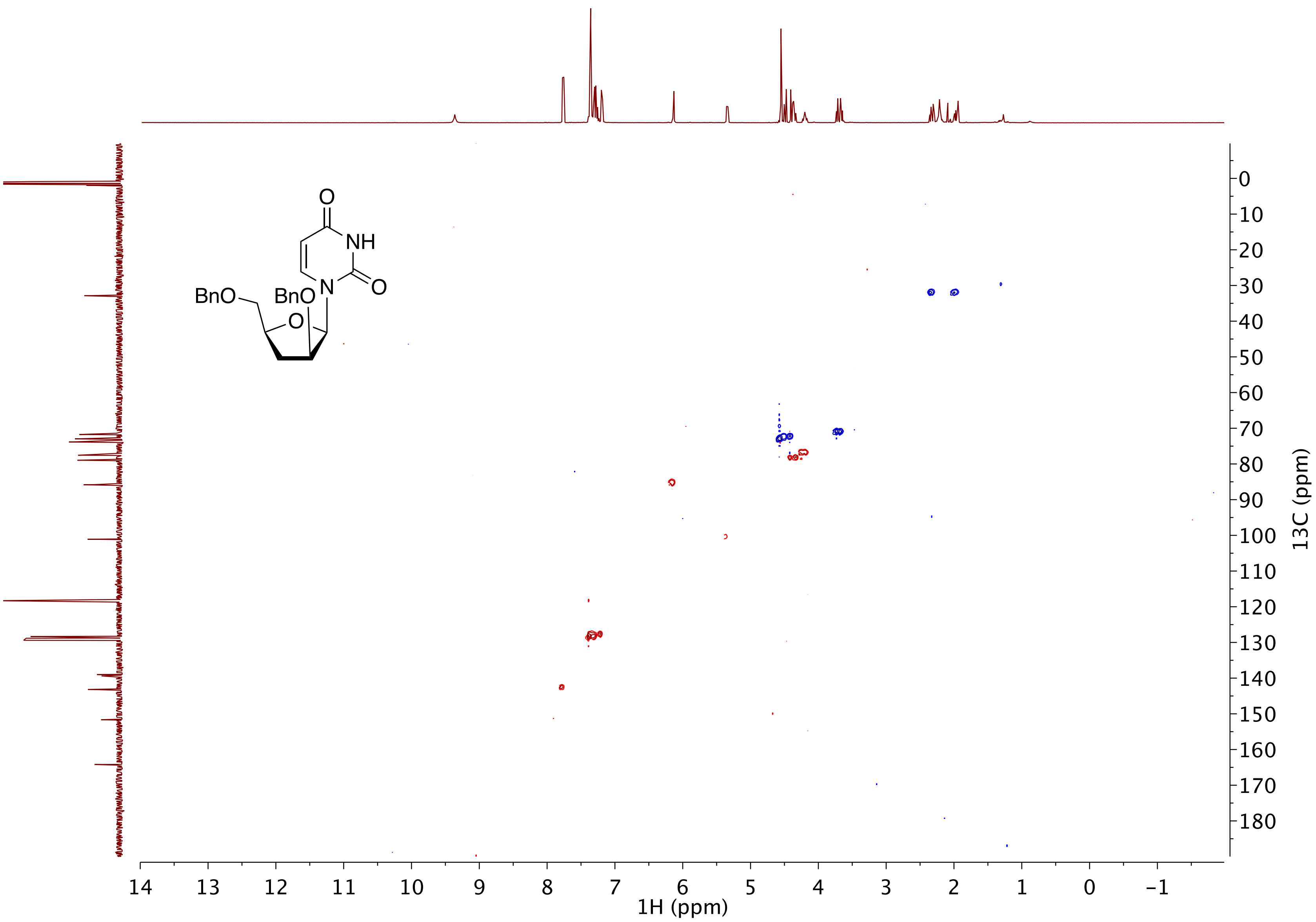

6 –HMBC (400 MHz, CD_3CN)

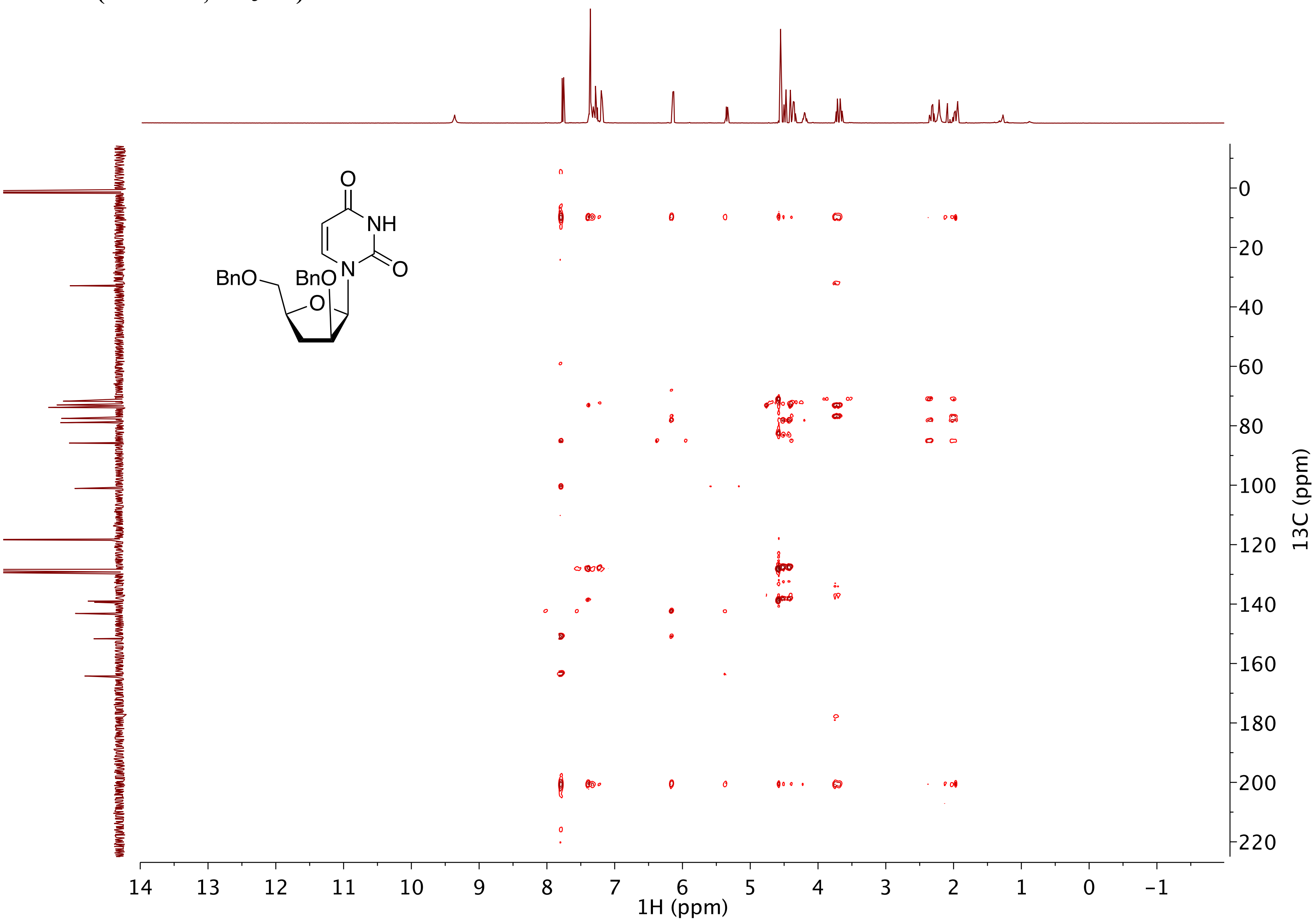

6 –NOESY (400 MHz, CD_3CN)

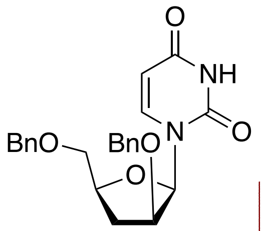

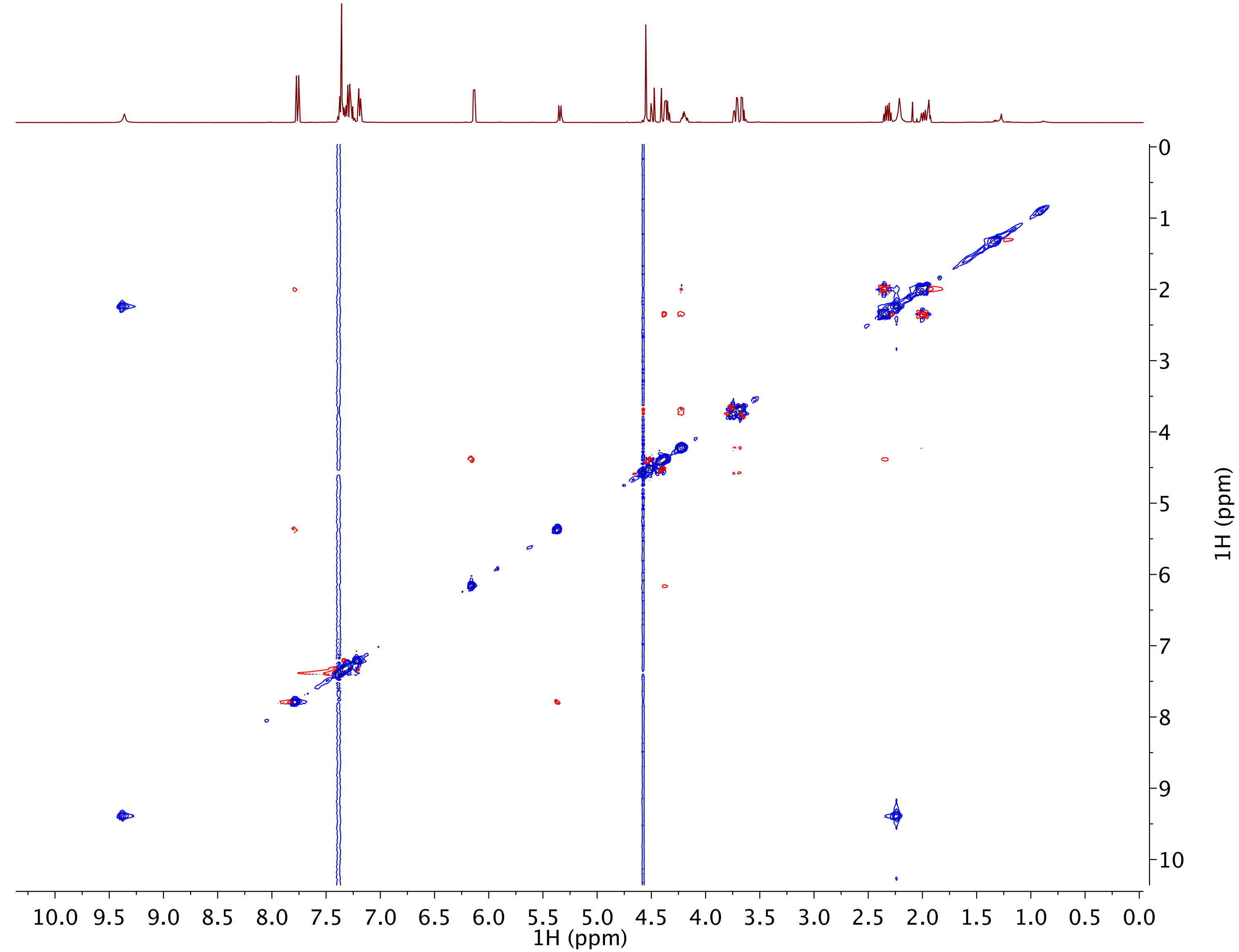

Supplement: SC-011-C9SC05173B-s001 [file SC-011-C9SC05173B-s001.pdf]
